# Proteomic Analysis in Type 2 Diabetes Patients before and after a Very Low Calorie Diet Reveals Potential Disease State and Intervention Specific Biomarkers

**DOI:** 10.1371/journal.pone.0112835

**Published:** 2014-11-21

**Authors:** Maria A. Sleddering, Albert J. Markvoort, Harish K. Dharuri, Skhandhan Jeyakar, Marieke Snel, Peter Juhasz, Moira Lynch, Wade Hines, Xiaohong Li, Ingrid M. Jazet, Aram Adourian, Peter A. J. Hilbers, Johannes W. A. Smit, Ko Willems Van Dijk

**Affiliations:** 1 Departments of General Internal Medicine and Endocrinology & Metabolism, Leiden University Medical Center, Leiden, The Netherlands; 2 Human Genetics, Leiden University Medical Center, Leiden, The Netherlands; 3 Department of Biomedical Engineering, Eindhoven University of Technology, Eindhoven, The Netherlands; 4 BG Medicine Inc., Waltham, Massachusetts, United States of America; German Diabetes Center, Leibniz Center for Diabetes Research at Heinrich Heine University Duesseldorf, Germany

## Abstract

Very low calorie diets (VLCD) with and without exercise programs lead to major metabolic improvements in obese type 2 diabetes patients. The mechanisms underlying these improvements have so far not been elucidated fully. To further investigate the mechanisms of a VLCD with or without exercise and to uncover possible biomarkers associated with these interventions, blood samples were collected from 27 obese type 2 diabetes patients before and after a 16-week VLCD (Modifast ∼450 kcal/day). Thirteen of these patients followed an exercise program in addition to the VCLD. Plasma was obtained from 27 lean and 27 obese controls as well. Proteomic analysis was performed using mass spectrometry (MS) and targeted multiple reaction monitoring (MRM) and a large scale isobaric tags for relative and absolute quantitation (iTRAQ) approach. After the 16-week VLCD, there was a significant decrease in body weight and HbA1c in all patients, without differences between the two intervention groups. Targeted MRM analysis revealed differences in several proteins, which could be divided in diabetes-associated (fibrinogen, transthyretin), obesity-associated (complement C3), and diet-associated markers (apolipoproteins, especially apolipoprotein A-IV). To further investigate the effects of exercise, large scale iTRAQ analysis was performed. However, no proteins were found showing an exercise effect. Thus, in this study, specific proteins were found to be differentially expressed in type 2 diabetes patients versus controls and before and after a VLCD. These proteins are potential disease state and intervention specific biomarkers.

**Trial Registration:**

Controlled-Trials.com ISRCTN76920690

## Introduction

The incidence of insulin resistant states, such as the metabolic syndrome and type 2 diabetes (T2DM), has increased dramatically in recent years [1],[2]. T2DM is a chronic multifactorial disease characterized by insulin resistance of the liver, skeletal muscle and adipose tissue and the progressive failure of pancreatic β-cells [3],[4]. Furthermore, research has shown that T2DM is associated with inflammation, oxidative stress and vascular dysfunction [3],[5].

Over 80% of T2DM patients is overweight or obese [6],[7], nevertheless T2DM develops in only about one-third of obese, insulin-resistant individuals. Simultaneously, some 30% of obese (BMI>30) individuals seem metabolically healthy. Whether these patients are protected from, or merely have a delayed risk for developing T2DM is not known [8],[9]. Because of the contribution of obesity to insulin resistance, it is essential for obese T2DM patients to reduce body weight. The most fundamental aspect of the treatment of obesity is life-style change, i.e. reduction of caloric intake and increase of physical activity. Very low calorie diets (VLCD) have been shown to lead to a substantial amount of weight loss and subsequently result in major metabolic improvements in obese T2DM patients [10]. Recently, we have shown that a 16-week VLCD in T2DM patients leads to a decrease in pericardial fat volume and an increase in quality of life (QoL) [11],[12]. In addition, adding an exercise program to the VLCD in these patients has been shown to have moderate additional favorable effects [13].

In the past decade large scale proteome analysis, also referred to as ‘proteomics’, has been used to identify new biomarkers for the risk prediction of various diseases, such as cancer, Alzheimer's disease, cardiovascular disease and diabetes. Proteomics can also be used to further elucidate disease mechanism and molecular processes and to investigate the response of the body to interventions [14],[15]. In diabetes research, proteomics have been analysed in various bodily fluids, cell-lines and tissues, such as blood, urine, saliva, semen, vitreous fluid, β-cells, adipocytes, hepatocytes and skeletal muscle [16]–[22]. However, most of the proteomics studies are cross-sectional and there are currently no studies on proteomic analysis in obese T2DM patients before and after a diet, the hallmark of their treatment.

To gain more insight into the pathophysiology of type 2 diabetes we performed plasma proteomics on the obese T2DM patients, before and after a VLCD with or without exercise, for which clinical and metabolic improvements after the VLCD were published before. [11]–[13],[23]–[25] Furthermore, we compared these T2DM patients before and after the diet with obese and lean controls. Because of the drastic weight loss and major improvements in glycemic control after such a diet, we hypothesized that differences in proteins can be found that might be involved in the development of, and recovery from, T2DM. By comparing the patients to controls, we aim to uncover proteins differentially expressed in T2DM patients as compared to lean and obese controls, and changes in these differences after the intervention. In addition, by comparing the groups with and without exercise, we aim to uncover possible biomarkers associated with the additional favorable effects of adding an exercise program to the VLCD. Firstly, we conducted a targeted MRM analysis of 13 abundant proteins hypothesized to be associated with T2DM and obesity, including apolipoproteins and markers of inflammation and coagulation. Subsequently, we performed a large scale iTRAQ analysis in samples of the T2DM patients before and after the diet to uncover differences between the VLCD with and without exercise groups also for less abundant proteins.

## Materials and Methods

### Patients

**Figure 1:**
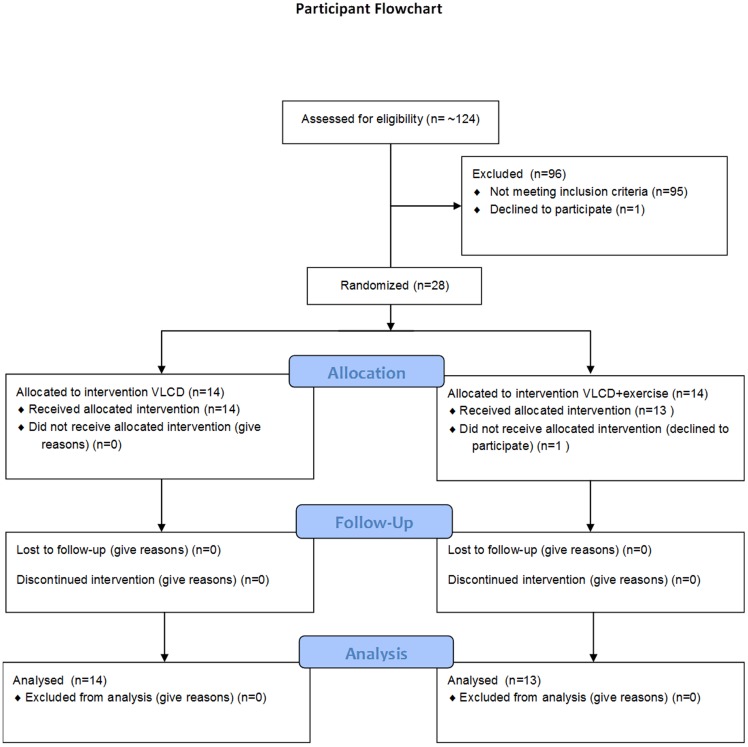
Participant flowchart.

The protocol of this study has been described previously [13]. In short, twenty-seven (14 men, 13 women) T2DM patients were included in the study (Figure 1). Diabetes duration was 8.9±0.8 years (mean ±SEM) and patients were obese with an average BMI of 37.2±0.9 kg/m^2^. All patients were on insulin therapy (average insulin dose 82±11 units/day) with or without additional oral glucose-lowering medication. Smoking, recent weight change (past 3 months), a history of cardiovascular disease or any other chronic disease were reasons for exclusion.

Two control subjects were recruited via advertisements for every T2DM patient, one lean and one obese subject. Control subjects were matched for gender, age, race and geographical area. In addition, obese control subjects were matched for BMI as well. Clinical characteristics are shown in Table 1.

**Table 1:** Clinical characteristics of type 2 diabetes patients before and after a 16-week VLCD +/− exercise and obese and lean controls.

	VLCD + excercise								VLCD only								controls						
	baseline				16 weeks				baseline				16 weeks				obese				lean		
Sex (M/F)	8/5								6/8								14/13				14/13		
Age (years)	53	±	2						56	±	2						55	±	2		56	±	2
Weight (kg)	113.5	±	5.1	*	86.3	±	4.2	# * †	112.7	±	5.6	*	89.0	±	4.3	# * †	117	±	4	*	73	±	2
BMI (kg/m^2^)	36.4	±	1.1	*	27.7	±	1	# * †	37.9	±	1.4	*	30.0	±	1.1	# * †	38.8	±	1.2	*	24.2	±	0.4
Waist (cm)	123	±	3	*	98	±	3	# * †	122	±	3	*	103	±	3	# * †	120	±	2	*	88	±	2
Fat mass (kg)	45.4	±	3.2	*	23.5	±	2.2	# * † ^$^	49.9	±	3.6	*	33.2	±	2.8	# †	43.0	±	3.4		35.3	±	3.1
Systolic BP (mmHg)	145	±	5	$	132	±	5	^#^ †	160	±	4	*	140	±	4	^#^ †	153	±	4	*	139	±	4
Diastolic BP (mmHg)	81	±	3	†	75	±	2	†	87	±	3		78	±	2	^#^ †	89	±	2	*	81	±	2
Glucose (mmol/L)	10.9	±	0.7	* †	6.6	±	0.8	# *	12.1	±	0.5	* †	7.7	±	0.6	# * †	5.3	±	0.2	*	4.8	±	0.1
Insulin (mU/L)	25	±	2.2	* †	9	±	0.8	* † ^#^	24	±	4.3	* †	13	±	2	# *	13	±	1.1	*	5	±	0.7
HbA1c (%)	7.8	±	0.4	* †	6.3	±	0.4	* #	7.8	±	0.3	* †	6.7	±	0.3	# * †	5.5	±	0.1	*	5.2	±	0.04
Total cholesterol (mmol/L)	5.4	±	0.4		4.5	±	0.3	# * † ^$^	6.1	±	0.4		5.5	±	0.3		6.2	±	0.2		6.2	±	0.2
Triglycerides (mmol/L)	2.5	±	0.5	*	1.2	±	0.1	^#^ †	2.3	±	0.2	* †	1.5	±	0.2	# *	1.7	±	0.2	*	1.1	±	0.1
LDL cholesterol (mmol/L)	3.6	±	0.3		3.0	±	0.2	# * †	4.4	±	0.4		3.7	±	0.3		4.0	±	0.2		3.8	±	0.2
HDL cholesterol (mmol/L)	1.1	±	0.0	* †	1.2	±	0.1	* †	1.2	±	0.1	* †	1.2	±	0.1	*	1.4	±	0.1	*	1.8	±	0.1
Diabetes duration (years)	7.9	±	1.2						9.8	±	1.1						N/A				N/A		
Insulin dose (units/day)	77				0				86				0				N/A				N/A		
Metformin (n)	10				0				9				0				N/A				N/A		
SU-derivatives (n)	3				0				1				0				N/A				N/A		

(These data were previously published REF VLCD, VLCD QoL, VLCD inflamm). Mean ± SEM, unless otherwise specified.

# Significant difference within group vs. baseline;

*significant difference vs. lean controls;

† significant difference vs. obese controls;

$ significant difference VLCD only vs. VLCD+exercise. BMI: body mass index; BP: blood pressure; HDL: high density lipoprotein; LDL: low density lipoprotein.

### Ethics statement

This study was conducted in accordance with the Declaration of Helsinki. The study protocol was approved by the local ethics committee (Commissie Medische Ethiek, Leiden University Medical Center) and written informed consent was obtained from all subjects. The study was registered under ISRCTN76920690 (http://www.controlled-trials.com/isrctn/). The study was conducted between 2006 and 2009. The proteomics analysis was performed in 2010–2011. The proteomic analysis was not planned when the study was approved by the ethics committee, but was added later. The proteomics protocol is described in detail below.

### Study design

All T2DM patients followed a VLCD for a period of 16 weeks. We randomly assigned 13 of the 27 patients to simultaneously follow an exercise program. All patients were provided with the same instruction forms and were all willing to be randomized to either intervention. We then assigned the first 13 fit candidates to the VLCD with exercise intervention. The following fit candidates were assigned to the VLCD-only intervention. The patients were not aware of the randomization order. Patients were studied before and after the VLCD intervention. Oral glucose-lowering medication was discontinued three weeks before the start of the study and insulin therapy was stopped the day before. During the 16-week intervention period, all glucose-lowering medication, including insulin, remained discontinued.

### VLCD

The VLCD consisted of three sachets of Modifast (Nutrition & Santé, Antwerp, Belgium), containing a total of 450 kcal per day. It provides about 50 to 60 grams of carbohydrate, 50 grams of protein, 7 to 9 grams of lipid, 10 grams of dietary fibers and all necessary vitamins and micronutrients. During the whole intervention period, patients visited the outpatient clinic weekly for measurement of body weight, to check glucoregulation and to confirm compliance with the diet.

### Exercise program

Thirteen of the 27 T2DM patients simultaneously participated in an exercise program. This program comprised a minimum of 4 days training at home for 30 min at 70% of maximum aerobic capacity on a cyclo-ergometer. Furthermore, patients participated in a weekly one-hour aerobic exercise training under supervision of a physiotherapist. Compliance was assessed by reading the heart rate monitor worn during exercise sessions both at home and in the hospital (Polar S610 _i_^tm^, Polar Electro Oy, Finland). Patients in the VLCD-only group were instructed to maintain their normal pattern of physical activity during the study.

### Anthropometric and laboratory measurements

At baseline and after the 16-week intervention period patients were studied after an overnight fast and after 2 days without any exercise. All T2DM patients completed the 16-week VLCD and no patients were lost to follow-up. The lean and obese control subjects were studied only once.

Height, weight, BMI and waist circumference were measured according to the World Health Organization recommendations. Blood pressure was measured with an Omron 705IT blood pressure device (Omron Matsusaka Co., Ltd., Japan) and recorded within the limits of 1 mmHg. Fat mass was assessed by bioelectrical impedance analysis (BIA, Bodystat 1500 MDD, Bodystat Ltd., Douglas, Isle of Man, United Kingdom). Blood samples were drawn for the measurement of fasting plasma levels of glucose, insulin, hemoglobin A1c (HbA1c), total cholesterol (TC), high density lipoprotein (HDL)-cholesterol, low density lipoprotein (LDL)-cholesterol and triglycerides (TG).

### Proteomics measurements

#### Targeted protein assays through multiple reaction monitoring (MRM)

Ten µL of plasma aliquots were processed in 1.5-mL screw cap tubes. One hundred ninety five µL of 100 mM TEAB/2M urea/10% acetonitrile/1% *n*-octyl-glucoside/10 mM TCEP was added to the plasma samples. Samples were incubated at room temperature for one hour for complete reduction. Four µL of 0.5 M iodoacetamide (Sigma-Aldrich) was added and alkylation was completed for 30 minutes at room temperature. Forty µL of each aliquot of reduced/alkylated plasma sample was digested with 12 µg sequencing grade trypsin. Digestion was stopped after overnight incubation at room temperature by adding 45 µL of 2 M urea/1% formic acid. To monitor LC/MS instrument trending, 0.3 µg fibrinopeptide A standard (AnaSpec, Fremont, CA) was spiked into each sample vial. Twenty µL of digested samples were injected for quantitative analysis.

LC-MRM analysis was performed on 4000QTrap instrument (AB/SCIEX, Concord, ON) interfaced with a U3000 HPLC system (Dionex, Sunnyvale, CA). Peptides were separated on a Targa C18 (5 µm) 150×1.0 mm column (Higgins Analytical, Mountain View, CA) utilizing a 200-µL/min flow rate. Peptides were eluted carried out over a 21-min gradient from 2% B to 32%B (A: 5% acetonitrile, 0.1% formic acid, B: 95% acetonitrile, 0.1% formic acid). The HPLC column compartment was kept at 50°C during analysis.

Two peptides and two fragments from each were carefully selected to represent the target proteins to be assayed. Thirteen target proteins were analyzed: apolipoproteins A-I, A-IV, B100, C-III, E, Beta-2-glycoprotein 1 alpha-I-antitrypsin, complement C3, fibrinogen alpha, beta, gamma chains, alpha-1-acid glycoprotein and transthyretin. Accession numbers of these proteins are given in Table 2, while Table S2 in File S1 shows the used peptide sequences.

**Table 2:** VLCD effect for proteins in the MRM dataset as compared to obese and lean controls.

	T2DM								controls						
Protein description (Accession number)	baseline				16 weeks				obese				lean		
Alpha-1-acid glycoprotein 1 (P02763)	1.02	±	0.08		0.91	±	0.05		1.06	±	0.07		0.84	±	0.06
Alpha-1-Antitrypsin (P01009)	1.01	±	0.03		1.11	±	0.04	# * †	0.95	±	0.05		0.97	±	0.04
Apolipoprotein A-I (P02647)	0.95	±	0.03	*	0.88	±	0.04	* †	1.03	±	0.05		1.17	±	0.06
Apolipoprotein A-IV (P06727)	1.33	±	0.08	* †	0.71	±	0.06	# * †	1.04	±	0.06		1.06	±	0.06
Apolipoprotein B-100 (P04114)	1.20	±	0.07	* †	1.00	±	0.05	#	0.98	±	0.04		0.92	±	0.04
Apolipoprotein C-III (P02656)	1.36	±	0.14	*	0.85	±	0.05	#	1.02	±	0.07		0.87	±	0.06
Apolipoprotein E	1.24	±	0.10	*	0.96	±	0.05	#	1.01	±	0.04		0.88	±	0.05
(P02649)															
Beta-2-glycoprotein 1 (P02749)	1.10	±	0.03	* †	1.02	±	0.04		0.97	±	0.05		0.94	±	0.04
Complement C3	1.17	±	0.03	*	0.97	±	0.04	# *	1.08	±	0.04	*	0.85	±	0.04
(P01024)															
Fibrinogen alpha chain (P02671)	1.04	±	0.05	* †	1.08	±	0.05	* †	0.87	±	0.05		0.79	±	0.09
Fibrinogen beta chain (P02675)	1.06	±	0.04	* †	1.12	±	0.05	* †	0.91	±	0.05		0.82	±	0.07
Fibrinogen gamma chain (P02679)	1.06	±	0.04	* †	1.13	±	0.05	* †	0.89	±	0.04		0.82	±	0.07
Transthyretin	0.87	±	0.04	* †	0.85	±	0.04	* †	1.07	±	0.06		1.04	±	0.04
(P02766)															

The numerical entries represent ratio measurements relative to a pooled reference sample.

Mean ± SEM.

# significant difference within group *vs.* baseline;

*significant difference *vs.* lean controls;

† significant difference *vs.* obese controls.

Specimens from all T2DM and control subjects (114 samples) were analyzed in three acquisition batches. Primary samples (following every four) were interleaved with QC reference samples. MRM signals (ion intensities of fragments) from the primary samples were normalized to the median signal from the same fragments in the QC samples. This accurate relative quantification could be achieved without the need of using isotope labeled peptide standards. MRM signals were integrated using the Multiquant v1.1 software tool (AB/SCIEX).

#### iTRAQ Discovery Proteomics

Proteomic analysis was carried out by utilizing the 8-plex iTRAQ reagent for relative quantification [26]. In this workflow a single 2D LC-MS/MS experiment is used for the quantification of peptides (and proteins) from up to eight samples. Eight-plex experiments were configured to profile six primary samples and two replicates of reference (QC) sample that was created by combining a fraction of the primary samples. By normalizing peptide measurements from the primary samples to those in the QC samples it is feasible to compare large numbers of primary samples analyzed in different experiments. The study - 54 primary samples, 18 reference QC samples - consisted of nine such iTRAQ experiments.

One hundred µL plasma samples were delipidated by diluting with 400 µL 1XPBS (Sigma-Aldrich, St. Louis, MO) and 250 µL tetrachloroethylene (Sigma-Aldrich), vortexing thoroughly and spinning at 14,000 rpm for 10 minutes at 4°C. The resulting top aqueous phase was transferred to a new tube for further processing.

Abundant proteins were removed from delipidated plasma in two stages utilizing IgY14 5-mL and Supermix 2-mL columns (Sigma) on a Vision HPLC Workstation (Applied Biosystems, Foster City, CA) as described earlier [27]. The protein fraction corresponding to the depletion flow-through was recovered on a Poros R1 reversed-phase column, eluted with 95% acetonitrile and dried down in a SpeedVac. Only this fraction was used for discovery proteomics. Dried protein fractions were re-suspended in 22 µL 2 M urea, 1 M TEAB, 1% *n*-octyl-glucoside buffer (pH 8.5) and reduced with 5 mM TCEP for one hour at room temperature. Reduced samples were alkylated by adding 1 µL 84 mM iodoacetamide and incubating in the dark for 30 minutes at room temperature. Trypsin digestion was completed overnight at a 1∶10 enzyme/substrate ratio (w/w) at room temperature by adding 5 µL 1 mg/mL sequencing grade trypsin (Promega, Madison, WI) in 4 mM N-acetyl cysteine (to quench remaining iodoacetamide). Digested samples were labeled by the 8-plex iTRAQ reagents following the manufacturer's protocols (Applied Biosystems) using an amount of digest pool containing approximately 40 µg material. Primary samples were labeled with the reagents yielding the *m/z* 114, 115, 116, 118, 119, 121 reporter fragments in the MS/MS scans. QC samples (replicates from the reference pool) were labeled with the 113 and 117 reagents. iTRAQ labeling was quenched by the addition of 1 M ammonium bicarbonate.

Eight samples were combined to an iTRAQ mix, desalted, and fractionated by strong cation exchange (SCX) chromatography using a Poly Sulfoethyl Strong Cation Exchange Column (PolyLC, Columbia, MD) on an Agilent 1200 instrument (Agilent, Santa Clara, CA). Peptides were collected into nine SCX fractions through eluting with a gradient of 10 mM KH_2_PO_4_ to 10 mM KH_2_PO_4_/1M KCl at pH 3.5. SCX fractions dried and re-suspended in 50 µL 95:5:0.1 water-acetonitrile-trifluoroacetic acid (TFA) (Buffer A for HPLC). Reversed-phase separation was performed on a Dionex U3000 HPLC (Dionex, Sunnyvale, CA) with a 60-min gradient from 5% solvent B (10% H2O/90% ACN/0.1% TFA) to 38% B. Eleven-second HPLC fractions were collected onto MALDI plates through a Probot fraction collector (Dionex). MALDI matrix and mass calibration standard were co-infused with a syringe pump at 2-µl/min flow rate. MALDI plates were analyzed on an AB4800 mass spectrometer (Applied Biosystems/MDS SCIEX, Concord, ON, Canada) utilizing internally developed scripts for MS/MS precursor selection that was optimized to select and measure a reproducible set of peptides from each iTRAQ mix.

Peptide quantification was carried out by calculating the average iTRAQ ion intensity ratios relative to the m/z 113 and 117 peaks. Protein ratios were determined as the medians of all peptide ratios matching to the same protein. Peptide mappings are shown in Table S5 in File S1. Peptide sequences were identified from MS/MS fragmentation spectra using the Mascot search engine (Matrix Science, UK) the IPI sequence database (v3.72 of human sequences). For peptide matching trypsin specificity was used with up to two missed cleavage sites. iTRAQ modification, cysteine-alkylation, methionine oxidation, asparagine deamidation, and N-terminal pyro-Gly and pyro-cmc formation were considered as variable modifications. Precursor ion mass tolerance was 50 ppm and fragment ion tolerance was 0.4 Da. Peptide matches were validated by an internally developed procedure with an estimated rate of false peptide identification of less than 1%, as explained by Juhasz et al [27]. Once all the study samples were analyzed, the complete set of identified peptides was re-mapped to a minimum, non-redundant protein set through an internally developed procedure. During this process proteins that had unique peptides matching to them were kept separate from protein groups that shared peptides. Measured values of protein expression were normalized using a procedure based on Vandesompele et al [28].

### Assays

Plasma glucose, TC, HDL-cholesterol and TG concentrations were analyzed as previously described [13] with a fully automated P-800 module (Roche, Almere, The Netherlands). Serum insulin was measured with an immunoradiometric assay (Biosource, Nivelles, Belgium). HbA1c was detected with a semi automated HPLC machine Primus Ultra 2 (Kordia, Leiden, The Netherlands).

### Statistics

The data of the two intervention groups, i.e., both the clinical data and protein expression levels measured from the MRM as well as iTRAQ platforms, were studied using a linear mixed effect model for repeated measures in order to study the influence of the VLCD and the additional exercise program. The model was fitted by Maximum Likelihood (ML). The initial model included the random patient effects to account for the correlation between 2 repeated measures within the same patient, and age, gender, treatment and time as fixed effects, for each outcome variable separately. The model was tested for significance of each individual factor and the interaction effect of time and treatment. On doing so, it was found that the effects of age and gender were not significant. The final model consisted of the random patient effects, the fixed treatment and time effects, and the interaction between treatment and time. The influence of the VLCD was tested by studying the effect of time on the model and the additional influence of exercise with VLCD was tested by studying the effect of treatment and time interaction on the model. The p values for each of the tests are reported.

Differences between all groups in the clinical dataset as well as in the MRM dataset, i.e., two intervention groups and the lean and obese control groups, were analysed using t-tests, where paired t-tests were used when comparing two time points for the same group and independent t-tests for all other comparisons. Adjustment for multiple hypothesis testing has been performed in all proteomics analyses using the Benjamini-Hochberg (BH) method (unless otherwise stated in the text). A significance level of p = 0.05 was used (unless otherwise stated in the text). Data are presented as mean ±SEM. The statistical analyses were conducted using the free software R version 2.10.1 with the lme4 and multcomp libraries [29]–[31].

## Results

### Effect on body weight and glucoregulation

Anthropometric and laboratory results were published previously. [11]–[13],[23]–[25] As shown in Table 1, there were no significant differences in clinical characteristics, except for systolic blood pressure, between the VLCD+exercise and the VLCD-only group at baseline. Furthermore, the control groups were well matched with both intervention groups with respect to age and gender and for the obese control group with both intervention groups at baseline with respect to weight, BMI and waist circumference. Both control groups had significantly lower levels of glucose, insulin and HbA1c.

After the 16-week VLCD there was a significant decrease in body weight in both intervention groups (−27.2±1.9 kg VLCD+exercise; −23.7±1.6 kg VLCD-only). Patients also lost a significant amount of fat mass and waist circumference. Moreover, the 16-week VLCD resulted in an impressive improvement in glycaemic control as shown by a significant decrease in HbA1c in both treatment groups (VLCD + exercise 7.8±0.4 vs. 6.3±0.4%; VLCD-only 7.8±0.3 vs. 6.7±0.3%), despite the discontinuation of all glucose-lowering medication. In both treatment groups, plasma TG were significantly decreased to near normal values. After the 16-week intervention period the VLCD+exercise group had significantly less fat mass and a significantly lower total cholesterol level as compared to the VLCD-only group. There was no significant difference in glucoregulation between the groups after the 16-week intervention period (Table 1).

### Targeted MRM analysis

A total of 15 proteins, including 2 internal control proteins (not shown), were quantified using MRM and mass spectrometry in the VLCD groups, with and without exercise and before and after the intervention. These proteins were also quantified in the obese and lean controls.

#### Intervention effects

After 16 weeks, there was a significant decrease in concentrations of apolipoproteins A-IV, B-100, C-III and E as well as of Complement C3 in both intervention groups. These effects were however not significantly different between the two intervention groups (see Table S1 in File S1). Since no additional influence of exercise with VLCD was observed for any of the proteins in the MRM set, the VLCD+exercise and VLCD-only groups were combined for further analysis into one group of T2DM patients. Table 2 shows the full comparison of the combined T2DM group at the two time points (T2DM0 and T2DM16, respectively) with the two control groups (lean and obese).

Apolipoprotein A-IV showed the most significant effect of VLCD among all proteins considered in the MRM dataset. Apolipoprotein A-IV concentration did not differ between both control groups (lean 1.06±0.06 vs. obese 1.04±0.06 A.U., p = 0.90), however, the level for T2DM patients was significantly higher (1.33±0.08 A.U.) compared to both control groups (p = 0.01 for lean and p = 0.04 for obese) before the diet, whereas the level for T2DM patients was significantly lower to those of the controls (p = 0.002 for lean and p = 0.003 for obese) after the diet.

Also for apolipoproteins E, C-III and B-100 the concentration levels were significantly higher for T2DM patients at baseline compared to the lean control group, and VLCD resulted in significant decrease in their concentration levels. Contrary to Apolipoprotein A-IV, these decreased levels after the diet are not significantly different from the control groups.

#### Disease state discriminating proteins

**Obesity associated markers -** Only Complement C3 showed a significant difference between the lean control group and all three other groups. These differences were highly significant for lean against obese (0.85±0.04 vs 1.08±0.04 A.U., p = 0.001) as well as for lean against the T2DM group at baseline (0.85±0.04 vs 1.17±0.03 A.U., p<0.001). Upon the VLCD, the concentrations of C3 decreased (from 1.17±0.03 to 0.97±0.04 A.U., p<0.001), and, although still significantly different (p = 0.04), approached the concentration in the lean control group.

**Diabetes associated markers -** The fibrinogens alpha, beta and gamma chains all showed the same behaviour. Namely, all three showed a significantly increased level for T2DM patients as compared to both the lean and the obese controls, both at baseline and after 16 weeks of VLCD, although these increased levels for T2DM patients at baseline as compared to the obese were only just significant (p = 0.04 for all three). Moreover, all three showed no significant difference between obese and lean (p = 0.58 for all three) nor between the T2DM patients before and after the diet (p≥0.12).

Transthyretin showed a very similar behaviour as the fibrinogens except for the fact that the transthyretin level was lower for T2DM patients as compared to the controls.

### Large scale iTRAQ analysis

A total of 635 proteins were quantified using iTRAQ and mass spectrometry in the VLCD groups, with and without exercise and before and after the intervention. Only 234 of those proteins could be measured for all 27 patients on both time-points (i.e., at baseline and after the 16-week VLCD). These included two proteins added as internal controls. The data analysis was applied on the remaining 232 proteins.

#### Exercise associated markers

Of the 232 proteins 18 showed a significant exercise effect when considering the unadjusted p-value measured by the interaction of treatment and time from the model (see Table S3 in File S1). Amongst these, for two proteins SHBG and MASP-1, the p-value was lower than 0.005. For SHBG, the mean of the measurements for VLCD+exercise at 16 weeks showed a stronger increase from the measurements at baseline (VLCD+exercise at 16 weeks: 1.32±0.16 A.U. vs VLCD+exercise at baseline: 0.69±0.09 A.U.) in comparison to the increase for VLCD-only (VLCD-only at 16 weeks: 1.04±0.08 A.U. vs VLCD-only at baseline: 0.74±0.05 A.U.). For MASP-1, the level for the VLCD+exercise group was decreased after 16 weeks (VLCD+exercise at 16 weeks: 0.86±0.04 A.U. vs VLCD+exercise at baseline: 0.99±0.03 A.U.) whereas the level for the VLCD-only group hardly changed (VLCD at 16 weeks: 0.93±0.02 A.U. vs VLCD at baseline: 0.92±0.03 A.U.). However, on applying the multiple testing correction, none of the analytes were found to be significant.

#### VLCD associated markers

Of the 232 proteins, 87 showed a significant VLCD effect, where the effect is considered to be statistically significant if the unadjusted p-value from the model for the effect of time was less than 0.05 and the Benjamini-Hochberg-adjusted p-value was less than 0.10. Fourtysix proteins from these significant cases were up-regulated after treatment, i.e., the measured expression levels were higher after 16 weeks of VLCD than at baseline, while the other 41 proteins were down-regulated after 16 weeks of VLCD. The top 13 proteins (based on p-value) identified from iTRAQ experiments showing a VLCD effect are shown in Table 3. A list of all proteins and their changes after 16 weeks of VLCD are shown in Table S4 in File S1. Fourtyfour of the significantly changed proteins could be traced to pathways in KEGG, with 17 of them being present in the Complement and Coagulation cascade.

**Table 3:** Top 13 proteins identified from iTRAQ experiments showing a VLCD effect.

	T2DM						
Protein description (Accession number; number of peptides quantified)	Baseline			16 weeks			adj. p-value
Biotinidase (P43251; 13)	0.96	±	0.02	0.86	±	0.02	6.0E-07
Selenoprotein P (P49908; 7)	0.80	±	0.02	0.95	±	0.02	1.0E-06
Insulin-like growth factor-binding protein 2 (P18065; 6)	0.68	±	0.04	1.04	±	0.06	1.0E-06
Inter-alpha-trypsin inhibitor heavy chain H4 (Q14624; 41)	0.79	±	0.02	0.97	±	0.02	1.0E-06
Sex hormone-binding globulin (P04278; 11)	0.72	±	0.05	1.18	±	0.09	1.7E-06
Interleukin-1 receptor accessory protein (Q9NPH3; 7)	0.75	±	0.02	0.88	±	0.03	2.0E-06
Afamin precursor (P43652; 40)	0.95	±	0.04	0.78	±	0.03	2.6E-05
Apolipoprotein A-IV precursor (P06727; 31)	1.23	±	0.09	0.78	±	0.06	2.6E-05
Leucine-rich alpha-2-glycoprotein (P02750; 18)	0.72	±	0.02	0.92	±	0.04	2.7E-05
Beta-Ala-His dipeptidase (Q96KN2; 19)	1.12	±	0.05	0.91	±	0.03	3.4E-05
Lysozyme C (P61626; 5)	0.81	±	0.04	0.92	±	0.04	6.6E-05
Pigment epithelium-derived factor (P36955; 17)	1.22	±	0.05	0.95	±	0.04	6.8E-05
Fructose-bisphosphate aldolase B (P05062; 6)	1.05	±	0.06	0.80	±	0.03	2.5E-04

The numerical entries represent ratio measurements relative to a pooled reference sample.

Mean ±SEM.

## Discussion

**Figure 2:**
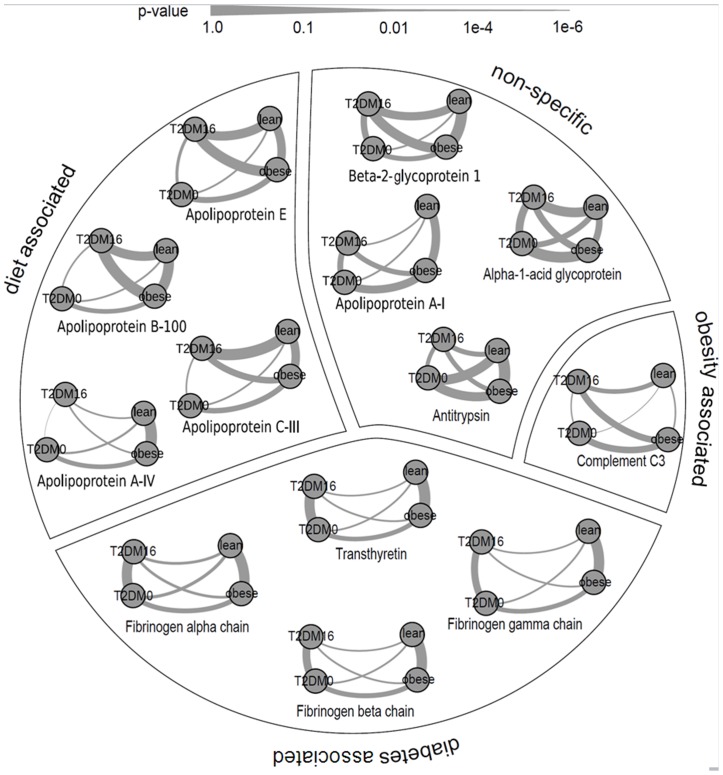
Graph representation of group wise comparisons for the proteins in the MRM data set. Comparisons between all pairs of the four groups, i.e., the diabetes patients at baseline (T2DM0) and after 16 weeks of VLCD (T2DM16) as well as the obese and lean control groups, are represented by edges, where the thickness of the edge represents the p-value. Groups that hardly can be discerned are thus connected by thick edges and located close together, whereas groups that can be well distinguished are connected by thin edges and are slightly further distinct. Furthermore, the proteins have been clustered into groups (i.e., obesity associated, diabetes associated, diet associated, and non- associated) based on similarity in patterns of differences between the groups.

Using a targeted MRM analysis we showed that several proteins differ between T2DM patients before and after a VLCD and between T2DM patients and lean and obese controls. As shown in Figure 2, these proteins can be divided in subgroups based on similar patterns of differences between the groups. Thereby a distinction can be made between potential biomarkers that are intervention (diet) or disease state (diabetes or obesity) associated.

### Diet associated markers

The proteins showing a diet effect most evidently in this study were the apolipoproteins, especially apolipoprotein A-IV (APOA-IV), as shown in Figure 2. APOA-IV is synthesized by the enterocytes of the small intestine in response to fat absorption [32],[33]. Although the precise role of APOA-IV has not been fully elucidated, studies suggest that it has anti-atherogenic [34] and anti-inflammatory [35] properties and that it serves as a satiety factor [32],[36].

Interestingly, APOA-IV levels were significantly higher in T2DM patients before the diet as compared to controls, which was also found in earlier studies [37],[38]. In contrast to our study, some also showed higher APOA-IV levels in obese, non-diabetic mice and humans [39],[40], though others did not find an association between APOA-IV and BMI [41]. An explanation for the higher APOA-IV levels, which is counter-intuitive, has not yet been identified. Shen et al. showed that obese mice, although peripheral APOA-IV levels were high, have lower APOA-IV levels in the hypothalamus, the site where APOA-IV is thought to exert its effect on satiety [39]. It has also been hypothesized that the high APOA-IV levels reflect a state of APOA-IV resistance [42], as is the case for leptin, which is also been thought to regulate APOA-IV [39].

APOA-IV also showed the highest MFC in response to the VLCD, resulting in significantly lower APOA-IV levels in T2DM patients after the diet than in controls. This decrease was consistently shown in 100% of the subjects, indicating that a decrease in APOA-IV might be a marker for weight loss. On the other hand it is known that APOA-IV levels are influenced by changes in dietary fat content [40],[43] and the observed decrease might thus be more reflective of the low amount of fat intake and caloric restriction during the VLCD. It would be interesting to investigate APOA-IV levels in patients back in a eucaloric state to elucidate this further. Furthermore, it has been hypothesized that, as APOA-IV serves as a satiety factor, lower APOA-IV levels can be a signal for stimulating feeding behavior [44]. Low APOA-IV levels may therefore contribute to the difficulties in maintaining achieved weight loss over longer periods of time. In this context it is interesting that in a study by Culnan et al., using iTRAQ proteomic analysis, an increase in APOA-IV levels was shown after weight loss induced by Roux-en-Y gastric bypass surgery (RYGB), which is known to result in more sustained weight loss as compared to diets [42]. The contrasting APOA-IV levels may, however, be explained by the fact that those after-surgery levels were measured after a mean follow-up of 19.2 months post-RYGB, and by the altered anatomy of the small intestine, the production site of APOA-IV.

### Complement C3 - an obesity associated marker

Accumulating evidence shows that both T2DM and obesity are associated with a chronic inflammatory state [3]. Complement C3 (C3) has an important role in the immune system and is produced by the liver, adipose tissue and macrophages [45]. Our MRM analysis showed higher concentrations of C3 in obese T2DM patients and healthy obese subjects as compared to lean controls. This agrees with several other studies, that also showed such elevated levels of C3 in patients with obesity [45],[46]. Furthermore, a significant decrease in C3 levels was seen after the VLCD, whereas no differences were shown between obese subjects with or without T2DM, indicating that C3 might be a marker of obesity rather than T2DM. However, other studies have demonstrated C3 levels to be increased in lean versus obese T2DM patients and to be associated with diabetes development independently of body weight [47],[48]. C3 has also proved to be higher in young adults with type 1 diabetes and a decrease in HbA1c in this group has been associated with a decrease in C3 levels [49]. These data indicate that C3 level and changes therein are dependent on the pathophysiology of the patient.

### Diabetes-associated markers

The fibrinogens were found to be elevated in T2DM patients as compared to both lean controls and obese controls, before as well as after the diet. Furthermore, concentrations did not differ between lean and obese controls, suggesting that fibrinogen is more diabetes than obesity associated. A high fibrinogen level is thought to reflect a hypercoagulable state and is suggested to be a strong independent cardiovascular risk factor [50],[51]. Other studies also found high fibrinogen levels in T2DM patients [52],[53] and this may contribute to the increased risk of cardiovascular events in type 2 diabetes [54],[55]. However, not all studies showed an increased fibrinogen level in T2DM patients [56]. After the VLCD we did not observe differences in the fibrinogen levels, although weight loss has been associated with a decrease in fibrinogen in literature [57].

Another interesting diabetes-associated marker found in this study is transthyrethin (TTR). TTR, previously known as pre-albumin, is a carrier protein for thyroid hormones and retinol-binding protein and is produced in the liver, choroid plexus and pancreatic islets [58]. TTR has been used as a biomarker for malnutrition [59]–[61] and has been shown to decrease in response to a VLCD [62]–[64]. However, it has been shown by Afolabi et al. that after an initial decrease at 5% weight loss, TTR levels returned back to baseline upon further weight loss [65]. In our study, where the average weight loss is 22%, also no differences were found after the VLCD.

### Exercise effect

No significant exercise effect was observed for any of the proteins in the MRM analysis. Therefore, we performed a large scale iTRAQ proteomic analysis to reveal candidate pathways involved in the additional beneficial effects of adding an exercise program that we have shown before [13]. Without correcting for multiple testing, concentrations were significantly different between the two VLCD groups for a few proteins, of which especially sex hormone-binding globulin (SHBG, P04278) and mannose-binding lectin (MBL)-associated serine protease (MASP-1, P48740) could be interesting. MASP-1 is a protease that contributes to the activation of the lectin complement pathway [66]. SHBG has been related to exercise before, although the influence of exercise on SHBG levels is less clear [67]–[70]. Moreover, SHBG levels are known to be inversely associated with insulin resistance and are thought to predict the risk on T2DM [71]. After correction for multiple testing, however, none of the proteins showed significant differences between the groups any more. Further research on these specific proteins is needed to uncover possible pathways involved in the beneficial effects of exercise.

### Strengths and limitations

The major strength of our study is the VLCD intervention. By studying T2DM patients before and after the diet, we showed that several proteins change with weight loss and improved glycemic control. By comparing the patients to obese and lean controls, these proteins could further be discerned between diabetes-associated and obesity-associated markers.

Limitations of our study are the lack of a control (non-diabetic obese) VLCD group as well as the absence of lean and obese control groups in the iTRAQ analysis. In addition, because of the many comparisons and the consequentially required correction for multiple testing, no significant differences were found using the iTRAQ analysis. The study would benefit from quantification of one or more promising candidates by an independent complementary technology (e.g. ELISA). This was, however, beyond the scope of the current study.

In conclusion, using proteomic analysis several potential disease state and intervention associated markers were found distinguishing T2DM patients from obese and lean controls and showing a VLCD effect. Although no specific exercise markers were discovered, the iTRAQ analysis indicated some proteins as potential interesting targets for further research.

## Supporting Information

File S1 **Supporting Tables.** Supplementary Table S1: exercise effect for proteins in the MRM dataset; Supplementary Table S2: peptide sequences for protein MRM measurements; Supplementary Table S3: exercise effect for proteins identified from iTRAQ experiments; Supplementary Table S4: VLCD effect for proteins identified from iTRAQ experiments; Supplementary Table S5: peptide mapping iTRAQ experiments.
(DOC)
