# Supplementary material for: Proteomic Analysis in Type 2 Diabetes Patients before and after a Very Low Calorie Diet Reveals Potential Disease State and Intervention Specific Biomarkers
Source: PLoS One. 2014 Nov 21;9(11):e112835. doi: 10.1371/journal.pone.0112835 (PMC4240577; doi:10.1371/journal.pone.0112835)
Supplement: File S1 — Supporting Tables. Supplementary Table S1: exercise effect for proteins in the MRM dataset; Supplementary Table S2: peptide sequences for protein MRM measurements; Supplementary Table S3: exercise effect for proteins identified from iTRAQ experiments; Supplementary Table S4: VLCD effect for proteins identified from iTRAQ experiments; Supplementary Table S5: peptide mapping iTRAQ experiments. (DOC) [file pone.0112835.s001.doc]

| **Supplementary Table S1.** Exercise effect for proteins in the MRM dataset. | | | | | | | | | | | | | |  |  |  |  |
| --- | --- | --- | --- | --- | --- | --- | --- | --- | --- | --- | --- | --- | --- | --- | --- | --- | --- |
|  |  |  |  |  |  |  |  |  |  |  |  |  |  |  |  |  |  |
|  | **VLCD+exercise** | | | | | | |  | **VLCD only** | | | | | | |  |  |
|  | baseline | | |  | 16 weeks | | |  | baseline | | |  | 16 weeks | | |  | adj. p-value |
| Alpha-1-acid glycoprotein | 0.99 | ± | 0.06 |  | 0.88 | ± | 0.09 |  | 1.05 | ± | 0.14 |  | 0.93 | ± | 0.07 |  | 0.96 |
| Antitrypsin | 1.02 | ± | 0.05 |  | 1.13 | ± | 0.05 |  | 1.01 | ± | 0.03 |  | 1.09 | ± | 0.07 |  | 0.80 |
| Apolipoprotein A-I | 0.93 | ± | 0.03 |  | 0.84 | ± | 0.04 |  | 0.96 | ± | 0.05 |  | 0.93 | ± | 0.07 |  | 0.60 |
| Apolipoprotein A-IV | 1.33 | ± | 0.10 |  | 0.68 | ± | 0.08 |  | 1.34 | ± | 0.13 |  | 0.74 | ± | 0.09 |  | 0.80 |
| Apolipoprotein B-100 | 1.15 | ± | 0.11 |  | 0.92 | ± | 0.07 |  | 1.25 | ± | 0.11 |  | 1.07 | ± | 0.06 |  | 0.78 |
| Apolipoprotein C-III | 1.33 | ± | 0.25 |  | 0.72 | ± | 0.06 |  | 1.40 | ± | 0.16 |  | 0.97 | ± | 0.07 |  | 0.78 |
| Apolipoprotein E | 1.29 | ± | 0.19 |  | 0.86 | ± | 0.06 |  | 1.20 | ± | 0.10 |  | 1.06 | ± | 0.07 |  | 0.43 |
| Beta-2-glycoprotein 1 | 1.14 | ± | 0.05 |  | 0.99 | ± | 0.05 |  | 1.08 | ± | 0.04 |  | 1.05 | ± | 0.06 |  | 0.43 |
| Complement C3 | 1.22 | ± | 0.05 |  | 0.94 | ± | 0.06 |  | 1.14 | ± | 0.04 |  | 1.00 | ± | 0.05 |  | 0.43 |
| Fibrinogen alpha chain | 1.09 | ± | 0.08 |  | 1.09 | ± | 0.09 |  | 0.99 | ± | 0.06 |  | 1.07 | ± | 0.07 |  | 0.72 |
| Fibrinogen beta chain | 1.11 | ± | 0.07 |  | 1.11 | ± | 0.08 |  | 1.01 | ± | 0.05 |  | 1.12 | ± | 0.05 |  | 0.43 |
| Fibrinogen gamma chain | 1.11 | ± | 0.07 |  | 1.13 | ± | 0.08 |  | 1.00 | ± | 0.05 |  | 1.13 | ± | 0.06 |  | 0.43 |
| Transthyretin | 0.92 | ± | 0.07 |  | 0.87 | ± | 0.07 |  | 0.83 | ± | 0.04 |  | 0.82 | ± | 0.04 |  | 0.78 |
| Mean ± SEM. |  |  |  |  |  |  |  |  |  |  |  |  |  |  |  |  |  |

**Supplementary Table S2.** Parameters for the protein MRM measurements.

| **Protein** | | **Gene Symbol** | **Peptide Sequence** | **Transition** | | **Q1 (m/z)** | | **Q3 (m/z)** | | |
| --- | --- | --- | --- | --- | --- | --- | --- | --- | --- | --- |
| Apolipoprotein A-I | | APOA1 | THLAPYSDELR | MH_3_^3+^ -> b_4_^+^ | | 434.6 | | 423.3 | | |
|  |  |  |  | MH_3_^3+^ -> y_5_^+^ | | 434.6 | | 619.3 | | |
|  |  |  | ATEHLSTLSEK | MH_3_^3+^ -> y_3_^+^ | | 405.9 | | 363.3 | | |
|  |  |  |  | MH_3_^3+^ -> y_9_^2+^ | | 405.9 | | 522.3 | | |
| Apolipoprotein A-IV | | APOA4 | IDQNVEELK | MH_2_^2+^ -> y_4_^+^ | | 544.3 | | 518.3 | | |
|  |  |  |  | MH_2_^2+^ -> y_6_^+^ | | 544.3 | | 731.4 | | |
|  |  |  | SLAPYAQDTQEK | MH_2_^2+^ -> y_9_^2+^ | | 675.8 | | 575.8 | | |
|  |  |  |  | MH_2_^2+^ -> y_10_^2+^ | | 675.8 | | 540.3 | | |
| Apolipoprotein B-100 | | APOB | TEVIPPLIENR | MH_2_^2+^ -> y_7_^+^ | | 640.8 | | 838.4 | | |
|  |  |  |  | MH_2_^2+^ -> y_7_^2+^ | | 640.8 | | 419.8 | | |
|  |  |  | FPEVDVLTK | MH_2_^2+^ -> y_7_^+^ | | 524.3 | | 803.5 | | |
|  |  |  |  | MH_2_^2+^ -> y_4_^+^ | | 524.3 | | 450.8 | | |
| Apolipoprotein C-III | | APOC3 | ADALSSVQESQVAQQAR | MH_3_^3+^ -> b_4_^+^ | | 572.9 | | 672.4 | | |
|  |  |  |  | MH_3_^3+^ -> y_5_^+^ | | 572.9 | | 800.4 | | |
|  |  |  | GWVTDGFSSLK | MH_2_^2+^ -> y_6_^+^ | | 598.8 | | 638.4 | | |
|  |  |  |  | MH_2_^2+^ -> y_8_^+^ | | 598.8 | | 854.4 | | |
| Apolipoprotein E | | APOE | LAVYQAGAR | MH_2_^2+^ -> y_6_^+^ | | 474.8 | | 665.3 | | |
|  |  |  |  | MH_2_^2+^ -> y_7_^+^ | | 474.8 | | 764.4 | | |
|  |  |  | LGPLVEQGR | MH_2_^2+^ -> y_5_^+^ | | 484.8 | | 588.3 | | |
|  |  |  |  | MH_2_^2+^ -> y_7_^2+^ | | 484.8 | | 399.7 | | |
| Beta-2-glycoprotein 1 | | APOH | ATVVYQGER | MH_2_^2+^ -> y_6_^+^ | | 511.8 | | 652.3 | | |
|  |  |  |  | MH_2_^2+^ -> y_7_^+^ | | 511.8 | | 751.4 | | |
|  |  |  | VCPFAGILENGAVR | MH_3_^3+^ -> y_5_^+^ | | 501.6 | | 516.3 | | |
|  |  |  |  | MH_3_^3+^ -> y_6_^+^ | | 501.6 | | 645.3 | | |
| Complement C3 | | C3 | SSLSVPYVIVPLK | MH_2_^2+^ -> y_3_^+^ | | 467.9 | | 357.3 | | |
|  |  |  |  | MH_2_^2+^ -> y_5_^+^ | | 467.9 | | 569.4 | | |
|  |  |  | TGLQEVEVK | MH_2_^2+^ -> y_6_^+^ | | 501.8 | | 603.3 | | |
|  |  |  |  | MH_2_^2+^ -> y_7_^+^ | | 501.8 | | 731.4 | | |
| Fibrinogen Alpha Chain | | FGA | NSLFEYQK | MH_2_^2+^ -> b_3_^+^ | | 514.8 | | 315.2 | | |
|  |  |  |  | MH_2_^2+^ -> y_5_^+^ | | 514.8 | | 714.4 | | |
|  |  |  | HPDEAAFFDTASTGK | MH_3_^3+^ -> y_7_^+^ | | 531.9 | | 621.3 | | |
|  |  |  |  | MH_3_^3+^ -> y_3_^+^ | | 531.9 | | 679.3 | | |
| Fibrinogen Beta Chain | | FGB | AHYGGFTVQNEANK | MH_2_^2+^ -> y_5_^+^ | | 512.6 | | 575.3 | | |
|  |  |  |  | MH_2_^2+^ -> y_6_^+^ | | 512.6 | | 703.3 | | |
|  |  |  | HGTDDGVVWMNWK | MH_3_^3+^ -> b_7_^+^ | | 515.8 | | 682.3 | | |
|  |  |  |  | MH_3_^3+^ -> y_6_^+^ | | 515.8 | | 432.2 | | |
| Fibrinogen Gamma Chain | | FGG | YEASILTHDSSIR | MH_3_^3+^ -> b_5_^+^ | | 497.9 | | 564.8 | | |
|  |  |  |  | MH_3_^3+^ -> y_11_^2+^ | | 497.9 | | 600.3 | | |
|  |  |  | IHLISTQSAIPYALR | MH_3_^3+^ -> b_3_^+^ | | 561.6 | | 364.2 | | |
|  |  |  |  | MH_3_^3+^ -> y_5_^+^ | | 561.6 | | 619.4 | | |
| Alpha-1-acid glycoprotein | | ORM1 | YVGGQEHFAHLLILR | MH_3_^3+^ -> y_13_^2+^ | | 584.9 | | 745.9 | | |
|  |  |  |  | MH_3_^3+^ -> y_14_^2+^ | | 584.9 | | 795.5 | | |
|  |  |  | NWGLSVYADKPETTK | MH_3_^3+^ -> y_11_^2+^ | | 570.3 | | 619.8 | | |
|  |  |  |  | MH_3_^3+^ -> y_13_^2+^ | | 570.3 | | 704.9 | | |
| Alpha-1-Antitrypsin | | SERPINA1 | LSITGTYDLK | MH_2_^2+^ -> y_6_^+^ | | 555.8 | | 696.4 | | |
|  |  |  |  | MH_2_^2+^ -> y_7_^+^ | | 555.8 | | 797.5 | | |
|  |  |  | AVLTIDEK | MH_2_^2+^ -> y_5_^+^ | | 444.7 | | 605.3 | | |
|  |  |  |  | MH_2_^2+^ -> y_6_^+^ | | 444.7 | | 718.4 | | |
| Transthyretin | | TTR | GSPAINVAVHVFR | MH_3_^3+^ -> y_11_^2+^ | | 456.3 | | 611.9 | | |
|  |  |  |  | MH_3_^3+^ -> y_13_^2+^ | | 456.3 | | 408.3 | | |
|  |  |  | AADDTWEPFASGK | MH_2_^2+^ -> y_6_^+^ | | 697.8 | | 606.3 | | |
|  |  |  |  | MH_2_^2+^ -> y_8_^+^ | | 697.8 | | 921.4 | | |
|  | |  |  |  | |  | |  | | |
|  | **Supplementary Table S3.** Exercise effect for proteins identified from iTRAQ experiments. | | | | | | | |  |  |
|  |  | | | |  | |  | |  |  |
|  |  | | | | **p-value** | | **adj. p-value** | | **MD** |  |
|  | Complement-activating component of Ra-reactive factor precursor | | | | 0.002 | | 0.337 | | -0.14 |  |
|  | Isoform 1 of Sex hormone-binding globulin | | | | 0.003 | | 0.337 | | 0.34 |  |
|  | Cartilage oligomeric matrix protein | | | | 0.013 | | 0.553 | | -0.11 |  |
|  | Isoform 2 of Inter-alpha-trypsin inhibitor heavy chain H4 | | | | 0.016 | | 0.553 | | 0.10 |  |
|  | Cathepsin D | | | | 0.017 | | 0.553 | | -0.14 |  |
|  | Isoform 1 of CD166 antigen | | | | 0.017 | | 0.553 | | -0.13 |  |
|  | Carboxypeptidase N subunit 2 precursor | | | | 0.018 | | 0.553 | | -0.09 |  |
|  | Apolipoprotein B-100 | | | | 0.027 | | 0.553 | | -0.11 |  |
|  | Isoform 1 of Pregnancy zone protein | | | | 0.029 | | 0.553 | | 0.22 |  |
|  | Alpha-amylase 2B | | | | 0.029 | | 0.553 | | -0.29 |  |
|  | Ig kappa chain V-IV region | | | | 0.030 | | 0.553 | | -0.37 |  |
|  | Isoform 2 of Vascular non-inflammatory molecule 3 | | | | 0.030 | | 0.553 | | -0.16 |  |
|  | Immunoglobulin superfamily containing leucine-rich repeat protein precursor | | | | 0.031 | | 0.553 | | -0.13 |  |
|  | Complement C5 precursor | | | | 0.036 | | 0.557 | | -0.18 |  |
|  | Cadherin-13 precursor | | | | 0.040 | | 0.557 | | -0.25 |  |
|  | Ig lambda chain V-I region NIG-64 | | | | 0.041 | | 0.557 | | -0.34 |  |
|  | Ig kappa chain V-I region CAR | | | | 0.041 | | 0.557 | | -0.25 |  |
|  | Ig lambda chain V-IV region Hil | | | | 0.043 | | 0.557 | | -0.33 |  |
|  | Muscle type neuropilin 1 | | | | 0.050 | | 0.576 | | 0.09 |  |
|  | Isoform 1 of Coagulation factor XI | | | | 0.056 | | 0.576 | | -0.07 |  |
|  | ADP-ribosyl cyclase 2 precursor | | | | 0.057 | | 0.576 | | -0.07 |  |
|  | Complement C5 precursor | | | | 0.057 | | 0.576 | | -0.12 |  |
|  | Cholinesterase precursor | | | | 0.060 | | 0.576 | | -0.08 |  |
|  | Isoform 1 of Ectonucleotide pyrophosphatase/phosphodiesterase family member 2 | | | | 0.061 | | 0.576 | | -0.13 |  |
|  | apolipoprotein A-IV precursor | | | | 0.063 | | 0.576 | | 0.72 |  |
|  | cDNA FLJ55673, highly similar to Complement factor B | | | | 0.065 | | 0.576 | | -0.05 |  |
|  | Monocyte differentiation antigen CD14 precursor | | | | 0.069 | | 0.576 | | 0.07 |  |
|  | 72 kDa type IV collagenase | | | | 0.071 | | 0.576 | | 0.08 |  |
|  | Coagulation factor X precursor | | | | 0.073 | | 0.576 | | -0.07 |  |
|  | Alpha-2-macroglobulin precursor | | | | 0.076 | | 0.576 | | 0.19 |  |
|  | Dopamine beta-hydroxylase | | | | 0.077 | | 0.576 | | -0.11 |  |
|  | Isoform 1 of Vitamin K-dependent protein Z precursor | | | | 0.081 | | 0.579 | | -0.10 |  |
|  | Alpha-2-macroglobulin precursor | | | | 0.082 | | 0.579 | | 0.16 |  |
|  | Isoform 1 of Contactin-1 precursor | | | | 0.089 | | 0.585 | | -0.08 |  |
|  | Carboxypeptidase N catalytic chain precursor | | | | 0.094 | | 0.585 | | -0.18 |  |
|  | Inter-alpha-trypsin inhibitor heavy chain H2 | | | | 0.094 | | 0.585 | | -0.08 |  |
|  | Ig kappa chain V-I region AU | | | | 0.097 | | 0.585 | | -0.17 |  |
|  | Complement C4-A | | | | 0.098 | | 0.585 | | -0.09 |  |
|  | Isoform 1 of Phosphatidylinositol-glycan-specific phospholipase D precursor | | | | 0.098 | | 0.585 | | -0.10 |  |
|  | AMBP protein precursor | | | | 0.101 | | 0.585 | | -0.10 |  |
|  | Reticulon-4 receptor-like 2 precursor | | | | 0.108 | | 0.601 | | -0.19 |  |
|  | Apolipoprotein B-100 precursor | | | | 0.109 | | 0.601 | | -0.08 |  |
|  | Isoform 2 of Collagen alpha-1(XVIII) chain precursor | | | | 0.113 | | 0.605 | | 0.08 |  |
|  | Membrane copper amine oxidase | | | | 0.115 | | 0.605 | | -0.11 |  |
|  | tropomyosin 1 alpha chain isoform 2 | | | | 0.124 | | 0.637 | | 0.31 |  |
|  | Ig mu heavy chain disease protein | | | | 0.126 | | 0.637 | | -0.33 |  |
|  | Procollagen C-endopeptidase enhancer 1 | | | | 0.137 | | 0.678 | | -0.07 |  |
|  | Complement component 6 precursor | | | | 0.144 | | 0.683 | | -0.06 |  |
|  | Retinoic acid receptor responder protein 2 precursor | | | | 0.144 | | 0.683 | | -0.06 |  |
|  | Cholinesterase precursor | | | | 0.176 | | 0.789 | | -0.07 |  |
|  | Coagulation factor XII precursor | | | | 0.181 | | 0.789 | | -0.10 |  |
|  | Vitamin D-binding protein precursor | | | | 0.181 | | 0.789 | | -0.05 |  |
|  | immunoglobulin J chain | | | | 0.183 | | 0.789 | | -0.19 |  |
|  | similar to complement component 3 | | | | 0.186 | | 0.789 | | -0.18 |  |
|  | Glutathione peroxidase 3 precursor | | | | 0.191 | | 0.789 | | -0.09 |  |
|  | SPARC-like protein 1 | | | | 0.199 | | 0.789 | | -0.09 |  |
|  | Hepatocyte growth factor activator precursor | | | | 0.203 | | 0.789 | | -0.04 |  |
|  | Leucine-rich alpha-2-glycoprotein precursor | | | | 0.205 | | 0.789 | | -0.11 |  |
|  | Transferrin receptor protein 1 | | | | 0.205 | | 0.789 | | -0.07 |  |
|  | Isoform 1 of Collagen alpha-3(VI) chain | | | | 0.206 | | 0.789 | | -0.05 |  |
|  | Complement C4-A | | | | 0.215 | | 0.789 | | -0.08 |  |
|  | Xaa-Pro dipeptidase | | | | 0.216 | | 0.789 | | -0.13 |  |
|  | Fructose-bisphosphate aldolase B | | | | 0.222 | | 0.789 | | -0.12 |  |
|  | Vitamin K-dependent protein S | | | | 0.230 | | 0.789 | | 0.05 |  |
|  | Alpha-1-antitrypsin | | | | 0.234 | | 0.789 | | 0.09 |  |
|  | Isoform 1 of Sulfhydryl oxidase 1 precursor | | | | 0.236 | | 0.789 | | -0.05 |  |
|  | Uncharacterized protein FETUB | | | | 0.237 | | 0.789 | | -0.10 |  |
|  | Complement component C7 | | | | 0.251 | | 0.789 | | -0.03 |  |
|  | Intercellular adhesion molecule 1 | | | | 0.256 | | 0.789 | | -0.05 |  |
|  | Isoform 2 of Multiple inositol polyphosphate phosphatase 1 | | | | 0.258 | | 0.789 | | 0.04 |  |
|  | Isoform Gamma-B of Fibrinogen gamma chain | | | | 0.260 | | 0.789 | | -0.17 |  |
|  | Clusterin precursor | | | | 0.262 | | 0.789 | | 0.08 |  |
|  | Transforming growth factor-beta-induced protein ig-h3 precursor | | | | 0.263 | | 0.789 | | -0.06 |  |
|  | Vitamin K-dependent protein C | | | | 0.265 | | 0.789 | | 0.06 |  |
|  | Apolipoprotein A-II precursor | | | | 0.274 | | 0.789 | | -0.04 |  |
|  | Insulin-like growth factor-binding protein 2 | | | | 0.274 | | 0.789 | | 0.10 |  |
|  | Serum paraoxonase/arylesterase 1 | | | | 0.281 | | 0.789 | | -0.07 |  |
|  | Transforming growth factor-beta-induced protein ig-h3 | | | | 0.288 | | 0.789 | | -0.05 |  |
|  | Beta-Ala-His dipeptidase | | | | 0.288 | | 0.789 | | -0.07 |  |
|  | Procollagen C-endopeptidase enhancer 1 precursor | | | | 0.290 | | 0.789 | | -0.05 |  |
|  | Histidine-rich glycoprotein precursor | | | | 0.291 | | 0.789 | | 0.04 |  |
|  | Plasma glutamate carboxypeptidase | | | | 0.292 | | 0.789 | | -0.09 |  |
|  | Isoform 3 of Neural cell adhesion molecule 1 | | | | 0.296 | | 0.789 | | -0.05 |  |
|  | Follistatin-related protein 1 | | | | 0.296 | | 0.789 | | 0.07 |  |
|  | Isoform 1 of Low affinity immunoglobulin gamma Fc region receptor II-a | | | | 0.302 | | 0.789 | | 0.05 |  |
|  | Isoform 1 of Vascular cell adhesion protein 1 precursor | | | | 0.304 | | 0.789 | | 0.05 |  |
|  | Isoform 1 of Extracellular matrix protein 1 | | | | 0.305 | | 0.789 | | 0.07 |  |
|  | Vitronectin precursor | | | | 0.306 | | 0.789 | | 0.07 |  |
|  | Dopamine beta-hydroxylase | | | | 0.307 | | 0.789 | | -0.08 |  |
|  | Fibrinogen beta chain precursor | | | | 0.311 | | 0.789 | | -0.21 |  |
|  | Corticosteroid-binding globulin precursor | | | | 0.314 | | 0.789 | | -0.04 |  |
|  | Hemopexin precursor | | | | 0.316 | | 0.789 | | -0.07 |  |
|  | Thrombospondin-4 precursor | | | | 0.320 | | 0.789 | | -0.08 |  |
|  | Uncharacterized protein KLKB1 | | | | 0.325 | | 0.789 | | -0.04 |  |
|  | Angiotensinogen | | | | 0.326 | | 0.789 | | 0.05 |  |
|  | Fc-gamma receptor IIIb | | | | 0.326 | | 0.789 | | -0.04 |  |
|  | CD5 antigen-like precursor | | | | 0.335 | | 0.800 | | -0.06 |  |
|  | Ig lambda chain V-I region NIG-64 | | | | 0.345 | | 0.808 | | -0.09 |  |
|  | Pantetheinase precursor | | | | 0.347 | | 0.808 | | -0.05 |  |
|  | Plastin-2 | | | | 0.349 | | 0.808 | | 0.06 |  |
|  | Isoform 1 of Fibronectin | | | | 0.353 | | 0.808 | | 0.13 |  |
|  | Isoform 2 of Neural cell adhesion molecule L1-like protein | | | | 0.358 | | 0.808 | | 0.04 |  |
|  | Isoform 1 of Mannan-binding lectin serine protease 2 precursor | | | | 0.365 | | 0.808 | | -0.10 |  |
|  | Coagulation factor IX | | | | 0.370 | | 0.808 | | 0.05 |  |
|  | alpha-2-glycoprotein 1, zinc | | | | 0.370 | | 0.808 | | 0.05 |  |
|  | Complement factor I | | | | 0.371 | | 0.808 | | -0.04 |  |
|  | Ceruloplasmin | | | | 0.372 | | 0.808 | | -0.06 |  |
|  | Isoform XB of Tenascin-X | | | | 0.382 | | 0.820 | | -0.05 |  |
|  | GUGU beta form | | | | 0.398 | | 0.847 | | -0.05 |  |
|  | Afamin precursor | | | | 0.410 | | 0.858 | | -0.05 |  |
|  | Phosphatidylcholine-sterol acyltransferase precursor | | | | 0.412 | | 0.858 | | -0.02 |  |
|  | Hepatocyte growth factor-like protein | | | | 0.417 | | 0.858 | | -0.02 |  |
|  | Fibrinogen beta chain precursor | | | | 0.418 | | 0.858 | | -0.15 |  |
|  | Coagulation factor X | | | | 0.423 | | 0.860 | | -0.06 |  |
|  | Serum amyloid P-component precursor | | | | 0.429 | | 0.865 | | 0.08 |  |
|  | von Willebrand factor | | | | 0.437 | | 0.868 | | 0.03 |  |
|  | Endothelial protein C receptor precursor | | | | 0.438 | | 0.868 | | 0.03 |  |
|  | Isoform HMW of Kininogen-1 | | | | 0.448 | | 0.881 | | -0.03 |  |
|  | Corticosteroid-binding globulin precursor | | | | 0.463 | | 0.886 | | 0.03 |  |
|  | Serotransferrin precursor | | | | 0.463 | | 0.886 | | -0.07 |  |
|  | Gamma-glutamyl hydrolase precursor | | | | 0.468 | | 0.886 | | -0.02 |  |
|  | Coagulation factor IX precursor | | | | 0.472 | | 0.886 | | 0.04 |  |
|  | alpha-2-glycoprotein 1, zinc | | | | 0.473 | | 0.886 | | 0.02 |  |
|  | Complement C5 precursor | | | | 0.474 | | 0.886 | | -0.07 |  |
|  | Isoform LAMP-2A of Lysosome-associated membrane glycoprotein 2 | | | | 0.482 | | 0.890 | | -0.03 |  |
|  | Insulin-like growth factor-binding protein complex acid labile chain | | | | 0.483 | | 0.890 | | -0.03 |  |
|  | cDNA FLJ55606, highly similar to Alpha-2-HS-glycoprotein | | | | 0.493 | | 0.901 | | -0.07 |  |
|  | Plasma serine protease inhibitor precursor | | | | 0.510 | | 0.918 | | -0.05 |  |
|  | Serotransferrin | | | | 0.511 | | 0.918 | | -0.07 |  |
|  | Prothrombin (Fragment) | | | | 0.517 | | 0.919 | | -0.05 |  |
|  | Prothrombin precursor (Fragment) | | | | 0.521 | | 0.919 | | -0.04 |  |
|  | Lumican precursor | | | | 0.523 | | 0.919 | | -0.03 |  |
|  | Pigment epithelium-derived factor precursor | | | | 0.534 | | 0.932 | | -0.06 |  |
|  | Peroxiredoxin-1 | | | | 0.541 | | 0.936 | | 0.08 |  |
|  | Apolipoprotein C-I precursor | | | | 0.546 | | 0.936 | | 0.05 |  |
|  | Tetranectin precursor | | | | 0.556 | | 0.936 | | -0.03 |  |
|  | Complement component C7 | | | | 0.558 | | 0.936 | | -0.05 |  |
|  | Plasma protease C1 inhibitor | | | | 0.559 | | 0.936 | | 0.03 |  |
|  | Selenoprotein P | | | | 0.562 | | 0.936 | | 0.02 |  |
|  | Complement component C9 precursor | | | | 0.566 | | 0.936 | | 0.05 |  |
|  | Reticulon-4 receptor-like 2 precursor | | | | 0.573 | | 0.936 | | -0.02 |  |
|  | Complement component C8 alpha chain precursor | | | | 0.574 | | 0.936 | | -0.02 |  |
|  | Pigment epithelium-derived factor precursor | | | | 0.577 | | 0.936 | | -0.04 |  |
|  | Isoform 1 of Gelsolin precursor | | | | 0.583 | | 0.938 | | 0.04 |  |
|  | Basement membrane-specific heparan sulfate proteoglycan core protein | | | | 0.586 | | 0.938 | | -0.03 |  |
|  | Coagulation factor XIII A chain | | | | 0.592 | | 0.941 | | 0.04 |  |
|  | Isoform 1 of Fibrinogen alpha chain | | | | 0.602 | | 0.942 | | -0.08 |  |
|  | Ribonuclease pancreatic precursor | | | | 0.606 | | 0.942 | | -0.03 |  |
|  | Coagulation factor V | | | | 0.608 | | 0.942 | | 0.04 |  |
|  | Hemopexin | | | | 0.617 | | 0.942 | | -0.04 |  |
|  | Isoform 1 of C-reactive protein | | | | 0.618 | | 0.942 | | 0.13 |  |
|  | Isoform 1 of Isocitrate dehydrogenase [NAD] subunit alpha, mitochondrial | | | | 0.623 | | 0.942 | | 0.04 |  |
|  | Isoform 2 of Carboxypeptidase B2 | | | | 0.626 | | 0.942 | | 0.02 |  |
|  | cDNA FLJ55673, highly similar to Complement factor B | | | | 0.628 | | 0.942 | | -0.01 |  |
|  | Apolipoprotein A-IV precursor | | | | 0.629 | | 0.942 | | -0.07 |  |
|  | Protein AMBP | | | | 0.636 | | 0.943 | | -0.04 |  |
|  | Isoform 3 of Mannan-binding lectin serine protease 1 | | | | 0.642 | | 0.943 | | -0.01 |  |
|  | Cadherin-5 | | | | 0.644 | | 0.943 | | 0.02 |  |
|  | Plastin-2 | | | | 0.648 | | 0.943 | | 0.02 |  |
|  | Complement C1s subcomponent | | | | 0.650 | | 0.943 | | -0.02 |  |
|  | Mannose-binding protein C precursor | | | | 0.661 | | 0.944 | | -0.03 |  |
|  | Isoform 1 of Peptidase inhibitor 16 precursor | | | | 0.662 | | 0.944 | | -0.02 |  |
|  | Alpha-1-acid glycoprotein 2 | | | | 0.666 | | 0.944 | | -0.07 |  |
|  | Isoform 1 of N-acetylmuramoyl-L-alanine amidase precursor | | | | 0.670 | | 0.944 | | 0.07 |  |
|  | Biotinidase | | | | 0.677 | | 0.944 | | -0.01 |  |
|  | 4F2 cell-surface antigen heavy chain | | | | 0.686 | | 0.944 | | -0.02 |  |
|  | Leucine-rich alpha-2-glycoprotein precursor | | | | 0.693 | | 0.944 | | 0.03 |  |
|  | Isoform 1 of Multiple inositol polyphosphate phosphatase 1 precursor | | | | 0.696 | | 0.944 | | -0.02 |  |
|  | Isoform 3 of Interleukin-1 receptor accessory protein | | | | 0.703 | | 0.944 | | 0.01 |  |
|  | Isoform 1 of Carboxypeptidase B2 precursor | | | | 0.707 | | 0.944 | | -0.02 |  |
|  | Properdin precursor | | | | 0.709 | | 0.944 | | -0.02 |  |
|  | Cystatin-C precursor | | | | 0.712 | | 0.944 | | 0.03 |  |
|  | Ceruloplasmin precursor | | | | 0.713 | | 0.944 | | -0.03 |  |
|  | Serpin peptidase inhibitor, clade D (Heparin cofactor), member 1 | | | | 0.716 | | 0.944 | | -0.05 |  |
|  | Putative uncharacterized protein ALB | | | | 0.719 | | 0.944 | | -0.21 |  |
|  | MAN1A1 protein | | | | 0.720 | | 0.944 | | -0.03 |  |
|  | Thyroxine-binding globulin precursor | | | | 0.720 | | 0.944 | | 0.02 |  |
|  | HP protein | | | | 0.727 | | 0.944 | | -0.02 |  |
|  | Complement C1s subcomponent | | | | 0.729 | | 0.944 | | 0.03 |  |
|  | Coagulation factor XIII B chain precursor | | | | 0.733 | | 0.945 | | 0.02 |  |
|  | Plasma serine protease inhibitor | | | | 0.740 | | 0.948 | | -0.02 |  |
|  | Insulin-like growth factor IA | | | | 0.751 | | 0.949 | | -0.04 |  |
|  | Galectin-3-binding protein | | | | 0.752 | | 0.949 | | -0.02 |  |
|  | Lysozyme C precursor | | | | 0.754 | | 0.949 | | -0.01 |  |
|  | Mannosyl-oligosaccharide 1,2-alpha-mannosidase IA | | | | 0.758 | | 0.949 | | -0.03 |  |
|  | Isoform 1 of Inter-alpha-trypsin inhibitor heavy chain H3 | | | | 0.761 | | 0.949 | | 0.01 |  |
|  | Kallistatin precursor | | | | 0.767 | | 0.951 | | -0.02 |  |
|  | Protein Z-dependent protease inhibitor precursor | | | | 0.772 | | 0.951 | | -0.01 |  |
|  | Apolipoprotein A-I precursor | | | | 0.775 | | 0.951 | | -0.03 |  |
|  | Isoform 1 of Cartilage acidic protein 1 precursor | | | | 0.798 | | 0.959 | | -0.01 |  |
|  | Serum paraoxonase/arylesterase 1 | | | | 0.800 | | 0.959 | | 0.02 |  |
|  | Isoform 1 of Attractin | | | | 0.801 | | 0.959 | | -0.01 |  |
|  | Aminopeptidase N | | | | 0.805 | | 0.959 | | -0.01 |  |
|  | Insulin-like growth factor-binding protein 3 | | | | 0.807 | | 0.959 | | -0.01 |  |
|  | Inter-alpha-trypsin inhibitor heavy chain H1 precursor | | | | 0.813 | | 0.959 | | 0.01 |  |
|  | 30 kDa protein | | | | 0.816 | | 0.959 | | 0.01 |  |
|  | Alpha-1-acid glycoprotein 2 precursor | | | | 0.816 | | 0.959 | | -0.03 |  |
|  | HSPA5 protein | | | | 0.823 | | 0.959 | | -0.01 |  |
|  | Complement factor D preproprotein | | | | 0.828 | | 0.959 | | -0.01 |  |
|  | Isoform A of Coagulation factor VII | | | | 0.829 | | 0.959 | | 0.01 |  |
|  | Carboxypeptidase N catalytic chain precursor | | | | 0.831 | | 0.959 | | -0.02 |  |
|  | Complement component C1q receptor | | | | 0.848 | | 0.971 | | -0.01 |  |
|  | Hemoglobin subunit epsilon | | | | 0.850 | | 0.971 | | -0.03 |  |
|  | Apolipoprotein C-III precursor | | | | 0.854 | | 0.971 | | -0.02 |  |
|  | 45 kDa protein | | | | 0.865 | | 0.976 | | 0.01 |  |
|  | Apolipoprotein E | | | | 0.872 | | 0.976 | | -0.01 |  |
|  | Complement C1r subcomponent precursor | | | | 0.878 | | 0.976 | | -0.01 |  |
|  | Vasorin precursor | | | | 0.881 | | 0.976 | | 0.00 |  |
|  | Complement component C8 beta chain precursor | | | | 0.882 | | 0.976 | | -0.02 |  |
|  | Angiogenin precursor | | | | 0.887 | | 0.976 | | -0.01 |  |
|  | Apolipoprotein A-I precursor | | | | 0.889 | | 0.976 | | -0.02 |  |
|  | Antithrombin III variant | | | | 0.894 | | 0.976 | | -0.01 |  |
|  | Carbonic anhydrase 1 | | | | 0.903 | | 0.976 | | 0.02 |  |
|  | Cadherin-2 | | | | 0.904 | | 0.976 | | 0.01 |  |
|  | Isoform 1 of Vinculin | | | | 0.906 | | 0.976 | | -0.01 |  |
|  | Complement C1r subcomponent-like protein | | | | 0.909 | | 0.976 | | 0.01 |  |
|  | Insulin-like growth factor-binding protein 5 | | | | 0.920 | | 0.976 | | -0.01 |  |
|  | Isoform 1 of Phosphatidylinositol-glycan-specific phospholipase D precursor | | | | 0.920 | | 0.976 | | 0.01 |  |
|  | Protein AMBP | | | | 0.921 | | 0.976 | | 0.01 |  |
|  | Flavin reductase | | | | 0.937 | | 0.978 | | 0.01 |  |
|  | Insulin-like growth factor-binding protein 6 precursor | | | | 0.937 | | 0.978 | | 0.00 |  |
|  | Apolipoprotein A-IV precursor | | | | 0.937 | | 0.978 | | -0.01 |  |
|  | Isoform 2 of Inter-alpha-trypsin inhibitor heavy chain H4 | | | | 0.940 | | 0.978 | | 0.01 |  |
|  | Lumican precursor | | | | 0.951 | | 0.985 | | 0.00 |  |
|  | Isoform 1 of Insulin-like growth factor II | | | | 0.965 | | 0.986 | | 0.00 |  |
|  | Isoform B of Fibulin-1 | | | | 0.965 | | 0.986 | | 0.00 |  |
|  | Insulin-like growth factor-binding protein 4 precursor | | | | 0.972 | | 0.986 | | 0.00 |  |
|  | Apolipoprotein C-II precursor | | | | 0.972 | | 0.986 | | 0.00 |  |
|  | Alpha-1B-glycoprotein | | | | 0.973 | | 0.986 | | 0.00 |  |
|  | Isoform 1 of EGF-containing fibulin-like extracellular matrix protein 1 | | | | 0.982 | | 0.987 | | 0.00 |  |
|  | Isoform 1 of Cell surface glycoprotein MUC18 precursor | | | | 0.987 | | 0.987 | | 0.00 |  |
|  | Angiotensinogen precursor | | | | 0.987 | | 0.987 | | 0.00 |  |
|  | MD = mean difference (mean of concentrations for group with exercise minus mean for group without exercise) | | | | | | | | |  |
|  |  |  |  |  |  |  |  |  |  |  |

| **Supplementary Table S4.** VLCD effect for proteins identified from iTRAQ experiments. | | |  |
| --- | --- | --- | --- |
|  |  |  |  |
|  | **p-value** | **Adj. p-value** | **MD** |
| Biotinidase | 2.59E-09 | 6.00E-07 | -0.10 |
| Selenoprotein P | 1.56E-08 | 1.02E-06 | 0.15 |
| Insulin-like growth factor-binding protein 2 | 1.73E-08 | 1.02E-06 | 0.36 |
| Isoform 2 of Inter-alpha-trypsin inhibitor heavy chain H4 | 1.76E-08 | 1.02E-06 | 0.18 |
| Isoform 1 of Sex hormone-binding globulin | 3.58E-08 | 1.66E-06 | 0.46 |
| Isoform 3 of Interleukin-1 receptor accessory protein | 5.27E-08 | 2.04E-06 | 0.13 |
| Afamin precursor | 8.02E-07 | 2.61E-05 | -0.18 |
| Apolipoprotein A-IV precursor | 9.00E-07 | 2.61E-05 | -0.45 |
| Leucine-rich alpha-2-glycoprotein precursor | 1.04E-06 | 2.68E-05 | 0.20 |
| Beta-Ala-His dipeptidase | 1.44E-06 | 3.35E-05 | -0.21 |
| Leucine-rich alpha-2-glycoprotein precursor | 1.61E-06 | 3.39E-05 | 0.25 |
| Apolipoprotein A-IV precursor | 3.03E-06 | 5.86E-05 | -0.43 |
| Lysozyme C precursor | 3.67E-06 | 6.55E-05 | 0.11 |
| Pigment epithelium-derived factor precursor | 4.10E-06 | 6.80E-05 | -0.27 |
| Fructose-bisphosphate aldolase B | 1.60E-05 | 2.48E-04 | -0.25 |
| Cholinesterase precursor | 2.30E-05 | 3.34E-04 | -0.13 |
| Pigment epithelium-derived factor precursor | 2.45E-05 | 3.35E-04 | -0.20 |
| Cathepsin D | 3.09E-05 | 3.99E-04 | -0.15 |
| Protein AMBP | 3.29E-05 | 4.02E-04 | -0.13 |
| Aminopeptidase N | 1.01E-04 | 1.17E-03 | 0.08 |
| Isoform 2 of Neural cell adhesion molecule L1-like protein | 1.34E-04 | 1.48E-03 | 0.09 |
| Complement component C9 precursor | 1.80E-04 | 1.90E-03 | 0.18 |
| Isoform 1 of EGF-containing fibulin-like extracellular matrix protein 1 | 2.26E-04 | 2.23E-03 | 0.10 |
| Isoform 3 of Mannan-binding lectin serine protease 1 | 2.30E-04 | 2.23E-03 | -0.06 |
| Coagulation factor X precursor | 2.62E-04 | 2.35E-03 | -0.08 |
| Isoform 1 of Attractin | 2.63E-04 | 2.35E-03 | -0.06 |
| Monocyte differentiation antigen CD14 precursor | 3.02E-04 | 2.53E-03 | 0.08 |
| Isoform 1 of Phosphatidylinositol-glycan-specific phospholipase D precursor | 3.06E-04 | 2.53E-03 | -0.13 |
| Complement component C8 alpha chain precursor | 3.67E-04 | 2.94E-03 | 0.07 |
| Alpha-1-antitrypsin | 3.90E-04 | 3.01E-03 | 0.15 |
| alpha-2-glycoprotein 1, zinc | 4.25E-04 | 3.18E-03 | 0.07 |
| Pantetheinase precursor | 4.88E-04 | 3.54E-03 | -0.10 |
| Coagulation factor X | 5.18E-04 | 3.64E-03 | -0.13 |
| Isoform 1 of Contactin-1 precursor | 6.64E-04 | 4.53E-03 | 0.09 |
| Isoform 2 of Vascular non-inflammatory molecule 3 | 7.75E-04 | 5.14E-03 | -0.15 |
| Isoform 1 of Vascular cell adhesion protein 1 precursor | 1.02E-03 | 6.56E-03 | 0.08 |
| Isoform 1 of Inter-alpha-trypsin inhibitor heavy chain H3 | 1.13E-03 | 7.06E-03 | 0.07 |
| Protein Z-dependent protease inhibitor precursor | 1.20E-03 | 7.31E-03 | -0.06 |
| Transforming growth factor-beta-induced protein ig-h3 | 1.28E-03 | 7.61E-03 | -0.08 |
| Plastin-2 | 1.45E-03 | 8.42E-03 | 0.08 |
| Isoform 1 of Isocitrate dehydrogenase [NAD] subunit alpha, mitochondrial | 1.54E-03 | 8.73E-03 | 0.15 |
| Isoform 2 of Inter-alpha-trypsin inhibitor heavy chain H4 | 1.64E-03 | 9.04E-03 | 0.15 |
| Cartilage oligomeric matrix protein | 2.00E-03 | 1.08E-02 | -0.08 |
| Vitamin K-dependent protein C | 2.20E-03 | 1.16E-02 | 0.09 |
| Apolipoprotein C-III precursor | 2.74E-03 | 1.41E-02 | -0.17 |
| Plasma serine protease inhibitor precursor | 2.99E-03 | 1.51E-02 | -0.13 |
| Alpha-2-macroglobulin precursor | 3.25E-03 | 1.59E-02 | 0.15 |
| Corticosteroid-binding globulin precursor | 3.30E-03 | 1.59E-02 | 0.07 |
| Cystatin-C precursor | 3.45E-03 | 1.61E-02 | 0.12 |
| Insulin-like growth factor-binding protein 6 precursor | 3.47E-03 | 1.61E-02 | -0.07 |
| Corticosteroid-binding globulin precursor | 4.06E-03 | 1.85E-02 | 0.07 |
| Isoform 1 of Pregnancy zone protein | 4.76E-03 | 2.13E-02 | 0.16 |
| Plastin-2 | 5.27E-03 | 2.31E-02 | 0.09 |
| Retinoic acid receptor responder protein 2 precursor | 5.60E-03 | 2.40E-02 | -0.07 |
| HSPA5 protein | 5.98E-03 | 2.46E-02 | 0.04 |
| 30 kDa protein | 6.03E-03 | 2.46E-02 | 0.07 |
| Serum amyloid P-component precursor | 6.11E-03 | 2.46E-02 | -0.14 |
| Isoform 1 of Cartilage acidic protein 1 precursor | 6.16E-03 | 2.46E-02 | 0.05 |
| Isoform 1 of Low affinity immunoglobulin gamma Fc region receptor II-a | 6.38E-03 | 2.51E-02 | -0.06 |
| Antithrombin III variant | 6.63E-03 | 2.56E-02 | 0.11 |
| Isoform 1 of Vinculin | 6.78E-03 | 2.58E-02 | 0.10 |
| Fc-gamma receptor IIIb | 6.94E-03 | 2.60E-02 | -0.06 |
| Coagulation factor XII precursor | 7.45E-03 | 2.74E-02 | -0.11 |
| Muscle type neuropilin 1 | 8.98E-03 | 3.25E-02 | 0.07 |
| 72 kDa type IV collagenase | 9.38E-03 | 3.35E-02 | 0.06 |
| Clusterin precursor | 1.07E-02 | 3.77E-02 | 0.10 |
| Alpha-2-macroglobulin precursor | 1.28E-02 | 4.42E-02 | 0.15 |
| Complement factor I | 1.34E-02 | 4.58E-02 | -0.06 |
| SPARC-like protein 1 | 1.39E-02 | 4.68E-02 | 0.10 |
| Complement component C1q receptor | 1.55E-02 | 5.06E-02 | 0.05 |
| Isoform 1 of Coagulation factor XI | 1.55E-02 | 5.06E-02 | -0.05 |
| apolipoprotein A-IV precursor | 1.57E-02 | 5.06E-02 | -0.51 |
| Histidine-rich glycoprotein precursor | 1.75E-02 | 5.57E-02 | 0.05 |
| Isoform 1 of Sulfhydryl oxidase 1 precursor | 1.88E-02 | 5.90E-02 | -0.05 |
| Isoform 1 of C-reactive protein | 1.95E-02 | 6.02E-02 | -0.33 |
| Isoform 1 of Ectonucleotide pyrophosphatase/phosphodiesterase family member 2 | 1.97E-02 | 6.02E-02 | -0.09 |
| Complement-activating component of Ra-reactive factor precursor | 2.00E-02 | 6.03E-02 | -0.06 |
| Ceruloplasmin | 2.39E-02 | 7.11E-02 | 0.09 |
| Intercellular adhesion molecule 1 | 2.43E-02 | 7.12E-02 | -0.06 |
| Xaa-Pro dipeptidase | 2.45E-02 | 7.12E-02 | -0.13 |
| Complement C1s subcomponent | 2.54E-02 | 7.21E-02 | -0.10 |
| Apolipoprotein B-100 | 2.55E-02 | 7.21E-02 | 0.06 |
| Tetranectin precursor | 2.98E-02 | 8.24E-02 | 0.06 |
| Isoform 1 of Vitamin K-dependent protein Z precursor | 2.98E-02 | 8.24E-02 | -0.06 |
| Cadherin-13 precursor | 3.18E-02 | 8.68E-02 | 0.14 |
| Isoform 1 of Phosphatidylinositol-glycan-specific phospholipase D precursor | 3.25E-02 | 8.78E-02 | -0.06 |
| Thyroxine-binding globulin precursor | 3.43E-02 | 9.15E-02 | 0.06 |
| Isoform 1 of Insulin-like growth factor II | 3.90E-02 | 1.03E-01 | 0.05 |
| Alpha-1-acid glycoprotein 2 precursor | 4.02E-02 | 1.05E-01 | -0.14 |
| Isoform A of Coagulation factor VII | 4.51E-02 | 1.16E-01 | -0.05 |
| Isoform 1 of Fibrinogen alpha chain | 4.68E-02 | 1.18E-01 | 0.16 |
| Fibrinogen beta chain precursor | 4.69E-02 | 1.18E-01 | 0.19 |
| Isoform 1 of Carboxypeptidase B2 precursor | 4.77E-02 | 1.18E-01 | 0.05 |
| Uncharacterized protein KLKB1 | 4.80E-02 | 1.18E-01 | -0.04 |
| Membrane copper amine oxidase | 4.98E-02 | 1.22E-01 | -0.07 |
| Apolipoprotein C-I precursor | 5.17E-02 | 1.25E-01 | -0.08 |
| Putative uncharacterized protein ALB | 5.60E-02 | 1.34E-01 | 0.58 |
| Basement membrane-specific heparan sulfate proteoglycan core protein | 5.89E-02 | 1.39E-01 | 0.05 |
| Ceruloplasmin precursor | 5.92E-02 | 1.39E-01 | 0.08 |
| Vasorin precursor | 6.03E-02 | 1.40E-01 | 0.03 |
| HP protein | 6.34E-02 | 1.46E-01 | -0.04 |
| cDNA FLJ55673, highly similar to Complement factor B | 6.80E-02 | 1.55E-01 | -0.02 |
| Cholinesterase precursor | 7.70E-02 | 1.73E-01 | -0.04 |
| Insulin-like growth factor IA | 8.08E-02 | 1.80E-01 | 0.11 |
| Vitamin D-binding protein precursor | 8.85E-02 | 1.96E-01 | 0.03 |
| alpha-2-glycoprotein 1, zinc | 9.34E-02 | 2.03E-01 | 0.05 |
| Phosphatidylcholine-sterol acyltransferase precursor | 9.38E-02 | 2.03E-01 | 0.03 |
| Isoform 1 of CD166 antigen | 1.07E-01 | 2.30E-01 | 0.05 |
| Plasma protease C1 inhibitor | 1.13E-01 | 2.40E-01 | 0.04 |
| Ig lambda chain V-I region NIG-64 | 1.17E-01 | 2.46E-01 | -0.14 |
| Ig lambda chain V-IV region Hil | 1.18E-01 | 2.46E-01 | -0.14 |
| Complement C1s subcomponent | 1.22E-01 | 2.51E-01 | -0.03 |
| Complement C5 precursor | 1.22E-01 | 2.51E-01 | -0.08 |
| Isoform 1 of Mannan-binding lectin serine protease 2 precursor | 1.40E-01 | 2.85E-01 | -0.08 |
| Alpha-1-acid glycoprotein 2 | 1.46E-01 | 2.94E-01 | -0.13 |
| Prothrombin precursor (Fragment) | 1.48E-01 | 2.94E-01 | -0.04 |
| Complement component 6 precursor | 1.49E-01 | 2.94E-01 | 0.03 |
| Plasma serine protease inhibitor | 1.49E-01 | 2.94E-01 | -0.04 |
| Hepatocyte growth factor activator precursor | 1.53E-01 | 2.99E-01 | -0.02 |
| Hemoglobin subunit epsilon | 1.56E-01 | 3.03E-01 | 0.11 |
| ADP-ribosyl cyclase 2 precursor | 1.59E-01 | 3.04E-01 | 0.03 |
| Hemopexin precursor | 1.64E-01 | 3.11E-01 | -0.05 |
| Gamma-glutamyl hydrolase precursor | 1.69E-01 | 3.19E-01 | -0.02 |
| Isoform Gamma-B of Fibrinogen gamma chain | 1.80E-01 | 3.37E-01 | 0.11 |
| Thrombospondin-4 precursor | 1.88E-01 | 3.50E-01 | -0.05 |
| Carbonic anhydrase 1 | 1.96E-01 | 3.62E-01 | 0.13 |
| Angiotensinogen precursor | 1.99E-01 | 3.64E-01 | -0.05 |
| Cadherin-2 | 2.02E-01 | 3.66E-01 | 0.03 |
| Ig kappa chain V-IV region | 2.07E-01 | 3.70E-01 | -0.11 |
| Coagulation factor IX | 2.07E-01 | 3.70E-01 | 0.03 |
| Isoform 1 of N-acetylmuramoyl-L-alanine amidase precursor | 2.15E-01 | 3.81E-01 | -0.10 |
| Isoform 1 of Fibronectin | 2.19E-01 | 3.84E-01 | -0.09 |
| Serum paraoxonase/arylesterase 1 | 2.20E-01 | 3.84E-01 | 0.04 |
| MAN1A1 protein | 2.34E-01 | 4.03E-01 | -0.06 |
| Complement C5 precursor | 2.35E-01 | 4.03E-01 | -0.04 |
| Kallistatin precursor | 2.43E-01 | 4.14E-01 | -0.04 |
| 45 kDa protein | 2.46E-01 | 4.14E-01 | 0.03 |
| Glutathione peroxidase 3 precursor | 2.46E-01 | 4.14E-01 | 0.04 |
| Serpin peptidase inhibitor, clade D (Heparin cofactor), member 1 | 2.48E-01 | 4.15E-01 | -0.08 |
| tropomyosin 1 alpha chain isoform 2 | 2.56E-01 | 4.25E-01 | 0.12 |
| Isoform 2 of Multiple inositol polyphosphate phosphatase 1 | 2.76E-01 | 4.45E-01 | -0.02 |
| Isoform 2 of Collagen alpha-1(XVIII) chain precursor | 2.77E-01 | 4.45E-01 | -0.03 |
| Apolipoprotein A-I precursor | 2.78E-01 | 4.45E-01 | 0.05 |
| Apolipoprotein A-II precursor | 2.78E-01 | 4.45E-01 | 0.02 |
| Vitamin K-dependent protein S | 2.78E-01 | 4.45E-01 | 0.02 |
| Inter-alpha-trypsin inhibitor heavy chain H1 precursor | 2.85E-01 | 4.54E-01 | 0.02 |
| Complement C1r subcomponent-like protein | 2.90E-01 | 4.57E-01 | -0.03 |
| Carboxypeptidase N subunit 2 precursor | 2.94E-01 | 4.62E-01 | -0.02 |
| Inter-alpha-trypsin inhibitor heavy chain H2 | 3.00E-01 | 4.63E-01 | 0.02 |
| Carboxypeptidase N catalytic chain precursor | 3.00E-01 | 4.63E-01 | -0.06 |
| Apolipoprotein A-I precursor | 3.15E-01 | 4.85E-01 | 0.06 |
| Apolipoprotein C-II precursor | 3.41E-01 | 5.20E-01 | -0.04 |
| Isoform 1 of Cell surface glycoprotein MUC18 precursor | 3.48E-01 | 5.26E-01 | -0.04 |
| Isoform HMW of Kininogen-1 | 3.49E-01 | 5.26E-01 | 0.02 |
| Complement component C7 | 3.53E-01 | 5.28E-01 | -0.04 |
| Dopamine beta-hydroxylase | 3.74E-01 | 5.57E-01 | -0.03 |
| Complement component C7 | 3.90E-01 | 5.77E-01 | -0.01 |
| Fibrinogen beta chain precursor | 3.95E-01 | 5.80E-01 | 0.09 |
| Peroxiredoxin-1 | 4.06E-01 | 5.92E-01 | 0.06 |
| Hemopexin | 4.09E-01 | 5.94E-01 | -0.03 |
| Flavin reductase | 4.20E-01 | 6.05E-01 | 0.06 |
| Apolipoprotein B-100 precursor | 4.28E-01 | 6.08E-01 | 0.02 |
| cDNA FLJ55673, highly similar to Complement factor B | 4.32E-01 | 6.08E-01 | -0.01 |
| Isoform 1 of Extracellular matrix protein 1 | 4.32E-01 | 6.08E-01 | -0.03 |
| Isoform 1 of Peptidase inhibitor 16 precursor | 4.33E-01 | 6.08E-01 | -0.02 |
| Prothrombin (Fragment) | 4.43E-01 | 6.20E-01 | -0.03 |
| CD5 antigen-like precursor | 4.55E-01 | 6.32E-01 | 0.02 |
| Coagulation factor XIII B chain precursor | 4.61E-01 | 6.36E-01 | -0.02 |
| Isoform 1 of Collagen alpha-3(VI) chain | 4.67E-01 | 6.41E-01 | 0.02 |
| Complement C1r subcomponent precursor | 4.73E-01 | 6.42E-01 | -0.02 |
| Carboxypeptidase N catalytic chain precursor | 4.74E-01 | 6.42E-01 | -0.03 |
| Isoform XB of Tenascin-X | 4.76E-01 | 6.42E-01 | -0.02 |
| Lumican precursor | 4.89E-01 | 6.55E-01 | 0.02 |
| Angiogenin precursor | 5.06E-01 | 6.74E-01 | -0.02 |
| Uncharacterized protein FETUB | 5.12E-01 | 6.78E-01 | 0.03 |
| Isoform 3 of Neural cell adhesion molecule 1 | 5.47E-01 | 7.19E-01 | 0.01 |
| Isoform 1 of Gelsolin precursor | 5.49E-01 | 7.19E-01 | -0.02 |
| 4F2 cell-surface antigen heavy chain | 5.67E-01 | 7.39E-01 | 0.02 |
| Coagulation factor V | 5.80E-01 | 7.50E-01 | -0.02 |
| Complement component C8 beta chain precursor | 5.83E-01 | 7.50E-01 | -0.04 |
| Mannose-binding protein C precursor | 5.85E-01 | 7.50E-01 | 0.02 |
| Follistatin-related protein 1 | 5.98E-01 | 7.62E-01 | 0.02 |
| Galectin-3-binding protein | 6.09E-01 | 7.70E-01 | -0.02 |
| Angiotensinogen | 6.11E-01 | 7.70E-01 | -0.01 |
| Serum paraoxonase/arylesterase 1 | 6.15E-01 | 7.71E-01 | 0.02 |
| Ig kappa chain V-I region CAR | 6.30E-01 | 7.86E-01 | -0.03 |
| Complement factor D preproprotein | 6.38E-01 | 7.92E-01 | 0.01 |
| Dopamine beta-hydroxylase | 6.64E-01 | 8.14E-01 | -0.02 |
| Lumican precursor | 6.64E-01 | 8.14E-01 | 0.01 |
| Alpha-amylase 2B | 6.67E-01 | 8.14E-01 | 0.03 |
| Vitronectin precursor | 6.70E-01 | 8.14E-01 | -0.02 |
| Ig kappa chain V-I region AU | 6.75E-01 | 8.16E-01 | -0.02 |
| cDNA FLJ55606, highly similar to Alpha-2-HS-glycoprotein | 6.87E-01 | 8.23E-01 | 0.02 |
| AMBP protein precursor | 6.88E-01 | 8.23E-01 | 0.01 |
| Ig lambda chain V-I region NIG-64 | 6.91E-01 | 8.23E-01 | -0.02 |
| Properdin precursor | 7.20E-01 | 8.50E-01 | -0.01 |
| Insulin-like growth factor-binding protein 3 | 7.22E-01 | 8.50E-01 | -0.01 |
| Isoform LAMP-2A of Lysosome-associated membrane glycoprotein 2 | 7.26E-01 | 8.50E-01 | 0.01 |
| Isoform 2 of Carboxypeptidase B2 | 7.42E-01 | 8.65E-01 | 0.01 |
| similar to complement component 3 | 7.47E-01 | 8.66E-01 | -0.02 |
| Transforming growth factor-beta-induced protein ig-h3 precursor | 7.59E-01 | 8.72E-01 | -0.01 |
| Coagulation factor XIII A chain | 7.59E-01 | 8.72E-01 | 0.01 |
| Cadherin-5 | 7.72E-01 | 8.82E-01 | -0.01 |
| Ig mu heavy chain disease protein | 7.84E-01 | 8.86E-01 | -0.03 |
| Plasma glutamate carboxypeptidase | 7.85E-01 | 8.86E-01 | 0.01 |
| Serotransferrin | 7.87E-01 | 8.86E-01 | -0.01 |
| Procollagen C-endopeptidase enhancer 1 | 7.91E-01 | 8.86E-01 | 0.01 |
| Apolipoprotein E | 8.27E-01 | 9.22E-01 | 0.01 |
| Complement C4-A | 8.30E-01 | 9.22E-01 | 0.01 |
| Procollagen C-endopeptidase enhancer 1 precursor | 8.37E-01 | 9.25E-01 | -0.01 |
| immunoglobulin J chain | 8.48E-01 | 9.33E-01 | 0.01 |
| Ribonuclease pancreatic precursor | 8.58E-01 | 9.39E-01 | 0.00 |
| Protein AMBP | 8.76E-01 | 9.50E-01 | -0.01 |
| Insulin-like growth factor-binding protein 4 precursor | 8.76E-01 | 9.50E-01 | 0.01 |
| Reticulon-4 receptor-like 2 precursor | 8.92E-01 | 9.58E-01 | 0.01 |
| Mannosyl-oligosaccharide 1,2-alpha-mannosidase IA | 8.94E-01 | 9.58E-01 | 0.01 |
| Complement C4-A | 8.96E-01 | 9.58E-01 | 0.00 |
| Alpha-1B-glycoprotein | 9.01E-01 | 9.59E-01 | 0.00 |
| Complement C5 precursor | 9.19E-01 | 9.74E-01 | 0.00 |
| Serotransferrin precursor | 9.27E-01 | 9.77E-01 | 0.00 |
| von Willebrand factor | 9.34E-01 | 9.80E-01 | 0.00 |
| Reticulon-4 receptor-like 2 precursor | 9.41E-01 | 9.83E-01 | 0.00 |
| Hepatocyte growth factor-like protein | 9.53E-01 | 9.91E-01 | 0.00 |
| Transferrin receptor protein 1 | 9.66E-01 | 9.93E-01 | 0.00 |
| Immunoglobulin superfamily containing leucine-rich repeat protein precursor | 9.68E-01 | 9.93E-01 | 0.00 |
| Insulin-like growth factor-binding protein 5 | 9.71E-01 | 9.93E-01 | 0.00 |
| Endothelial protein C receptor precursor | 9.71E-01 | 9.93E-01 | 0.00 |
| GUGU beta form | 9.77E-01 | 9.94E-01 | 0.00 |
| Coagulation factor IX precursor | 9.89E-01 | 9.94E-01 | 0.00 |
| Insulin-like growth factor-binding protein complex acid labile chain | 9.90E-01 | 9.94E-01 | 0.00 |
| Isoform B of Fibulin-1 | 9.90E-01 | 9.94E-01 | 0.00 |
| Isoform 1 of Multiple inositol polyphosphate phosphatase 1 precursor | 9.97E-01 | 9.97E-01 | 0.00 |
| MD = mean difference (mean of concentrations after VLCD minus mean at baseline). Analytes are considered significant if the p-value is less than 0.05 and the adjusted p-value is less than 0.1 | | | |
|

**Supplementary Table S5. Peptide mapping iTRAQ experiments.**

| **Peptide sequence** | **PROTEIN, ACC** | **Protein description** | **Gene Symbol** |
| --- | --- | --- | --- |
| GQLLPGGAR | IPI00043567 | hypothetical protein |  |
| VLLDGVQNLR | IPI00887739 | hypothetical protein, partial |  |
| BTVYLQMBSLR | IPI00382481 | Ig heavy chain V-III region BUT |  |
| EVQLVETGGGLIQPGGSLR | IPI00382481 | Ig heavy chain V-III region BUT |  |
| BTLYLQMNSLR | IPI00382482 | Ig heavy chain V-III region CAM |  |
| LSCAASGFTFSNYAMHWVR | IPI00382482 | Ig heavy chain V-III region CAM |  |
| DIQLTQSPSSLSASVGDR | IPI00385555, IPI00829836 | Ig kappa chain V-I region BAN |  |
| DIQMTQSPSSLSVSVGDR | IPI00387097 | Ig kappa chain V-I region Lay |  |
| ZIVLTZSPGTLSLSPGZR | IPI00387113 | Ig kappa chain V-III region B6 |  |
| LEPEDFAVYYCQQYGSSPR | IPI00385252 | Ig kappa chain V-III region GOL |  |
| ASQSVSNSYLAWYQQKPGQAPR | IPI00387115 | Ig kappa chain V-III region SIE |  |
| AAPSVTLFPPSSEELQANK | IPI00942688, IPI00941543, IPI00013438, IPI00744476, IPI00383016, IPI00943342, IPI00386134, IPI00940052, IPI00017457, IPI00784935, IPI00382420, IPI00154742, IPI00829640, IPI00827875, IPI00829877, IPI00385985, IPI00807428, IPI00939270, IPI00852577, IPI00887169, IPI00940807, IPI00827923, IPI00939461, IPI00719373, IPI00385254, IPI00386839, IPI00719452, IPI00880175, IPI00893178, IPI00658130, IPI00830047, IPI00815938, IPI00788824, IPI00939627, IPI00003939, IPI00940101, IPI00450309, IPI00829740, IPI00642632 | Ig lambda chain V-I region NIG-64 |  |
| AGVETTKPSK | IPI00942688, IPI00941543, IPI00013438, IPI00744476, IPI00383016, IPI00943342, IPI00386134, IPI00940052, IPI00017457, IPI00784935, IPI00382420, IPI00154742, IPI00829640, IPI00827875, IPI00829877, IPI00385985, IPI00807428, IPI00939270, IPI00852577, IPI00887169, IPI00940807, IPI00827923, IPI00939461, IPI00719373, IPI00385254, IPI00386839, IPI00719452, IPI00880175, IPI00893178, IPI00658130, IPI00830047, IPI00815938, IPI00788824, IPI00939627, IPI00003939, IPI00940101, IPI00450309, IPI00829740, IPI00642632 | Ig lambda chain V-I region NIG-64 |  |
| AGVETTTPSK | IPI00942688, IPI00941543, IPI00013438, IPI00744476, IPI00383016, IPI00943342, IPI00386134, IPI00940052, IPI00017457, IPI00784935, IPI00382420, IPI00154742, IPI00829640, IPI00827875, IPI00829877, IPI00385985, IPI00807428, IPI00939270, IPI00852577, IPI00887169, IPI00940807, IPI00827923, IPI00939461, IPI00719373, IPI00385254, IPI00386839, IPI00719452, IPI00880175, IPI00893178, IPI00658130, IPI00830047, IPI00815938, IPI00788824, IPI00939627, IPI00003939, IPI00940101, IPI00450309, IPI00829740, IPI00642632 | Ig lambda chain V-I region NIG-64 |  |
| ANPTVTLFPPSSEELQANK | IPI00942688, IPI00941543, IPI00013438, IPI00744476, IPI00383016, IPI00943342, IPI00386134, IPI00940052, IPI00017457, IPI00784935, IPI00382420, IPI00154742, IPI00829640, IPI00827875, IPI00829877, IPI00385985, IPI00807428, IPI00939270, IPI00852577, IPI00887169, IPI00940807, IPI00827923, IPI00939461, IPI00719373, IPI00385254, IPI00386839, IPI00719452, IPI00880175, IPI00893178, IPI00658130, IPI00830047, IPI00815938, IPI00788824, IPI00939627, IPI00003939, IPI00940101, IPI00450309, IPI00829740, IPI00642632 | Ig lambda chain V-I region NIG-64 |  |
| ATLVCLISDFYPGAVTVAWK | IPI00942688, IPI00941543, IPI00013438, IPI00744476, IPI00383016, IPI00943342, IPI00386134, IPI00940052, IPI00017457, IPI00784935, IPI00382420, IPI00154742, IPI00829640, IPI00827875, IPI00829877, IPI00385985, IPI00807428, IPI00939270, IPI00852577, IPI00887169, IPI00940807, IPI00827923, IPI00939461, IPI00719373, IPI00385254, IPI00386839, IPI00719452, IPI00880175, IPI00893178, IPI00658130, IPI00830047, IPI00815938, IPI00788824, IPI00939627, IPI00003939, IPI00940101, IPI00450309, IPI00829740, IPI00642632 | Ig lambda chain V-I region NIG-64 |  |
| FSGSNSGNTATLTISR | IPI00942688, IPI00941543, IPI00013438, IPI00744476, IPI00383016, IPI00943342, IPI00386134, IPI00940052, IPI00017457, IPI00784935, IPI00382420, IPI00154742, IPI00829640, IPI00827875, IPI00829877, IPI00385985, IPI00807428, IPI00939270, IPI00852577, IPI00887169, IPI00940807, IPI00827923, IPI00939461, IPI00719373, IPI00385254, IPI00386839, IPI00719452, IPI00880175, IPI00893178, IPI00658130, IPI00830047, IPI00815938, IPI00788824, IPI00939627, IPI00003939, IPI00940101, IPI00450309, IPI00829740, IPI00642632 | Ig lambda chain V-I region NIG-64 |  |
| LLIYDNNK | IPI00942688, IPI00941543, IPI00013438, IPI00744476, IPI00383016, IPI00943342, IPI00386134, IPI00940052, IPI00017457, IPI00784935, IPI00382420, IPI00154742, IPI00829640, IPI00827875, IPI00829877, IPI00385985, IPI00807428, IPI00939270, IPI00852577, IPI00887169, IPI00940807, IPI00827923, IPI00939461, IPI00719373, IPI00385254, IPI00386839, IPI00719452, IPI00880175, IPI00893178, IPI00658130, IPI00830047, IPI00815938, IPI00788824, IPI00939627, IPI00003939, IPI00940101, IPI00450309, IPI00829740, IPI00642632 | Ig lambda chain V-I region NIG-64 |  |
| LTVLGQPK | IPI00942688, IPI00941543, IPI00013438, IPI00744476, IPI00383016, IPI00943342, IPI00386134, IPI00940052, IPI00017457, IPI00784935, IPI00382420, IPI00154742, IPI00829640, IPI00827875, IPI00829877, IPI00385985, IPI00807428, IPI00939270, IPI00852577, IPI00887169, IPI00940807, IPI00827923, IPI00939461, IPI00719373, IPI00385254, IPI00386839, IPI00719452, IPI00880175, IPI00893178, IPI00658130, IPI00830047, IPI00815938, IPI00788824, IPI00939627, IPI00003939, IPI00940101, IPI00450309, IPI00829740, IPI00642632 | Ig lambda chain V-I region NIG-64 |  |
| LTVLSQPK | IPI00942688, IPI00941543, IPI00013438, IPI00744476, IPI00383016, IPI00943342, IPI00386134, IPI00940052, IPI00017457, IPI00784935, IPI00382420, IPI00154742, IPI00829640, IPI00827875, IPI00829877, IPI00385985, IPI00807428, IPI00939270, IPI00852577, IPI00887169, IPI00940807, IPI00827923, IPI00939461, IPI00719373, IPI00385254, IPI00386839, IPI00719452, IPI00880175, IPI00893178, IPI00658130, IPI00830047, IPI00815938, IPI00788824, IPI00939627, IPI00003939, IPI00940101, IPI00450309, IPI00829740, IPI00642632 | Ig lambda chain V-I region NIG-64 |  |
| SGTSASLAISGLR | IPI00942688, IPI00941543, IPI00013438, IPI00744476, IPI00383016, IPI00943342, IPI00386134, IPI00940052, IPI00017457, IPI00784935, IPI00382420, IPI00154742, IPI00829640, IPI00827875, IPI00829877, IPI00385985, IPI00807428, IPI00939270, IPI00852577, IPI00887169, IPI00940807, IPI00827923, IPI00939461, IPI00719373, IPI00385254, IPI00386839, IPI00719452, IPI00880175, IPI00893178, IPI00658130, IPI00830047, IPI00815938, IPI00788824, IPI00939627, IPI00003939, IPI00940101, IPI00450309, IPI00829740, IPI00642632 | Ig lambda chain V-I region NIG-64 |  |
| SYSCQVTHEGSTVEK | IPI00942688, IPI00941543, IPI00013438, IPI00744476, IPI00383016, IPI00943342, IPI00386134, IPI00940052, IPI00017457, IPI00784935, IPI00382420, IPI00154742, IPI00829640, IPI00827875, IPI00829877, IPI00385985, IPI00807428, IPI00939270, IPI00852577, IPI00887169, IPI00940807, IPI00827923, IPI00939461, IPI00719373, IPI00385254, IPI00386839, IPI00719452, IPI00880175, IPI00893178, IPI00658130, IPI00830047, IPI00815938, IPI00788824, IPI00939627, IPI00003939, IPI00940101, IPI00450309, IPI00829740, IPI00642632 | Ig lambda chain V-I region NIG-64 |  |
| VTVLGQPK | IPI00942688, IPI00941543, IPI00013438, IPI00744476, IPI00383016, IPI00943342, IPI00386134, IPI00940052, IPI00017457, IPI00784935, IPI00382420, IPI00154742, IPI00829640, IPI00827875, IPI00829877, IPI00385985, IPI00807428, IPI00939270, IPI00852577, IPI00887169, IPI00940807, IPI00827923, IPI00939461, IPI00719373, IPI00385254, IPI00386839, IPI00719452, IPI00880175, IPI00893178, IPI00658130, IPI00830047, IPI00815938, IPI00788824, IPI00939627, IPI00003939, IPI00940101, IPI00450309, IPI00829740, IPI00642632 | Ig lambda chain V-I region NIG-64 |  |
| YAASSYLSLTPEQWK | IPI00942688, IPI00941543, IPI00013438, IPI00744476, IPI00383016, IPI00943342, IPI00386134, IPI00940052, IPI00017457, IPI00784935, IPI00382420, IPI00154742, IPI00829640, IPI00827875, IPI00829877, IPI00385985, IPI00807428, IPI00939270, IPI00852577, IPI00887169, IPI00940807, IPI00827923, IPI00939461, IPI00719373, IPI00385254, IPI00386839, IPI00719452, IPI00880175, IPI00893178, IPI00658130, IPI00830047, IPI00815938, IPI00788824, IPI00939627, IPI00003939, IPI00940101, IPI00450309, IPI00829740, IPI00642632 | Ig lambda chain V-I region NIG-64 |  |
| YAASSYLSLTPEQWR | IPI00942688, IPI00941543, IPI00013438, IPI00744476, IPI00383016, IPI00943342, IPI00386134, IPI00940052, IPI00017457, IPI00784935, IPI00382420, IPI00154742, IPI00829640, IPI00827875, IPI00829877, IPI00385985, IPI00807428, IPI00939270, IPI00852577, IPI00887169, IPI00940807, IPI00827923, IPI00939461, IPI00719373, IPI00385254, IPI00386839, IPI00719452, IPI00880175, IPI00893178, IPI00658130, IPI00830047, IPI00815938, IPI00788824, IPI00939627, IPI00003939, IPI00940101, IPI00450309, IPI00829740, IPI00642632 | Ig lambda chain V-I region NIG-64 |  |
| YVLTQPPSVSVAPGETAR | IPI00385985 | Ig lambda chain V-III region LOI |  |
| SYELTQPPSVSVSPGQTAR | IPI00550162, IPI00382440, IPI00747752, IPI00941756 | Ig lambda chain V-IV region Hil |  |
| YELTQPPSVSVSPGQTATISCSGDK | IPI00386576 | Ig lambda chain V-IV region MOL |  |
| VEDTAVYYCAR | IPI00940245 | Immunoglobulin heavy chain variant (Fragment) |  |
| ALEWLALIYWDDDKR | IPI00007906 | Myosin-reactive immunoglobulin heavy chain variable region (Fragment) |  |
| LITSQAMDILR | IPI00022913 | PRO1575 |  |
| NFPPSQDASGDLCTTSSQLTLPATQCPDGK | IPI00784758 | Putative uncharacterized protein DKFZp686M08189 |  |
| GTTALVPLLLLLK | IPI00477087 | Putative uncharacterized protein ENSP00000349693 (Fragment) |  |
| LFEHPLYR | IPI00892908, IPI00470607, IPI00894513 | family with sequence similarity 20, member C | IGI17711851 |
| LPPAAEPAER | IPI00892908, IPI00470607, IPI00894513 | family with sequence similarity 20, member C | IGI17711851 |
| VAVPPLTEEDVLFNVNSDTR | IPI00892908, IPI00470607, IPI00894513 | family with sequence similarity 20, member C | IGI17711851 |
| GQSEDPGSLLSLFR | IPI00554702, IPI00027493, IPI00554722, IPI00604710, IPI00554611, IPI00554481 | 4F2 cell-surface antigen heavy chain | 4F2 |
| LDYLSSLK | IPI00554702, IPI00027493, IPI00554722, IPI00604710, IPI00554611, IPI00554481 | 4F2 cell-surface antigen heavy chain | 4F2 |
| LLTSFLPAQLLR | IPI00554702, IPI00027493, IPI00554722, IPI00604710, IPI00554611, IPI00554481 | 4F2 cell-surface antigen heavy chain | 4F2 |
| VILDLTPNYR | IPI00554702, IPI00027493, IPI00554722, IPI00604710, IPI00554611, IPI00554481 | 4F2 cell-surface antigen heavy chain | 4F2 |
| CEGPIPDVTFELLR | IPI00902880, IPI00646799, IPI00022895, IPI00644018 | Alpha-1B-glycoprotein | A1BG |
| CLAPLEGAR | IPI00902880, IPI00646799, IPI00022895, IPI00644018 | Alpha-1B-glycoprotein | A1BG |
| LELHVDGPPPRPQLR | IPI00902880, IPI00646799, IPI00022895, IPI00644018 | Alpha-1B-glycoprotein | A1BG |
| LHDNQNGWSGDSAPVELILSDETLPAPEFSPEPESGR | IPI00902880, IPI00646799, IPI00022895, IPI00644018 | Alpha-1B-glycoprotein | A1BG |
| TPGAAANLELIFVGPQHAGNYR | IPI00902880, IPI00646799, IPI00022895, IPI00644018 | Alpha-1B-glycoprotein | A1BG |
| VTLTCVAPLSGVDFQLR | IPI00902880, IPI00646799, IPI00022895, IPI00644018 | Alpha-1B-glycoprotein | A1BG |
| LLELTGPK | IPI00022895 | Alpha-1B-glycoprotein precursor | A1BG |
| NGVAQEPVHLDSPAIK | IPI00022895 | Alpha-1B-glycoprotein precursor | A1BG |
| SGLSTGWTQLSK | IPI00022895 | Alpha-1B-glycoprotein precursor | A1BG |
| SLPAPWLSMAPVSWITPGLK | IPI00022895 | Alpha-1B-glycoprotein precursor | A1BG |
| GHFSISIPVK | IPI00789547 | 19 kDa protein | A2M |
| AAQVTIQSSGTFSSK | IPI00478003 | Alpha-2-macroglobulin precursor | A2M |
| AHTSFQISLSVSYTGSR | IPI00478003 | Alpha-2-macroglobulin precursor | A2M |
| AIGYLNTGYQR | IPI00478003 | Alpha-2-macroglobulin precursor | A2M |
| ALLAYAFALAGNQDK | IPI00478003 | Alpha-2-macroglobulin precursor | A2M |
| ALLAYAFALAGNQDKR | IPI00478003 | Alpha-2-macroglobulin precursor | A2M |
| AVDQSVLLMKPDAELSASSVYNLLPEK | IPI00478003 | Alpha-2-macroglobulin precursor | A2M |
| AYIFIDEAHITQALIWLSQR | IPI00478003 | Alpha-2-macroglobulin precursor | A2M |
| DLTGFPGPLNDQDDEDCINR | IPI00478003 | Alpha-2-macroglobulin precursor | A2M |
| DTVIKPLLVEPEGLEK | IPI00478003 | Alpha-2-macroglobulin precursor | A2M |
| EQAPHCICANGR | IPI00478003 | Alpha-2-macroglobulin precursor | A2M |
| ETTFNSLLCPSGGEVSEELSLK | IPI00478003 | Alpha-2-macroglobulin precursor | A2M |
| ETTFNSLLCPSGGEVSEELSLKLPPNVVEESAR | IPI00478003 | Alpha-2-macroglobulin precursor | A2M |
| FEVQVTVPK | IPI00478003 | Alpha-2-macroglobulin precursor | A2M |
| FQVDNNNR | IPI00478003 | Alpha-2-macroglobulin precursor | A2M |
| FRVVSMDENFHPLNELIPLVYIQDPK | IPI00478003 | Alpha-2-macroglobulin precursor | A2M |
| FSGQLNSHGCFYQQVK | IPI00478003 | Alpha-2-macroglobulin precursor | A2M |
| GEAFTLK | IPI00478003 | Alpha-2-macroglobulin precursor | A2M |
| GVPIPNKVIFIR | IPI00478003 | Alpha-2-macroglobulin precursor | A2M |
| HYDGSYSTFGER | IPI00478003 | Alpha-2-macroglobulin precursor | A2M |
| IAQWQSFQLEGGLK | IPI00478003 | Alpha-2-macroglobulin precursor | A2M |
| KDTVIKPLLVEPEGLEK | IPI00478003 | Alpha-2-macroglobulin precursor | A2M |
| KYSDASDCHGEDSQAFCEK | IPI00478003 | Alpha-2-macroglobulin precursor | A2M |
| LHTEAQIQEEGTVVELTGR | IPI00478003 | Alpha-2-macroglobulin precursor | A2M |
| LLLQQVSLPELPGEYSMK | IPI00478003 | Alpha-2-macroglobulin precursor | A2M |
| LPPNVVEESAR | IPI00478003 | Alpha-2-macroglobulin precursor | A2M |
| LVHVEEPHTETVR | IPI00478003 | Alpha-2-macroglobulin precursor | A2M |
| NALFCLESAWK | IPI00478003 | Alpha-2-macroglobulin precursor | A2M |
| QFSFPLSSEPFQGSYK | IPI00478003 | Alpha-2-macroglobulin precursor | A2M |
| QGIPFFGQVR | IPI00478003 | Alpha-2-macroglobulin precursor | A2M |
| QQNAQGGFSSTQDTVVALHALSK | IPI00478003 | Alpha-2-macroglobulin precursor | A2M |
| QQNAQGGFSSTQDTVVALHALSKYGAATFTR | IPI00478003 | Alpha-2-macroglobulin precursor | A2M |
| QTVSWAVTPK | IPI00478003 | Alpha-2-macroglobulin precursor | A2M |
| SASNMAIVDVK | IPI00478003 | Alpha-2-macroglobulin precursor | A2M |
| SLNEEAVK | IPI00478003 | Alpha-2-macroglobulin precursor | A2M |
| TEHPFTVEEFVLPK | IPI00478003 | Alpha-2-macroglobulin precursor | A2M |
| VGFYESDVMGR | IPI00478003 | Alpha-2-macroglobulin precursor | A2M |
| VSNQTLSLFFTVLQDVPVR | IPI00478003 | Alpha-2-macroglobulin precursor | A2M |
| VSVQLEASPAFLAVPVEK | IPI00478003 | Alpha-2-macroglobulin precursor | A2M |
| VTGEGCVYLQTSLK | IPI00478003 | Alpha-2-macroglobulin precursor | A2M |
| VVSMDENFHPLNELIPLVYIQDPK | IPI00478003 | Alpha-2-macroglobulin precursor | A2M |
| VYDYYETDEFAIAEYNAPCSK | IPI00478003 | Alpha-2-macroglobulin precursor | A2M |
| YNILPEKEEFPFALGVQTLPQTCDEPK | IPI00478003 | Alpha-2-macroglobulin precursor | A2M |
| YSDASDCHGEDSQAFCEK | IPI00478003 | Alpha-2-macroglobulin precursor | A2M |
| FYNIGDQR | IPI00440825, IPI00911107, IPI00419966, IPI00440822, IPI00939199 | Isoform 2 of Target of Nesh-SH3 | ABI3BP |
| NPLGEGPVSNTVAFSTESADPR | IPI00440825, IPI00911107, IPI00419966, IPI00440822, IPI00939199 | Isoform 2 of Target of Nesh-SH3 | ABI3BP |
| EVVIVSATR | IPI00030363, IPI00440499, IPI00062003 | Acetyl-CoA acetyltransferase, mitochondrial precursor | ACAT1 |
| KYEDLLWAWEGWR | IPI00909226, IPI00877100, IPI00178017, IPI00028147, IPI00796552, IPI00437751 | Isoform Testis-specific of Angiotensin-converting enzyme | ACE |
| DITYFIQQLLR | IPI00007068, IPI00642069, IPI00892652, IPI00888282, IPI00028091, IPI00554433 | Actin-related protein 3 | ACRP3 |
| LAIFGSVR | IPI00297635, IPI00402513 | Isoform 1 of Acyl-coenzyme A synthetase ACSM3, mitochondrial precursor | ACSM3 |
| AVFPSIVGRPR | IPI00894498, IPI00739539, IPI00917545, IPI00930226, IPI00021439, IPI00740545, IPI00942659, IPI00921887, IPI00008603, IPI00917282, IPI00894365, IPI00556391, IPI00645534, IPI00893981, IPI00514530, IPI00935352, IPI00021428, IPI00887316, IPI00479743, IPI00893604, IPI00916212, IPI00414057, IPI00878173, IPI00894523, IPI00917457, IPI00927545, IPI00003269, IPI00025416, IPI00023006, IPI00930066, IPI00796881, IPI00888712, IPI00021440, IPI00790339, IPI00555733, IPI00922693, IPI00930343, IPI00917820 | Beta-actin-like protein 2 | ACTBL2 |
| SYELPDGQVITIGNER | IPI00894498, IPI00739539, IPI00917545, IPI00930226, IPI00021439, IPI00740545, IPI00942659, IPI00921887, IPI00008603, IPI00917282, IPI00894365, IPI00556391, IPI00645534, IPI00893981, IPI00514530, IPI00935352, IPI00021428, IPI00887316, IPI00479743, IPI00893604, IPI00916212, IPI00414057, IPI00878173, IPI00894523, IPI00917457, IPI00927545, IPI00003269, IPI00025416, IPI00023006, IPI00930066, IPI00796881, IPI00888712, IPI00021440, IPI00790339, IPI00555733, IPI00922693, IPI00930343, IPI00917820 | Beta-actin-like protein 2 | ACTBL2 |
| VAPEEHPVLLTEAPLNPK | IPI00894498, IPI00739539, IPI00917545, IPI00930226, IPI00021439, IPI00740545, IPI00942659, IPI00921887, IPI00008603, IPI00917282, IPI00894365, IPI00556391, IPI00645534, IPI00893981, IPI00514530, IPI00935352, IPI00021428, IPI00887316, IPI00479743, IPI00893604, IPI00916212, IPI00414057, IPI00878173, IPI00894523, IPI00917457, IPI00927545, IPI00003269, IPI00025416, IPI00023006, IPI00930066, IPI00796881, IPI00888712, IPI00021440, IPI00790339, IPI00555733, IPI00922693, IPI00930343, IPI00917820 | Beta-actin-like protein 2 | ACTBL2 |
| GYEEWLLNEIR | IPI00013508, IPI00759776, IPI00019884, IPI00908458, IPI00909239, IPI00013808, IPI00921118 | Alpha-actinin-1 | ACTN1 |
| AGFALDEGIANPTDAFTVFYSER | IPI00792542, IPI00791053, IPI00940464, IPI00788719, IPI00009268 | 16 kDa protein | ACY1 |
| LHEAVFLR | IPI00792542, IPI00791053, IPI00940464, IPI00788719, IPI00009268 | 16 kDa protein | ACY1 |
| AGAQQPAVALETCNPQPCPAR | IPI00453457, IPI00334996, IPI00449028, IPI00645987, IPI00427822 | Putative uncharacterized protein C9orf8 | ADAMTS13 |
| LLPGPQENSVQSSACGR | IPI00453457, IPI00334996, IPI00449028, IPI00645987, IPI00427822 | Putative uncharacterized protein C9orf8 | ADAMTS13 |
| QAWVWAAVR | IPI00453457, IPI00334996, IPI00449028, IPI00645987, IPI00427822 | Putative uncharacterized protein C9orf8 | ADAMTS13 |
| SLVELTPIAAVHGR | IPI00453457, IPI00334996, IPI00449028, IPI00645987, IPI00427822 | Putative uncharacterized protein C9orf8 | ADAMTS13 |
| YGSQLAPETFYR | IPI00453457, IPI00334996, IPI00449028, IPI00645987, IPI00427822 | Putative uncharacterized protein C9orf8 | ADAMTS13 |
| MLSPGFDSSVYSDLCEAAEAVRPEER | IPI00790458, IPI00737614, IPI00644346 | ADAMTS-like protein 2 precursor | ADAMTSL2 |
| ACSQAPCPPEQPDPR | IPI00896415, IPI00374068, IPI00550741 | ADAMTSL4 protein | ADAMTSL4 |
| ILWIPAGALR | IPI00896415, IPI00374068, IPI00550741 | ADAMTSL4 protein | ADAMTSL4 |
| GDIGETGVPGAEGPR | IPI00020019 | Adiponectin precursor | ADIPOQ |
| SAFSVGLETYVTIPNMPIR | IPI00020019 | Adiponectin precursor | ADIPOQ |
| AESPEVCFNEESPK | IPI00019943 | Afamin precursor | AFM |
| AFSSYQK | IPI00019943 | Afamin precursor | AFM |
| AIPVTQYLK | IPI00019943 | Afamin precursor | AFM |
| DADPDTFFAK | IPI00019943 | Afamin precursor | AFM |
| DMVEYK | IPI00019943 | Afamin precursor | AFM |
| DMVEYKDR | IPI00019943 | Afamin precursor | AFM |
| ELISLVEDVSSNYDGCCEGDVVQCIR | IPI00019943 | Afamin precursor | AFM |
| ESLLNHFLYEVAR | IPI00019943 | Afamin precursor | AFM |
| FLVNLVK | IPI00019943 | Afamin precursor | AFM |
| FTDSENVCQER | IPI00019943 | Afamin precursor | AFM |
| FTFEYSR | IPI00019943 | Afamin precursor | AFM |
| GQCIINSNK | IPI00019943 | Afamin precursor | AFM |
| GQCIINSNKDDRPK | IPI00019943 | Afamin precursor | AFM |
| HELTDEELQSLFTNFANVVDK | IPI00019943 | Afamin precursor | AFM |
| HFQNLGK | IPI00019943 | Afamin precursor | AFM |
| HPDLSIPELLR | IPI00019943 | Afamin precursor | AFM |
| IAPQLSTEELVSLGEK | IPI00019943 | Afamin precursor | AFM |
| ICAMEGLPQK | IPI00019943 | Afamin precursor | AFM |
| IEFKELISLVEDVSSNYDGCCEGDVVQCIR | IPI00019943 | Afamin precursor | AFM |
| IKECCEK | IPI00019943 | Afamin precursor | AFM |
| IVQIYKDLLR | IPI00019943 | Afamin precursor | AFM |
| KSDVGFLPPFPTLDPEEK | IPI00019943 | Afamin precursor | AFM |
| LCFFYNK | IPI00019943 | Afamin precursor | AFM |
| LKHELTDEELQSLFTNFANVVDK | IPI00019943 | Afamin precursor | AFM |
| LPNNVLQEK | IPI00019943 | Afamin precursor | AFM |
| MVQQECK | IPI00019943 | Afamin precursor | AFM |
| NPFVFAPTLLTVAVHFEEVAK | IPI00019943 | Afamin precursor | AFM |
| RHPDLSIPELLR | IPI00019943 | Afamin precursor | AFM |
| RLCFFYNK | IPI00019943 | Afamin precursor | AFM |
| RNPFVFAPTLLTVAVHFEEVAK | IPI00019943 | Afamin precursor | AFM |
| RPCFESLK | IPI00019943 | Afamin precursor | AFM |
| SCCEEQNKVNCLQTR | IPI00019943 | Afamin precursor | AFM |
| SDVGFLPPFPTLDPEEK | IPI00019943 | Afamin precursor | AFM |
| TINPAVDHCCK | IPI00019943 | Afamin precursor | AFM |
| TYVPPPFSQDLFTFHADMCQSQNEELQR | IPI00019943 | Afamin precursor | AFM |
| VMNHICSK | IPI00019943 | Afamin precursor | AFM |
| VNCLQTR | IPI00019943 | Afamin precursor | AFM |
| VVHFIYIAILSQK | IPI00019943 | Afamin precursor | AFM |
| VVHFIYIAILSQKFPK | IPI00019943 | Afamin precursor | AFM |
| YHYLIR | IPI00019943 | Afamin precursor | AFM |
| FLPSYQAVEYMR | IPI00026259 | N(4)-(beta-N-acetylglucosaminyl)-L-asparaginase | AGA |
| AAMVGMLANFLGFR | IPI00032220, IPI00908365 | Angiotensinogen | AGT |
| ALQDQLVLVAAK | IPI00032220, IPI00908365 | Angiotensinogen | AGT |
| ALQDQLVLVAAKLDTEDKLR | IPI00032220, IPI00908365 | Angiotensinogen | AGT |
| ANAGKPKDPTFIPAPIQAK | IPI00032220, IPI00908365 | Angiotensinogen | AGT |
| DPTFIPAPIQAK | IPI00032220, IPI00908365 | Angiotensinogen | AGT |
| FMQAVTGWK | IPI00032220, IPI00908365 | Angiotensinogen | AGT |
| LDTEDKLR | IPI00032220, IPI00908365 | Angiotensinogen | AGT |
| TGCSLMGASVDSTLAFNTYVHFQGK | IPI00032220, IPI00908365 | Angiotensinogen | AGT |
| VEGLTFQQNSLNWMK | IPI00032220, IPI00908365 | Angiotensinogen | AGT |
| VGEVLNSIFFELEADER | IPI00032220, IPI00908365 | Angiotensinogen | AGT |
| ADSQAQLLLSTVVGVFTAPGLHLK | IPI00032220 | Angiotensinogen precursor | AGT |
| LDAHKVLSALQAVQGLLVAQGR | IPI00032220 | Angiotensinogen precursor | AGT |
| LQAILGVPWK | IPI00032220 | Angiotensinogen precursor | AGT |
| QPFVQGLALYTPVVLPR | IPI00032220 | Angiotensinogen precursor | AGT |
| SLDFTELDVAAEK | IPI00032220 | Angiotensinogen precursor | AGT |
| SLDFTELDVAAEKIDR | IPI00032220 | Angiotensinogen precursor | AGT |
| VLSALQAVQGLLVAQGR | IPI00032220 | Angiotensinogen precursor | AGT |
| EHAVEGDCDFQLLK | IPI00922262, IPI00926249, IPI00795830, IPI00022431 | Alpha-2-HS-glycoprotein | AHSG |
| HTLNQIDEVK | IPI00922262, IPI00926249, IPI00795830, IPI00022431 | Alpha-2-HS-glycoprotein | AHSG |
| QPNCDDPETEEAALVAIDYINQNLPWGYK | IPI00922262, IPI00926249, IPI00795830, IPI00022431 | Alpha-2-HS-glycoprotein | AHSG |
| TVVQPSVGAAAGPVVPPCPGR | IPI00922262, IPI00926249, IPI00795830, IPI00022431 | Alpha-2-HS-glycoprotein | AHSG |
| VWPQQPSGELFEIEIDTLETTCHVLDPTPVAR | IPI00922262, IPI00926249, IPI00795830, IPI00022431 | Alpha-2-HS-glycoprotein | AHSG |
| GQLVPLETVLDMLR | IPI00018342, IPI00640817 | Adenylate kinase isoenzyme 1 | AK1 |
| AAFTECCQAADK | IPI00908876, IPI00384697, IPI00878517, IPI00022434, IPI00216773, IPI00878282, IPI00745872 | Serum albumin precursor | ALB |
| ADDKETCFAEEGK | IPI00908876, IPI00384697, IPI00878517, IPI00022434, IPI00216773, IPI00878282, IPI00745872 | Serum albumin precursor | ALB |
| ADDKETCFAEEGKK | IPI00908876, IPI00384697, IPI00878517, IPI00022434, IPI00216773, IPI00878282, IPI00745872 | Serum albumin precursor | ALB |
| AEFAEVSK | IPI00908876, IPI00384697, IPI00878517, IPI00022434, IPI00216773, IPI00878282, IPI00745872 | Serum albumin precursor | ALB |
| AFKAWAVAR | IPI00908876, IPI00384697, IPI00878517, IPI00022434, IPI00216773, IPI00878282, IPI00745872 | Serum albumin precursor | ALB |
| ALVLIAFAQYLQQCPFEDHVK | IPI00908876, IPI00384697, IPI00878517, IPI00022434, IPI00216773, IPI00878282, IPI00745872 | Serum albumin precursor | ALB |
| AVMDDFAAFVEK | IPI00908876, IPI00384697, IPI00878517, IPI00022434, IPI00216773, IPI00878282, IPI00745872 | Serum albumin precursor | ALB |
| CCAAADPHECYAK | IPI00908876, IPI00384697, IPI00878517, IPI00022434, IPI00216773, IPI00878282, IPI00745872 | Serum albumin precursor | ALB |
| CCTESLVNR | IPI00908876, IPI00384697, IPI00878517, IPI00022434, IPI00216773, IPI00878282, IPI00745872 | Serum albumin precursor | ALB |
| DDNPNLPR | IPI00908876, IPI00384697, IPI00878517, IPI00022434, IPI00216773, IPI00878282, IPI00745872 | Serum albumin precursor | ALB |
| DLGEENFK | IPI00908876, IPI00384697, IPI00878517, IPI00022434, IPI00216773, IPI00878282, IPI00745872 | Serum albumin precursor | ALB |
| DVFLGMFLYEYAR | IPI00908876, IPI00384697, IPI00878517, IPI00022434, IPI00216773, IPI00878282, IPI00745872 | Serum albumin precursor | ALB |
| ECCEKPLLEK | IPI00908876, IPI00384697, IPI00878517, IPI00022434, IPI00216773, IPI00878282, IPI00745872 | Serum albumin precursor | ALB |
| EFNAETFTFHADICTLSEK | IPI00908876, IPI00384697, IPI00878517, IPI00022434, IPI00216773, IPI00878282, IPI00745872 | Serum albumin precursor | ALB |
| ETYGEMADCCAK | IPI00908876, IPI00384697, IPI00878517, IPI00022434, IPI00216773, IPI00878282, IPI00745872 | Serum albumin precursor | ALB |
| FKDLGEENFK | IPI00908876, IPI00384697, IPI00878517, IPI00022434, IPI00216773, IPI00878282, IPI00745872 | Serum albumin precursor | ALB |
| FQNALLVR | IPI00908876, IPI00384697, IPI00878517, IPI00022434, IPI00216773, IPI00878282, IPI00745872 | Serum albumin precursor | ALB |
| HPDYSVVLLLR | IPI00908876, IPI00384697, IPI00878517, IPI00022434, IPI00216773, IPI00878282, IPI00745872 | Serum albumin precursor | ALB |
| HPYFYAPELLFFAK | IPI00908876, IPI00384697, IPI00878517, IPI00022434, IPI00216773, IPI00878282, IPI00745872 | Serum albumin precursor | ALB |
| KLVAASQAALGL | IPI00908876, IPI00384697, IPI00878517, IPI00022434, IPI00216773, IPI00878282, IPI00745872 | Serum albumin precursor | ALB |
| KQTALVELVK | IPI00908876, IPI00384697, IPI00878517, IPI00022434, IPI00216773, IPI00878282, IPI00745872 | Serum albumin precursor | ALB |
| KVPQVSTPTLVEVSR | IPI00908876, IPI00384697, IPI00878517, IPI00022434, IPI00216773, IPI00878282, IPI00745872 | Serum albumin precursor | ALB |
| KYLYEIAR | IPI00908876, IPI00384697, IPI00878517, IPI00022434, IPI00216773, IPI00878282, IPI00745872 | Serum albumin precursor | ALB |
| LCTVATLR | IPI00908876, IPI00384697, IPI00878517, IPI00022434, IPI00216773, IPI00878282, IPI00745872 | Serum albumin precursor | ALB |
| LDELRDEGK | IPI00908876, IPI00384697, IPI00878517, IPI00022434, IPI00216773, IPI00878282, IPI00745872 | Serum albumin precursor | ALB |
| LKECCEKPLLEK | IPI00908876, IPI00384697, IPI00878517, IPI00022434, IPI00216773, IPI00878282, IPI00745872 | Serum albumin precursor | ALB |
| LVNEVTEFAK | IPI00908876, IPI00384697, IPI00878517, IPI00022434, IPI00216773, IPI00878282, IPI00745872 | Serum albumin precursor | ALB |
| LVRPEVDVMCTAFHDNEETFLK | IPI00908876, IPI00384697, IPI00878517, IPI00022434, IPI00216773, IPI00878282, IPI00745872 | Serum albumin precursor | ALB |
| LVRPEVDVMCTAFHDNEETFLKK | IPI00908876, IPI00384697, IPI00878517, IPI00022434, IPI00216773, IPI00878282, IPI00745872 | Serum albumin precursor | ALB |
| MPCAEDYLSVVLNQLCVLHEK | IPI00908876, IPI00384697, IPI00878517, IPI00022434, IPI00216773, IPI00878282, IPI00745872 | Serum albumin precursor | ALB |
| NECFLQHK | IPI00908876, IPI00384697, IPI00878517, IPI00022434, IPI00216773, IPI00878282, IPI00745872 | Serum albumin precursor | ALB |
| NECFLQHKDDNPNLPR | IPI00908876, IPI00384697, IPI00878517, IPI00022434, IPI00216773, IPI00878282, IPI00745872 | Serum albumin precursor | ALB |
| QEPERNECFLQHK | IPI00908876, IPI00384697, IPI00878517, IPI00022434, IPI00216773, IPI00878282, IPI00745872 | Serum albumin precursor | ALB |
| QEPERNECFLQHKDDNPNLPR | IPI00908876, IPI00384697, IPI00878517, IPI00022434, IPI00216773, IPI00878282, IPI00745872 | Serum albumin precursor | ALB |
| QNCELFEQLGEYK | IPI00908876, IPI00384697, IPI00878517, IPI00022434, IPI00216773, IPI00878282, IPI00745872 | Serum albumin precursor | ALB |
| QTALVELVK | IPI00908876, IPI00384697, IPI00878517, IPI00022434, IPI00216773, IPI00878282, IPI00745872 | Serum albumin precursor | ALB |
| RHPDYSVVLLLR | IPI00908876, IPI00384697, IPI00878517, IPI00022434, IPI00216773, IPI00878282, IPI00745872 | Serum albumin precursor | ALB |
| RHPYFYAPELLFFAK | IPI00908876, IPI00384697, IPI00878517, IPI00022434, IPI00216773, IPI00878282, IPI00745872 | Serum albumin precursor | ALB |
| RMPCAEDYLSVVLNQLCVLHEK | IPI00908876, IPI00384697, IPI00878517, IPI00022434, IPI00216773, IPI00878282, IPI00745872 | Serum albumin precursor | ALB |
| RPCFSALEVDETYVPK | IPI00908876, IPI00384697, IPI00878517, IPI00022434, IPI00216773, IPI00878282, IPI00745872 | Serum albumin precursor | ALB |
| SHCIAEVENDEMPADLPSLAADFVESK | IPI00908876, IPI00384697, IPI00878517, IPI00022434, IPI00216773, IPI00878282, IPI00745872 | Serum albumin precursor | ALB |
| SLHTLFGDK | IPI00908876, IPI00384697, IPI00878517, IPI00022434, IPI00216773, IPI00878282, IPI00745872 | Serum albumin precursor | ALB |
| TCVADESAENCDK | IPI00908876, IPI00384697, IPI00878517, IPI00022434, IPI00216773, IPI00878282, IPI00745872 | Serum albumin precursor | ALB |
| TYETTLEK | IPI00908876, IPI00384697, IPI00878517, IPI00022434, IPI00216773, IPI00878282, IPI00745872 | Serum albumin precursor | ALB |
| VFDEFKPLVEEPQNLIK | IPI00908876, IPI00384697, IPI00878517, IPI00022434, IPI00216773, IPI00878282, IPI00745872 | Serum albumin precursor | ALB |
| VHTECCHGDLLECADDR | IPI00908876, IPI00384697, IPI00878517, IPI00022434, IPI00216773, IPI00878282, IPI00745872 | Serum albumin precursor | ALB |
| VPQVSTPTLVEVSR | IPI00908876, IPI00384697, IPI00878517, IPI00022434, IPI00216773, IPI00878282, IPI00745872 | Serum albumin precursor | ALB |
| YICENQDSISSK | IPI00908876, IPI00384697, IPI00878517, IPI00022434, IPI00216773, IPI00878282, IPI00745872 | Serum albumin precursor | ALB |
| CLGNGNPPPEEFLFYLPGQPEGIR | IPI00807403, IPI00015102, IPI00795504, IPI00748835 | Isoform 1 of CD166 antigen | ALCAM |
| QIGDALPVSCTISASR | IPI00807403, IPI00015102, IPI00795504, IPI00748835 | Isoform 1 of CD166 antigen | ALCAM |
| SMIASTAITVHYLDLSLNPSGEVTR | IPI00807403, IPI00015102, IPI00795504, IPI00748835 | Isoform 1 of CD166 antigen | ALCAM |
| VLHPLEGAVVIIFK | IPI00807403, IPI00015102, IPI00795504, IPI00748835 | Isoform 1 of CD166 antigen | ALCAM |
| QAFQIGSPWR | IPI00910463, IPI00644628, IPI00218914, IPI00644077, IPI00642144 | Retinal dehydrogenase 1 | ALDH1A1 |
| ADDGRPFPQVIK | IPI00465439, IPI00796333 | Fructose-bisphosphate aldolase A | ALDOA |
| FSHEEIAMATVTALR | IPI00465439, IPI00796333 | Fructose-bisphosphate aldolase A | ALDOA |
| GILAADESTGSIAKR | IPI00465439, IPI00796333 | Fructose-bisphosphate aldolase A | ALDOA |
| GVVPLAGTNGETTTQGLDGLSER | IPI00465439, IPI00796333 | Fructose-bisphosphate aldolase A | ALDOA |
| IGEHTPSALAIMENANVLAR | IPI00465439, IPI00796333 | Fructose-bisphosphate aldolase A | ALDOA |
| QLLLTADDR | IPI00465439, IPI00796333 | Fructose-bisphosphate aldolase A | ALDOA |
| ALQASALAAWGGK | IPI00942961, IPI00218407, IPI00513830 | Fructose-bisphosphate aldolase B | ALDOB |
| ETTIQGLDGLSER | IPI00942961, IPI00218407, IPI00513830 | Fructose-bisphosphate aldolase B | ALDOB |
| GILAADESVGTMGNR | IPI00218407 | Fructose-bisphosphate aldolase B | ALDOB |
| IADQCPSSLAIQENANALAR | IPI00942961, IPI00218407, IPI00513830 | Fructose-bisphosphate aldolase B | ALDOB |
| KYTPEQVAMATVTALHR | IPI00942961, IPI00218407, IPI00513830 | Fructose-bisphosphate aldolase B | ALDOB |
| YTPEQVAMATVTALHR | IPI00942961, IPI00218407, IPI00513830 | Fructose-bisphosphate aldolase B | ALDOB |
| GVVPLAGTDGETTTQGLDGLSER | IPI00792375, IPI00789171, IPI00418262 | Fructose-bisphosphate aldolase | ALDOC |
| GFFLLVEGGR | IPI00419916 | Alkaline phosphatase, tissue-nonspecific isozyme precursor | ALPL |
| AFIQLWAFDAVK | IPI00022426 | AMBP protein precursor | AMBP |
| CVLFPYGGCQGNGNK | IPI00022426 | AMBP protein precursor | AMBP |
| EYCGVPGDGDEELLR | IPI00022426 | AMBP protein precursor | AMBP |
| TVAACNLPIVR | IPI00022426 | AMBP protein precursor | AMBP |
| WYNLAIGSTCPWLK | IPI00022426 | AMBP protein precursor | AMBP |
| EDSCQLGYSAGPCMGMTSR | IPI00022426, IPI00922298 | Protein AMBP | AMBP |
| ETLLQDFR | IPI00022426, IPI00922298 | Protein AMBP | AMBP |
| GECVPGEQEPEPILIPR | IPI00022426, IPI00922298 | Protein AMBP | AMBP |
| GVCEETSGAYEK | IPI00022426, IPI00922298 | Protein AMBP | AMBP |
| KEDSCQLGYSAGPCMGMTSR | IPI00022426, IPI00922298 | Protein AMBP | AMBP |
| KGVCEETSGAYEK | IPI00022426, IPI00922298 | Protein AMBP | AMBP |
| MTVSTLVLGEGATEAEISMTSTR | IPI00022426, IPI00922298 | Protein AMBP | AMBP |
| VVAQGVGIPEDSIFTMADR | IPI00022426, IPI00922298 | Protein AMBP | AMBP |
| IAEYMNHLIDIGVAGFR | IPI00640335, IPI00644068, IPI00300786, IPI00646265, IPI00923515, IPI00939512, IPI00641425, IPI00021447, IPI00645326, IPI00025476 | Alpha-amylase 2B | AMY2B |
| LSGLLDLALGK | IPI00640335, IPI00644068, IPI00300786, IPI00646265, IPI00923515, IPI00939512, IPI00641425, IPI00021447, IPI00645326, IPI00025476 | Alpha-amylase 2B | AMY2B |
| TGSGDIENYNDATQVR | IPI00640335, IPI00644068, IPI00300786, IPI00646265, IPI00923515, IPI00939512, IPI00641425, IPI00021447, IPI00645326, IPI00025476 | Alpha-amylase 2B | AMY2B |
| WVDIALECER | IPI00640335, IPI00644068, IPI00300786, IPI00646265, IPI00923515, IPI00939512, IPI00641425, IPI00021447, IPI00645326, IPI00025476 | Alpha-amylase 2B | AMY2B |
| DINTFIHGNK | IPI00008554 | Angiogenin precursor | ANG |
| LHGGSPWPPCQYR | IPI00008554 | Angiogenin precursor | ANG |
| NVVVACENGLPVHLDQSIFR | IPI00008554 | Angiogenin precursor | ANG |
| SSFQVTTCK | IPI00008554 | Angiogenin precursor | ANG |
| YCESIMR | IPI00008554 | Angiogenin precursor | ANG |
| GQINDIFQK | IPI00004957 | Angiopoietin-related protein 3 precursor | ANGPTL3 |
| LDGEFWLGLEK | IPI00004957 | Angiopoietin-related protein 3 precursor | ANGPTL3 |
| DLMVLNDVYR | IPI00221224 | Aminopeptidase N | ANPEP |
| DLTALSNMLPK | IPI00221224 | Aminopeptidase N | ANPEP |
| DSQYEMDSEFEGELADDLAGFYR | IPI00221224 | Aminopeptidase N | ANPEP |
| ELWILNR | IPI00221224 | Aminopeptidase N | ANPEP |
| ENSLLFDPLSSSSSNK | IPI00221224 | Aminopeptidase N | ANPEP |
| EVVLQWFTENSK | IPI00221224 | Aminopeptidase N | ANPEP |
| FSTEYELQQLEQFK | IPI00221224 | Aminopeptidase N | ANPEP |
| FYISK | IPI00221224 | Aminopeptidase N | ANPEP |
| GLYVFK | IPI00221224 | Aminopeptidase N | ANPEP |
| KQVTPLFIHFR | IPI00221224 | Aminopeptidase N | ANPEP |
| KVVATTQMQAADAR | IPI00221224 | Aminopeptidase N | ANPEP |
| LFNDYGGGSFSFSNLIQAVTR | IPI00221224 | Aminopeptidase N | ANPEP |
| QQQDYWLIDVR | IPI00221224 | Aminopeptidase N | ANPEP |
| QVTPLFIHFR | IPI00221224 | Aminopeptidase N | ANPEP |
| QWMENPNNNPIHPNLR | IPI00221224 | Aminopeptidase N | ANPEP |
| QYMPWEAALSSLSYFK | IPI00221224 | Aminopeptidase N | ANPEP |
| SDQIGLPDFNAGAMENWGLVTYR | IPI00221224 | Aminopeptidase N | ANPEP |
| SIQLPTTVR | IPI00221224 | Aminopeptidase N | ANPEP |
| STVYCNAIAQGGEEEWDFAWEQFR | IPI00221224 | Aminopeptidase N | ANPEP |
| VVATTQMQAADAR | IPI00221224 | Aminopeptidase N | ANPEP |
| YLSYTLNPDLIR | IPI00221224 | Aminopeptidase N | ANPEP |
| DAFCVFEQNQGLPLR | IPI00004457 | Membrane copper amine oxidase | AOC3 |
| EALAIVFFGR | IPI00004457 | Membrane copper amine oxidase | AOC3 |
| EELTAVMR | IPI00024357, IPI00293889, IPI00004457 | Membrane copper amine oxidase | AOC3 |
| LLEMEEQAAFLVGSATPR | IPI00004457 | Membrane copper amine oxidase | AOC3 |
| RPVLFQEYLDIDQMIFNR | IPI00004457 | Membrane copper amine oxidase | AOC3 |
| SPVPPGPAPPLQFYPQGPR | IPI00004457 | Membrane copper amine oxidase | AOC3 |
| AYSLFSYNTQGR | IPI00022391 | Serum amyloid P-component precursor | APCS |
| GYVIIKPLVWV | IPI00022391 | Serum amyloid P-component precursor | APCS |
| IVLGQEQDSYGGK | IPI00022391 | Serum amyloid P-component precursor | APCS |
| IVLGQEQDSYGGKFDR | IPI00022391 | Serum amyloid P-component precursor | APCS |
| QGYFVEAQPK | IPI00022391 | Serum amyloid P-component precursor | APCS |
| VGEYSLYIGR | IPI00022391 | Serum amyloid P-component precursor | APCS |
| AKPALEDLR | IPI00853525, IPI00021841 | Apolipoprotein A-I precursor | APOA1 |
| ATEHLSTLSEK | IPI00853525, IPI00021841 | Apolipoprotein A-I precursor | APOA1 |
| DLATVYVDVLK | IPI00021841 | Apolipoprotein A-I precursor | APOA1 |
| DYVSQFEGSALGK | IPI00021841 | Apolipoprotein A-I precursor | APOA1 |
| EQLGPVTQEFWDNLEK | IPI00853525, IPI00021841 | Apolipoprotein A-I precursor | APOA1 |
| EQLGPVTQEFWDNLEKETEGLR | IPI00853525, IPI00021841 | Apolipoprotein A-I precursor | APOA1 |
| EQLGPVTQEFWDNLEKETEGLRQEMSK | IPI00853525, IPI00021841 | Apolipoprotein A-I precursor | APOA1 |
| HFWQQDEPPQSPWDR | IPI00021841 | Apolipoprotein A-I precursor | APOA1 |
| KWQEEMELYR | IPI00853525, IPI00021841 | Apolipoprotein A-I precursor | APOA1 |
| LEALKENGGAR | IPI00853525, IPI00021841 | Apolipoprotein A-I precursor | APOA1 |
| LLDNWDSVTSTFSK | IPI00853525, IPI00021841 | Apolipoprotein A-I precursor | APOA1 |
| LSPLGEEMR | IPI00853525, IPI00021841 | Apolipoprotein A-I precursor | APOA1 |
| QGLLPVLESFK | IPI00853525, IPI00021841 | Apolipoprotein A-I precursor | APOA1 |
| QKVEPLRAELQEGAR | IPI00853525, IPI00021841 | Apolipoprotein A-I precursor | APOA1 |
| THLAPYSDELR | IPI00853525, IPI00021841 | Apolipoprotein A-I precursor | APOA1 |
| VEPLRAELQEGAR | IPI00853525, IPI00021841 | Apolipoprotein A-I precursor | APOA1 |
| VKDLATVYVDVLK | IPI00021841 | Apolipoprotein A-I precursor | APOA1 |
| VQPYLDDFQK | IPI00853525, IPI00021841 | Apolipoprotein A-I precursor | APOA1 |
| VSFLSALEEYTK | IPI00853525, IPI00021841 | Apolipoprotein A-I precursor | APOA1 |
| WQEEMELYR | IPI00853525, IPI00021841 | Apolipoprotein A-I precursor | APOA1 |
| EPCVESLVSQYFQTVTDYGK | IPI00021854 | Apolipoprotein A-II precursor | APOA2 |
| EQLTPLIK | IPI00021854 | Apolipoprotein A-II precursor | APOA2 |
| KAGTELVNFLSYFVELGTQPATQ | IPI00021854 | Apolipoprotein A-II precursor | APOA2 |
| SKEQLTPLIK | IPI00021854 | Apolipoprotein A-II precursor | APOA2 |
| SPELQAEAK | IPI00021854 | Apolipoprotein A-II precursor | APOA2 |
| VKSPELQAEAK | IPI00021854 | Apolipoprotein A-II precursor | APOA2 |
| AEVSADQVATVMWDYFSQLSNNAK | IPI00847179, IPI00304273 | Apolipoprotein A-IV precursor | APOA4 |
| ALVQQMEQLR | IPI00847179, IPI00304273 | Apolipoprotein A-IV precursor | APOA4 |
| DKVNSFFSTFK | IPI00847179, IPI00304273 | Apolipoprotein A-IV precursor | APOA4 |
| EAVEHLQK | IPI00847179, IPI00304273 | Apolipoprotein A-IV precursor | APOA4 |
| ENADSLQASLRPHADELK | IPI00847179, IPI00304273 | Apolipoprotein A-IV precursor | APOA4 |
| GNTEGLQK | IPI00847179, IPI00304273 | Apolipoprotein A-IV precursor | APOA4 |
| IDQNVEELK | IPI00847179, IPI00304273 | Apolipoprotein A-IV precursor | APOA4 |
| IDQTVEELR | IPI00847179, IPI00304273 | Apolipoprotein A-IV precursor | APOA4 |
| IDQTVEELRR | IPI00847179, IPI00304273 | Apolipoprotein A-IV precursor | APOA4 |
| ISASAEELR | IPI00847179, IPI00304273 | Apolipoprotein A-IV precursor | APOA4 |
| KLVPFATELHER | IPI00847179, IPI00304273 | Apolipoprotein A-IV precursor | APOA4 |
| LAPLAEDVR | IPI00847179, IPI00304273 | Apolipoprotein A-IV precursor | APOA4 |
| LEPYADQLR | IPI00847179, IPI00304273 | Apolipoprotein A-IV precursor | APOA4 |
| LGEVNTYAGDLQK | IPI00847179, IPI00304273 | Apolipoprotein A-IV precursor | APOA4 |
| LKEEIGKELEELR | IPI00847179, IPI00304273 | Apolipoprotein A-IV precursor | APOA4 |
| LLPHANEVSQK | IPI00847179, IPI00304273 | Apolipoprotein A-IV precursor | APOA4 |
| LNHQLEGLTFQMK | IPI00847179, IPI00304273 | Apolipoprotein A-IV precursor | APOA4 |
| LTPYADEFK | IPI00847179, IPI00304273 | Apolipoprotein A-IV precursor | APOA4 |
| LVPFATELHER | IPI00847179, IPI00304273 | Apolipoprotein A-IV precursor | APOA4 |
| QLTPYAQR | IPI00847179, IPI00304273 | Apolipoprotein A-IV precursor | APOA4 |
| RVEPYGENFNK | IPI00847179, IPI00304273 | Apolipoprotein A-IV precursor | APOA4 |
| SELTQQLNALFQDK | IPI00847179, IPI00304273 | Apolipoprotein A-IV precursor | APOA4 |
| SLAELGGHLDQQVEEFR | IPI00847179, IPI00304273 | Apolipoprotein A-IV precursor | APOA4 |
| SLAELGGHLDQQVEEFRR | IPI00847179, IPI00304273 | Apolipoprotein A-IV precursor | APOA4 |
| SLAPYAQDTQEK | IPI00847179, IPI00304273 | Apolipoprotein A-IV precursor | APOA4 |
| TQVNTQAEQLR | IPI00304273 | Apolipoprotein A-IV precursor | APOA4 |
| TQVSTQAEQLR | IPI00847179 | apolipoprotein A-IV precursor | APOA4 |
| VEPYGENFNK | IPI00847179, IPI00304273 | Apolipoprotein A-IV precursor | APOA4 |
| VKIDQTVEELR | IPI00847179, IPI00304273 | Apolipoprotein A-IV precursor | APOA4 |
| VNSFFSTFK | IPI00847179, IPI00304273 | Apolipoprotein A-IV precursor | APOA4 |
| VNSFFSTFKEK | IPI00847179, IPI00304273 | Apolipoprotein A-IV precursor | APOA4 |
| DKDQEVLLQTFLDDASPGDKR | IPI00022229, IPI00894122 | Apolipoprotein B-100 | APOB |
| GFEPTLEALFGK | IPI00022229, IPI00894122 | Apolipoprotein B-100 | APOB |
| ILGEELGFASLHDLQLLGK | IPI00022229, IPI00894122 | Apolipoprotein B-100 | APOB |
| IVQILPWEQNEQVK | IPI00022229, IPI00894122 | Apolipoprotein B-100 | APOB |
| KGNVATEISTER | IPI00022229, IPI00894122 | Apolipoprotein B-100 | APOB |
| KYTYNYEAESSSGVPGTADSR | IPI00022229, IPI00894122 | Apolipoprotein B-100 | APOB |
| LAAYLMLMR | IPI00022229, IPI00894122 | Apolipoprotein B-100 | APOB |
| NFVASHIANILNSEELDIQDLK | IPI00022229, IPI00894122 | Apolipoprotein B-100 | APOB |
| TGISPLALIK | IPI00022229, IPI00894122 | Apolipoprotein B-100 | APOB |
| TTLTAFGFASADLIEIGLEGK | IPI00022229, IPI00894122 | Apolipoprotein B-100 | APOB |
| VLVDHFGYTK | IPI00022229, IPI00894122 | Apolipoprotein B-100 | APOB |
| ATFQTPDFIVPLTDLR | IPI00022229 | Apolipoprotein B-100 precursor | APOB |
| AVSMPSFSILGSDVR | IPI00022229 | Apolipoprotein B-100 precursor | APOB |
| DAVEKPQEFTIVAFVK | IPI00022229 | Apolipoprotein B-100 precursor | APOB |
| DFSLWEK | IPI00022229 | Apolipoprotein B-100 precursor | APOB |
| DLKVEDIPLAR | IPI00022229 | Apolipoprotein B-100 precursor | APOB |
| EFNLQNMGLPDFHIPENLFLK | IPI00022229 | Apolipoprotein B-100 precursor | APOB |
| EFQVPTFTIPK | IPI00022229 | Apolipoprotein B-100 precursor | APOB |
| FSVPAGIVIPSFQALTAR | IPI00022229 | Apolipoprotein B-100 precursor | APOB |
| HFVINLIGDFEVAEK | IPI00022229 | Apolipoprotein B-100 precursor | APOB |
| HINIDQFVR | IPI00022229 | Apolipoprotein B-100 precursor | APOB |
| HLIDSLIDFLNFPR | IPI00022229 | Apolipoprotein B-100 precursor | APOB |
| HSITNPLAVLCEFISQSIK | IPI00022229 | Apolipoprotein B-100 precursor | APOB |
| IADFELPTIIVPEQTIEIPSIK | IPI00022229 | Apolipoprotein B-100 precursor | APOB |
| IDDIWNLEVK | IPI00022229 | Apolipoprotein B-100 precursor | APOB |
| IEFEWNTGTNVDTK | IPI00022229 | Apolipoprotein B-100 precursor | APOB |
| IEIPLPFGGK | IPI00022229 | Apolipoprotein B-100 precursor | APOB |
| ITLPDFR | IPI00022229 | Apolipoprotein B-100 precursor | APOB |
| IYSLWEHSTK | IPI00022229 | Apolipoprotein B-100 precursor | APOB |
| LELELRPTGEIEQYSVSATYELQR | IPI00022229 | Apolipoprotein B-100 precursor | APOB |
| LIDVISMYR | IPI00022229 | Apolipoprotein B-100 precursor | APOB |
| LIVAMSSWLQK | IPI00022229 | Apolipoprotein B-100 precursor | APOB |
| LNDLNSVLVMPTFHVPFTDLQVPSCK | IPI00022229 | Apolipoprotein B-100 precursor | APOB |
| LPQQANDYLNSFNWER | IPI00022229 | Apolipoprotein B-100 precursor | APOB |
| LPYTIITTPPLKDFSLWEK | IPI00022229 | Apolipoprotein B-100 precursor | APOB |
| LSNDMMGSYAEMK | IPI00022229 | Apolipoprotein B-100 precursor | APOB |
| LVGFIDDAVK | IPI00022229 | Apolipoprotein B-100 precursor | APOB |
| NIFNFK | IPI00022229 | Apolipoprotein B-100 precursor | APOB |
| NLQNNAEWVYQGAIR | IPI00022229 | Apolipoprotein B-100 precursor | APOB |
| NNALDFVTK | IPI00022229 | Apolipoprotein B-100 precursor | APOB |
| NQDVHSINLPFFETLQEYFER | IPI00022229 | Apolipoprotein B-100 precursor | APOB |
| SLWDFLK | IPI00022229 | Apolipoprotein B-100 precursor | APOB |
| TEVIPPLIENR | IPI00022229 | Apolipoprotein B-100 precursor | APOB |
| TIHDLHLFIENIDFNK | IPI00022229 | Apolipoprotein B-100 precursor | APOB |
| TLQGIPQMIGEVIR | IPI00022229 | Apolipoprotein B-100 precursor | APOB |
| TQFNNNEYSQDLDAYNTK | IPI00022229 | Apolipoprotein B-100 precursor | APOB |
| VLLDQLGTTISFER | IPI00022229 | Apolipoprotein B-100 precursor | APOB |
| VNWEEEAASGLLTSLK | IPI00022229 | Apolipoprotein B-100 precursor | APOB |
| VPSYTLILPSLELPVLHVPR | IPI00022229 | Apolipoprotein B-100 precursor | APOB |
| EFGNTLEDK | IPI00021855 | Apolipoprotein C-I precursor | APOC1 |
| EWFSETFQK | IPI00021855 | Apolipoprotein C-I precursor | APOC1 |
| QSELSAK | IPI00021855 | Apolipoprotein C-I precursor | APOC1 |
| ESLSSYWESAK | IPI00021856 | Apolipoprotein C-II precursor | APOC2 |
| STAAMSTYTGIFTDQVLSVLK | IPI00021856 | Apolipoprotein C-II precursor | APOC2 |
| TAAQNLYEK | IPI00021856 | Apolipoprotein C-II precursor | APOC2 |
| TYLPAVDEK | IPI00021856 | Apolipoprotein C-II precursor | APOC2 |
| DALSSVQESQVAQQAR | IPI00657670, IPI00021857 | Apolipoprotein C-III precursor | APOC3 |
| GWVTDGFSSLK | IPI00657670, IPI00021857 | Apolipoprotein C-III precursor | APOC3 |
| AWFLESK | IPI00022731 | Apolipoprotein C-IV precursor | APOC4 |
| DGWQWFWSPSTFR | IPI00022731 | Apolipoprotein C-IV precursor | APOC4 |
| GFMQTYYDDHLR | IPI00022731 | Apolipoprotein C-IV precursor | APOC4 |
| MKELLETVVNR | IPI00022731 | Apolipoprotein C-IV precursor | APOC4 |
| CPNPPVQENFDVNK | IPI00006662, IPI00924574, IPI00927914, IPI00910432, IPI00927707 | Apolipoprotein D | APOD |
| IPTTFENGR | IPI00006662, IPI00924574, IPI00927914, IPI00910432, IPI00927707 | Apolipoprotein D | APOD |
| MTVTDQVNCPK | IPI00006662, IPI00924574, IPI00927914, IPI00910432, IPI00927707 | Apolipoprotein D | APOD |
| NILTSNNIDVK | IPI00006662, IPI00924574, IPI00927914, IPI00910432, IPI00927707 | Apolipoprotein D | APOD |
| AATVGSLAGQPLQER | IPI00879456, IPI00878953, IPI00879368, IPI00021842 | Apolipoprotein E | APOE |
| AYKSELEEQLTPVAEETR | IPI00879456, IPI00878953, IPI00879368, IPI00021842 | Apolipoprotein E | APOE |
| GEVQAMLGQSTEELR | IPI00879456, IPI00878953, IPI00879368, IPI00021842 | Apolipoprotein E | APOE |
| LAVYQAGAR | IPI00879456, IPI00878953, IPI00879368, IPI00021842 | Apolipoprotein E | APOE |
| LGADMEDVCGR | IPI00879456, IPI00878953, IPI00879368, IPI00021842 | Apolipoprotein E | APOE |
| LGPLVEQGR | IPI00879456, IPI00878953, IPI00879368, IPI00021842 | Apolipoprotein E | APOE |
| SELEEQLTPVAEETR | IPI00879456, IPI00878953, IPI00879368, IPI00021842 | Apolipoprotein E | APOE |
| WVQTLSEQVQEELLSSQVTQELR | IPI00879456, IPI00878953, IPI00879368, IPI00021842 | Apolipoprotein E | APOE |
| LEEQAQQIR | IPI00021842 | Apolipoprotein E precursor | APOE |
| LQAEAFQAR | IPI00021842 | Apolipoprotein E precursor | APOE |
| QWAGLVEK | IPI00021842 | Apolipoprotein E precursor | APOE |
| SWFEPLVEDMQR | IPI00021842 | Apolipoprotein E precursor | APOE |
| FLVSLALR | IPI00299435 | apolipoprotein F precursor | APOF |
| SGVQQLIQYYQDQK | IPI00299435 | apolipoprotein F precursor | APOF |
| EFLGENISNFLSLAGNTYQLTR | IPI00914948, IPI00514475, IPI00186903 | Isoform 2 of Apolipoprotein L1 | APOL1 |
| LNILNNNYK | IPI00914948, IPI00514475, IPI00186903 | Isoform 2 of Apolipoprotein L1 | APOL1 |
| VAQELEEK | IPI00914948, IPI00514475, IPI00186903 | Isoform 2 of Apolipoprotein L1 | APOL1 |
| VTEPISAESGEQVER | IPI00914948, IPI00514475, IPI00186903 | Isoform 2 of Apolipoprotein L1 | APOL1 |
| TSQGTSFTFGGLNQAR | IPI00013682, IPI00909926, IPI00220292, IPI00220293 | Isoform 3 of Ecto-ADP-ribosyltransferase 3 | ART3 |
| GFPLITITVR | IPI00477831, IPI00165949 | Isoform 2 of Adipocyte-derived leucine aminopeptidase precursor | ARTS-1 |
| WNDDFCLQVYR | IPI00941065, IPI00011155, IPI00796731, IPI00877085, IPI00797460, IPI00220266, IPI00150200 | HBxAg-binding protein | ASGR2 |
| ITLHVPEHLIADGSR | IPI00647130, IPI00022367, IPI00514985, IPI00478895 | Astrotactin 1 | ASTN |
| CFSSDFMAYDIACDR | IPI00027235, IPI00939169, IPI00218460, IPI00162735, IPI00940485 | Isoform 1 of Attractin | ATRN |
| CNPGTGQCVCPAGWVGEQCQHCGGR | IPI00027235, IPI00939169, IPI00218460, IPI00162735, IPI00940485 | Isoform 1 of Attractin | ATRN |
| CTWLIEGQPNR | IPI00027235, IPI00939169, IPI00218460, IPI00162735, IPI00940485 | Isoform 1 of Attractin | ATRN |
| CVWNTGSSQCISWALATDEQEEK | IPI00027235, IPI00939169, IPI00218460, IPI00162735, IPI00940485 | Isoform 1 of Attractin | ATRN |
| DLDMFINASK | IPI00027235, IPI00939169, IPI00218460, IPI00162735, IPI00940485 | Isoform 1 of Attractin | ATRN |
| DNPMYYCNK | IPI00027235, IPI00939169, IPI00218460, IPI00162735, IPI00940485 | Isoform 1 of Attractin | ATRN |
| FRLTGSSGFVTDGPGNYK | IPI00027235, IPI00939169, IPI00218460, IPI00162735, IPI00940485 | Isoform 1 of Attractin | ATRN |
| GDECQLCEVENR | IPI00027235, IPI00939169, IPI00218460, IPI00162735, IPI00940485 | Isoform 1 of Attractin | ATRN |
| LTGSSGFVTDGPGNYK | IPI00027235, IPI00939169, IPI00218460, IPI00162735, IPI00940485 | Isoform 1 of Attractin | ATRN |
| LTLTPWVGLR | IPI00027235, IPI00939169, IPI00218460, IPI00162735, IPI00940485 | Isoform 1 of Attractin | ATRN |
| NQECIALPENICGIGWHLVGNSCLK | IPI00027235, IPI00939169, IPI00218460, IPI00162735, IPI00940485 | Isoform 1 of Attractin | ATRN |
| SCALDQNCQWEPR | IPI00027235, IPI00939169, IPI00218460, IPI00162735, IPI00940485 | Isoform 1 of Attractin | ATRN |
| TACGDCTSGSSECMWCSNMK | IPI00027235, IPI00939169, IPI00218460, IPI00162735, IPI00940485 | Isoform 1 of Attractin | ATRN |
| TATITVLPQQPR | IPI00296992, IPI00397361 | AXL receptor tyrosine kinase isoform 1 | AXL |
| AGEVQEPELR | IPI00924948, IPI00871622, IPI00939278, IPI00816309, IPI00166729, IPI00924815 | alpha-2-glycoprotein 1, zinc | AZGP1 |
| AREDIFMETLK | IPI00924948, IPI00871622, IPI00939278, IPI00816309, IPI00166729, IPI00924815 | alpha-2-glycoprotein 1, zinc | AZGP1 |
| AYLEEECPATLR | IPI00924948, IPI00871622, IPI00939278, IPI00816309, IPI00166729, IPI00924815 | alpha-2-glycoprotein 1, zinc | AZGP1 |
| CLAYDFYPGK | IPI00924948, IPI00871622, IPI00939278, IPI00816309, IPI00166729, IPI00924815 | alpha-2-glycoprotein 1, zinc | AZGP1 |
| DIVEYYNDSNGSHVLQGR | IPI00924948, IPI00871622, IPI00939278, IPI00816309, IPI00166729, IPI00924815 | alpha-2-glycoprotein 1, zinc | AZGP1 |
| EDIFMETLK | IPI00924948, IPI00871622, IPI00939278, IPI00816309, IPI00166729, IPI00924815 | alpha-2-glycoprotein 1, zinc | AZGP1 |
| EIPAWVPFDPAAQITK | IPI00924948, IPI00871622, IPI00939278, IPI00816309, IPI00166729, IPI00924815 | alpha-2-glycoprotein 1, zinc | AZGP1 |
| HVEDVPAFQALGSLNDLQFFR | IPI00924948, IPI00871622, IPI00939278, IPI00816309, IPI00166729, IPI00924815 | alpha-2-glycoprotein 1, zinc | AZGP1 |
| KSQPMGLWR | IPI00924948, IPI00871622, IPI00939278, IPI00816309, IPI00166729, IPI00924815 | alpha-2-glycoprotein 1, zinc | AZGP1 |
| NILDRQDPPSVVVTSHQAPGEK | IPI00166729 | alpha-2-glycoprotein 1, zinc | AZGP1 |
| QDPPSVVVTSHQAPGEK | IPI00166729 | alpha-2-glycoprotein 1, zinc | AZGP1 |
| QKWEAEPVYVQR | IPI00924948, IPI00871622, IPI00939278, IPI00816309, IPI00166729, IPI00924815 | alpha-2-glycoprotein 1, zinc | AZGP1 |
| QVEGMEDWK | IPI00924948, IPI00871622, IPI00939278, IPI00816309, IPI00166729, IPI00924815 | alpha-2-glycoprotein 1, zinc | AZGP1 |
| QVEGMEDWKQDSQLQK | IPI00924948, IPI00871622, IPI00939278, IPI00816309, IPI00166729, IPI00924815 | alpha-2-glycoprotein 1, zinc | AZGP1 |
| SQPMGLWR | IPI00924948, IPI00871622, IPI00939278, IPI00816309, IPI00166729, IPI00924815 | alpha-2-glycoprotein 1, zinc | AZGP1 |
| WEAEPVYVQR | IPI00924948, IPI00871622, IPI00939278, IPI00816309, IPI00166729, IPI00924815 | alpha-2-glycoprotein 1, zinc | AZGP1 |
| YYYDGKDYIEFNK | IPI00924948, IPI00871622, IPI00939278, IPI00816309, IPI00166729, IPI00924815 | alpha-2-glycoprotein 1, zinc | AZGP1 |
| DWSFYLLYYTEFTPTEKDEYACR | IPI00930068, IPI00004656, IPI00796379, IPI00941461 | Beta-2-microglobulin | B2M |
| SNFLNCYVSGFHPSDIEVDLLK | IPI00930068, IPI00004656, IPI00796379, IPI00941461 | Beta-2-microglobulin | B2M |
| VNHVTLSQPK | IPI00930068, IPI00004656, IPI00796379, IPI00941461 | Beta-2-microglobulin | B2M |
| ALGLVPQAHPGFLTAWPADR | IPI00184094 | UDP-GlcNAc:betaGal beta-1,3-N-acetylglucosaminyltransferase 8 | B3GALT7 |
| AQDDAFVHTPALLAHLR | IPI00184094 | UDP-GlcNAc:betaGal beta-1,3-N-acetylglucosaminyltransferase 8 | B3GALT7 |
| DLLLLAWLGR | IPI00184094 | UDP-GlcNAc:betaGal beta-1,3-N-acetylglucosaminyltransferase 8 | B3GALT7 |
| LAPWLLR | IPI00184094 | UDP-GlcNAc:betaGal beta-1,3-N-acetylglucosaminyltransferase 8 | B3GALT7 |
| FKDFLLYLR | IPI00217345, IPI00745207, IPI00257239 | Isoform 2 of UDP-GlcNAc:betaGal beta-1,3-N-acetylglucosaminyltransferase 2 | B3GNT1 |
| VTSVVTGFNNLPDR | IPI00217345, IPI00745207, IPI00257239 | Isoform 2 of UDP-GlcNAc:betaGal beta-1,3-N-acetylglucosaminyltransferase 2 | B3GNT1 |
| EMLDQSNQWGGTALVVPAFEIR | IPI00009997 | N-acetyllactosaminide beta-1,3-N-acetylglucosaminyltransferase | B3GNT6 |
| EPGEFALLR | IPI00009997 | N-acetyllactosaminide beta-1,3-N-acetylglucosaminyltransferase | B3GNT6 |
| TALASGGVLDASGDYR | IPI00009997 | N-acetyllactosaminide beta-1,3-N-acetylglucosaminyltransferase | B3GNT6 |
| QQLDYGIYVINQAGDTIFNR | IPI00215767, IPI00759755 | Isoform Long of Beta-1,4-galactosyltransferase 1 | B4GALT1 |
| AAEAAAAPAESAAPAAGEEPSKEEGEPK | IPI00299024 | Brain acid soluble protein 1 | BASP1 |
| AEPPKAPEQEQAAPGPAAGGEAPK | IPI00299024 | Brain acid soluble protein 1 | BASP1 |
| ESEPQAAAEPAEAK | IPI00908521, IPI00299024 | Brain acid soluble protein 1 | BASP1 |
| AEEILSR | IPI00025864 | Cholinesterase precursor | BCHE |
| AILQSGSFNAPWAVTSLYEAR | IPI00025864 | Cholinesterase precursor | BCHE |
| DEGTAFLVYGAPGFSK | IPI00025864 | Cholinesterase precursor | BCHE |
| EALGDVVGDYNFICPALEFTK | IPI00025864 | Cholinesterase precursor | BCHE |
| ESILFHYTDWVDDQRPENYR | IPI00025864 | Cholinesterase precursor | BCHE |
| FSEWGNNAFFYYFEHR | IPI00025864 | Cholinesterase precursor | BCHE |
| FWTSFFPK | IPI00025864, IPI00797309 | Cholinesterase precursor | BCHE |
| IFFPGVSEFGK | IPI00025864 | Cholinesterase precursor | BCHE |
| KEFQEGLK | IPI00025864 | Cholinesterase precursor | BCHE |
| KFSEWGNNAFFYYFEHR | IPI00025864 | Cholinesterase precursor | BCHE |
| NIAAFGGNPK | IPI00025864 | Cholinesterase precursor | BCHE |
| NQFNDYTSK | IPI00025864, IPI00797309 | Cholinesterase precursor | BCHE |
| TQILVGVNKDEGTAFLVYGAPGFSK | IPI00025864 | Cholinesterase precursor | BCHE |
| VIVVSMNYR | IPI00025864 | Cholinesterase precursor | BCHE |
| WANFAK | IPI00025864 | Cholinesterase precursor | BCHE |
| WNNYMMDWK | IPI00025864, IPI00797309 | Cholinesterase precursor | BCHE |
| YLTLNTESTR | IPI00025864 | Cholinesterase precursor | BCHE |
| QGFIDLPEFPFGLEPR | IPI00004101, IPI00910055 | Betaine--homocysteine S-methyltransferase 1 | BHMT |
| LPSEGPRPAHVVVGDVLQAADVDK | IPI00783862, IPI00219910 | Flavin reductase | BLVRB |
| TVAGQDAVIVLLGTR | IPI00783862, IPI00219910 | Flavin reductase | BLVRB |
| YVAVMPPHIGDQPLTGAYTVTLDGR | IPI00783862, IPI00219910 | Flavin reductase | BLVRB |
| LLPYWNER | IPI00215979 | Bisphosphoglycerate mutase | BPGM |
| ALLSPEQR | IPI00026240, IPI00657860 | ADP-ribosyl cyclase 2 precursor | BST1 |
| DMGFQYSCINDYRPVK | IPI00026240, IPI00657860 | ADP-ribosyl cyclase 2 precursor | BST1 |
| FMPLSDVLYGR | IPI00026240, IPI00657860 | ADP-ribosyl cyclase 2 precursor | BST1 |
| GFFADYEIPNLQK | IPI00026240, IPI00657860 | ADP-ribosyl cyclase 2 precursor | BST1 |
| IEIWVMHEIGGPNVESCGEGSMK | IPI00026240, IPI00657860 | ADP-ribosyl cyclase 2 precursor | BST1 |
| SLFWENSHLLVNSFADNTR | IPI00026240, IPI00657860 | ADP-ribosyl cyclase 2 precursor | BST1 |
| VADFLSWCR | IPI00026240, IPI00657860 | ADP-ribosyl cyclase 2 precursor | BST1 |
| DAQEVHCDEATK | IPI00218413, IPI00744685, IPI00789424, IPI00926737, IPI00927683 | Biotinidase | BTD |
| GDMFLVANLGTK | IPI00218413, IPI00744685, IPI00789424, IPI00926737, IPI00927683 | Biotinidase | BTD |
| HNLYFEAAFDVPLKVDLITFDTPFAGR | IPI00218413, IPI00744685, IPI00789424, IPI00926737, IPI00927683 | Biotinidase | BTD |
| HVVYPTAWMNQLPLLAAIEIQK | IPI00218413, IPI00744685, IPI00789424, IPI00926737, IPI00927683 | Biotinidase | BTD |
| ILSGDPYCEK | IPI00218413, IPI00744685, IPI00789424, IPI00926737, IPI00927683 | Biotinidase | BTD |
| ILSGDPYCEKDAQEVHCDEATK | IPI00218413, IPI00744685, IPI00789424, IPI00926737, IPI00927683 | Biotinidase | BTD |
| KHNLYFEAAFDVPLK | IPI00218413, IPI00744685, IPI00789424, IPI00926737, IPI00927683 | Biotinidase | BTD |
| LSSGLVTAALYGR | IPI00218413, IPI00744685, IPI00789424, IPI00926737, IPI00927683 | Biotinidase | BTD |
| QEALELMNQNLDIYEQQVMTAAQK | IPI00218413, IPI00744685, IPI00789424, IPI00926737, IPI00927683 | Biotinidase | BTD |
| SHLIIAQVAK | IPI00218413, IPI00744685, IPI00789424, IPI00926737, IPI00927683 | Biotinidase | BTD |
| TSIYPFLDFMPSPQVVR | IPI00218413, IPI00744685, IPI00789424, IPI00926737, IPI00927683 | Biotinidase | BTD |
| VDLITFDTPFAGR | IPI00218413, IPI00744685, IPI00789424, IPI00926737, IPI00927683 | Biotinidase | BTD |
| WNPCLEPHR | IPI00218413, IPI00744685, IPI00789424, IPI00926737, IPI00927683 | Biotinidase | BTD |
| QTHQPPAPNSLIR | IPI00022394 | Complement C1q subcomponent subunit C precursor | C1QG |
| CVSLLLDLSQPLLPSR | IPI00909029, IPI00299485 | Complement component C1q receptor | C1QR1 |
| LLDDLVTCASR | IPI00909029, IPI00299485 | Complement component C1q receptor | C1QR1 |
| LSAAEAQNHCNQNGGNLATVK | IPI00909029, IPI00299485 | Complement component C1q receptor | C1QR1 |
| EFMSQGNK | IPI00296165 | Complement C1r subcomponent precursor | C1R |
| FCGQLGSPLGNPPGK | IPI00296165 | Complement C1r subcomponent precursor | C1R |
| FCGQLGSPLGNPPGKK | IPI00296165 | Complement C1r subcomponent precursor | C1R |
| FLEPFDIDDHQQVHCPYDQLQIYANGK | IPI00296165 | Complement C1r subcomponent precursor | C1R |
| KEFMSQGNK | IPI00296165 | Complement C1r subcomponent precursor | C1R |
| LFGEVTSPLFPKPYPNNFETTTVITVPTGYR | IPI00296165 | Complement C1r subcomponent precursor | C1R |
| LVFQQFDLEPSEGCFYDYVK | IPI00296165 | Complement C1r subcomponent precursor | C1R |
| WILTAAHTVYPK | IPI00795055 | CDNA FLJ14022 fis, clone HEMBA1003538, weakly similar to COMPLEMENT C1R COMPONENT | C1RL |
| APEGFAVR | IPI00791901, IPI00872573, IPI00009793, IPI00296165, IPI00941440, IPI00795931, IPI00441082, IPI00923551, IPI00795055 | Complement C1r subcomponent-like protein | C1RL |
| CLPVCGKPVNPVEQR | IPI00791901, IPI00872573, IPI00009793, IPI00296165, IPI00941440, IPI00795931, IPI00441082, IPI00923551, IPI00795055 | Complement C1r subcomponent-like protein | C1RL |
| DYFIATCK | IPI00791901, IPI00872573, IPI00009793, IPI00296165, IPI00941440, IPI00795931, IPI00441082, IPI00923551, IPI00795055 | Complement C1r subcomponent-like protein | C1RL |
| EACNAWLQK | IPI00791901, IPI00872573, IPI00009793, IPI00296165, IPI00941440, IPI00795931, IPI00441082, IPI00923551, IPI00795055 | Complement C1r subcomponent-like protein | C1RL |
| GFLAYYQAVDLDECASR | IPI00791901, IPI00872573, IPI00009793, IPI00296165, IPI00941440, IPI00795931, IPI00441082, IPI00923551, IPI00795055 | Complement C1r subcomponent-like protein | C1RL |
| GGGALLGDR | IPI00791901, IPI00872573, IPI00009793, IPI00296165, IPI00941440, IPI00795931, IPI00441082, IPI00923551, IPI00795055 | Complement C1r subcomponent-like protein | C1RL |
| GSEAINAPGDNPAK | IPI00791901, IPI00872573, IPI00009793, IPI00296165, IPI00941440, IPI00795931, IPI00441082, IPI00923551, IPI00795055 | Complement C1r subcomponent-like protein | C1RL |
| HSCQAECSSELYTEASGYISSLEYPR | IPI00791901, IPI00872573, IPI00009793, IPI00296165, IPI00941440, IPI00795931, IPI00441082, IPI00923551, IPI00795055 | Complement C1r subcomponent-like protein | C1RL |
| IKDCGQPR | IPI00791901, IPI00872573, IPI00009793, IPI00296165, IPI00941440, IPI00795931, IPI00441082, IPI00923551, IPI00795055 | Complement C1r subcomponent-like protein | C1RL |
| IQYYCHEPYYK | IPI00791901, IPI00872573, IPI00009793, IPI00296165, IPI00941440, IPI00795931, IPI00441082, IPI00923551, IPI00795055 | Complement C1r subcomponent-like protein | C1RL |
| LGNFPWQAFTSIHGR | IPI00791901, IPI00872573, IPI00009793, IPI00296165, IPI00941440, IPI00795931, IPI00441082, IPI00923551, IPI00795055 | Complement C1r subcomponent-like protein | C1RL |
| LPVANPQACENWLR | IPI00791901, IPI00872573, IPI00009793, IPI00296165, IPI00941440, IPI00795931, IPI00441082, IPI00923551, IPI00795055 | Complement C1r subcomponent-like protein | C1RL |
| MDVFSQNMFCAGHPSLK | IPI00791901, IPI00872573, IPI00009793, IPI00296165, IPI00941440, IPI00795931, IPI00441082, IPI00923551, IPI00795055 | Complement C1r subcomponent-like protein | C1RL |
| MGNFPWQVFTNIHGR | IPI00791901, IPI00872573, IPI00009793, IPI00296165, IPI00941440, IPI00795931, IPI00441082, IPI00923551, IPI00795055 | Complement C1r subcomponent-like protein | C1RL |
| NIGEFCGK | IPI00791901, IPI00872573, IPI00009793, IPI00296165, IPI00941440, IPI00795931, IPI00441082, IPI00923551, IPI00795055 | Complement C1r subcomponent-like protein | C1RL |
| NLPNGDFR | IPI00791901, IPI00872573, IPI00009793, IPI00296165, IPI00941440, IPI00795931, IPI00441082, IPI00923551, IPI00795055 | Complement C1r subcomponent-like protein | C1RL |
| QDACQGDSGGVFAVR | IPI00791901, IPI00872573, IPI00009793, IPI00296165, IPI00941440, IPI00795931, IPI00441082, IPI00923551, IPI00795055 | Complement C1r subcomponent-like protein | C1RL |
| QGYQLIEGNQVLHSFTAVCQDDGTWHR | IPI00791901, IPI00872573, IPI00009793, IPI00296165, IPI00941440, IPI00795931, IPI00441082, IPI00923551, IPI00795055 | Complement C1r subcomponent-like protein | C1RL |
| QRPPDLDTSSNAVDLLFFTDESGDSR | IPI00791901, IPI00872573, IPI00009793, IPI00296165, IPI00941440, IPI00795931, IPI00441082, IPI00923551, IPI00795055 | Complement C1r subcomponent-like protein | C1RL |
| SGLLGYVSGFGMEMGWLTTELK | IPI00791901, IPI00872573, IPI00009793, IPI00296165, IPI00941440, IPI00795931, IPI00441082, IPI00923551, IPI00795055 | Complement C1r subcomponent-like protein | C1RL |
| TLDEFTIIQNLQPQYQFR | IPI00791901, IPI00872573, IPI00009793, IPI00296165, IPI00941440, IPI00795931, IPI00441082, IPI00923551, IPI00795055 | Complement C1r subcomponent-like protein | C1RL |
| VLNYVDWIK | IPI00791901, IPI00872573, IPI00009793, IPI00296165, IPI00941440, IPI00795931, IPI00441082, IPI00923551, IPI00795055 | Complement C1r subcomponent-like protein | C1RL |
| VQNHCQEPYYQAAAAGALTCATPGTWK | IPI00791901, IPI00872573, IPI00009793, IPI00296165, IPI00941440, IPI00795931, IPI00441082, IPI00923551, IPI00795055 | Complement C1r subcomponent-like protein | C1RL |
| VVVHPDYR | IPI00791901, IPI00872573, IPI00009793, IPI00296165, IPI00941440, IPI00795931, IPI00441082, IPI00923551, IPI00795055 | Complement C1r subcomponent-like protein | C1RL |
| WILTAAHTLYPK | IPI00791901, IPI00872573, IPI00009793, IPI00296165, IPI00941440, IPI00795931, IPI00441082, IPI00923551, IPI00795055 | Complement C1r subcomponent-like protein | C1RL |
| WVATGIVSWGIGCSR | IPI00791901, IPI00872573, IPI00009793, IPI00296165, IPI00941440, IPI00795931, IPI00441082, IPI00923551, IPI00795055 | Complement C1r subcomponent-like protein | C1RL |
| YTTTMGVNTYK | IPI00791901, IPI00872573, IPI00009793, IPI00296165, IPI00941440, IPI00795931, IPI00441082, IPI00923551, IPI00795055 | Complement C1r subcomponent-like protein | C1RL |
| QRPEVFSDNMFCVGDETQR | IPI00009793 | Complement C1r-like protein | C1RL |
| VLSYVDWIK | IPI00009793 | Complement C1r-like protein | C1RL |
| CQPVDCGIPESIENGK | IPI00791987, IPI00385294, IPI00878772, IPI00017696, IPI00790679, IPI00877989, IPI00749179 | Complement C1s subcomponent | C1S |
| CQPVDCGIPESIENGKVEDPESTLFGSVIR | IPI00791987, IPI00385294, IPI00878772, IPI00017696, IPI00790679, IPI00877989, IPI00749179 | Complement C1s subcomponent | C1S |
| CVPVCGVPR | IPI00791987, IPI00385294, IPI00878772, IPI00017696, IPI00790679, IPI00877989, IPI00749179 | Complement C1s subcomponent | C1S |
| DVVQITCLDGFEVVEGR | IPI00791987, IPI00385294, IPI00878772, IPI00017696, IPI00790679, IPI00877989, IPI00749179 | Complement C1s subcomponent | C1S |
| EDFDVEAADSAGNCLDSLVFVAGDR | IPI00791987, IPI00385294, IPI00878772, IPI00017696, IPI00790679, IPI00877989, IPI00749179 | Complement C1s subcomponent | C1S |
| EDTPNSVWEPAK | IPI00791987, IPI00385294, IPI00878772, IPI00017696, IPI00790679, IPI00877989, IPI00749179 | Complement C1s subcomponent | C1S |
| EPTMYVGSTSVQTSR | IPI00791987, IPI00385294, IPI00878772, IPI00017696, IPI00790679, IPI00877989, IPI00749179 | Complement C1s subcomponent | C1S |
| FYAAGLVSWGPQCGTYGLYTR | IPI00791987, IPI00385294, IPI00878772, IPI00017696, IPI00790679, IPI00877989, IPI00749179 | Complement C1s subcomponent | C1S |
| GDSGGAFAVQDPNDK | IPI00791987, IPI00385294, IPI00878772, IPI00017696, IPI00790679, IPI00877989, IPI00749179 | Complement C1s subcomponent | C1S |
| GDSGGAFAVQDPNDKTK | IPI00791987, IPI00385294, IPI00878772, IPI00017696, IPI00790679, IPI00877989, IPI00749179 | Complement C1s subcomponent | C1S |
| GFQVVVTLR | IPI00791987, IPI00385294, IPI00878772, IPI00017696, IPI00790679, IPI00877989, IPI00749179 | Complement C1s subcomponent | C1S |
| IIGGSDADIK | IPI00791987, IPI00385294, IPI00878772, IPI00017696, IPI00790679, IPI00877989, IPI00749179 | Complement C1s subcomponent | C1S |
| LKCQPVDCGIPESIENGKVEDPESTLFGSVIR | IPI00791987, IPI00385294, IPI00878772, IPI00017696, IPI00790679, IPI00877989, IPI00749179 | Complement C1s subcomponent | C1S |
| LLEVPEGR | IPI00791987, IPI00385294, IPI00878772, IPI00017696, IPI00790679, IPI00877989, IPI00749179 | Complement C1s subcomponent | C1S |
| LPVAPLRK | IPI00791987, IPI00385294, IPI00878772, IPI00017696, IPI00790679, IPI00877989, IPI00749179 | Complement C1s subcomponent | C1S |
| MGPTVSPICLPGTSSDYNLMDGDLGLISGWGR | IPI00791987, IPI00385294, IPI00878772, IPI00017696, IPI00790679, IPI00877989, IPI00749179 | Complement C1s subcomponent | C1S |
| MLTPEHVFIHPGWK | IPI00791987, IPI00385294, IPI00878772, IPI00017696, IPI00790679, IPI00877989, IPI00749179 | Complement C1s subcomponent | C1S |
| NYVDWIMK | IPI00791987, IPI00385294, IPI00878772, IPI00017696, IPI00790679, IPI00877989, IPI00749179 | Complement C1s subcomponent | C1S |
| QFGPYCGHGFPGPLNIETK | IPI00791987, IPI00385294, IPI00878772, IPI00017696, IPI00790679, IPI00877989, IPI00749179 | Complement C1s subcomponent | C1S |
| REDFDVEAADSAGNCLDSLVFVAGDR | IPI00791987, IPI00385294, IPI00878772, IPI00017696, IPI00790679, IPI00877989, IPI00749179 | Complement C1s subcomponent | C1S |
| SNALDIIFQTDLTGQK | IPI00791987, IPI00385294, IPI00878772, IPI00017696, IPI00790679, IPI00877989, IPI00749179 | Complement C1s subcomponent | C1S |
| SSNNPHSPIVEEFQVPYNK | IPI00791987, IPI00385294, IPI00878772, IPI00017696, IPI00790679, IPI00877989, IPI00749179 | Complement C1s subcomponent | C1S |
| TNFDNDIALVR | IPI00791987, IPI00385294, IPI00878772, IPI00017696, IPI00790679, IPI00877989, IPI00749179 | Complement C1s subcomponent | C1S |
| VEDPESTLFGSVIR | IPI00791987, IPI00385294, IPI00878772, IPI00017696, IPI00790679, IPI00877989, IPI00749179 | Complement C1s subcomponent | C1S |
| VEKPTADAEAYVFTPNMICAGGEK | IPI00791987, IPI00385294, IPI00878772, IPI00017696, IPI00790679, IPI00877989, IPI00749179 | Complement C1s subcomponent | C1S |
| VGATSFYSTCQSNGK | IPI00791987, IPI00385294, IPI00878772, IPI00017696, IPI00790679, IPI00877989, IPI00749179 | Complement C1s subcomponent | C1S |
| VGATSFYSTCQSNGKWSNSK | IPI00791987, IPI00385294, IPI00878772, IPI00017696, IPI00790679, IPI00877989, IPI00749179 | Complement C1s subcomponent | C1S |
| VKNYVDWIMK | IPI00791987, IPI00385294, IPI00878772, IPI00017696, IPI00790679, IPI00877989, IPI00749179 | Complement C1s subcomponent | C1S |
| YHGDPMPCPK | IPI00791987, IPI00385294, IPI00878772, IPI00017696, IPI00790679, IPI00877989, IPI00749179 | Complement C1s subcomponent | C1S |
| ACEPGVDYVYK | IPI00739237, IPI00942927, IPI00783987, IPI00887739 | Complement component 3 | C3 |
| ADIGCTPGSGKDYAGVFSDAGLTFTSSSGQQTAQR | IPI00739237, IPI00942927, IPI00783987, IPI00887739 | Complement component 3 | C3 |
| AEDLVGKSLYVSATVILHSGSDMVQAER | IPI00739237, IPI00942927, IPI00783987, IPI00887739 | Complement component 3 | C3 |
| AGDFLEANYMNLQR | IPI00739237, IPI00942927, IPI00783987, IPI00887739 | Complement component 3 | C3 |
| AKDQLTCNK | IPI00739237, IPI00942927, IPI00783987, IPI00887739 | Complement component 3 | C3 |
| AYYENSPQQVFSTEFEVK | IPI00739237, IPI00942927, IPI00783987, IPI00887739 | Complement component 3 | C3 |
| CAEENCFIQK | IPI00739237, IPI00942927, IPI00783987, IPI00887739 | Complement component 3 | C3 |
| DAPDHQELNLDVSLQLPSR | IPI00739237, IPI00942927, IPI00783987, IPI00887739 | Complement component 3 | C3 |
| DFDFVPPVVR | IPI00739237, IPI00942927, IPI00783987, IPI00887739 | Complement component 3 | C3 |
| DICEEQVNSLPGSITK | IPI00739237, IPI00942927, IPI00783987, IPI00887739 | Complement component 3 | C3 |
| DMALTAFVLISLQEAK | IPI00739237, IPI00942927, IPI00783987, IPI00887739 | Complement component 3 | C3 |
| DSCVGSLVVK | IPI00739237, IPI00942927, IPI00783987, IPI00887739 | Complement component 3 | C3 |
| DTWVEHWPEEDECQDEENQK | IPI00739237, IPI00942927, IPI00783987, IPI00887739 | Complement component 3 | C3 |
| DYAGVFSDAGLTFTSSSGQQTAQR | IPI00739237, IPI00942927, IPI00783987, IPI00887739 | Complement component 3 | C3 |
| EALKLEEK | IPI00739237, IPI00942927, IPI00783987, IPI00887739 | Complement component 3 | C3 |
| EDIPPADLSDQVPDTESETR | IPI00739237, IPI00942927, IPI00783987, IPI00887739 | Complement component 3 | C3 |
| EGVQKEDIPPADLSDQVPDTESETR | IPI00739237, IPI00942927, IPI00783987, IPI00887739 | Complement component 3 | C3 |
| ENEGFTVTAEGK | IPI00739237, IPI00942927, IPI00783987, IPI00887739 | Complement component 3 | C3 |
| EPGQDLVVLPLSITTDFIPSFR | IPI00739237, IPI00942927, IPI00783987, IPI00887739 | Complement component 3 | C3 |
| EVVADSVWVDVK | IPI00739237, IPI00942927, IPI00783987, IPI00887739 | Complement component 3 | C3 |
| FISLGEACK | IPI00739237, IPI00942927, IPI00783987, IPI00887739 | Complement component 3 | C3 |
| FLYGKKVEGTAFVIFGIQDGEQR | IPI00739237, IPI00942927, IPI00783987, IPI00887739 | Complement component 3 | C3 |
| FYYIYNEK | IPI00739237, IPI00942927, IPI00783987, IPI00887739 | Complement component 3 | C3 |
| GDQDATMSILDISMMTGFAPDTDDLK | IPI00739237, IPI00942927, IPI00783987, IPI00887739 | Complement component 3 | C3 |
| GICVADPFEVTVMQDFFIDLR | IPI00739237, IPI00942927, IPI00783987, IPI00887739 | Complement component 3 | C3 |
| GLEVTITAR | IPI00739237, IPI00942927, IPI00783987, IPI00887739 | Complement component 3 | C3 |
| GYTQQLAFR | IPI00739237, IPI00942927, IPI00783987, IPI00887739 | Complement component 3 | C3 |
| IHWESASLLR | IPI00739237, IPI00942927, IPI00783987, IPI00887739 | Complement component 3 | C3 |
| ILLQGTPVAQMTEDAVDAER | IPI00739237, IPI00942927, IPI00783987, IPI00887739 | Complement component 3 | C3 |
| IPIEDGSGEVVLSR | IPI00739237, IPI00942927, IPI00783987, IPI00887739 | Complement component 3 | C3 |
| ISLPESLK | IPI00739237, IPI00942927, IPI00783987, IPI00887739 | Complement component 3 | C3 |
| KGYTQQLAFR | IPI00739237, IPI00942927, IPI00783987, IPI00887739 | Complement component 3 | C3 |
| KQELSEAEQATR | IPI00739237, IPI00942927, IPI00783987, IPI00887739 | Complement component 3 | C3 |
| KVEGTAFVIFGIQDGEQR | IPI00739237, IPI00942927, IPI00783987, IPI00887739 | Complement component 3 | C3 |
| KVFLDCCNYITELR | IPI00739237, IPI00942927, IPI00783987, IPI00887739 | Complement component 3 | C3 |
| LESEETMVLEAHDAQGDVPVTVTVHDFPGK | IPI00739237, IPI00942927, IPI00783987, IPI00887739 | Complement component 3 | C3 |
| LKGPLLNK | IPI00739237, IPI00942927, IPI00783987, IPI00887739 | Complement component 3 | C3 |
| LVAYYTLIGASGQR | IPI00739237, IPI00942927, IPI00783987, IPI00887739 | Complement component 3 | C3 |
| NTLIIYLDK | IPI00739237, IPI00942927, IPI00783987, IPI00887739 | Complement component 3 | C3 |
| NTMILEICTR | IPI00739237, IPI00942927, IPI00783987, IPI00887739 | Complement component 3 | C3 |
| QDSLSSQNQLGVLPLSWDIPELVNMGQWK | IPI00739237, IPI00942927, IPI00783987, IPI00887739 | Complement component 3 | C3 |
| QELSEAEQATR | IPI00739237, IPI00942927, IPI00783987, IPI00887739 | Complement component 3 | C3 |
| QGALELIK | IPI00739237, IPI00942927, IPI00783987, IPI00887739 | Complement component 3 | C3 |
| QKPDGVFQEDAPVIHQEMIGGLR | IPI00739237, IPI00942927, IPI00783987, IPI00887739 | Complement component 3 | C3 |
| QLYNVEATSYALLALLQLK | IPI00739237, IPI00942927, IPI00783987, IPI00887739 | Complement component 3 | C3 |
| QPSSAFAAFVK | IPI00739237, IPI00942927, IPI00783987, IPI00887739 | Complement component 3 | C3 |
| QPVPGQQMTLK | IPI00739237, IPI00942927, IPI00783987, IPI00887739 | Complement component 3 | C3 |
| RIPIEDGSGEVVLSR | IPI00739237, IPI00942927, IPI00783987, IPI00887739 | Complement component 3 | C3 |
| RQGALELIK | IPI00739237, IPI00942927, IPI00783987, IPI00887739 | Complement component 3 | C3 |
| SEETKENEGFTVTAEGK | IPI00739237, IPI00942927, IPI00783987, IPI00887739 | Complement component 3 | C3 |
| SEFPESWLWNVEDLKEPPK | IPI00739237, IPI00942927, IPI00783987, IPI00887739 | Complement component 3 | C3 |
| SGIPIVTSPYQIHFTK | IPI00739237, IPI00942927, IPI00783987, IPI00887739 | Complement component 3 | C3 |
| SGQSEDRQPVPGQQMTLK | IPI00739237, IPI00942927, IPI00783987, IPI00887739 | Complement component 3 | C3 |
| SLYVSATVILHSGSDMVQAER | IPI00739237, IPI00942927, IPI00783987, IPI00887739 | Complement component 3 | C3 |
| SNLDEDIIAEENIVSR | IPI00739237, IPI00942927, IPI00783987, IPI00887739 | Complement component 3 | C3 |
| SSLSVPYVIVPLK | IPI00739237, IPI00942927, IPI00783987, IPI00887739 | Complement component 3 | C3 |
| SYTVAIAGYALAQMGR | IPI00739237, IPI00942927, IPI00783987, IPI00887739 | Complement component 3 | C3 |
| TELRPGETLNVNFLLR | IPI00739237, IPI00942927, IPI00783987, IPI00887739 | Complement component 3 | C3 |
| TGLQEVEVK | IPI00739237, IPI00942927, IPI00783987, IPI00887739 | Complement component 3 | C3 |
| TIYTPGSTVLYR | IPI00739237, IPI00942927, IPI00783987, IPI00887739 | Complement component 3 | C3 |
| TVMVNIENPEGIPVK | IPI00739237, IPI00942927, IPI00783987, IPI00887739 | Complement component 3 | C3 |
| VEGTAFVIFGIQDGEQR | IPI00739237, IPI00942927, IPI00783987, IPI00887739 | Complement component 3 | C3 |
| VELLHNPAFCSLATTK | IPI00739237, IPI00942927, IPI00783987, IPI00887739 | Complement component 3 | C3 |
| VFLDCCNYITELR | IPI00739237, IPI00942927, IPI00783987, IPI00887739 | Complement component 3 | C3 |
| VHQYFNVELIQPGAVK | IPI00739237, IPI00942927, IPI00783987, IPI00887739 | Complement component 3 | C3 |
| VPVAVQGEDTVQSLTQGDGVAK | IPI00739237, IPI00942927, IPI00783987, IPI00887739 | Complement component 3 | C3 |
| VQLSNDFDEYIMAIEQTIK | IPI00739237, IPI00942927, IPI00783987, IPI00887739 | Complement component 3 | C3 |
| VYAYYNLEESCTR | IPI00739237, IPI00942927, IPI00783987, IPI00887739 | Complement component 3 | C3 |
| YFKPGMPFDLMVFVTNPDGSPAYR | IPI00739237, IPI00942927, IPI00783987, IPI00887739 | Complement component 3 | C3 |
| YRGDQDATMSILDISMMTGFAPDTDDLK | IPI00739237, IPI00942927, IPI00783987, IPI00887739 | Complement component 3 | C3 |
| YYTYLIMNK | IPI00739237, IPI00942927, IPI00783987, IPI00887739 | Complement component 3 | C3 |
| AEMADQAAAWLTR | IPI00887154, IPI00654875, IPI00643525, IPI00922744, IPI00937598, IPI00892547, IPI00032258, IPI00418163, IPI00935601, IPI00892604, IPI00889723, IPI00843913 | Complement C4-A | C4A |
| ALEILQEEDLIDEDDIPVR | IPI00887154, IPI00654875, IPI00643525, IPI00922744, IPI00937598, IPI00892547, IPI00032258, IPI00418163, IPI00935601, IPI00892604, IPI00889723, IPI00843913 | Complement C4-A | C4A |
| ASAGLLGAHAAAITAYALTLTK | IPI00887154, IPI00654875, IPI00643525, IPI00922744, IPI00937598, IPI00892547, IPI00032258, IPI00418163, IPI00935601, IPI00892604, IPI00889723, IPI00843913 | Complement C4-A | C4A |
| AVGSGATFSHYYYMILSR | IPI00887154, IPI00654875, IPI00643525, IPI00922744, IPI00937598, IPI00892547, IPI00032258, IPI00418163, IPI00935601, IPI00892604, IPI00889723, IPI00843913 | Complement C4-A | C4A |
| DDPDAPLQPVTPLQLFEGR | IPI00887154, IPI00654875, IPI00643525, IPI00922744, IPI00937598, IPI00892547, IPI00032258, IPI00418163, IPI00935601, IPI00892604, IPI00889723, IPI00843913 | Complement C4-A | C4A |
| DFALLSLQVPLKDAK | IPI00887154, IPI00654875, IPI00643525, IPI00922744, IPI00937598, IPI00892547, IPI00032258, IPI00418163, IPI00935601, IPI00892604, IPI00889723, IPI00843913 | Complement C4-A | C4A |
| DSSTWLTAFVLK | IPI00887154, IPI00654875, IPI00643525, IPI00922744, IPI00937598, IPI00892547, IPI00032258, IPI00418163, IPI00935601, IPI00892604, IPI00889723, IPI00843913 | Complement C4-A | C4A |
| EELVYELNPLDHR | IPI00887154, IPI00654875, IPI00643525, IPI00922744, IPI00937598, IPI00892547, IPI00032258, IPI00418163, IPI00935601, IPI00892604, IPI00889723, IPI00843913 | Complement C4-A | C4A |
| EMSGSPASGIPVK | IPI00887154, IPI00654875, IPI00643525, IPI00922744, IPI00937598, IPI00892547, IPI00032258, IPI00418163, IPI00935601, IPI00892604, IPI00889723, IPI00843913 | Complement C4-A | C4A |
| EPFLSCCQFAESLR | IPI00887154, IPI00654875, IPI00643525, IPI00922744, IPI00937598, IPI00892547, IPI00032258, IPI00418163, IPI00935601, IPI00892604, IPI00889723, IPI00843913 | Complement C4-A | C4A |
| GHLFLQTDQPIYNPGQR | IPI00887154, IPI00654875, IPI00643525, IPI00922744, IPI00937598, IPI00892547, IPI00032258, IPI00418163, IPI00935601, IPI00892604, IPI00889723, IPI00843913 | Complement C4-A | C4A |
| GLCVATPVQLR | IPI00887154, IPI00654875, IPI00643525, IPI00922744, IPI00937598, IPI00892547, IPI00032258, IPI00418163, IPI00935601, IPI00892604, IPI00889723, IPI00843913 | Complement C4-A | C4A |
| GLEEELQFSLGSK | IPI00887154, IPI00654875, IPI00643525, IPI00922744, IPI00937598, IPI00892547, IPI00032258, IPI00418163, IPI00935601, IPI00892604, IPI00889723, IPI00843913 | Complement C4-A | C4A |
| GSFEFPVGDAVSK | IPI00887154, IPI00654875, IPI00643525, IPI00922744, IPI00937598, IPI00892547, IPI00032258, IPI00418163, IPI00935601, IPI00892604, IPI00889723, IPI00843913 | Complement C4-A | C4A |
| HLVPGAPFLLQALVR | IPI00887154, IPI00654875, IPI00643525, IPI00922744, IPI00937598, IPI00892547, IPI00032258, IPI00418163, IPI00935601, IPI00892604, IPI00889723, IPI00843913 | Complement C4-A | C4A |
| ITPGKPYILTVPGHLDEMQLDIQAR | IPI00887154, IPI00654875, IPI00643525, IPI00922744, IPI00937598, IPI00892547, IPI00032258, IPI00418163, IPI00935601, IPI00892604, IPI00889723, IPI00843913 | Complement C4-A | C4A |
| ITQVLHFTK | IPI00887154, IPI00654875, IPI00643525, IPI00922744, IPI00937598, IPI00892547, IPI00032258, IPI00418163, IPI00935601, IPI00892604, IPI00889723, IPI00843913 | Complement C4-A | C4A |
| LLLFSPSVVHLGVPLSVGVQLQDVPR | IPI00887154, IPI00654875, IPI00643525, IPI00922744, IPI00937598, IPI00892547, IPI00032258, IPI00418163, IPI00935601, IPI00892604, IPI00889723, IPI00843913 | Complement C4-A | C4A |
| LNMGITDLQGLR | IPI00887154, IPI00654875, IPI00643525, IPI00922744, IPI00937598, IPI00892547, IPI00032258, IPI00418163, IPI00935601, IPI00892604, IPI00889723, IPI00843913 | Complement C4-A | C4A |
| LQETSNWLLSQQQADGSFQDLSPVIHR | IPI00887154, IPI00654875, IPI00643525, IPI00922744, IPI00937598, IPI00892547, IPI00032258, IPI00418163, IPI00935601, IPI00892604, IPI00889723, IPI00843913 | Complement C4-A | C4A |
| LQETSNWLLSQQQADGSFQDPCPVLDR | IPI00887154, IPI00654875, IPI00643525, IPI00922744, IPI00937598, IPI00892547, IPI00032258, IPI00418163, IPI00935601, IPI00892604, IPI00889723, IPI00843913 | Complement C4-A | C4A |
| LTVAAPPSGGPGFLSIERPDSRPPR | IPI00887154, IPI00654875, IPI00643525, IPI00922744, IPI00937598, IPI00892547, IPI00032258, IPI00418163, IPI00935601, IPI00892604, IPI00889723, IPI00843913 | Complement C4-A | C4A |
| SFFPENWLWR | IPI00887154, IPI00654875, IPI00643525, IPI00922744, IPI00937598, IPI00892547, IPI00032258, IPI00418163, IPI00935601, IPI00892604, IPI00889723, IPI00843913 | Complement C4-A | C4A |
| STQDTVIALDALSAYWIASHTTEER | IPI00887154, IPI00654875, IPI00643525, IPI00922744, IPI00937598, IPI00892547, IPI00032258, IPI00418163, IPI00935601, IPI00892604, IPI00889723, IPI00843913 | Complement C4-A | C4A |
| TLEIPGNSDPNMIPDGDFNSYVR | IPI00887154, IPI00654875, IPI00643525, IPI00922744, IPI00937598, IPI00892547, IPI00032258, IPI00418163, IPI00935601, IPI00892604, IPI00889723, IPI00843913 | Complement C4-A | C4A |
| TTNIQGINLLFSSR | IPI00887154, IPI00654875, IPI00643525, IPI00922744, IPI00937598, IPI00892547, IPI00032258, IPI00418163, IPI00935601, IPI00892604, IPI00889723, IPI00843913 | Complement C4-A | C4A |
| VDFTLSSER | IPI00887154, IPI00654875, IPI00643525, IPI00922744, IPI00937598, IPI00892547, IPI00032258, IPI00418163, IPI00935601, IPI00892604, IPI00889723, IPI00843913 | Complement C4-A | C4A |
| VDVQAGACEGK | IPI00887154, IPI00654875, IPI00643525, IPI00922744, IPI00937598, IPI00892547, IPI00032258, IPI00418163, IPI00935601, IPI00892604, IPI00889723, IPI00843913 | Complement C4-A | C4A |
| VGDTLNLNLR | IPI00887154, IPI00654875, IPI00643525, IPI00922744, IPI00937598, IPI00892547, IPI00032258, IPI00418163, IPI00935601, IPI00892604, IPI00889723, IPI00843913 | Complement C4-A | C4A |
| VGLSGMAIADVTLLSGFHALR | IPI00887154, IPI00654875, IPI00643525, IPI00922744, IPI00937598, IPI00892547, IPI00032258, IPI00418163, IPI00935601, IPI00892604, IPI00889723, IPI00843913 | Complement C4-A | C4A |
| VTASDPLDTLGSEGALSPGGVASLLR | IPI00887154, IPI00654875, IPI00643525, IPI00922744, IPI00937598, IPI00892547, IPI00032258, IPI00418163, IPI00935601, IPI00892604, IPI00889723, IPI00843913 | Complement C4-A | C4A |
| YVLPNFEVK | IPI00887154, IPI00654875, IPI00643525, IPI00922744, IPI00937598, IPI00892547, IPI00032258, IPI00418163, IPI00935601, IPI00892604, IPI00889723, IPI00843913 | Complement C4-A | C4A |
| YVSHFETEGPHVLLYFDSVPTSR | IPI00887154, IPI00654875, IPI00643525, IPI00922744, IPI00937598, IPI00892547, IPI00032258, IPI00418163, IPI00935601, IPI00892604, IPI00889723, IPI00843913 | Complement C4-A | C4A |
| LSLEIEQLELQR | IPI00021727, IPI00872510 | C4b-binding protein alpha chain precursor | C4BPA |
| WTPYQGCEALCCPEPK | IPI00021727 | C4b-binding protein alpha chain precursor | C4BPA |
| NLCEAMENFMQQLK | IPI00025862, IPI00555752 | Isoform 1 of C4b-binding protein beta chain | C4BPB |
| AFTECCVVASQLR | IPI00032291, IPI00816741 | Complement C5 precursor | C5 |
| CCYDGACVNNDETCEQR | IPI00032291, IPI00816741 | Complement C5 precursor | C5 |
| CVEADCGQMQEELDLTISAETR | IPI00032291 | Complement C5 precursor | C5 |
| DGHVILQLNSIPSSDFLCVR | IPI00032291 | Complement C5 precursor | C5 |
| DINYVNPVIK | IPI00032291 | Complement C5 precursor | C5 |
| DVFLEMNIPYSVVR | IPI00032291, IPI00816741 | Complement C5 precursor | C5 |
| EDLKDDQK | IPI00032291, IPI00816741 | Complement C5 precursor | C5 |
| EGMLSIMSYR | IPI00032291 | Complement C5 precursor | C5 |
| ELSYYSLEDLNNK | IPI00032291, IPI00816741 | Complement C5 precursor | C5 |
| ENSLYLTAFTVIGIR | IPI00032291 | Complement C5 precursor | C5 |
| ESYSGVTLDPR | IPI00032291, IPI00816741 | Complement C5 precursor | C5 |
| FSDASYQSINIPVTQNMVPSSR | IPI00032291, IPI00816741 | Complement C5 precursor | C5 |
| GALHNYK | IPI00032291 | Complement C5 precursor | C5 |
| GGSASTWLTAFALR | IPI00032291 | Complement C5 precursor | C5 |
| GIYGTISR | IPI00032291, IPI00816741 | Complement C5 precursor | C5 |
| GYGNSDYK | IPI00032291 | Complement C5 precursor | C5 |
| IDTQDIEASHYR | IPI00032291 | Complement C5 precursor | C5 |
| IVACASYKPSR | IPI00032291 | Complement C5 precursor | C5 |
| KCCYDGACVNNDETCEQR | IPI00032291, IPI00816741 | Complement C5 precursor | C5 |
| LNLVATPLFLKPGIPYPIK | IPI00032291, IPI00816741 | Complement C5 precursor | C5 |
| LQGTLPVEAR | IPI00032291 | Complement C5 precursor | C5 |
| MSAVEGICTSESPVIDHQGTK | IPI00032291, IPI00816741 | Complement C5 precursor | C5 |
| MVETTAYALLTSLNLK | IPI00032291 | Complement C5 precursor | C5 |
| NADYSYSVWK | IPI00032291 | Complement C5 precursor | C5 |
| QCTMFYSTSNIK | IPI00032291 | Complement C5 precursor | C5 |
| SYFPESWLWEVHLVPR | IPI00032291, IPI00816741 | Complement C5 precursor | C5 |
| TDAPDLPEENQAR | IPI00032291, IPI00816741 | Complement C5 precursor | C5 |
| TLLPVSKPEIR | IPI00032291, IPI00816741 | Complement C5 precursor | C5 |
| VFKDVFLEMNIPYSVVR | IPI00032291, IPI00816741 | Complement C5 precursor | C5 |
| VYSLNDDLKPAK | IPI00032291, IPI00816741 | Complement C5 precursor | C5 |
| VYSLNDDLKPAKR | IPI00032291, IPI00816741 | Complement C5 precursor | C5 |
| WLSEEQR | IPI00032291 | Complement C5 precursor | C5 |
| YGGGFYSTQDTINAIEGLTEYSLLVK | IPI00032291 | Complement C5 precursor | C5 |
| YIYPLDSLTWIEYWPR | IPI00032291 | Complement C5 precursor | C5 |
| YNFSFR | IPI00032291 | Complement C5 precursor | C5 |
| ENPAVIDFELAPIVDLVR | IPI00879709 | Complement component 6 precursor | C6 |
| GEVLDNSFTGGICK | IPI00879709 | Complement component 6 precursor | C6 |
| GGNQLYCVK | IPI00879709 | Complement component 6 precursor | C6 |
| IGESIELTCPK | IPI00879709 | Complement component 6 precursor | C6 |
| KLECNGENDCGDNSDER | IPI00879709 | Complement component 6 precursor | C6 |
| QLEWGLER | IPI00879709 | Complement component 6 precursor | C6 |
| SEYGAALAWEK | IPI00879709 | Complement component 6 precursor | C6 |
| SVLRPSQFGGQPCTEPLVAFQPCIPSK | IPI00879709 | Complement component 6 precursor | C6 |
| TECIKPVVQEVLTITPFQR | IPI00879709 | Complement component 6 precursor | C6 |
| TFSEWLESVK | IPI00879709 | Complement component 6 precursor | C6 |
| EFPFDVDALFPER | IPI00844295, IPI00026795, IPI00903142, IPI00848136, IPI00794859, IPI00329132 | Isoform 4 of UPF0635 protein C6orf134 | C6orf134 |
| CFSGQCISK | IPI00909594, IPI00296608 | Complement component C7 | C7 |
| DGFVQDEGTMFPVGK | IPI00909594, IPI00296608 | Complement component C7 | C7 |
| FRCFSGQCISK | IPI00909594, IPI00296608 | Complement component C7 | C7 |
| LLEPHCFPLSLVPTEFCPSPPALK | IPI00909594, IPI00296608 | Complement component C7 | C7 |
| LTPLYELVK | IPI00909594, IPI00296608 | Complement component C7 | C7 |
| NVVYTCNEGYSLIGNPVAR | IPI00909594, IPI00296608 | Complement component C7 | C7 |
| QNDFNSVEEK | IPI00909594, IPI00296608 | Complement component C7 | C7 |
| SCVGETTESTQCEDEELEHLR | IPI00909594, IPI00296608 | Complement component C7 | C7 |
| SVAVYGQYGGQPCVGNAFETQSCEPTR | IPI00909594, IPI00296608 | Complement component C7 | C7 |
| VLFYVDSEK | IPI00909594, IPI00296608 | Complement component C7 | C7 |
| YSAWAESVTNLPQVIK | IPI00909594, IPI00296608 | Complement component C7 | C7 |
| DSCTLPASAEK | IPI00296608 | Complement component C7 precursor | C7 |
| ELSHLPSLYDYSAYR | IPI00296608 | Complement component C7 precursor | C7 |
| EQTMSECEAGALR | IPI00296608 | Complement component C7 precursor | C7 |
| GGGAGFISGLSYLELDNPAGNK | IPI00296608 | Complement component C7 precursor | C7 |
| GGGAGFISGLSYLELDNPAGNKR | IPI00296608 | Complement component C7 precursor | C7 |
| GQSISVTSIRPCAAETQ | IPI00296608 | Complement component C7 precursor | C7 |
| IACVLPVLMDGIQSHPQKPFYTVGEK | IPI00296608 | Complement component C7 precursor | C7 |
| ILPLTVCK | IPI00296608 | Complement component C7 precursor | C7 |
| LIDQYGTHYLQSGSLGGEYR | IPI00296608 | Complement component C7 precursor | C7 |
| LSGNVLSYTFQVK | IPI00296608 | Complement component C7 precursor | C7 |
| MPYECGPSLDVCAQDER | IPI00296608 | Complement component C7 precursor | C7 |
| RPSCDIDKPPPNIELTGNGYNELTGQFR | IPI00296608 | Complement component C7 precursor | C7 |
| SLVCNGDSDCDEDSADEDRCEDSER | IPI00296608 | Complement component C7 precursor | C7 |
| VTVSCSGGMSLEGPSAFLCGSSLK | IPI00296608 | Complement component C7 precursor | C7 |
| WLVGEMHCQK | IPI00296608 | Complement component C7 precursor | C7 |
| AIDEDCSQYEPIPGSQK | IPI00011252 | Complement component C8 alpha chain precursor | C8A |
| ALDQYLMEFNACR | IPI00011252 | Complement component C8 alpha chain precursor | C8A |
| AMAVEDIISR | IPI00011252 | Complement component C8 alpha chain precursor | C8A |
| CGPCFNNGVPILEGTSCR | IPI00011252 | Complement component C8 alpha chain precursor | C8A |
| DITTCFGGSLGIQYEDK | IPI00011252 | Complement component C8 alpha chain precursor | C8A |
| FGGTICSGDIWDQASCSSSTTCVR | IPI00011252 | Complement component C8 alpha chain precursor | C8A |
| HLVCNGDQDCLDGSDEDDCEDVR | IPI00011252 | Complement component C8 alpha chain precursor | C8A |
| LGSLGAACEQTQTEGAK | IPI00011252 | Complement component C8 alpha chain precursor | C8A |
| QAQCGQDFQCK | IPI00011252 | Complement component C8 alpha chain precursor | C8A |
| SLLQPNK | IPI00011252 | Complement component C8 alpha chain precursor | C8A |
| YNPVVIDFEMQPIHEVLR | IPI00011252 | Complement component C8 alpha chain precursor | C8A |
| CDCICPVGSQGLACEVSYR | IPI00294395 | Complement component C8 beta chain precursor | C8B |
| CEGFVCAQTGR | IPI00294395 | Complement component C8 beta chain precursor | C8B |
| CQHEMDQYWGIGSLASGINLFTNSFEGPVLDHR | IPI00294395 | Complement component C8 beta chain precursor | C8B |
| DFGTHYITEAVLGGIYEYTLVMNK | IPI00294395 | Complement component C8 beta chain precursor | C8B |
| DTMVEDLVVLVR | IPI00294395 | Complement component C8 beta chain precursor | C8B |
| EVSSCHCAPCQGNGVPVLK | IPI00294395 | Complement component C8 beta chain precursor | C8B |
| EYESYSDFER | IPI00294395 | Complement component C8 beta chain precursor | C8B |
| FRKPYNVESYTPQTQGK | IPI00294395 | Complement component C8 beta chain precursor | C8B |
| GDYTLNNVHACAK | IPI00294395 | Complement component C8 beta chain precursor | C8B |
| GILNEIKDR | IPI00294395 | Complement component C8 beta chain precursor | C8B |
| IGGAIEEVYVSLGVSVGK | IPI00294395 | Complement component C8 beta chain precursor | C8B |
| KNTPIDGK | IPI00294395 | Complement component C8 beta chain precursor | C8B |
| KPYNVESYTPQTQGK | IPI00294395 | Complement component C8 beta chain precursor | C8B |
| LLCNGDNDCGDQSDEANCR | IPI00294395 | Complement component C8 beta chain precursor | C8B |
| LPLEYSYGEYR | IPI00294395 | Complement component C8 beta chain precursor | C8B |
| NTPIDGK | IPI00294395 | Complement component C8 beta chain precursor | C8B |
| QALEEFQK | IPI00294395 | Complement component C8 beta chain precursor | C8B |
| RDTMVEDLVVLVR | IPI00294395 | Complement component C8 beta chain precursor | C8B |
| RLPLEYSYGEYR | IPI00294395 | Complement component C8 beta chain precursor | C8B |
| SDLEVAHYK | IPI00294395 | Complement component C8 beta chain precursor | C8B |
| SGFSFGFK | IPI00294395 | Complement component C8 beta chain precursor | C8B |
| SLMLHYEFLQR | IPI00294395 | Complement component C8 beta chain precursor | C8B |
| VEPLYELVTATDFAYSSTVR | IPI00294395 | Complement component C8 beta chain precursor | C8B |
| VKVEPLYELVTATDFAYSSTVR | IPI00294395 | Complement component C8 beta chain precursor | C8B |
| YYAGGCSPHYILNTR | IPI00294395 | Complement component C8 beta chain precursor | C8B |
| AEATTLHVAPQGTAMAVSTFR | IPI00011261, IPI00513935 | Complement component C8 gamma chain precursor | C8G |
| ANFDAQQFAGTWLLVAVGSACR | IPI00011261, IPI00513935 | Complement component C8 gamma chain precursor | C8G |
| QLYGDTGVLGR | IPI00011261, IPI00513935 | Complement component C8 gamma chain precursor | C8G |
| RPASPISTIQPK | IPI00011261, IPI00513935 | Complement component C8 gamma chain precursor | C8G |
| SLPVSDSVLSGFEQR | IPI00011261 | Complement component C8 gamma chain precursor | C8G |
| VQEAHLTEDQIFYFPK | IPI00011261 | Complement component C8 gamma chain precursor | C8G |
| YGFCEAADQFHVLDEVR | IPI00011261 | Complement component C8 gamma chain precursor | C8G |
| AIEDYINEFSVR | IPI00022395 | Complement component C9 precursor | C9 |
| CLCACPFK | IPI00022395 | Complement component C9 precursor | C9 |
| CNGDNDCGDFSDEDDCESEPRPPCR | IPI00022395 | Complement component C9 precursor | C9 |
| DVVLTTTFVDDIK | IPI00022395 | Complement component C9 precursor | C9 |
| FEGIACEISK | IPI00022395 | Complement component C9 precursor | C9 |
| GTVIDVTDFVNWASSINDAPVLISQK | IPI00022395 | Complement component C9 precursor | C9 |
| MFLHVK | IPI00022395 | Complement component C9 precursor | C9 |
| QCVPTEPCEDAEDDCGNDFQCSTGR | IPI00022395 | Complement component C9 precursor | C9 |
| RPWNVASLIYETK | IPI00022395 | Complement component C9 precursor | C9 |
| SIEVFGQFNGK | IPI00022395 | Complement component C9 precursor | C9 |
| SIEVFGQFNGKR | IPI00022395 | Complement component C9 precursor | C9 |
| TAGYGINILGMDPLSTPFDNEFYNGLCNR | IPI00022395 | Complement component C9 precursor | C9 |
| TSNFNAAISLK | IPI00022395 | Complement component C9 precursor | C9 |
| VVEESELAR | IPI00022395 | Complement component C9 precursor | C9 |
| ADGLAVIGVLMK | IPI00788926, IPI00215983, IPI00798267, IPI00796435 | Carbonic anhydrase 1 | CA1 |
| EIINVGHSFHVNFEDNDNR | IPI00215983 | Carbonic anhydrase 1 | CA1 |
| ESISVSSEQLAQFR | IPI00788926, IPI00215983, IPI00798267, IPI00796435 | Carbonic anhydrase 1 | CA1 |
| LYPIANGNNQSPVDIK | IPI00215983 | Carbonic anhydrase 1 | CA1 |
| SLLSNVEGDNAVPMQHNNRPTQPLK | IPI00788926, IPI00215983, IPI00798267, IPI00796435 | Carbonic anhydrase 1 | CA1 |
| VLDALQAIK | IPI00788926, IPI00215983, IPI00798267, IPI00796435 | Carbonic anhydrase 1 | CA1 |
| AVQQPDGLAVLGIFLK | IPI00218414 | Carbonic anhydrase 2 | CA2 |
| EQFLDGDGWTSR | IPI00383751, IPI00020599 | Calreticulin | CALR |
| IKDPDASKPEDWDER | IPI00020599 | Calreticulin precursor | CALR |
| ADVLTTGAGNPVGDKLNVITVGPR | IPI00465436 | Catalase | CAT |
| DAQIFIQK | IPI00465436 | Catalase | CAT |
| FNTANDDNVTQVR | IPI00465436 | Catalase | CAT |
| FSTVAGESGSADTVR | IPI00465436 | Catalase | CAT |
| FYTEDGNWDLVGNNTPIFFIR | IPI00465436 | Catalase | CAT |
| LFAYPDTHR | IPI00465436 | Catalase | CAT |
| LGPNYLHIPVNCPYR | IPI00465436 | Catalase | CAT |
| LNVITVGPR | IPI00465436 | Catalase | CAT |
| MASWGGEK | IPI00793233, IPI00479279, IPI00792540, IPI00334492, IPI00791244, IPI00876839, IPI00295898, IPI00939592, IPI00242921, IPI00791896, IPI00893813 | Putative coiled-coil domain-containing protein 144 N-terminal-like | CCD144 |
| EDPLFIIR | IPI00015618, IPI00792575 | cDNA FLJ55385 | CCDC49 |
| EVCTNPNDDWVQEYIKDPNLPLLPTR | IPI00006717 | C-C motif chemokine 16 precursor | CCL16 |
| VQITAIGDVLGPSINGLASLIR | IPI00152540, IPI00795801, IPI00788676 | Isoform 1 of CD109 antigen precursor | CD109 |
| AFPALTSLDLSDNPGLGER | IPI00029260 | Monocyte differentiation antigen CD14 precursor | CD14 |
| FPAIQNLALR | IPI00029260 | Monocyte differentiation antigen CD14 precursor | CD14 |
| GLMAALCPHK | IPI00029260 | Monocyte differentiation antigen CD14 precursor | CD14 |
| GLMAALCPHKFPAIQNLALR | IPI00029260 | Monocyte differentiation antigen CD14 precursor | CD14 |
| ITGTMPPLPLEATGLALSSLR | IPI00029260 | Monocyte differentiation antigen CD14 precursor | CD14 |
| LKELTLEDLK | IPI00029260 | Monocyte differentiation antigen CD14 precursor | CD14 |
| LTVGAAQVPAQLLVGALR | IPI00029260 | Monocyte differentiation antigen CD14 precursor | CD14 |
| QYADTVK | IPI00029260 | Monocyte differentiation antigen CD14 precursor | CD14 |
| RLTVGAAQVPAQLLVGALR | IPI00029260 | Monocyte differentiation antigen CD14 precursor | CD14 |
| STLSVGVSGTLVLLQGAR | IPI00029260 | Monocyte differentiation antigen CD14 precursor | CD14 |
| SWLAELQQWLKPGLK | IPI00029260 | Monocyte differentiation antigen CD14 precursor | CD14 |
| VLDLSCNR | IPI00029260 | Monocyte differentiation antigen CD14 precursor | CD14 |
| VLSIAQAHSPAFSCEQVR | IPI00029260 | Monocyte differentiation antigen CD14 precursor | CD14 |
| CAGTVEVEIQR | IPI00759525, IPI00759642, IPI00104074, IPI00513892 | Isoform 1 of Scavenger receptor cysteine-rich type 1 protein M130 precursor | CD163 |
| LLWIGLQR | IPI00006971 | Isoform 1 of Endosialin precursor | CD248 |
| ALSIGFETCR | IPI00827658, IPI00827893, IPI00922478, IPI00827982, IPI00827555, IPI00828056, IPI00827650, IPI00918020, IPI00827795, IPI00305064, IPI00297160, IPI00827937, IPI00828192, IPI00828108, IPI00828064, IPI00418465, IPI00419219, IPI00828117 | CD44 | CD44 |
| YGFIEGHVVIPR | IPI00827658, IPI00827893, IPI00922478, IPI00827982, IPI00827555, IPI00828056, IPI00827650, IPI00918020, IPI00827795, IPI00305064, IPI00297160, IPI00827937, IPI00828192, IPI00828108, IPI00828064, IPI00418465, IPI00419219, IPI00828117 | CD44 | CD44 |
| CSGEEQSLEQCQHR | IPI00025204 | CD5 antigen-like precursor | CD5L |
| ELGCGAASGTPSGILYEPPAEK | IPI00025204 | CD5 antigen-like precursor | CD5L |
| FWGFHDCTHQEDVAVICSG | IPI00025204 | CD5 antigen-like precursor | CD5L |
| GQWGTVCDDGWDIK | IPI00025204 | CD5 antigen-like precursor | CD5L |
| GQWGTVCDDGWDIKDVAVLCR | IPI00025204 | CD5 antigen-like precursor | CD5L |
| GVWGSVCDDNWGEK | IPI00025204 | CD5 antigen-like precursor | CD5L |
| GVWGSVCDDNWGEKEDQVVCK | IPI00025204 | CD5 antigen-like precursor | CD5L |
| HQNQWYTVCQTGWSLR | IPI00025204 | CD5 antigen-like precursor | CD5L |
| IWLDNVR | IPI00025204 | CD5 antigen-like precursor | CD5L |
| KPIWLSQMSCSGR | IPI00025204 | CD5 antigen-like precursor | CD5L |
| LEVLHK | IPI00025204 | CD5 antigen-like precursor | CD5L |
| LVGGDNLCSGR | IPI00025204 | CD5 antigen-like precursor | CD5L |
| NTCNHDEDTWVECEDPFDLR | IPI00025204 | CD5 antigen-like precursor | CD5L |
| ENETLIQR | IPI00300659 | Parafibromin | CDC73 |
| DQVTTLEVSVCDCEGAAGVCR | IPI00911003, IPI00025861, IPI00000513, IPI00744889 | Cadherin-1 | CDH1 |
| DTANWLEINPDTGAISTR | IPI00911003, IPI00025861, IPI00000513, IPI00744889 | Cadherin-1 | CDH1 |
| NTGVISVVTTGLDR | IPI00911003, IPI00025861, IPI00000513, IPI00744889 | Cadherin-1 | CDH1 |
| VFYSITGQGADTPPVGVFIIER | IPI00911003, IPI00025861, IPI00000513, IPI00744889 | Cadherin-1 | CDH1 |
| EVIAVYQLFVETTDVNGK | IPI00024046 | Cadherin-13 precursor | CDH13 |
| TLEGPVPLEVIVIDQNDNRPIFR | IPI00024046 | Cadherin-13 precursor | CDH13 |
| SIVVSPILIPENQR | IPI00879347, IPI00879357, IPI00024046 | cDNA FLJ52398, highly similar to Cadherin-13 | CDH13 |
| FAIQTDPNSNDGLVTVVKPIDFETNR | IPI00853291, IPI00788754, IPI00658202, IPI00290085, IPI00789821, IPI00916622 | Cadherin-2 | CDH2 |
| FLEAGIYEVPIIITDSGNPPK | IPI00853291, IPI00788754, IPI00658202, IPI00290085, IPI00789821, IPI00916622 | Cadherin-2 | CDH2 |
| GPFPQELVR | IPI00853291, IPI00788754, IPI00658202, IPI00290085, IPI00789821, IPI00916622 | Cadherin-2 | CDH2 |
| LSDPANWLK | IPI00853291, IPI00788754, IPI00658202, IPI00290085, IPI00789821, IPI00916622 | Cadherin-2 | CDH2 |
| YSVTGPGADQPPTGIFIINPISGQLSVTKPLDR | IPI00853291, IPI00788754, IPI00658202, IPI00290085, IPI00789821, IPI00916622 | Cadherin-2 | CDH2 |
| ELDSTGTPTGK | IPI00607725, IPI00910166, IPI00012792 | Cadherin-5 | CDH5 |
| KPLIGTVLAMDPDAAR | IPI00607725, IPI00910166, IPI00012792 | Cadherin-5 | CDH5 |
| VDAETGDVFAIER | IPI00607725, IPI00910166, IPI00012792 | Cadherin-5 | CDH5 |
| VHFLPVVISDNGMPSR | IPI00607725, IPI00910166, IPI00012792 | Cadherin-5 | CDH5 |
| YTFVVPEDTR | IPI00607725, IPI00910166, IPI00012792 | Cadherin-5 | CDH5 |
| LAYILQIR | IPI00217314, IPI00024035 | Isoform 1 of Cadherin-6 precursor | CDH6 |
| GAALHLHDIGIVTMDWLVR | IPI00303071, IPI00852630, IPI00252768, IPI00748521 | cat eye syndrome critical region protein 1 isoform b | CECR1 |
| LPYFFHAGETDWQGTSIDR | IPI00303071, IPI00852630, IPI00252768, IPI00748521 | cat eye syndrome critical region protein 1 isoform b | CECR1 |
| WILLEDYR | IPI00303071 | Cat eye syndrome critical region protein 1 precursor | CECR1 |
| TEFQQVLSNR | IPI00296365, IPI00868715, IPI00619925 | Isoform 1 of Centromere-associated protein E | CENPE |
| TVIGDHGDELFSVFGAPFLK | IPI00607693, IPI00940556, IPI00010180, IPI00939157, IPI00942507, IPI00514878, IPI00607801 | Isoform 1 of Liver carboxylesterase 1 | CES1 |
| EINVISNIMADFVQTR | IPI00641481, IPI00006173 | Isoform 1 of Cholesteryl ester transfer protein precursor | CETP |
| GVSLFDIINPEIITR | IPI00641481, IPI00006173 | Isoform 1 of Cholesteryl ester transfer protein precursor | CETP |
| ALFVSEEEK | IPI00643506, IPI00939824, IPI00939713, IPI00893864, IPI00942200, IPI00218508, IPI00893812, IPI00303963, IPI00916995, IPI00910539, IPI00910028, IPI00514172, IPI00939333, IPI00019591, IPI00515098, IPI00791030, IPI00645500, IPI00921523, IPI00943098, IPI00514968, IPI00942221 | Complement factor B | CFB |
| ALHQVFEHMLDVSK | IPI00643506, IPI00939824, IPI00939713, IPI00893864, IPI00942200, IPI00218508, IPI00893812, IPI00303963, IPI00916995, IPI00910539, IPI00910028, IPI00514172, IPI00939333, IPI00019591, IPI00515098, IPI00791030, IPI00645500, IPI00921523, IPI00943098, IPI00514968, IPI00942221 | Complement factor B | CFB |
| AVISPGFDVFAK | IPI00643506, IPI00939824, IPI00939713, IPI00893864, IPI00942200, IPI00218508, IPI00893812, IPI00303963, IPI00916995, IPI00910539, IPI00910028, IPI00514172, IPI00939333, IPI00019591, IPI00515098, IPI00791030, IPI00645500, IPI00921523, IPI00943098, IPI00514968, IPI00942221 | Complement factor B | CFB |
| CLVNLIEK | IPI00643506, IPI00939824, IPI00939713, IPI00893864, IPI00942200, IPI00218508, IPI00893812, IPI00303963, IPI00916995, IPI00910539, IPI00910028, IPI00514172, IPI00939333, IPI00019591, IPI00515098, IPI00791030, IPI00645500, IPI00921523, IPI00943098, IPI00514968, IPI00942221 | Complement factor B | CFB |
| CPAPVSFENGIYTPR | IPI00643506, IPI00939824, IPI00939713, IPI00893864, IPI00942200, IPI00218508, IPI00893812, IPI00303963, IPI00916995, IPI00910539, IPI00910028, IPI00514172, IPI00939333, IPI00019591, IPI00515098, IPI00791030, IPI00645500, IPI00921523, IPI00943098, IPI00514968, IPI00942221 | Complement factor B | CFB |
| CSSNLVLTGSSER | IPI00643506, IPI00939824, IPI00939713, IPI00893864, IPI00942200, IPI00218508, IPI00893812, IPI00303963, IPI00916995, IPI00910539, IPI00910028, IPI00514172, IPI00939333, IPI00019591, IPI00515098, IPI00791030, IPI00645500, IPI00921523, IPI00943098, IPI00514968, IPI00942221 | Complement factor B | CFB |
| DAQYAPGYDK | IPI00643506, IPI00939824, IPI00939713, IPI00893864, IPI00942200, IPI00218508, IPI00893812, IPI00303963, IPI00916995, IPI00910539, IPI00910028, IPI00514172, IPI00939333, IPI00019591, IPI00515098, IPI00791030, IPI00645500, IPI00921523, IPI00943098, IPI00514968, IPI00942221 | Complement factor B | CFB |
| DFHINLFQVLPWLK | IPI00643506, IPI00939824, IPI00939713, IPI00893864, IPI00942200, IPI00218508, IPI00893812, IPI00303963, IPI00916995, IPI00910539, IPI00910028, IPI00514172, IPI00939333, IPI00019591, IPI00515098, IPI00791030, IPI00645500, IPI00921523, IPI00943098, IPI00514968, IPI00942221 | Complement factor B | CFB |
| DFHINLFR | IPI00643506, IPI00939824, IPI00939713, IPI00893864, IPI00942200, IPI00218508, IPI00893812, IPI00303963, IPI00916995, IPI00910539, IPI00910028, IPI00514172, IPI00939333, IPI00019591, IPI00515098, IPI00791030, IPI00645500, IPI00921523, IPI00943098, IPI00514968, IPI00942221 | Complement factor B | CFB |
| DGNDHSLWR | IPI00643506, IPI00939824, IPI00939713, IPI00893864, IPI00942200, IPI00218508, IPI00893812, IPI00303963, IPI00916995, IPI00910539, IPI00910028, IPI00514172, IPI00939333, IPI00019591, IPI00515098, IPI00791030, IPI00645500, IPI00921523, IPI00943098, IPI00514968, IPI00942221 | Complement factor B | CFB |
| DHENELLNK | IPI00643506, IPI00939824, IPI00939713, IPI00893864, IPI00942200, IPI00218508, IPI00893812, IPI00303963, IPI00916995, IPI00910539, IPI00910028, IPI00514172, IPI00939333, IPI00019591, IPI00515098, IPI00791030, IPI00645500, IPI00921523, IPI00943098, IPI00514968, IPI00942221 | Complement factor B | CFB |
| DISEVVTPR | IPI00643506, IPI00939824, IPI00939713, IPI00893864, IPI00942200, IPI00218508, IPI00893812, IPI00303963, IPI00916995, IPI00910539, IPI00910028, IPI00514172, IPI00939333, IPI00019591, IPI00515098, IPI00791030, IPI00645500, IPI00921523, IPI00943098, IPI00514968, IPI00942221 | Complement factor B | CFB |
| DLEIEVVLFHPNYNINGK | IPI00643506, IPI00939824, IPI00939713, IPI00893864, IPI00942200, IPI00218508, IPI00893812, IPI00303963, IPI00916995, IPI00910539, IPI00910028, IPI00514172, IPI00939333, IPI00019591, IPI00515098, IPI00791030, IPI00645500, IPI00921523, IPI00943098, IPI00514968, IPI00942221 | Complement factor B | CFB |
| DMTEVISSLENANYK | IPI00643506, IPI00939824, IPI00939713, IPI00893864, IPI00942200, IPI00218508, IPI00893812, IPI00303963, IPI00916995, IPI00910539, IPI00910028, IPI00514172, IPI00939333, IPI00019591, IPI00515098, IPI00791030, IPI00645500, IPI00921523, IPI00943098, IPI00514968, IPI00942221 | Complement factor B | CFB |
| EAGIPEFYDYDVALIK | IPI00643506, IPI00939824, IPI00939713, IPI00893864, IPI00942200, IPI00218508, IPI00893812, IPI00303963, IPI00916995, IPI00910539, IPI00910028, IPI00514172, IPI00939333, IPI00019591, IPI00515098, IPI00791030, IPI00645500, IPI00921523, IPI00943098, IPI00514968, IPI00942221 | Complement factor B | CFB |
| ECQGNGVWSGTEPICR | IPI00643506, IPI00939824, IPI00939713, IPI00893864, IPI00942200, IPI00218508, IPI00893812, IPI00303963, IPI00916995, IPI00910539, IPI00910028, IPI00514172, IPI00939333, IPI00019591, IPI00515098, IPI00791030, IPI00645500, IPI00921523, IPI00943098, IPI00514968, IPI00942221 | Complement factor B | CFB |
| EDYLDVYVFGVGPLVNQVNINALASK | IPI00643506, IPI00939824, IPI00939713, IPI00893864, IPI00942200, IPI00218508, IPI00893812, IPI00303963, IPI00916995, IPI00910539, IPI00910028, IPI00514172, IPI00939333, IPI00019591, IPI00515098, IPI00791030, IPI00645500, IPI00921523, IPI00943098, IPI00514968, IPI00942221 | Complement factor B | CFB |
| EILNINQK | IPI00643506, IPI00939824, IPI00939713, IPI00893864, IPI00942200, IPI00218508, IPI00893812, IPI00303963, IPI00916995, IPI00910539, IPI00910028, IPI00514172, IPI00939333, IPI00019591, IPI00515098, IPI00791030, IPI00645500, IPI00921523, IPI00943098, IPI00514968, IPI00942221 | Complement factor B | CFB |
| EKLQDEDLGFL | IPI00643506, IPI00939824, IPI00939713, IPI00893864, IPI00942200, IPI00218508, IPI00893812, IPI00303963, IPI00916995, IPI00910539, IPI00910028, IPI00514172, IPI00939333, IPI00019591, IPI00515098, IPI00791030, IPI00645500, IPI00921523, IPI00943098, IPI00514968, IPI00942221 | Complement factor B | CFB |
| ELNELGSK | IPI00643506, IPI00939824, IPI00939713, IPI00893864, IPI00942200, IPI00218508, IPI00893812, IPI00303963, IPI00916995, IPI00910539, IPI00910028, IPI00514172, IPI00939333, IPI00019591, IPI00515098, IPI00791030, IPI00645500, IPI00921523, IPI00943098, IPI00514968, IPI00942221 | Complement factor B | CFB |
| ESASLMVDR | IPI00643506, IPI00939824, IPI00939713, IPI00893864, IPI00942200, IPI00218508, IPI00893812, IPI00303963, IPI00916995, IPI00910539, IPI00910028, IPI00514172, IPI00939333, IPI00019591, IPI00515098, IPI00791030, IPI00645500, IPI00921523, IPI00943098, IPI00514968, IPI00942221 | Complement factor B | CFB |
| EVVTDQFLCSGTQEDESPCK | IPI00643506, IPI00939824, IPI00939713, IPI00893864, IPI00942200, IPI00218508, IPI00893812, IPI00303963, IPI00916995, IPI00910539, IPI00910028, IPI00514172, IPI00939333, IPI00019591, IPI00515098, IPI00791030, IPI00645500, IPI00921523, IPI00943098, IPI00514968, IPI00942221 | Complement factor B | CFB |
| EVVTDQFLCSGTQEDESPCKGESGGAVFLER | IPI00643506, IPI00939824, IPI00939713, IPI00893864, IPI00942200, IPI00218508, IPI00893812, IPI00303963, IPI00916995, IPI00910539, IPI00910028, IPI00514172, IPI00939333, IPI00019591, IPI00515098, IPI00791030, IPI00645500, IPI00921523, IPI00943098, IPI00514968, IPI00942221 | Complement factor B | CFB |
| FFQVGLVSWGLYNPCLGSADK | IPI00643506, IPI00939824, IPI00939713, IPI00893864, IPI00942200, IPI00218508, IPI00893812, IPI00303963, IPI00916995, IPI00910539, IPI00910028, IPI00514172, IPI00939333, IPI00019591, IPI00515098, IPI00791030, IPI00645500, IPI00921523, IPI00943098, IPI00514968, IPI00942221 | Complement factor B | CFB |
| FLCTGGVSPYADPNTCR | IPI00643506, IPI00939824, IPI00939713, IPI00893864, IPI00942200, IPI00218508, IPI00893812, IPI00303963, IPI00916995, IPI00910539, IPI00910028, IPI00514172, IPI00939333, IPI00019591, IPI00515098, IPI00791030, IPI00645500, IPI00921523, IPI00943098, IPI00514968, IPI00942221 | Complement factor B | CFB |
| GALISDQWVLTAAHCFR | IPI00643506, IPI00939824, IPI00939713, IPI00893864, IPI00942200, IPI00218508, IPI00893812, IPI00303963, IPI00916995, IPI00910539, IPI00910028, IPI00514172, IPI00939333, IPI00019591, IPI00515098, IPI00791030, IPI00645500, IPI00921523, IPI00943098, IPI00514968, IPI00942221 | Complement factor B | CFB |
| GDSGGPLIVHK | IPI00643506, IPI00939824, IPI00939713, IPI00893864, IPI00942200, IPI00218508, IPI00893812, IPI00303963, IPI00916995, IPI00910539, IPI00910028, IPI00514172, IPI00939333, IPI00019591, IPI00515098, IPI00791030, IPI00645500, IPI00921523, IPI00943098, IPI00514968, IPI00942221 | Complement factor B | CFB |
| GESGGAVFLER | IPI00643506, IPI00939824, IPI00939713, IPI00893864, IPI00942200, IPI00218508, IPI00893812, IPI00303963, IPI00916995, IPI00910539, IPI00910028, IPI00514172, IPI00939333, IPI00019591, IPI00515098, IPI00791030, IPI00645500, IPI00921523, IPI00943098, IPI00514968, IPI00942221 | Complement factor B | CFB |
| HAFILQDTK | IPI00643506, IPI00939824, IPI00939713, IPI00893864, IPI00942200, IPI00218508, IPI00893812, IPI00303963, IPI00916995, IPI00910539, IPI00910028, IPI00514172, IPI00939333, IPI00019591, IPI00515098, IPI00791030, IPI00645500, IPI00921523, IPI00943098, IPI00514968, IPI00942221 | Complement factor B | CFB |
| HAIILLTDGK | IPI00643506, IPI00939824, IPI00939713, IPI00893864, IPI00942200, IPI00218508, IPI00893812, IPI00303963, IPI00916995, IPI00910539, IPI00910028, IPI00514172, IPI00939333, IPI00019591, IPI00515098, IPI00791030, IPI00645500, IPI00921523, IPI00943098, IPI00514968, IPI00942221 | Complement factor B | CFB |
| HVIILMTDGLHNMGGDPITVIDEIR | IPI00643506, IPI00939824, IPI00939713, IPI00893864, IPI00942200, IPI00218508, IPI00893812, IPI00303963, IPI00916995, IPI00910539, IPI00910028, IPI00514172, IPI00939333, IPI00019591, IPI00515098, IPI00791030, IPI00645500, IPI00921523, IPI00943098, IPI00514968, IPI00942221 | Complement factor B | CFB |
| ISVIRPSK | IPI00643506, IPI00939824, IPI00939713, IPI00893864, IPI00942200, IPI00218508, IPI00893812, IPI00303963, IPI00916995, IPI00910539, IPI00910028, IPI00514172, IPI00939333, IPI00019591, IPI00515098, IPI00791030, IPI00645500, IPI00921523, IPI00943098, IPI00514968, IPI00942221 | Complement factor B | CFB |
| KNPREDYLDVYVFGVGPLVNQVNINALASK | IPI00643506, IPI00939824, IPI00939713, IPI00893864, IPI00942200, IPI00218508, IPI00893812, IPI00303963, IPI00916995, IPI00910539, IPI00910028, IPI00514172, IPI00939333, IPI00019591, IPI00515098, IPI00791030, IPI00645500, IPI00921523, IPI00943098, IPI00514968, IPI00942221 | Complement factor B | CFB |
| KNQGILEFYGDDIALLK | IPI00643506, IPI00939824, IPI00939713, IPI00893864, IPI00942200, IPI00218508, IPI00893812, IPI00303963, IPI00916995, IPI00910539, IPI00910028, IPI00514172, IPI00939333, IPI00019591, IPI00515098, IPI00791030, IPI00645500, IPI00921523, IPI00943098, IPI00514968, IPI00942221 | Complement factor B | CFB |
| LLGMETMAWQEIR | IPI00643506, IPI00939824, IPI00939713, IPI00893864, IPI00942200, IPI00218508, IPI00893812, IPI00303963, IPI00916995, IPI00910539, IPI00910028, IPI00514172, IPI00939333, IPI00019591, IPI00515098, IPI00791030, IPI00645500, IPI00921523, IPI00943098, IPI00514968, IPI00942221 | Complement factor B | CFB |
| LLQEGQALEYVCPSGFYPYPVQTR | IPI00643506, IPI00939824, IPI00939713, IPI00893864, IPI00942200, IPI00218508, IPI00893812, IPI00303963, IPI00916995, IPI00910539, IPI00910028, IPI00514172, IPI00939333, IPI00019591, IPI00515098, IPI00791030, IPI00645500, IPI00921523, IPI00943098, IPI00514968, IPI00942221 | Complement factor B | CFB |
| LPPTTTCQQQK | IPI00643506, IPI00939824, IPI00939713, IPI00893864, IPI00942200, IPI00218508, IPI00893812, IPI00303963, IPI00916995, IPI00910539, IPI00910028, IPI00514172, IPI00939333, IPI00019591, IPI00515098, IPI00791030, IPI00645500, IPI00921523, IPI00943098, IPI00514968, IPI00942221 | Complement factor B | CFB |
| LPPTTTCQQQKEELLPAQDIK | IPI00643506, IPI00939824, IPI00939713, IPI00893864, IPI00942200, IPI00218508, IPI00893812, IPI00303963, IPI00916995, IPI00910539, IPI00910028, IPI00514172, IPI00939333, IPI00019591, IPI00515098, IPI00791030, IPI00645500, IPI00921523, IPI00943098, IPI00514968, IPI00942221 | Complement factor B | CFB |
| MGVEWTSCAEVVSQEK | IPI00643506, IPI00939824, IPI00939713, IPI00893864, IPI00942200, IPI00218508, IPI00893812, IPI00303963, IPI00916995, IPI00910539, IPI00910028, IPI00514172, IPI00939333, IPI00019591, IPI00515098, IPI00791030, IPI00645500, IPI00921523, IPI00943098, IPI00514968, IPI00942221 | Complement factor B | CFB |
| NDYLDIYAIGVGK | IPI00643506, IPI00939824, IPI00939713, IPI00893864, IPI00942200, IPI00218508, IPI00893812, IPI00303963, IPI00916995, IPI00910539, IPI00910028, IPI00514172, IPI00939333, IPI00019591, IPI00515098, IPI00791030, IPI00645500, IPI00921523, IPI00943098, IPI00514968, IPI00942221 | Complement factor B | CFB |
| NQGILEFYGDDIALLK | IPI00643506, IPI00939824, IPI00939713, IPI00893864, IPI00942200, IPI00218508, IPI00893812, IPI00303963, IPI00916995, IPI00910539, IPI00910028, IPI00514172, IPI00939333, IPI00019591, IPI00515098, IPI00791030, IPI00645500, IPI00921523, IPI00943098, IPI00514968, IPI00942221 | Complement factor B | CFB |
| QCRPNGMWDGETAVCDNGAGHCPNPGISLGAVR | IPI00643506, IPI00939824, IPI00939713, IPI00893864, IPI00942200, IPI00218508, IPI00893812, IPI00303963, IPI00916995, IPI00910539, IPI00910028, IPI00514172, IPI00939333, IPI00019591, IPI00515098, IPI00791030, IPI00645500, IPI00921523, IPI00943098, IPI00514968, IPI00942221 | Complement factor B | CFB |
| QHLGDVLNFLPL | IPI00643506, IPI00939824, IPI00939713, IPI00893864, IPI00942200, IPI00218508, IPI00893812, IPI00303963, IPI00916995, IPI00910539, IPI00910028, IPI00514172, IPI00939333, IPI00019591, IPI00515098, IPI00791030, IPI00645500, IPI00921523, IPI00943098, IPI00514968, IPI00942221 | Complement factor B | CFB |
| QLNEINYEDHK | IPI00643506, IPI00939824, IPI00939713, IPI00893864, IPI00942200, IPI00218508, IPI00893812, IPI00303963, IPI00916995, IPI00910539, IPI00910028, IPI00514172, IPI00939333, IPI00019591, IPI00515098, IPI00791030, IPI00645500, IPI00921523, IPI00943098, IPI00514968, IPI00942221 | Complement factor B | CFB |
| QPYSYDFPEDVAPALGTSFSHMLGATNPTQK | IPI00643506, IPI00939824, IPI00939713, IPI00893864, IPI00942200, IPI00218508, IPI00893812, IPI00303963, IPI00916995, IPI00910539, IPI00910028, IPI00514172, IPI00939333, IPI00019591, IPI00515098, IPI00791030, IPI00645500, IPI00921523, IPI00943098, IPI00514968, IPI00942221 | Complement factor B | CFB |
| RNDYLDIYAIGVGK | IPI00643506, IPI00939824, IPI00939713, IPI00893864, IPI00942200, IPI00218508, IPI00893812, IPI00303963, IPI00916995, IPI00910539, IPI00910028, IPI00514172, IPI00939333, IPI00019591, IPI00515098, IPI00791030, IPI00645500, IPI00921523, IPI00943098, IPI00514968, IPI00942221 | Complement factor B | CFB |
| SSGQWQTPGATR | IPI00643506, IPI00939824, IPI00939713, IPI00893864, IPI00942200, IPI00218508, IPI00893812, IPI00303963, IPI00916995, IPI00910539, IPI00910028, IPI00514172, IPI00939333, IPI00019591, IPI00515098, IPI00791030, IPI00645500, IPI00921523, IPI00943098, IPI00514968, IPI00942221 | Complement factor B | CFB |
| STGSWSTLK | IPI00643506, IPI00939824, IPI00939713, IPI00893864, IPI00942200, IPI00218508, IPI00893812, IPI00303963, IPI00916995, IPI00910539, IPI00910028, IPI00514172, IPI00939333, IPI00019591, IPI00515098, IPI00791030, IPI00645500, IPI00921523, IPI00943098, IPI00514968, IPI00942221 | Complement factor B | CFB |
| TPWHVTIKPK | IPI00643506, IPI00939824, IPI00939713, IPI00893864, IPI00942200, IPI00218508, IPI00893812, IPI00303963, IPI00916995, IPI00910539, IPI00910028, IPI00514172, IPI00939333, IPI00019591, IPI00515098, IPI00791030, IPI00645500, IPI00921523, IPI00943098, IPI00514968, IPI00942221 | Complement factor B | CFB |
| VKDISEVVTPR | IPI00643506, IPI00939824, IPI00939713, IPI00893864, IPI00942200, IPI00218508, IPI00893812, IPI00303963, IPI00916995, IPI00910539, IPI00910028, IPI00514172, IPI00939333, IPI00019591, IPI00515098, IPI00791030, IPI00645500, IPI00921523, IPI00943098, IPI00514968, IPI00942221 | Complement factor B | CFB |
| VLMSVLNDNSR | IPI00643506, IPI00939824, IPI00939713, IPI00893864, IPI00942200, IPI00218508, IPI00893812, IPI00303963, IPI00916995, IPI00910539, IPI00910028, IPI00514172, IPI00939333, IPI00019591, IPI00515098, IPI00791030, IPI00645500, IPI00921523, IPI00943098, IPI00514968, IPI00942221 | Complement factor B | CFB |
| WSGQTAICDNGAGYCSNPGIPIGTR | IPI00643506, IPI00939824, IPI00939713, IPI00893864, IPI00942200, IPI00218508, IPI00893812, IPI00303963, IPI00916995, IPI00910539, IPI00910028, IPI00514172, IPI00939333, IPI00019591, IPI00515098, IPI00791030, IPI00645500, IPI00921523, IPI00943098, IPI00514968, IPI00942221 | Complement factor B | CFB |
| YGLVTYATYPK | IPI00643506, IPI00939824, IPI00939713, IPI00893864, IPI00942200, IPI00218508, IPI00893812, IPI00303963, IPI00916995, IPI00910539, IPI00910028, IPI00514172, IPI00939333, IPI00019591, IPI00515098, IPI00791030, IPI00645500, IPI00921523, IPI00943098, IPI00514968, IPI00942221 | Complement factor B | CFB |
| YGQTIRPICLPCTEGTTR | IPI00643506, IPI00939824, IPI00939713, IPI00893864, IPI00942200, IPI00218508, IPI00893812, IPI00303963, IPI00916995, IPI00910539, IPI00910028, IPI00514172, IPI00939333, IPI00019591, IPI00515098, IPI00791030, IPI00645500, IPI00921523, IPI00943098, IPI00514968, IPI00942221 | Complement factor B | CFB |
| ACDGINDCGDQSDELCCK | IPI00291867, IPI00935408, IPI00796990 | Complement factor I | CFI |
| ADSPMDDFFQCVNGK | IPI00291867, IPI00935408, IPI00796990 | Complement factor I | CFI |
| AQLGDLPWQVAIK | IPI00291867, IPI00935408, IPI00796990 | Complement factor I | CFI |
| DASGITCGGIYIGGCWILTAAHCLR | IPI00291867, IPI00935408, IPI00796990 | Complement factor I | CFI |
| EANVACLDLGFQQGADTQR | IPI00291867, IPI00935408, IPI00796990 | Complement factor I | CFI |
| EMECAGTYDGSIDACK | IPI00291867, IPI00935408, IPI00796990 | Complement factor I | CFI |
| GLETSLAECTFTK | IPI00291867, IPI00935408, IPI00796990 | Complement factor I | CFI |
| HGNTDSEGIVEVK | IPI00291867, IPI00935408, IPI00796990 | Complement factor I | CFI |
| IIFHENYNAGTYQNDIALIEMK | IPI00291867, IPI00935408, IPI00796990 | Complement factor I | CFI |
| SFPTYCQQK | IPI00291867, IPI00935408, IPI00796990 | Complement factor I | CFI |
| SLECLHPGTK | IPI00291867, IPI00935408, IPI00796990 | Complement factor I | CFI |
| TMGYQDFADVVCYTQK | IPI00291867, IPI00935408, IPI00796990 | Complement factor I | CFI |
| VANYFDWISYHVGRPFISQYNV | IPI00291867, IPI00935408, IPI00796990 | Complement factor I | CFI |
| VFCQPWQR | IPI00291867, IPI00935408, IPI00796990 | Complement factor I | CFI |
| VFSLQWGEVK | IPI00291867, IPI00935408, IPI00796990 | Complement factor I | CFI |
| YQIWTTVVDWIHPDLK | IPI00291867, IPI00935408, IPI00796990 | Complement factor I | CFI |
| YQIWTTVVDWIHPDLKR | IPI00291867, IPI00935408, IPI00796990 | Complement factor I | CFI |
| RPEDQELESLSAIEAELEK | IPI00290315, IPI00383975 | Chromogranin-A | CHGA |
| AEGNNQAPGEEEEEEEEATNTHPPASLPSQK | IPI00290315 | Chromogranin-A precursor | CHGA |
| EAGTLAYYEICDFLR | IPI00002147 | Chitinase-3-like protein 1 precursor | CHI3L1 |
| LVMGIPTFGR | IPI00002147 | Chitinase-3-like protein 1 precursor | CHI3L1 |
| CTASNFLGTATHDFHVIVEEPPR | IPI00299059, IPI00924657, IPI00927924, IPI00925181, IPI00924980, IPI00783390, IPI00794030, IPI00927259 | Isoform 2 of Neural cell adhesion molecule L1-like protein | CHL1 |
| GLPPLHIYWMNIELEHIEQDER | IPI00299059, IPI00924657, IPI00927924, IPI00925181, IPI00924980, IPI00783390, IPI00794030, IPI00927259 | Isoform 2 of Neural cell adhesion molecule L1-like protein | CHL1 |
| GYQINWWK | IPI00299059, IPI00924657, IPI00927924, IPI00925181, IPI00924980, IPI00783390, IPI00794030, IPI00927259 | Isoform 2 of Neural cell adhesion molecule L1-like protein | CHL1 |
| KTTVILPLAPFVR | IPI00299059, IPI00924657, IPI00927924, IPI00925181, IPI00924980, IPI00783390, IPI00794030, IPI00927259 | Isoform 2 of Neural cell adhesion molecule L1-like protein | CHL1 |
| TTVILPLAPFVR | IPI00299059, IPI00924657, IPI00927924, IPI00925181, IPI00924980, IPI00783390, IPI00794030, IPI00927259 | Isoform 2 of Neural cell adhesion molecule L1-like protein | CHL1 |
| VNGSPVDNHPFAGDVVFPR | IPI00299059, IPI00924657, IPI00927924, IPI00925181, IPI00924980, IPI00783390, IPI00794030, IPI00927259 | Isoform 2 of Neural cell adhesion molecule L1-like protein | CHL1 |
| VQVAFPFDEYFQIECEAK | IPI00299059, IPI00924657, IPI00927924, IPI00925181, IPI00924980, IPI00783390, IPI00794030, IPI00927259 | Isoform 2 of Neural cell adhesion molecule L1-like protein | CHL1 |
| QSAFQYLQSTPAQSPAAGTVQGR | IPI00791803, IPI00289275 | Cartilage intermediate layer protein 1 precursor | CILP |
| EGTPASWTGDLLAWWPNPQEFR | IPI00216780 | Cartilage intermediate layer protein 2 precursor | CILP2 |
| LLESPATALGDIR | IPI00216780 | Cartilage intermediate layer protein 2 precursor | CILP2 |
| GTGGVDTAAVGSVFDVSNADR | IPI00908787, IPI00027487, IPI00909684 | Creatine kinase M-type | CKM |
| CFLAFTQTK | IPI00791350, IPI00009028, IPI00792115 | Tetranectin precursor | CLEC3B |
| EQQALQTVCLK | IPI00791350, IPI00009028, IPI00792115 | Tetranectin precursor | CLEC3B |
| GGTLSTPQTGSENDALYEYLR | IPI00009028 | Tetranectin precursor | CLEC3B |
| KDVVNTK | IPI00009028 | Tetranectin precursor | CLEC3B |
| LDTLAQEVALLK | IPI00791350, IPI00009028, IPI00792115 | Tetranectin precursor | CLEC3B |
| NWETEITAQPDGGK | IPI00791350, IPI00009028, IPI00792115 | Tetranectin precursor | CLEC3B |
| NWETEITAQPDGGKTENCAVLSGAANGK | IPI00791350, IPI00009028, IPI00792115 | Tetranectin precursor | CLEC3B |
| QSVGNEAEIWLGLNDMAAEGTWVDMTGAR | IPI00791350, IPI00009028, IPI00792115 | Tetranectin precursor | CLEC3B |
| TENCAVLSGAANGK | IPI00791350, IPI00009028, IPI00792115 | Tetranectin precursor | CLEC3B |
| TFHEASEDCISR | IPI00791350, IPI00009028, IPI00792115 | Tetranectin precursor | CLEC3B |
| LQEIYQELTR | IPI00045017, IPI00943042, IPI00410204, IPI00922313, IPI00940396, IPI00410004, IPI00290006, IPI00003402, IPI00410005, IPI00045033, IPI00874038, IPI00382671, IPI00642143, IPI00941934, IPI00290007, IPI00922589, IPI00410295, IPI00385903, IPI00410201, IPI00410294, IPI00942542, IPI00748018, IPI00382673, IPI00409772, IPI00872805, IPI00410289, IPI00410009, IPI00465229, IPI00290003, IPI00156091 | Isoform 9 of C-type lectin domain family 4 member M | CLEC4M |
| AASEFESSEGVFLFPELR | IPI00644294, IPI00007257, IPI00413959 | Calsyntenin-1 precursor | CLSTN1 |
| QFPTPGIR | IPI00644294, IPI00007257, IPI00413959 | Calsyntenin-1 precursor | CLSTN1 |
| ASSIIDELFQDR | IPI00793848, IPI00795633, IPI00400826, IPI00291262 | Clusterin precursor | CLU |
| ASSIIDELFQDRFFTR | IPI00793848, IPI00795633, IPI00400826, IPI00291262 | Clusterin precursor | CLU |
| EILSVDCSTNNPSQAK | IPI00793848, IPI00795633, IPI00400826, IPI00291262 | Clusterin precursor | CLU |
| EIQNAVNGVK | IPI00793848, IPI00795633, IPI00400826, IPI00291262 | Clusterin precursor | CLU |
| ELDESLQVAER | IPI00793848, IPI00795633, IPI00400826, IPI00291262 | Clusterin precursor | CLU |
| EPQDTYHYLPFSLPHR | IPI00793848, IPI00795633, IPI00400826, IPI00291262 | Clusterin precursor | CLU |
| FMETVAEK | IPI00793848, IPI00795633, IPI00400826, IPI00291262 | Clusterin precursor | CLU |
| IDSLLENDR | IPI00793848, IPI00795633, IPI00400826, IPI00291262 | Clusterin precursor | CLU |
| KTLLSNLEEAK | IPI00793848, IPI00795633, IPI00400826, IPI00291262 | Clusterin precursor | CLU |
| LANLTQGEDQYYLR | IPI00793848, IPI00795633, IPI00400826, IPI00291262 | Clusterin precursor | CLU |
| LFDSDPITVTVPVEVSR | IPI00793848, IPI00795633, IPI00400826, IPI00291262 | Clusterin precursor | CLU |
| QQTHMLDVMQDHFSR | IPI00793848, IPI00795633, IPI00400826, IPI00291262 | Clusterin precursor | CLU |
| RELDESLQVAER | IPI00793848, IPI00795633, IPI00400826, IPI00291262 | Clusterin precursor | CLU |
| TLLSNLEEAK | IPI00793848, IPI00795633, IPI00400826, IPI00291262 | Clusterin precursor | CLU |
| AIHLDLEEYR | IPI00749466, IPI00642045, IPI00064667 | Beta-Ala-His dipeptidase | CNDP1 |
| ALEQDLPVNIK | IPI00749466, IPI00642045, IPI00064667 | Beta-Ala-His dipeptidase | CNDP1 |
| EEILMHLWR | IPI00749466, IPI00642045, IPI00064667 | Beta-Ala-His dipeptidase | CNDP1 |
| EWVAIESDSVQPVPR | IPI00749466, IPI00642045, IPI00064667 | Beta-Ala-His dipeptidase | CNDP1 |
| FFSGVDYIVISDNLWISQR | IPI00749466, IPI00642045, IPI00064667 | Beta-Ala-His dipeptidase | CNDP1 |
| FIIEGMEEAGSVALEELVEK | IPI00749466, IPI00642045, IPI00064667 | Beta-Ala-His dipeptidase | CNDP1 |
| FLFDTKEEILMHLWR | IPI00749466, IPI00642045, IPI00064667 | Beta-Ala-His dipeptidase | CNDP1 |
| GDGWLTDPYVLTEVDGK | IPI00749466, IPI00642045, IPI00064667 | Beta-Ala-His dipeptidase | CNDP1 |
| GNSYFMVEVK | IPI00749466, IPI00642045, IPI00064667 | Beta-Ala-His dipeptidase | CNDP1 |
| GPVLAWINAVSAFR | IPI00749466, IPI00642045, IPI00064667 | Beta-Ala-His dipeptidase | CNDP1 |
| HLEDVFSK | IPI00749466, IPI00642045, IPI00064667 | Beta-Ala-His dipeptidase | CNDP1 |
| LFAAFFLEMAQLH | IPI00749466, IPI00642045, IPI00064667 | Beta-Ala-His dipeptidase | CNDP1 |
| MFQEIVHK | IPI00749466, IPI00642045, IPI00064667 | Beta-Ala-His dipeptidase | CNDP1 |
| MMAVAADTLQR | IPI00749466, IPI00642045, IPI00064667 | Beta-Ala-His dipeptidase | CNDP1 |
| MVVSMTLGLHPWIANIDDTQYLAAK | IPI00749466, IPI00642045, IPI00064667 | Beta-Ala-His dipeptidase | CNDP1 |
| SVVLIPLGAVDDGEHSQNEK | IPI00749466, IPI00642045, IPI00064667 | Beta-Ala-His dipeptidase | CNDP1 |
| TVFGTEPDMIR | IPI00749466, IPI00642045, IPI00064667 | Beta-Ala-His dipeptidase | CNDP1 |
| VFQYIDLHQDEFVQTLK | IPI00749466, IPI00642045, IPI00064667 | Beta-Ala-His dipeptidase | CNDP1 |
| WNYIEGTK | IPI00749466, IPI00642045, IPI00064667 | Beta-Ala-His dipeptidase | CNDP1 |
| AVDLIPWMEYEFR | IPI00216641, IPI00479304, IPI00029751 | Isoform 1 of Contactin-1 precursor | CNTN1 |
| FIPLIPIPER | IPI00216641, IPI00479304, IPI00029751 | Isoform 1 of Contactin-1 precursor | CNTN1 |
| GMVLLCDPPYHFPDDLSYR | IPI00216641, IPI00479304, IPI00029751 | Isoform 1 of Contactin-1 precursor | CNTN1 |
| GPPGPPGGLR | IPI00216641, IPI00479304, IPI00029751 | Isoform 1 of Contactin-1 precursor | CNTN1 |
| IFNIQLEDEGIYECEAENIR | IPI00216641, IPI00479304, IPI00029751 | Isoform 1 of Contactin-1 precursor | CNTN1 |
| IKTDGAAPNVAPSDVGGGGGR | IPI00216641, IPI00479304, IPI00029751 | Isoform 1 of Contactin-1 precursor | CNTN1 |
| STEATLSFGYLDPFPPEERPEVR | IPI00216641, IPI00479304, IPI00029751 | Isoform 1 of Contactin-1 precursor | CNTN1 |
| TDGAAPNVAPSDVGGGGGR | IPI00216641, IPI00479304, IPI00029751 | Isoform 1 of Contactin-1 precursor | CNTN1 |
| TDPPIIEGNMEAAR | IPI00216641, IPI00479304, IPI00029751 | Isoform 1 of Contactin-1 precursor | CNTN1 |
| WLLNEFPVFITMDK | IPI00216641, IPI00479304, IPI00029751 | Isoform 1 of Contactin-1 precursor | CNTN1 |
| LQFAYLENFK | IPI00292791 | Contactin-3 precursor | CNTN3 |
| TEEAVPEVPPSEVNGGGGSR | IPI00292791 | Contactin-3 precursor | CNTN3 |
| LQFAYLDNFK | IPI00178854, IPI00927871, IPI00927158, IPI00796619, IPI00479016 | Isoform 1 of Contactin-4 | CNTN4 |
| AAVPIVNLKDELLFPSWEALFSGSEGPLKPGAR | IPI00414694, IPI00022822, IPI00828004, IPI00783931, IPI00796842, IPI00479309 | Isoform 2 of Collagen alpha-1(XVIII) chain precursor | COL18A1 |
| AVGLAGTFR | IPI00414694, IPI00022822, IPI00828004, IPI00783931, IPI00796842, IPI00479309 | Isoform 2 of Collagen alpha-1(XVIII) chain precursor | COL18A1 |
| DELLFPSWEALFSGSEGPLKPGAR | IPI00414694, IPI00022822, IPI00828004, IPI00783931, IPI00796842, IPI00479309 | Isoform 2 of Collagen alpha-1(XVIII) chain precursor | COL18A1 |
| DFQPVLHLVALNSPLSGGMR | IPI00414694, IPI00022822, IPI00828004, IPI00783931, IPI00796842, IPI00479309 | Isoform 2 of Collagen alpha-1(XVIII) chain precursor | COL18A1 |
| GADFQCFQQAR | IPI00414694, IPI00022822, IPI00828004, IPI00783931, IPI00796842, IPI00479309 | Isoform 2 of Collagen alpha-1(XVIII) chain precursor | COL18A1 |
| LQDLYSIVR | IPI00414694, IPI00022822, IPI00828004, IPI00783931, IPI00796842, IPI00479309 | Isoform 2 of Collagen alpha-1(XVIII) chain precursor | COL18A1 |
| LTESYCETWR | IPI00414694, IPI00022822, IPI00828004, IPI00783931, IPI00796842, IPI00479309 | Isoform 2 of Collagen alpha-1(XVIII) chain precursor | COL18A1 |
| TEAPSATGQASSLLGGR | IPI00414694, IPI00022822, IPI00828004, IPI00783931, IPI00796842, IPI00479309 | Isoform 2 of Collagen alpha-1(XVIII) chain precursor | COL18A1 |
| NCPGAEVPEGECCPVCPDGSESPTDQETTGVEGPK | IPI00297646 | Collagen alpha-1(I) chain precursor | COL1A1 |
| VLCDDVICDETK | IPI00297646 | Collagen alpha-1(I) chain precursor | COL1A1 |
| AVILQGSNDVELVAEGNSR | IPI00909936, IPI00304962 | Collagen alpha-2(I) chain | COL1A2 |
| IALVITDGR | IPI00291136 | Collagen alpha-1(VI) chain precursor | COL6A1 |
| VFAVVITDGR | IPI00220613, IPI00073454, IPI00304840 | Isoform 2C2A' of Collagen alpha-2(VI) chain | COL6A2 |
| AELQHIATDDNLVFTVPEFR | IPI00220701, IPI00917583, IPI00941465, IPI00022200, IPI00871621, IPI00072917, IPI00072918, IPI00853061 | Isoform 1 of Collagen alpha-3(VI) chain | COL6A3 |
| ALGSAIEYTIENVFESAPNPR | IPI00220701, IPI00917583, IPI00941465, IPI00022200, IPI00871621, IPI00072917, IPI00072918, IPI00853061 | Isoform 1 of Collagen alpha-3(VI) chain | COL6A3 |
| ALNLGYALDYAQR | IPI00220701, IPI00917583, IPI00941465, IPI00022200, IPI00871621, IPI00072917, IPI00072918, IPI00853061 | Isoform 1 of Collagen alpha-3(VI) chain | COL6A3 |
| DILFLFDGSANLVGQFPVVR | IPI00220701, IPI00917583, IPI00941465, IPI00022200, IPI00871621, IPI00072917, IPI00072918, IPI00853061 | Isoform 1 of Collagen alpha-3(VI) chain | COL6A3 |
| DVVFLIDGSQSAGPEFQYVR | IPI00220701, IPI00917583, IPI00941465, IPI00022200, IPI00871621, IPI00072917, IPI00072918, IPI00853061 | Isoform 1 of Collagen alpha-3(VI) chain | COL6A3 |
| DVVFLLDGSEGVR | IPI00220701, IPI00917583, IPI00941465, IPI00022200, IPI00871621, IPI00072917, IPI00072918, IPI00853061 | Isoform 1 of Collagen alpha-3(VI) chain | COL6A3 |
| FWYGGCGGNENK | IPI00220701, IPI00917583, IPI00941465, IPI00022200, IPI00871621, IPI00072917, IPI00072918, IPI00853061 | Isoform 1 of Collagen alpha-3(VI) chain | COL6A3 |
| GADQAELEEIAFDSSLVFIPAEFR | IPI00220701, IPI00917583, IPI00941465, IPI00022200, IPI00871621, IPI00072917, IPI00072918, IPI00853061 | Isoform 1 of Collagen alpha-3(VI) chain | COL6A3 |
| IEEGVPQFLVLISSGK | IPI00220701, IPI00917583, IPI00941465, IPI00022200, IPI00871621, IPI00072917, IPI00072918, IPI00853061 | Isoform 1 of Collagen alpha-3(VI) chain | COL6A3 |
| IGDLHPQIVNLLK | IPI00220701, IPI00917583, IPI00941465, IPI00022200, IPI00871621, IPI00072917, IPI00072918, IPI00853061 | Isoform 1 of Collagen alpha-3(VI) chain | COL6A3 |
| IIDELNVKPEGTR | IPI00220701, IPI00917583, IPI00941465, IPI00022200, IPI00871621, IPI00072917, IPI00072918, IPI00853061 | Isoform 1 of Collagen alpha-3(VI) chain | COL6A3 |
| ISLSPEYVFSVSTFR | IPI00220701, IPI00917583, IPI00941465, IPI00022200, IPI00871621, IPI00072917, IPI00072918, IPI00853061 | Isoform 1 of Collagen alpha-3(VI) chain | COL6A3 |
| ITEGVPQLLIVLTADR | IPI00220701, IPI00917583, IPI00941465, IPI00022200, IPI00871621, IPI00072917, IPI00072918, IPI00853061 | Isoform 1 of Collagen alpha-3(VI) chain | COL6A3 |
| IVVLMLTGEVPEQQLEEAQR | IPI00220701, IPI00917583, IPI00941465, IPI00022200, IPI00871621, IPI00072917, IPI00072918, IPI00853061 | Isoform 1 of Collagen alpha-3(VI) chain | COL6A3 |
| LLPYIVGVAQR | IPI00220701, IPI00917583, IPI00941465, IPI00022200, IPI00871621, IPI00072917, IPI00072918, IPI00853061 | Isoform 1 of Collagen alpha-3(VI) chain | COL6A3 |
| LLTPITTLTSEQIQK | IPI00220701, IPI00917583, IPI00941465, IPI00022200, IPI00871621, IPI00072917, IPI00072918, IPI00853061 | Isoform 1 of Collagen alpha-3(VI) chain | COL6A3 |
| LLVLITGGK | IPI00220701, IPI00917583, IPI00941465, IPI00022200, IPI00871621, IPI00072917, IPI00072918, IPI00853061 | Isoform 1 of Collagen alpha-3(VI) chain | COL6A3 |
| LVDYLDVGFDTTR | IPI00220701, IPI00917583, IPI00941465, IPI00022200, IPI00871621, IPI00072917, IPI00072918, IPI00853061 | Isoform 1 of Collagen alpha-3(VI) chain | COL6A3 |
| MKPLDGSALYTGSALDFVR | IPI00220701, IPI00917583, IPI00941465, IPI00022200, IPI00871621, IPI00072917, IPI00072918, IPI00853061 | Isoform 1 of Collagen alpha-3(VI) chain | COL6A3 |
| QINVGNALEYVSR | IPI00220701, IPI00917583, IPI00941465, IPI00022200, IPI00871621, IPI00072917, IPI00072918, IPI00853061 | Isoform 1 of Collagen alpha-3(VI) chain | COL6A3 |
| QLGTVQQVISER | IPI00220701, IPI00917583, IPI00941465, IPI00022200, IPI00871621, IPI00072917, IPI00072918, IPI00853061 | Isoform 1 of Collagen alpha-3(VI) chain | COL6A3 |
| QLTLLGGPTPNTGAALEFVLR | IPI00220701, IPI00917583, IPI00941465, IPI00022200, IPI00871621, IPI00072917, IPI00072918, IPI00853061 | Isoform 1 of Collagen alpha-3(VI) chain | COL6A3 |
| QSGVVPFIFQAK | IPI00220701, IPI00917583, IPI00941465, IPI00022200, IPI00871621, IPI00072917, IPI00072918, IPI00853061 | Isoform 1 of Collagen alpha-3(VI) chain | COL6A3 |
| DSDGDGIGDACDNCPQK | IPI00028030, IPI00910249, IPI00643348, IPI00328550 | Cartilage oligomeric matrix protein | COMP |
| DTDLDGFPDEK | IPI00028030, IPI00910249, IPI00643348, IPI00328550 | Cartilage oligomeric matrix protein | COMP |
| GSFQCGPCQPGFVGDQASGCQR | IPI00028030, IPI00910249, IPI00643348, IPI00328550 | Cartilage oligomeric matrix protein | COMP |
| IDVCPENAEVTLTDFR | IPI00028030, IPI00910249, IPI00643348, IPI00328550 | Cartilage oligomeric matrix protein | COMP |
| LGVFCFSQENIIWANLR | IPI00028030, IPI00910249, IPI00643348, IPI00328550 | Cartilage oligomeric matrix protein | COMP |
| LVPNPGQEDADR | IPI00028030, IPI00910249, IPI00643348, IPI00328550 | Cartilage oligomeric matrix protein | COMP |
| LVPNPGQEDADRDGVGDVCQDDFDADK | IPI00028030, IPI00910249, IPI00643348, IPI00328550 | Cartilage oligomeric matrix protein | COMP |
| NALWHTGDTESQVR | IPI00028030, IPI00910249, IPI00643348, IPI00328550 | Cartilage oligomeric matrix protein | COMP |
| QMEQTYWQANPFR | IPI00028030, IPI00910249, IPI00643348, IPI00328550 | Cartilage oligomeric matrix protein | COMP |
| QVCTDINECETGQHNCVPNSVCINTR | IPI00028030, IPI00910249, IPI00643348, IPI00328550 | Cartilage oligomeric matrix protein | COMP |
| VPNSDQKDSDGDGIGDACDNCPQK | IPI00028030, IPI00910249, IPI00643348, IPI00328550 | Cartilage oligomeric matrix protein | COMP |
| AEEEHLGILGPQLHADVGDK | IPI00792393, IPI00793626, IPI00794184, IPI00017601, IPI00879084, IPI00793108 | Ceruloplasmin | CP |
| AGLQAFFQVQECNK | IPI00792393, IPI00793626, IPI00794184, IPI00017601, IPI00879084, IPI00793108 | Ceruloplasmin | CP |
| ALYLQYTDETFR | IPI00792393, IPI00793626, IPI00794184, IPI00017601, IPI00879084, IPI00793108 | Ceruloplasmin | CP |
| DIFTGLIGPMK | IPI00792393, IPI00793626, IPI00794184, IPI00017601, IPI00879084, IPI00793108 | Ceruloplasmin | CP |
| DLYSGLIGPLIVCR | IPI00792393, IPI00793626, IPI00794184, IPI00017601, IPI00879084, IPI00793108 | Ceruloplasmin | CP |
| DVDKEFYLFPTVFDENESLLLEDNIR | IPI00792393, IPI00793626, IPI00794184, IPI00017601, IPI00879084, IPI00793108 | Ceruloplasmin | CP |
| EFYLFPTVFDENESLLLEDNIR | IPI00792393, IPI00793626, IPI00794184, IPI00017601, IPI00879084, IPI00793108 | Ceruloplasmin | CP |
| ERGPEEEHLGILGPVIWAEVGDTIR | IPI00792393, IPI00793626, IPI00794184, IPI00017601, IPI00879084, IPI00793108 | Ceruloplasmin | CP |
| EVGPTNADPVCLAK | IPI00792393, IPI00793626, IPI00794184, IPI00017601, IPI00879084, IPI00793108 | Ceruloplasmin | CP |
| EYTDASFTNR | IPI00792393, IPI00793626, IPI00794184, IPI00017601, IPI00879084, IPI00793108 | Ceruloplasmin | CP |
| FNKNNEGTYYSPNYNPQSR | IPI00792393, IPI00793626, IPI00794184, IPI00017601, IPI00879084, IPI00793108 | Ceruloplasmin | CP |
| GAYPLSIEPIGVR | IPI00792393, IPI00793626, IPI00794184, IPI00017601, IPI00879084, IPI00793108 | Ceruloplasmin | CP |
| GPEEEHLGILGPVIWAEVGDTIR | IPI00792393, IPI00793626, IPI00794184, IPI00017601, IPI00879084, IPI00793108 | Ceruloplasmin | CP |
| GVYSSDVFDIFPGTYQTLEMFPR | IPI00792393, IPI00793626, IPI00794184, IPI00017601, IPI00879084, IPI00793108 | Ceruloplasmin | CP |
| HYYIAAEEIIWNYAPSGIDIFTK | IPI00792393, IPI00793626, IPI00794184, IPI00017601, IPI00879084, IPI00793108 | Ceruloplasmin | CP |
| KAEEEHLGILGPQLHADVGDK | IPI00792393, IPI00793626, IPI00794184, IPI00017601, IPI00879084, IPI00793108 | Ceruloplasmin | CP |
| MFTTAPDQVDKEDEDFQESNK | IPI00792393, IPI00793626, IPI00794184, IPI00017601, IPI00879084, IPI00793108 | Ceruloplasmin | CP |
| MHSMNGFMYGNQPGLTMCK | IPI00792393, IPI00793626, IPI00794184, IPI00017601, IPI00879084, IPI00793108 | Ceruloplasmin | CP |
| MYSVNGYTFGSLPGLSMCAEDR | IPI00792393, IPI00793626, IPI00794184, IPI00017601, IPI00879084, IPI00793108 | Ceruloplasmin | CP |
| NNEGTYYSPNYNPQSR | IPI00792393, IPI00793626, IPI00794184, IPI00017601, IPI00879084, IPI00793108 | Ceruloplasmin | CP |
| QKDVDKEFYLFPTVFDENESLLLEDNIR | IPI00792393, IPI00793626, IPI00794184, IPI00017601, IPI00879084, IPI00793108 | Ceruloplasmin | CP |
| QSEDSTFYLGER | IPI00792393, IPI00793626, IPI00794184, IPI00017601, IPI00879084, IPI00793108 | Ceruloplasmin | CP |
| SGAGTEDSACIPWAYYSTVDQVK | IPI00792393, IPI00793626, IPI00794184, IPI00017601, IPI00879084, IPI00793108 | Ceruloplasmin | CP |
| SVPPSASHVAPTETFTYEWTVPK | IPI00792393, IPI00793626, IPI00794184, IPI00017601, IPI00879084, IPI00793108 | Ceruloplasmin | CP |
| TTIEKPVWLGFLGPIIK | IPI00792393, IPI00793626, IPI00794184, IPI00017601, IPI00879084, IPI00793108 | Ceruloplasmin | CP |
| TYCSEPEKVDKDNEDFQESNR | IPI00792393, IPI00793626, IPI00794184, IPI00017601, IPI00879084, IPI00793108 | Ceruloplasmin | CP |
| TYYIAAVEVEWDYSPQR | IPI00792393, IPI00793626, IPI00794184, IPI00017601, IPI00879084, IPI00793108 | Ceruloplasmin | CP |
| VDKDNEDFQESNR | IPI00792393, IPI00793626, IPI00794184, IPI00017601, IPI00879084, IPI00793108 | Ceruloplasmin | CP |
| VNKDDEEFIESNK | IPI00792393, IPI00793626, IPI00794184, IPI00017601, IPI00879084, IPI00793108 | Ceruloplasmin | CP |
| WYLFGMGNEVDVHAAFFHGQALTNK | IPI00792393, IPI00793626, IPI00794184, IPI00017601, IPI00879084, IPI00793108 | Ceruloplasmin | CP |
| ADDKVYPGEQYTYMLLATEEQSPGEGDGNCVTR | IPI00017601 | Ceruloplasmin precursor | CP |
| DIASGLIGPLIICK | IPI00017601 | Ceruloplasmin precursor | CP |
| KDSLDKEK | IPI00017601 | Ceruloplasmin precursor | CP |
| KLISVDTEHSNIYLQNGPDR | IPI00017601 | Ceruloplasmin precursor | CP |
| LISVDTEHSNIYLQNGPDR | IPI00017601 | Ceruloplasmin precursor | CP |
| MYYSAVDPTK | IPI00017601 | Ceruloplasmin precursor | CP |
| MYYSAVDPTKDIFTGLIGPMK | IPI00017601 | Ceruloplasmin precursor | CP |
| VYPGEQYTYMLLATEEQSPGEGDGNCVTR | IPI00017601 | Ceruloplasmin precursor | CP |
| DTGTYGFLLPER | IPI00329775 | Isoform 1 of Carboxypeptidase B2 precursor | CPB2 |
| EAFAAVSK | IPI00329775 | Isoform 1 of Carboxypeptidase B2 precursor | CPB2 |
| IAWHVIR | IPI00329775 | Isoform 1 of Carboxypeptidase B2 precursor | CPB2 |
| LVDFYVMPVVNVDGYDYSWK | IPI00329775 | Isoform 1 of Carboxypeptidase B2 precursor | CPB2 |
| YIKPTCR | IPI00329775 | Isoform 1 of Carboxypeptidase B2 precursor | CPB2 |
| YSFTIELR | IPI00329775 | Isoform 1 of Carboxypeptidase B2 precursor | CPB2 |
| ASASYYEQYHSLNEIYSWIEFITER | IPI00940541, IPI00293057, IPI00329775 | Isoform 2 of Carboxypeptidase B2 | CPB2 |
| AYISMHSYSQHIVFPYSYTR | IPI00940541, IPI00293057, IPI00329775 | Isoform 2 of Carboxypeptidase B2 | CPB2 |
| DHEELSLVASEAVR | IPI00940541, IPI00293057, IPI00329775 | Isoform 2 of Carboxypeptidase B2 | CPB2 |
| IHIGSSFEK | IPI00940541, IPI00293057, IPI00329775 | Isoform 2 of Carboxypeptidase B2 | CPB2 |
| NAIWIDCGIHAR | IPI00940541, IPI00293057, IPI00329775 | Isoform 2 of Carboxypeptidase B2 | CPB2 |
| SFYANNHCIGTDLNR | IPI00940541, IPI00293057, IPI00329775 | Isoform 2 of Carboxypeptidase B2 | CPB2 |
| ALIEWIR | IPI00027078 | Carboxypeptidase D precursor | CPD |
| NLWVLVVGR | IPI00026270 | Carboxypeptidase M precursor | CPM |
| EALIQFLEQVHQGIK | IPI00010295, IPI00641144 | Carboxypeptidase N catalytic chain precursor | CPN1 |
| ELMLQLSEFLCEEFR | IPI00010295 | Carboxypeptidase N catalytic chain precursor | CPN1 |
| FPPEEELQR | IPI00010295, IPI00641144 | Carboxypeptidase N catalytic chain precursor | CPN1 |
| IHILPSMNPDGYEVAAAQGPNKPGYLVGR | IPI00010295 | Carboxypeptidase N catalytic chain precursor | CPN1 |
| IVQLIQDTR | IPI00010295 | Carboxypeptidase N catalytic chain precursor | CPN1 |
| NFPDLNTYIYYNEK | IPI00010295 | Carboxypeptidase N catalytic chain precursor | CPN1 |
| SIPQVSPVR | IPI00010295 | Carboxypeptidase N catalytic chain precursor | CPN1 |
| TASTPTPDDKLFQK | IPI00010295, IPI00641144 | Carboxypeptidase N catalytic chain precursor | CPN1 |
| VQNECPGITR | IPI00010295 | Carboxypeptidase N catalytic chain precursor | CPN1 |
| YVGNMHGNEALGR | IPI00010295 | Carboxypeptidase N catalytic chain precursor | CPN1 |
| AGGSWDLAVQER | IPI00479116 | Carboxypeptidase N subunit 2 precursor | CPN2 |
| DHLGFQVTWPDESK | IPI00479116 | Carboxypeptidase N subunit 2 precursor | CPN2 |
| LELLSLSK | IPI00479116 | Carboxypeptidase N subunit 2 precursor | CPN2 |
| LFQPLTHLK | IPI00479116 | Carboxypeptidase N subunit 2 precursor | CPN2 |
| LLNIQTYCAGPAYLK | IPI00479116 | Carboxypeptidase N subunit 2 precursor | CPN2 |
| LSNNALSGLPQGVFGK | IPI00479116 | Carboxypeptidase N subunit 2 precursor | CPN2 |
| LTVSIEAR | IPI00479116 | Carboxypeptidase N subunit 2 precursor | CPN2 |
| NIIFVETSFTTLETR | IPI00479116 | Carboxypeptidase N subunit 2 precursor | CPN2 |
| QLVCPVTR | IPI00479116 | Carboxypeptidase N subunit 2 precursor | CPN2 |
| RLFQPLTHLK | IPI00479116 | Carboxypeptidase N subunit 2 precursor | CPN2 |
| SLMLSYNAITHLPAGIFR | IPI00479116 | Carboxypeptidase N subunit 2 precursor | CPN2 |
| SQCTYSNPEGTVVLACDQAQCR | IPI00479116 | Carboxypeptidase N subunit 2 precursor | CPN2 |
| VVFLNTQLCQFRPDAFGGLPR | IPI00479116 | Carboxypeptidase N subunit 2 precursor | CPN2 |
| TSTVPPSLR | IPI00004084, IPI00514304, IPI00221087 | Isoform 2 of Cyclic AMP-dependent transcription factor ATF-6 beta | CREBL1 |
| SSQNVAMIFFR | IPI00306844 | Corticotropin-releasing factor-binding protein precursor | CRHBP |
| AFTVCLHFYTELSSTR | IPI00218876, IPI00943038, IPI00642842, IPI00022389 | Isoform 1 of C-reactive protein | CRP |
| APLTKPLK | IPI00218876, IPI00943038, IPI00642842, IPI00022389 | Isoform 1 of C-reactive protein | CRP |
| ESDTSYVSLK | IPI00218876, IPI00943038, IPI00642842, IPI00022389 | Isoform 1 of C-reactive protein | CRP |
| YEVQGEVFTKPQLWP | IPI00218876, IPI00943038, IPI00642842, IPI00022389 | Isoform 1 of C-reactive protein | CRP |
| GYSIFSYATK | IPI00022389 | Isoform 1 of C-reactive protein precursor | CRP |
| RQDNEILIFWSK | IPI00022389 | Isoform 1 of C-reactive protein precursor | CRP |
| DVAAEAGVSK | IPI00451624, IPI00451625, IPI00451626 | Isoform 1 of Cartilage acidic protein 1 precursor | CRTAC1 |
| EHGDPLIEELNPGDALEPEGR | IPI00451624, IPI00451625, IPI00451626 | Isoform 1 of Cartilage acidic protein 1 precursor | CRTAC1 |
| FSMPSPVR | IPI00451624, IPI00451625, IPI00451626 | Isoform 1 of Cartilage acidic protein 1 precursor | CRTAC1 |
| GNQGFNNNWLR | IPI00451624, IPI00451625, IPI00451626 | Isoform 1 of Cartilage acidic protein 1 precursor | CRTAC1 |
| GTGGVVTDFDGDGMLDLILSHGESMAQPLSVFR | IPI00451624, IPI00451625, IPI00451626 | Isoform 1 of Cartilage acidic protein 1 precursor | CRTAC1 |
| GVASLFAGR | IPI00451624, IPI00451625, IPI00451626 | Isoform 1 of Cartilage acidic protein 1 precursor | CRTAC1 |
| GVSVGPILSSSASDIFCDNENGPNFLFHNR | IPI00451624, IPI00451625, IPI00451626 | Isoform 1 of Cartilage acidic protein 1 precursor | CRTAC1 |
| IIDGGSGYLCEMEPVAHFGLGK | IPI00451624, IPI00451625, IPI00451626 | Isoform 1 of Cartilage acidic protein 1 precursor | CRTAC1 |
| LVNIAVDER | IPI00451624, IPI00451625, IPI00451626 | Isoform 1 of Cartilage acidic protein 1 precursor | CRTAC1 |
| NVASGEMNSVLEILYPR | IPI00451624, IPI00451625, IPI00451626 | Isoform 1 of Cartilage acidic protein 1 precursor | CRTAC1 |
| QGNAIGVTACDIDGDGR | IPI00451624, IPI00451625, IPI00451626 | Isoform 1 of Cartilage acidic protein 1 precursor | CRTAC1 |
| TVITADFDNDQELEIFFNNIAYR | IPI00451624, IPI00451625, IPI00451626 | Isoform 1 of Cartilage acidic protein 1 precursor | CRTAC1 |
| VDIVYGNWNGPHR | IPI00451624, IPI00451625, IPI00451626 | Isoform 1 of Cartilage acidic protein 1 precursor | CRTAC1 |
| WEDILSDEVNVAR | IPI00451624, IPI00451625, IPI00451626 | Isoform 1 of Cartilage acidic protein 1 precursor | CRTAC1 |
| FIQSQDYQCSALMGGR | IPI00011218, IPI00922334 | Macrophage colony-stimulating factor 1 receptor | CSF1R |
| VIPGPPALTLVPAELVR | IPI00011218, IPI00922334 | Macrophage colony-stimulating factor 1 receptor | CSF1R |
| ALTFELTLR | IPI00011218 | Macrophage colony-stimulating factor 1 receptor precursor | CSF1R |
| AFCSFQIYAVPWQGTMTLSK | IPI00032293 | Cystatin-C precursor | CST3 |
| ALDFAVGEYNK | IPI00032293 | Cystatin-C precursor | CST3 |
| KAFCSFQIYAVPWQGTMTLSK | IPI00032293 | Cystatin-C precursor | CST3 |
| KQIVAGVNYFLDVELGR | IPI00032293 | Cystatin-C precursor | CST3 |
| LVGGPMDASVEEEGVR | IPI00032293 | Cystatin-C precursor | CST3 |
| LVGGPMDASVEEEGVRR | IPI00032293 | Cystatin-C precursor | CST3 |
| QIVAGVNYFLDVELGR | IPI00032293 | Cystatin-C precursor | CST3 |
| TQPNLDNCPFHDQPHLK | IPI00032293 | Cystatin-C precursor | CST3 |
| DLSPDDPQVQK | IPI00019954 | Cystatin-M precursor | CST6 |
| ATYIQNYR | IPI00643023, IPI00007778 | Di-N-acetylchitobiase precursor | CTBS |
| GDVSLKDIIDPAFR | IPI00643023, IPI00007778 | Di-N-acetylchitobiase precursor | CTBS |
| GIGMWNANCLDYSGDAVAK | IPI00643023, IPI00007778 | Di-N-acetylchitobiase precursor | CTBS |
| HHPDFEVFVFDVGQK | IPI00007778 | Di-N-acetylchitobiase precursor | CTBS |
| SYDWSQITTVATFGK | IPI00007778 | Di-N-acetylchitobiase precursor | CTBS |
| ILHLPTSWDWR | IPI00022810 | Dipeptidyl-peptidase 1 precursor | CTSC |
| EGCEAIVDTGTSLMVGPVDEVR | IPI00011229, IPI00892793, IPI00852597, IPI00658053, IPI00879328, IPI00853455 | Cathepsin D | CTSD |
| FDGILGMAYPR | IPI00011229, IPI00892793, IPI00852597, IPI00658053, IPI00879328, IPI00853455 | Cathepsin D | CTSD |
| ISVNNVLPVFDNLMQQK | IPI00011229, IPI00892793, IPI00852597, IPI00658053, IPI00879328, IPI00853455 | Cathepsin D | CTSD |
| LLDIACWIHHK | IPI00011229, IPI00892793, IPI00852597, IPI00658053, IPI00879328, IPI00853455 | Cathepsin D | CTSD |
| LVDQNIFSFYLSR | IPI00011229, IPI00892793, IPI00852597, IPI00658053, IPI00879328, IPI00853455 | Cathepsin D | CTSD |
| LSVFVNNMVR | IPI00002816 | Cathepsin F precursor | CTSF |
| STYPRPHEYLSPADLPK | IPI00002745, IPI00448792 | Cathepsin Z precursor | CTSZ |
| YGLFPANYVELR | IPI00029601, IPI00062884, IPI00792087 | Src substrate cortactin | CTTN |
| LGCSLNQNSVPDIHGVEAPAR | IPI00028911, IPI00910536 | Dystroglycan | DAG1 |
| AFYYPEEAGLAFGGPGSSR | IPI00171678 | Dopamine beta-hydroxylase | DBH |
| AGVLFGMSDR | IPI00514852, IPI00171678 | Dopamine beta-hydroxylase | DBH |
| DYLIEDGTVHLVYGILEEPFR | IPI00171678 | Dopamine beta-hydroxylase | DBH |
| FNAGIMELGLVYTPVMAIPPR | IPI00171678 | Dopamine beta-hydroxylase | DBH |
| FNNEDVCTCPQASVSQQFTSVPWNSFNR | IPI00171678 | Dopamine beta-hydroxylase | DBH |
| FQGEWNLQPLPK | IPI00171678 | Dopamine beta-hydroxylase | DBH |
| GQIHLDPQQDYQLLQVQR | IPI00171678 | Dopamine beta-hydroxylase | DBH |
| HVLAAWALGAK | IPI00514852, IPI00171678 | Dopamine beta-hydroxylase | DBH |
| KVVTVLVR | IPI00171678 | Dopamine beta-hydroxylase | DBH |
| TPEGLTLLFK | IPI00171678 | Dopamine beta-hydroxylase | DBH |
| VISTLEEPTPQCPTSQGR | IPI00171678 | Dopamine beta-hydroxylase | DBH |
| YFHLINR | IPI00171678 | Dopamine beta-hydroxylase | DBH |
| ATLGPAVRPLPWQR | IPI00165972 | Complement factor D preproprotein | DF |
| AVPHPDSQPDTIDHDLLLLQLSEK | IPI00165972 | Complement factor D preproprotein | DF |
| DSCKGDSGGPLVCGGVLEGVVTSGSR | IPI00165972 | Complement factor D preproprotein | DF |
| DVAPGTLCDVAGWGIVNHAGR | IPI00165972 | Complement factor D preproprotein | DF |
| GDSGGPLVCGGVLEGVVTSGSR | IPI00165972 | Complement factor D preproprotein | DF |
| VDRDVAPGTLCDVAGWGIVNHAGR | IPI00165972 | Complement factor D preproprotein | DF |
| VQVLLGAHSLSQPEPSK | IPI00165972 | Complement factor D preproprotein | DF |
| VQVLLGAHSLSQPEPSKR | IPI00165972 | Complement factor D preproprotein | DF |
| GLLFPVCTPLPVEGELCHDPASR | IPI00910436, IPI00383937, IPI00386812, IPI00940990, IPI00002714 | RIG-like 7-1 | DKK3 |
| LTLEQIDLIR | IPI00007127, IPI00902632, IPI00290954, IPI00007131, IPI00641736 | Isoform 1 of Dipeptidase 2 | DPEP2 |
| NVPDDILQLLK | IPI00007127, IPI00902632, IPI00290954, IPI00007131, IPI00641736 | Isoform 1 of Dipeptidase 2 | DPEP2 |
| ISLQWLR | IPI00018953 | Dipeptidyl peptidase 4 | DPP4 |
| LAYVWNNDIYVK | IPI00018953 | Dipeptidyl peptidase 4 | DPP4 |
| LGTFEVEDQIEAAR | IPI00018953 | Dipeptidyl peptidase 4 | DPP4 |
| SFSIFLSDGQR | IPI00386975, IPI00216099 | Isoform 1A of Desmocollin-1 | DSC1 |
| AWITAPVALR | IPI00028931 | Desmoglein-2 precursor | DSG2 |
| GITEPPFGIFVFNK | IPI00028931 | Desmoglein-2 precursor | DSG2 |
| GQIIGNFQAFDEDTGLPAHAR | IPI00028931 | Desmoglein-2 precursor | DSG2 |
| SEIQFLISDNQGFSCPEK | IPI00028931 | Desmoglein-2 precursor | DSG2 |
| ALPLALVLHELGAGR | IPI00853163, IPI00852987, IPI00292858 | Thymidine phosphorylase precursor | ECGF1 |
| ACPSHQPDISSGLELPFPPGVPTLDNIK | IPI00909747, IPI00006969, IPI00645849, IPI00003351 | Isoform 1 of Extracellular matrix protein 1 | ECM1 |
| AWEDTLDK | IPI00909747, IPI00006969, IPI00645849, IPI00003351 | Isoform 1 of Extracellular matrix protein 1 | ECM1 |
| CCDLPFPEQACCAEEEK | IPI00909747, IPI00006969, IPI00645849, IPI00003351 | Isoform 1 of Extracellular matrix protein 1 | ECM1 |
| DILTIDIGR | IPI00909747, IPI00006969, IPI00645849, IPI00003351 | Isoform 1 of Extracellular matrix protein 1 | ECM1 |
| DPALCCYLSPGDEQVNCFNINYLR | IPI00909747, IPI00006969, IPI00645849, IPI00003351 | Isoform 1 of Extracellular matrix protein 1 | ECM1 |
| ELLALIQLER | IPI00909747, IPI00006969, IPI00645849, IPI00003351 | Isoform 1 of Extracellular matrix protein 1 | ECM1 |
| ELPSLQHPNEQK | IPI00909747, IPI00006969, IPI00645849, IPI00003351 | Isoform 1 of Extracellular matrix protein 1 | ECM1 |
| EVGPPLPQEAVPLQK | IPI00909747, IPI00006969, IPI00645849, IPI00003351 | Isoform 1 of Extracellular matrix protein 1 | ECM1 |
| FCEAEFSVK | IPI00909747, IPI00006969, IPI00645849, IPI00003351 | Isoform 1 of Extracellular matrix protein 1 | ECM1 |
| FSCFQEEAPQPHYQLR | IPI00909747, IPI00006969, IPI00645849, IPI00003351 | Isoform 1 of Extracellular matrix protein 1 | ECM1 |
| LDGFPPGRPSPDNLNQICLPNR | IPI00909747, IPI00006969, IPI00645849, IPI00003351 | Isoform 1 of Extracellular matrix protein 1 | ECM1 |
| LLPAQLPAEK | IPI00909747, IPI00006969, IPI00645849, IPI00003351 | Isoform 1 of Extracellular matrix protein 1 | ECM1 |
| LTFINDLCGPR | IPI00909747, IPI00006969, IPI00645849, IPI00003351 | Isoform 1 of Extracellular matrix protein 1 | ECM1 |
| LVWEEAMSR | IPI00909747, IPI00006969, IPI00645849, IPI00003351 | Isoform 1 of Extracellular matrix protein 1 | ECM1 |
| NIWRDPALCCYLSPGDEQVNCFNINYLR | IPI00909747, IPI00006969, IPI00645849, IPI00003351 | Isoform 1 of Extracellular matrix protein 1 | ECM1 |
| NLPATDPLQR | IPI00909747, IPI00006969, IPI00645849, IPI00003351 | Isoform 1 of Extracellular matrix protein 1 | ECM1 |
| NVALVSGDTENAK | IPI00909747, IPI00006969, IPI00645849, IPI00003351 | Isoform 1 of Extracellular matrix protein 1 | ECM1 |
| QGETLNFLEIGYSR | IPI00909747, IPI00006969, IPI00645849, IPI00003351 | Isoform 1 of Extracellular matrix protein 1 | ECM1 |
| VTPNLMGHLCGNQR | IPI00909747, IPI00006969, IPI00645849, IPI00003351 | Isoform 1 of Extracellular matrix protein 1 | ECM1 |
| SLLHLVLLGNQIER | IPI00015315, IPI00942702 | Extracellular matrix protein 2 | ECM2 |
| CPQTVIPEGECCPVCSATEQR | IPI00942702 | Putative uncharacterized protein DKFZp686O0186 (Fragment) | ECM2 |
| ADQVCINLR | IPI00893517, IPI00220814, IPI00908496, IPI00922935, IPI00220813, IPI00029658, IPI00220815 | Isoform 1 of EGF-containing fibulin-like extracellular matrix protein 1 | EFEMP1 |
| FSCMCPQGYQVVR | IPI00893517, IPI00220814, IPI00908496, IPI00922935, IPI00220813, IPI00029658, IPI00220815 | Isoform 1 of EGF-containing fibulin-like extracellular matrix protein 1 | EFEMP1 |
| GSFACQCPPGYQK | IPI00893517, IPI00220814, IPI00908496, IPI00922935, IPI00220813, IPI00029658, IPI00220815 | Isoform 1 of EGF-containing fibulin-like extracellular matrix protein 1 | EFEMP1 |
| LNCEDIDECR | IPI00893517, IPI00220814, IPI00908496, IPI00922935, IPI00220813, IPI00029658, IPI00220815 | Isoform 1 of EGF-containing fibulin-like extracellular matrix protein 1 | EFEMP1 |
| NPCQDPYILTPENR | IPI00893517, IPI00220814, IPI00908496, IPI00922935, IPI00220813, IPI00029658, IPI00220815 | Isoform 1 of EGF-containing fibulin-like extracellular matrix protein 1 | EFEMP1 |
| SVPSDIFQIQATTIYANTINTFR | IPI00893517, IPI00220814, IPI00908496, IPI00922935, IPI00220813, IPI00029658, IPI00220815 | Isoform 1 of EGF-containing fibulin-like extracellular matrix protein 1 | EFEMP1 |
| TCQDINECETTNECR | IPI00893517, IPI00220814, IPI00908496, IPI00922935, IPI00220813, IPI00029658, IPI00220815 | Isoform 1 of EGF-containing fibulin-like extracellular matrix protein 1 | EFEMP1 |
| CGSGGPGSCPVPQECVPQDGAAGAGLCR | IPI00791343, IPI00027310, IPI00607580 | Isoform 1 of Multiple epidermal growth factor-like domains 8 | EGFL4 |
| ENVMLQSQK | IPI00167002, IPI00384476 | ETS-related transcription factor Elf-1 | ELF1 |
| GPITSAAELNDPQSILLR | IPI00219625, IPI00017567 | Isoform Long of Endoglin precursor | ENG |
| LPDTPQGLLGEAR | IPI00219625, IPI00017567 | Isoform Long of Endoglin precursor | ENG |
| LAMQEFMILPVGAANFR | IPI00465248, IPI00759806 | Isoform alpha-enolase of Alpha-enolase | ENO1 |
| VVIGMDVAASEFFR | IPI00465248, IPI00759806 | Isoform alpha-enolase of Alpha-enolase | ENO1 |
| ASLIDDAFALAR | IPI00014375 | Glutamyl aminopeptidase | ENPEP |
| AAECPAGFVRPPLIIFSVDGFR | IPI00794024, IPI00303210, IPI00878576, IPI00156171 | Isoform 1 of Ectonucleotide pyrophosphatase/phosphodiesterase family member 2 | ENPP2 |
| AGTFFWSVVIPHER | IPI00794024, IPI00303210, IPI00878576, IPI00156171 | Isoform 1 of Ectonucleotide pyrophosphatase/phosphodiesterase family member 2 | ENPP2 |
| CFELQEAGPPDCR | IPI00794024, IPI00303210, IPI00878576, IPI00156171 | Isoform 1 of Ectonucleotide pyrophosphatase/phosphodiesterase family member 2 | ENPP2 |
| DIEHLTSLDFFR | IPI00794024, IPI00303210, IPI00878576, IPI00156171 | Isoform 1 of Ectonucleotide pyrophosphatase/phosphodiesterase family member 2 | ENPP2 |
| IEDIHLLVER | IPI00794024, IPI00303210, IPI00878576, IPI00156171 | Isoform 1 of Ectonucleotide pyrophosphatase/phosphodiesterase family member 2 | ENPP2 |
| TEFLSNYLTNVDDITLVPGTLGR | IPI00794024, IPI00303210, IPI00878576, IPI00156171 | Isoform 1 of Ectonucleotide pyrophosphatase/phosphodiesterase family member 2 | ENPP2 |
| WWGGQPLWITATK | IPI00794024, IPI00303210, IPI00878576, IPI00156171 | Isoform 1 of Ectonucleotide pyrophosphatase/phosphodiesterase family member 2 | ENPP2 |
| YGPFGPEMTNPLR | IPI00794024, IPI00303210, IPI00878576, IPI00156171 | Isoform 1 of Ectonucleotide pyrophosphatase/phosphodiesterase family member 2 | ENPP2 |
| APGEGPQVACTGPPSAPR | IPI00294250, IPI00908698, IPI00911030 | Ephrin type-A receptor 1 | EPHA1 |
| QLPSFQTFFAPALDVIR | IPI00892952, IPI00303161, IPI00892879 | Endothelial cell-selective adhesion molecule | ESAM |
| IAESYQNILAAIEGSR | IPI00293128 | Exostosin-1 | EXT1 |
| LLLQIPSTIR | IPI00293128 | Exostosin-1 | EXT1 |
| GTNHLLFNMLPGGPPDYNTALDVPR | IPI00414468, IPI00004047, IPI00942173 | Isoform 1 of Exostosin-2 | EXT2 |
| FSGSGAGTDFTLK | IPI00829827, IPI00924820 | 13 kDa protein | EXTL2 |
| VIVVWNNIGEK | IPI00002732, IPI00640273, IPI00641761, IPI00939725 | 13 kDa protein | EXTL2 |
| ACIPTGPYPCGK | IPI00916794, IPI00552633, IPI00916124, IPI00019576 | Coagulation factor X | F10 |
| GYTLADNGK | IPI00916794, IPI00552633, IPI00916124, IPI00019576 | Coagulation factor X | F10 |
| KLCSLDNGDCDQFCHEEQNSVVCSCAR | IPI00916794, IPI00552633, IPI00916124, IPI00019576 | Coagulation factor X | F10 |
| LCSLDNGDCDQFCHEEQNSVVCSCAR | IPI00916794, IPI00552633, IPI00916124, IPI00019576 | Coagulation factor X | F10 |
| YKDGDQCETSPCQNQGK | IPI00916794, IPI00552633, IPI00916124, IPI00019576 | Coagulation factor X | F10 |
| DTYFVTGIVSWGEGCAR | IPI00019576 | Coagulation factor X precursor | F10 |
| DWAESTLMTQK | IPI00019576 | Coagulation factor X precursor | F10 |
| ETYDFDIAVLR | IPI00019576 | Coagulation factor X precursor | F10 |
| FKDTYFVTGIVSWGEGCAR | IPI00019576 | Coagulation factor X precursor | F10 |
| LKTPITFR | IPI00019576 | Coagulation factor X precursor | F10 |
| MLEVPYVDR | IPI00019576 | Coagulation factor X precursor | F10 |
| MNVAPACLPER | IPI00019576 | Coagulation factor X precursor | F10 |
| MNVAPACLPERDWAESTLMTQK | IPI00019576 | Coagulation factor X precursor | F10 |
| NTEQEEGGEAVHEVEVVIK | IPI00019576 | Coagulation factor X precursor | F10 |
| TGIVSGFGR | IPI00019576 | Coagulation factor X precursor | F10 |
| ALSGFSLQSCR | IPI00478635, IPI00419573, IPI00654888, IPI00930446, IPI00376200, IPI00915376, IPI00025400, IPI00010274, IPI00879123, IPI00879718, IPI00879984, IPI00424527, IPI00794017, IPI00419942, IPI00424528, IPI00216588, IPI00879551, IPI00411302, IPI00219931, IPI00941504, IPI00008556, IPI00641709, IPI00942399, IPI00216662, IPI00941399, IPI00472739 | Isoform 1 of Coagulation factor XI | F11 |
| CLLFSFLPASSINDMEK | IPI00478635, IPI00419573, IPI00654888, IPI00930446, IPI00376200, IPI00915376, IPI00025400, IPI00010274, IPI00879123, IPI00879718, IPI00879984, IPI00424527, IPI00794017, IPI00419942, IPI00424528, IPI00216588, IPI00879551, IPI00411302, IPI00219931, IPI00941504, IPI00008556, IPI00641709, IPI00942399, IPI00216662, IPI00941399, IPI00472739 | Isoform 1 of Coagulation factor XI | F11 |
| CLLFTFTAESPSEDPTR | IPI00478635, IPI00419573, IPI00654888, IPI00930446, IPI00376200, IPI00915376, IPI00025400, IPI00010274, IPI00879123, IPI00879718, IPI00879984, IPI00424527, IPI00794017, IPI00419942, IPI00424528, IPI00216588, IPI00879551, IPI00411302, IPI00219931, IPI00941504, IPI00008556, IPI00641709, IPI00942399, IPI00216662, IPI00941399, IPI00472739 | Isoform 1 of Coagulation factor XI | F11 |
| CQFFSYATQTFHK | IPI00478635, IPI00419573, IPI00654888, IPI00930446, IPI00376200, IPI00915376, IPI00025400, IPI00010274, IPI00879123, IPI00879718, IPI00879984, IPI00424527, IPI00794017, IPI00419942, IPI00424528, IPI00216588, IPI00879551, IPI00411302, IPI00219931, IPI00941504, IPI00008556, IPI00641709, IPI00942399, IPI00216662, IPI00941399, IPI00472739 | Isoform 1 of Coagulation factor XI | F11 |
| CQFFTYSLLPEDCK | IPI00478635, IPI00419573, IPI00654888, IPI00930446, IPI00376200, IPI00915376, IPI00025400, IPI00010274, IPI00879123, IPI00879718, IPI00879984, IPI00424527, IPI00794017, IPI00419942, IPI00424528, IPI00216588, IPI00879551, IPI00411302, IPI00219931, IPI00941504, IPI00008556, IPI00641709, IPI00942399, IPI00216662, IPI00941399, IPI00472739 | Isoform 1 of Coagulation factor XI | F11 |
| CQFFTYSLLPEDCKEEK | IPI00478635, IPI00419573, IPI00654888, IPI00930446, IPI00376200, IPI00915376, IPI00025400, IPI00010274, IPI00879123, IPI00879718, IPI00879984, IPI00424527, IPI00794017, IPI00419942, IPI00424528, IPI00216588, IPI00879551, IPI00411302, IPI00219931, IPI00941504, IPI00008556, IPI00641709, IPI00942399, IPI00216662, IPI00941399, IPI00472739 | Isoform 1 of Coagulation factor XI | F11 |
| CQFFTYTPAQASCNEGK | IPI00478635, IPI00419573, IPI00654888, IPI00930446, IPI00376200, IPI00915376, IPI00025400, IPI00010274, IPI00879123, IPI00879718, IPI00879984, IPI00424527, IPI00794017, IPI00419942, IPI00424528, IPI00216588, IPI00879551, IPI00411302, IPI00219931, IPI00941504, IPI00008556, IPI00641709, IPI00942399, IPI00216662, IPI00941399, IPI00472739 | Isoform 1 of Coagulation factor XI | F11 |
| DIFPNTVFADSNIDSVMAPDAFVCGR | IPI00478635, IPI00419573, IPI00654888, IPI00930446, IPI00376200, IPI00915376, IPI00025400, IPI00010274, IPI00879123, IPI00879718, IPI00879984, IPI00424527, IPI00794017, IPI00419942, IPI00424528, IPI00216588, IPI00879551, IPI00411302, IPI00219931, IPI00941504, IPI00008556, IPI00641709, IPI00942399, IPI00216662, IPI00941399, IPI00472739 | Isoform 1 of Coagulation factor XI | F11 |
| DSVTETLPR | IPI00478635, IPI00419573, IPI00654888, IPI00930446, IPI00376200, IPI00915376, IPI00025400, IPI00010274, IPI00879123, IPI00879718, IPI00879984, IPI00424527, IPI00794017, IPI00419942, IPI00424528, IPI00216588, IPI00879551, IPI00411302, IPI00219931, IPI00941504, IPI00008556, IPI00641709, IPI00942399, IPI00216662, IPI00941399, IPI00472739 | Isoform 1 of Coagulation factor XI | F11 |
| DSVTGTLPK | IPI00478635, IPI00419573, IPI00654888, IPI00930446, IPI00376200, IPI00915376, IPI00025400, IPI00010274, IPI00879123, IPI00879718, IPI00879984, IPI00424527, IPI00794017, IPI00419942, IPI00424528, IPI00216588, IPI00879551, IPI00411302, IPI00219931, IPI00941504, IPI00008556, IPI00641709, IPI00942399, IPI00216662, IPI00941399, IPI00472739 | Isoform 1 of Coagulation factor XI | F11 |
| DTCFEGGDITTVFTPSAK | IPI00478635, IPI00419573, IPI00654888, IPI00930446, IPI00376200, IPI00915376, IPI00025400, IPI00010274, IPI00879123, IPI00879718, IPI00879984, IPI00424527, IPI00794017, IPI00419942, IPI00424528, IPI00216588, IPI00879551, IPI00411302, IPI00219931, IPI00941504, IPI00008556, IPI00641709, IPI00942399, IPI00216662, IPI00941399, IPI00472739 | Isoform 1 of Coagulation factor XI | F11 |
| DTPFSQIK | IPI00478635, IPI00419573, IPI00654888, IPI00930446, IPI00376200, IPI00915376, IPI00025400, IPI00010274, IPI00879123, IPI00879718, IPI00879984, IPI00424527, IPI00794017, IPI00419942, IPI00424528, IPI00216588, IPI00879551, IPI00411302, IPI00219931, IPI00941504, IPI00008556, IPI00641709, IPI00942399, IPI00216662, IPI00941399, IPI00472739 | Isoform 1 of Coagulation factor XI | F11 |
| EDTSFFGVQEIIIHDQYK | IPI00478635, IPI00419573, IPI00654888, IPI00930446, IPI00376200, IPI00915376, IPI00025400, IPI00010274, IPI00879123, IPI00879718, IPI00879984, IPI00424527, IPI00794017, IPI00419942, IPI00424528, IPI00216588, IPI00879551, IPI00411302, IPI00219931, IPI00941504, IPI00008556, IPI00641709, IPI00942399, IPI00216662, IPI00941399, IPI00472739 | Isoform 1 of Coagulation factor XI | F11 |
| EIIIHQNYK | IPI00478635, IPI00419573, IPI00654888, IPI00930446, IPI00376200, IPI00915376, IPI00025400, IPI00010274, IPI00879123, IPI00879718, IPI00879984, IPI00424527, IPI00794017, IPI00419942, IPI00424528, IPI00216588, IPI00879551, IPI00411302, IPI00219931, IPI00941504, IPI00008556, IPI00641709, IPI00942399, IPI00216662, IPI00941399, IPI00472739 | Isoform 1 of Coagulation factor XI | F11 |
| ERPGVYTNVVEYVDWILEK | IPI00478635, IPI00419573, IPI00654888, IPI00930446, IPI00376200, IPI00915376, IPI00025400, IPI00010274, IPI00879123, IPI00879718, IPI00879984, IPI00424527, IPI00794017, IPI00419942, IPI00424528, IPI00216588, IPI00879551, IPI00411302, IPI00219931, IPI00941504, IPI00008556, IPI00641709, IPI00942399, IPI00216662, IPI00941399, IPI00472739 | Isoform 1 of Coagulation factor XI | F11 |
| GDSGGPLVCK | IPI00478635, IPI00419573, IPI00654888, IPI00930446, IPI00376200, IPI00915376, IPI00025400, IPI00010274, IPI00879123, IPI00879718, IPI00879984, IPI00424527, IPI00794017, IPI00419942, IPI00424528, IPI00216588, IPI00879551, IPI00411302, IPI00219931, IPI00941504, IPI00008556, IPI00641709, IPI00942399, IPI00216662, IPI00941399, IPI00472739 | Isoform 1 of Coagulation factor XI | F11 |
| GDTSTIYTNCWVTGWGFSK | IPI00478635, IPI00419573, IPI00654888, IPI00930446, IPI00376200, IPI00915376, IPI00025400, IPI00010274, IPI00879123, IPI00879718, IPI00879984, IPI00424527, IPI00794017, IPI00419942, IPI00424528, IPI00216588, IPI00879551, IPI00411302, IPI00219931, IPI00941504, IPI00008556, IPI00641709, IPI00942399, IPI00216662, IPI00941399, IPI00472739 | Isoform 1 of Coagulation factor XI | F11 |
| GEWPWQVTLHTTSPTQR | IPI00478635, IPI00419573, IPI00654888, IPI00930446, IPI00376200, IPI00915376, IPI00025400, IPI00010274, IPI00879123, IPI00879718, IPI00879984, IPI00424527, IPI00794017, IPI00419942, IPI00424528, IPI00216588, IPI00879551, IPI00411302, IPI00219931, IPI00941504, IPI00008556, IPI00641709, IPI00942399, IPI00216662, IPI00941399, IPI00472739 | Isoform 1 of Coagulation factor XI | F11 |
| GGDVASMYTPNAQYCQMR | IPI00478635, IPI00419573, IPI00654888, IPI00930446, IPI00376200, IPI00915376, IPI00025400, IPI00010274, IPI00879123, IPI00879718, IPI00879984, IPI00424527, IPI00794017, IPI00419942, IPI00424528, IPI00216588, IPI00879551, IPI00411302, IPI00219931, IPI00941504, IPI00008556, IPI00641709, IPI00942399, IPI00216662, IPI00941399, IPI00472739 | Isoform 1 of Coagulation factor XI | F11 |
| GGISGYTLR | IPI00478635, IPI00419573, IPI00654888, IPI00930446, IPI00376200, IPI00915376, IPI00025400, IPI00010274, IPI00879123, IPI00879718, IPI00879984, IPI00424527, IPI00794017, IPI00419942, IPI00424528, IPI00216588, IPI00879551, IPI00411302, IPI00219931, IPI00941504, IPI00008556, IPI00641709, IPI00942399, IPI00216662, IPI00941399, IPI00472739 | Isoform 1 of Coagulation factor XI | F11 |
| GVNVCQETCTK | IPI00478635, IPI00419573, IPI00654888, IPI00930446, IPI00376200, IPI00915376, IPI00025400, IPI00010274, IPI00879123, IPI00879718, IPI00879984, IPI00424527, IPI00794017, IPI00419942, IPI00424528, IPI00216588, IPI00879551, IPI00411302, IPI00219931, IPI00941504, IPI00008556, IPI00641709, IPI00942399, IPI00216662, IPI00941399, IPI00472739 | Isoform 1 of Coagulation factor XI | F11 |
| HLCGGSLIGHQWVLTAAHCFDGLPLQDVWR | IPI00478635, IPI00419573, IPI00654888, IPI00930446, IPI00376200, IPI00915376, IPI00025400, IPI00010274, IPI00879123, IPI00879718, IPI00879984, IPI00424527, IPI00794017, IPI00419942, IPI00424528, IPI00216588, IPI00879551, IPI00411302, IPI00219931, IPI00941504, IPI00008556, IPI00641709, IPI00942399, IPI00216662, IPI00941399, IPI00472739 | Isoform 1 of Coagulation factor XI | F11 |
| HNEVWHLVGITSWGEGCAQR | IPI00478635, IPI00419573, IPI00654888, IPI00930446, IPI00376200, IPI00915376, IPI00025400, IPI00010274, IPI00879123, IPI00879718, IPI00879984, IPI00424527, IPI00794017, IPI00419942, IPI00424528, IPI00216588, IPI00879551, IPI00411302, IPI00219931, IPI00941504, IPI00008556, IPI00641709, IPI00942399, IPI00216662, IPI00941399, IPI00472739 | Isoform 1 of Coagulation factor XI | F11 |
| IAYGTQGSSGYSLR | IPI00478635, IPI00419573, IPI00654888, IPI00930446, IPI00376200, IPI00915376, IPI00025400, IPI00010274, IPI00879123, IPI00879718, IPI00879984, IPI00424527, IPI00794017, IPI00419942, IPI00424528, IPI00216588, IPI00879551, IPI00411302, IPI00219931, IPI00941504, IPI00008556, IPI00641709, IPI00942399, IPI00216662, IPI00941399, IPI00472739 | Isoform 1 of Coagulation factor XI | F11 |
| ICTHHPGCLFFTFFSQEWPK | IPI00478635, IPI00419573, IPI00654888, IPI00930446, IPI00376200, IPI00915376, IPI00025400, IPI00010274, IPI00879123, IPI00879718, IPI00879984, IPI00424527, IPI00794017, IPI00419942, IPI00424528, IPI00216588, IPI00879551, IPI00411302, IPI00219931, IPI00941504, IPI00008556, IPI00641709, IPI00942399, IPI00216662, IPI00941399, IPI00472739 | Isoform 1 of Coagulation factor XI | F11 |
| IPLVTNEECQK | IPI00478635, IPI00419573, IPI00654888, IPI00930446, IPI00376200, IPI00915376, IPI00025400, IPI00010274, IPI00879123, IPI00879718, IPI00879984, IPI00424527, IPI00794017, IPI00419942, IPI00424528, IPI00216588, IPI00879551, IPI00411302, IPI00219931, IPI00941504, IPI00008556, IPI00641709, IPI00942399, IPI00216662, IPI00941399, IPI00472739 | Isoform 1 of Coagulation factor XI | F11 |
| IVGGTASVR | IPI00478635, IPI00419573, IPI00654888, IPI00930446, IPI00376200, IPI00915376, IPI00025400, IPI00010274, IPI00879123, IPI00879718, IPI00879984, IPI00424527, IPI00794017, IPI00419942, IPI00424528, IPI00216588, IPI00879551, IPI00411302, IPI00219931, IPI00941504, IPI00008556, IPI00641709, IPI00942399, IPI00216662, IPI00941399, IPI00472739 | Isoform 1 of Coagulation factor XI | F11 |
| IYSGILNLSDITK | IPI00478635, IPI00419573, IPI00654888, IPI00930446, IPI00376200, IPI00915376, IPI00025400, IPI00010274, IPI00879123, IPI00879718, IPI00879984, IPI00424527, IPI00794017, IPI00419942, IPI00424528, IPI00216588, IPI00879551, IPI00411302, IPI00219931, IPI00941504, IPI00008556, IPI00641709, IPI00942399, IPI00216662, IPI00941399, IPI00472739 | Isoform 1 of Coagulation factor XI | F11 |
| LCNTGDNSVCTTK | IPI00478635, IPI00419573, IPI00654888, IPI00930446, IPI00376200, IPI00915376, IPI00025400, IPI00010274, IPI00879123, IPI00879718, IPI00879984, IPI00424527, IPI00794017, IPI00419942, IPI00424528, IPI00216588, IPI00879551, IPI00411302, IPI00219931, IPI00941504, IPI00008556, IPI00641709, IPI00942399, IPI00216662, IPI00941399, IPI00472739 | Isoform 1 of Coagulation factor XI | F11 |
| LSMDGSPTR | IPI00478635, IPI00419573, IPI00654888, IPI00930446, IPI00376200, IPI00915376, IPI00025400, IPI00010274, IPI00879123, IPI00879718, IPI00879984, IPI00424527, IPI00794017, IPI00419942, IPI00424528, IPI00216588, IPI00879551, IPI00411302, IPI00219931, IPI00941504, IPI00008556, IPI00641709, IPI00942399, IPI00216662, IPI00941399, IPI00472739 | Isoform 1 of Coagulation factor XI | F11 |
| MAESGYDIALLK | IPI00478635, IPI00419573, IPI00654888, IPI00930446, IPI00376200, IPI00915376, IPI00025400, IPI00010274, IPI00879123, IPI00879718, IPI00879984, IPI00424527, IPI00794017, IPI00419942, IPI00424528, IPI00216588, IPI00879551, IPI00411302, IPI00219931, IPI00941504, IPI00008556, IPI00641709, IPI00942399, IPI00216662, IPI00941399, IPI00472739 | Isoform 1 of Coagulation factor XI | F11 |
| NVIYTDCWVTGWGYR | IPI00478635, IPI00419573, IPI00654888, IPI00930446, IPI00376200, IPI00915376, IPI00025400, IPI00010274, IPI00879123, IPI00879718, IPI00879984, IPI00424527, IPI00794017, IPI00419942, IPI00424528, IPI00216588, IPI00879551, IPI00411302, IPI00219931, IPI00941504, IPI00008556, IPI00641709, IPI00942399, IPI00216662, IPI00941399, IPI00472739 | Isoform 1 of Coagulation factor XI | F11 |
| SCALSNLACIR | IPI00478635, IPI00419573, IPI00654888, IPI00930446, IPI00376200, IPI00915376, IPI00025400, IPI00010274, IPI00879123, IPI00879718, IPI00879984, IPI00424527, IPI00794017, IPI00419942, IPI00424528, IPI00216588, IPI00879551, IPI00411302, IPI00219931, IPI00941504, IPI00008556, IPI00641709, IPI00942399, IPI00216662, IPI00941399, IPI00472739 | Isoform 1 of Coagulation factor XI | F11 |
| TAAISGYSFK | IPI00478635, IPI00419573, IPI00654888, IPI00930446, IPI00376200, IPI00915376, IPI00025400, IPI00010274, IPI00879123, IPI00879718, IPI00879984, IPI00424527, IPI00794017, IPI00419942, IPI00424528, IPI00216588, IPI00879551, IPI00411302, IPI00219931, IPI00941504, IPI00008556, IPI00641709, IPI00942399, IPI00216662, IPI00941399, IPI00472739 | Isoform 1 of Coagulation factor XI | F11 |
| TICTYHPNCLFFTFYTNVWK | IPI00478635, IPI00419573, IPI00654888, IPI00930446, IPI00376200, IPI00915376, IPI00025400, IPI00010274, IPI00879123, IPI00879718, IPI00879984, IPI00424527, IPI00794017, IPI00419942, IPI00424528, IPI00216588, IPI00879551, IPI00411302, IPI00219931, IPI00941504, IPI00008556, IPI00641709, IPI00942399, IPI00216662, IPI00941399, IPI00472739 | Isoform 1 of Coagulation factor XI | F11 |
| TLPEPCHSK | IPI00478635, IPI00419573, IPI00654888, IPI00930446, IPI00376200, IPI00915376, IPI00025400, IPI00010274, IPI00879123, IPI00879718, IPI00879984, IPI00424527, IPI00794017, IPI00419942, IPI00424528, IPI00216588, IPI00879551, IPI00411302, IPI00219931, IPI00941504, IPI00008556, IPI00641709, IPI00942399, IPI00216662, IPI00941399, IPI00472739 | Isoform 1 of Coagulation factor XI | F11 |
| VLTPDAFVCR | IPI00478635, IPI00419573, IPI00654888, IPI00930446, IPI00376200, IPI00915376, IPI00025400, IPI00010274, IPI00879123, IPI00879718, IPI00879984, IPI00424527, IPI00794017, IPI00419942, IPI00424528, IPI00216588, IPI00879551, IPI00411302, IPI00219931, IPI00941504, IPI00008556, IPI00641709, IPI00942399, IPI00216662, IPI00941399, IPI00472739 | Isoform 1 of Coagulation factor XI | F11 |
| VSEGNHDIALIK | IPI00478635, IPI00419573, IPI00654888, IPI00930446, IPI00376200, IPI00915376, IPI00025400, IPI00010274, IPI00879123, IPI00879718, IPI00879984, IPI00424527, IPI00794017, IPI00419942, IPI00424528, IPI00216588, IPI00879551, IPI00411302, IPI00219931, IPI00941504, IPI00008556, IPI00641709, IPI00942399, IPI00216662, IPI00941399, IPI00472739 | Isoform 1 of Coagulation factor XI | F11 |
| VSSVEECQK | IPI00478635, IPI00419573, IPI00654888, IPI00930446, IPI00376200, IPI00915376, IPI00025400, IPI00010274, IPI00879123, IPI00879718, IPI00879984, IPI00424527, IPI00794017, IPI00419942, IPI00424528, IPI00216588, IPI00879551, IPI00411302, IPI00219931, IPI00941504, IPI00008556, IPI00641709, IPI00942399, IPI00216662, IPI00941399, IPI00472739 | Isoform 1 of Coagulation factor XI | F11 |
| VVSGFSLK | IPI00478635, IPI00419573, IPI00654888, IPI00930446, IPI00376200, IPI00915376, IPI00025400, IPI00010274, IPI00879123, IPI00879718, IPI00879984, IPI00424527, IPI00794017, IPI00419942, IPI00424528, IPI00216588, IPI00879551, IPI00411302, IPI00219931, IPI00941504, IPI00008556, IPI00641709, IPI00942399, IPI00216662, IPI00941399, IPI00472739 | Isoform 1 of Coagulation factor XI | F11 |
| YCQVVCTYHPR | IPI00478635, IPI00419573, IPI00654888, IPI00930446, IPI00376200, IPI00915376, IPI00025400, IPI00010274, IPI00879123, IPI00879718, IPI00879984, IPI00424527, IPI00794017, IPI00419942, IPI00424528, IPI00216588, IPI00879551, IPI00411302, IPI00219931, IPI00941504, IPI00008556, IPI00641709, IPI00942399, IPI00216662, IPI00941399, IPI00472739 | Isoform 1 of Coagulation factor XI | F11 |
| YSPGGTPTAIK | IPI00478635, IPI00419573, IPI00654888, IPI00930446, IPI00376200, IPI00915376, IPI00025400, IPI00010274, IPI00879123, IPI00879718, IPI00879984, IPI00424527, IPI00794017, IPI00419942, IPI00424528, IPI00216588, IPI00879551, IPI00411302, IPI00219931, IPI00941504, IPI00008556, IPI00641709, IPI00942399, IPI00216662, IPI00941399, IPI00472739 | Isoform 1 of Coagulation factor XI | F11 |
| AEEHTVVLTVTGEPCHFPFQYHR | IPI00019581 | Coagulation factor XII precursor | F12 |
| CFEPQLLR | IPI00019581 | Coagulation factor XII precursor | F12 |
| EQPPSLTR | IPI00019581 | Coagulation factor XII precursor | F12 |
| GRPGPQPWCATTPNFDQDQR | IPI00019581 | Coagulation factor XII precursor | F12 |
| LCHCPVGYTGPFCDVDTK | IPI00019581 | Coagulation factor XII precursor | F12 |
| LHEAFSPVSYQHDLALLR | IPI00019581 | Coagulation factor XII precursor | F12 |
| LTLQGIISWGSGCGDR | IPI00019581 | Coagulation factor XII precursor | F12 |
| NEIWYR | IPI00019581 | Coagulation factor XII precursor | F12 |
| NKPGVYTDVAYYLAWIR | IPI00019581 | Coagulation factor XII precursor | F12 |
| NKPGVYTDVAYYLAWIREHTVS | IPI00019581 | Coagulation factor XII precursor | F12 |
| NPDNDIRPWCFVLNR | IPI00019581 | Coagulation factor XII precursor | F12 |
| NWGLGGHAFCR | IPI00019581 | Coagulation factor XII precursor | F12 |
| RLTLQGIISWGSGCGDR | IPI00019581 | Coagulation factor XII precursor | F12 |
| TTLSGAPCQPWASEATYR | IPI00019581 | Coagulation factor XII precursor | F12 |
| VVGGLVALR | IPI00019581 | Coagulation factor XII precursor | F12 |
| WGYCLEPK | IPI00019581 | Coagulation factor XII precursor | F12 |
| AVPPNNSNAAEDDLPTVELQGVVPR | IPI00855773, IPI00884176, IPI00513858, IPI00642188, IPI00855854, IPI00297550 | Coagulation factor XIII A chain | F13A1 |
| ETFDVTLEPLSFK | IPI00855773, IPI00884176, IPI00513858, IPI00642188, IPI00855854, IPI00297550 | Coagulation factor XIII A chain | F13A1 |
| GTYIPVPIVSELQSGK | IPI00855773, IPI00884176, IPI00513858, IPI00642188, IPI00855854, IPI00297550 | Coagulation factor XIII A chain | F13A1 |
| HVYGELDVQIQR | IPI00855773, IPI00884176, IPI00513858, IPI00642188, IPI00855854, IPI00297550 | Coagulation factor XIII A chain | F13A1 |
| KLIASMSSDSLR | IPI00855773, IPI00884176, IPI00513858, IPI00642188, IPI00855854, IPI00297550 | Coagulation factor XIII A chain | F13A1 |
| LALETALMYGAK | IPI00855773, IPI00884176, IPI00513858, IPI00642188, IPI00855854, IPI00297550 | Coagulation factor XIII A chain | F13A1 |
| MYVAVWTPYGVLR | IPI00855773, IPI00884176, IPI00513858, IPI00642188, IPI00855854, IPI00297550 | Coagulation factor XIII A chain | F13A1 |
| QIGGDGMMDITDTYK | IPI00855773, IPI00884176, IPI00513858, IPI00642188, IPI00855854, IPI00297550 | Coagulation factor XIII A chain | F13A1 |
| SNVDMDFEVENAVLGK | IPI00855773, IPI00884176, IPI00513858, IPI00642188, IPI00855854, IPI00297550 | Coagulation factor XIII A chain | F13A1 |
| YGQCWVFAGVFNTFLR | IPI00855773, IPI00884176, IPI00513858, IPI00642188, IPI00855854, IPI00297550 | Coagulation factor XIII A chain | F13A1 |
| CFDHHFLEGSR | IPI00007240 | Coagulation factor XIII B chain precursor | F13B |
| CPPPPLPINSK | IPI00007240 | Coagulation factor XIII B chain precursor | F13B |
| DKVQYECATGYYTAGGK | IPI00007240 | Coagulation factor XIII B chain precursor | F13B |
| GDTYPAELYITGSILR | IPI00007240 | Coagulation factor XIII B chain precursor | F13B |
| HPPVVMNGAVADGILASYATGSSVEYR | IPI00007240 | Coagulation factor XIII B chain precursor | F13B |
| IAQYYYTFK | IPI00007240 | Coagulation factor XIII B chain precursor | F13B |
| KLSFFCLAGYTTESGR | IPI00007240 | Coagulation factor XIII B chain precursor | F13B |
| LIENGYFHPVK | IPI00007240 | Coagulation factor XIII B chain precursor | F13B |
| LSFFCLAGYTTESGR | IPI00007240 | Coagulation factor XIII B chain precursor | F13B |
| QEEQTTCTTEGWSPEPR | IPI00007240 | Coagulation factor XIII B chain precursor | F13B |
| QGYDLSPLTPLSELSVQCNR | IPI00007240 | Coagulation factor XIII B chain precursor | F13B |
| QSTLSYQEPLR | IPI00007240 | Coagulation factor XIII B chain precursor | F13B |
| QSTLSYQEPLRT | IPI00007240 | Coagulation factor XIII B chain precursor | F13B |
| SFYFPMSIDK | IPI00007240 | Coagulation factor XIII B chain precursor | F13B |
| TTGGKDEEVVQCLSDGWSSQPTCR | IPI00007240 | Coagulation factor XIII B chain precursor | F13B |
| VACEEPPFIENGAANLHSK | IPI00007240 | Coagulation factor XIII B chain precursor | F13B |
| VLHGDLIDFVCK | IPI00007240 | Coagulation factor XIII B chain precursor | F13B |
| VQYECATGYYTAGGK | IPI00007240 | Coagulation factor XIII B chain precursor | F13B |
| VTYACK | IPI00007240 | Coagulation factor XIII B chain precursor | F13B |
| WSSPPVCLEPCTVNVDYMNR | IPI00007240 | Coagulation factor XIII B chain precursor | F13B |
| WTLPPECVENNENCK | IPI00007240 | Coagulation factor XIII B chain precursor | F13B |
| ANTFLEEVR | IPI00877967, IPI00019568 | Prothrombin (Fragment) | F2 |
| ANTFLEEVRK | IPI00877967, IPI00019568 | Prothrombin (Fragment) | F2 |
| DKLAACLEGNCAEGLGTNYR | IPI00877967, IPI00019568 | Prothrombin (Fragment) | F2 |
| HQDFNSAVQLVENFCR | IPI00877967, IPI00019568 | Prothrombin (Fragment) | F2 |
| LAACLEGNCAEGLGTNYR | IPI00877967, IPI00019568 | Prothrombin (Fragment) | F2 |
| LAVTTHGLPCLAWASAQAK | IPI00877967, IPI00019568 | Prothrombin (Fragment) | F2 |
| NPDSSTTGPWCYTTDPTVR | IPI00877967, IPI00019568 | Prothrombin (Fragment) | F2 |
| QECSIPVCGQDQVTVAMTPR | IPI00877967, IPI00019568 | Prothrombin (Fragment) | F2 |
| RQECSIPVCGQDQVTVAMTPR | IPI00877967, IPI00019568 | Prothrombin (Fragment) | F2 |
| SEGSSVNLSPPLEQCVPDR | IPI00877967, IPI00019568 | Prothrombin (Fragment) | F2 |
| SGIECQLWR | IPI00877967, IPI00019568 | Prothrombin (Fragment) | F2 |
| TATSEYQTFFNPR | IPI00877967, IPI00019568 | Prothrombin (Fragment) | F2 |
| ELLESYIDGR | IPI00019568 | Prothrombin precursor (Fragment) | F2 |
| ENLDRDIALMK | IPI00019568 | Prothrombin precursor (Fragment) | F2 |
| ETAASLLQAGYK | IPI00019568 | Prothrombin precursor (Fragment) | F2 |
| ETWTANVGK | IPI00019568 | Prothrombin precursor (Fragment) | F2 |
| GDACEGDSGGPFVMK | IPI00019568 | Prothrombin precursor (Fragment) | F2 |
| GQPSVLQVVNLPIVERPVCK | IPI00019568 | Prothrombin precursor (Fragment) | F2 |
| ISMLEK | IPI00019568 | Prothrombin precursor (Fragment) | F2 |
| ITDNMFCAGYKPDEGK | IPI00019568 | Prothrombin precursor (Fragment) | F2 |
| ITDNMFCAGYKPDEGKR | IPI00019568 | Prothrombin precursor (Fragment) | F2 |
| IVEGSDAEIGMSPWQVMLFR | IPI00019568 | Prothrombin precursor (Fragment) | F2 |
| IYIHPR | IPI00019568 | Prothrombin precursor (Fragment) | F2 |
| KPVAFSDYIHPVCLPDR | IPI00019568 | Prothrombin precursor (Fragment) | F2 |
| KPVAFSDYIHPVCLPDRETAASLLQAGYK | IPI00019568 | Prothrombin precursor (Fragment) | F2 |
| KSPQELLCGASLISDR | IPI00019568 | Prothrombin precursor (Fragment) | F2 |
| LKKPVAFSDYIHPVCLPDR | IPI00019568 | Prothrombin precursor (Fragment) | F2 |
| RGDACEGDSGGPFVMK | IPI00019568 | Prothrombin precursor (Fragment) | F2 |
| SPQELLCGASLISDR | IPI00019568 | Prothrombin precursor (Fragment) | F2 |
| TFGSGEADCGLRPLFEK | IPI00019568 | Prothrombin precursor (Fragment) | F2 |
| VTGWGNLK | IPI00019568 | Prothrombin precursor (Fragment) | F2 |
| WYQMGIVSWGEGCDR | IPI00019568 | Prothrombin precursor (Fragment) | F2 |
| WYQMGIVSWGEGCDRDGK | IPI00019568 | Prothrombin precursor (Fragment) | F2 |
| YGFYTHVFR | IPI00019568 | Prothrombin precursor (Fragment) | F2 |
| AADIEQQAVFAVFDENK | IPI00815941, IPI00022937, IPI00478809 | Coagulation factor V | F5 |
| AEVDDVIQVR | IPI00815941, IPI00022937, IPI00478809 | Coagulation factor V | F5 |
| AGMQTPFLIMDR | IPI00815941, IPI00022937, IPI00478809 | Coagulation factor V | F5 |
| ASEFLGYWEPR | IPI00815941, IPI00022937, IPI00478809 | Coagulation factor V | F5 |
| DIASGLIGLLLICK | IPI00815941, IPI00022937, IPI00478809 | Coagulation factor V | F5 |
| EDGILGPIIR | IPI00815941, IPI00022937, IPI00478809 | Coagulation factor V | F5 |
| GEYEEHLGILGPIIR | IPI00815941, IPI00022937, IPI00478809 | Coagulation factor V | F5 |
| LLSLGAGEFK | IPI00815941, IPI00022937, IPI00478809 | Coagulation factor V | F5 |
| QWLEIDLLK | IPI00815941, IPI00022937, IPI00478809 | Coagulation factor V | F5 |
| SWWGDYWEPFR | IPI00815941, IPI00022937, IPI00478809 | Coagulation factor V | F5 |
| SWYLEDNINK | IPI00815941, IPI00022937, IPI00478809 | Coagulation factor V | F5 |
| WIISSLTPK | IPI00815941, IPI00022937, IPI00478809 | Coagulation factor V | F5 |
| DQLQSYICFCLPAFEGR | IPI00940181, IPI00798065, IPI00329555 | Isoform A of Coagulation factor VII | F7 |
| FSLVSGWGQLLDR | IPI00940181, IPI00798065, IPI00329555 | Isoform A of Coagulation factor VII | F7 |
| GATALELMVLNVPR | IPI00940181, IPI00798065, IPI00329555 | Isoform A of Coagulation factor VII | F7 |
| LHQPVVLTDHVVPLCLPER | IPI00940181, IPI00798065, IPI00329555 | Isoform A of Coagulation factor VII | F7 |
| LMTQDCLQQSR | IPI00940181, IPI00798065, IPI00329555 | Isoform A of Coagulation factor VII | F7 |
| VAQVIIPSTYVPGTTNHDIALLR | IPI00940181, IPI00798065, IPI00329555 | Isoform A of Coagulation factor VII | F7 |
| VSQYIEWLQK | IPI00940181, IPI00798065, IPI00329555 | Isoform A of Coagulation factor VII | F7 |
| EYTNIFLK | IPI00942510, IPI00296176, IPI00930648 | Coagulation factor IX | F9 |
| FGSGYVSGWGR | IPI00942510, IPI00296176, IPI00930648 | Coagulation factor IX | F9 |
| FTIYNNMFCAGFHEGGR | IPI00942510, IPI00296176, IPI00930648 | Coagulation factor IX | F9 |
| IIPHHNYNAAINK | IPI00942510, IPI00296176, IPI00930648 | Coagulation factor IX | F9 |
| ITVVAGEHNIEETEHTEQK | IPI00942510, IPI00296176, IPI00930648 | Coagulation factor IX | F9 |
| SALVLQYLR | IPI00942510, IPI00296176, IPI00930648 | Coagulation factor IX | F9 |
| SCEPAVPFPCGR | IPI00942510, IPI00296176, IPI00930648 | Coagulation factor IX | F9 |
| VDAFCGGSIVNEK | IPI00942510, IPI00296176, IPI00930648 | Coagulation factor IX | F9 |
| VVCSCTEGYR | IPI00942510, IPI00296176, IPI00930648 | Coagulation factor IX | F9 |
| VVGGEDAKPGQFPWQVVLNGK | IPI00942510, IPI00296176, IPI00930648 | Coagulation factor IX | F9 |
| WIVTAAHCVETGVK | IPI00942510, IPI00296176, IPI00930648 | Coagulation factor IX | F9 |
| YVNWIK | IPI00942510 | Coagulation factor IX | F9 |
| DDINSYECWCPFGFEGK | IPI00296176 | Coagulation factor IX precursor | F9 |
| NCELDVTCNIK | IPI00296176 | Coagulation factor IX precursor | F9 |
| NCELDVTCNIKNGR | IPI00296176 | Coagulation factor IX precursor | F9 |
| EVGVGFATR | IPI00215746 | Fatty acid-binding protein, adipocyte | FABP4 |
| AHEHIFGMVLMNDWSAR | IPI00794773, IPI00793664, IPI00031708, IPI00794247 | Fumarylacetoacetase | FAH |
| ASSVVVSGTPIR | IPI00794773, IPI00793664, IPI00031708, IPI00794247 | Fumarylacetoacetase | FAH |
| GTKPIDLGNGQTR | IPI00031708 | Fumarylacetoacetase | FAH |
| VFLQNLLSVSQAR | IPI00794773, IPI00793664, IPI00031708, IPI00794247 | Fumarylacetoacetase | FAH |
| LSLLMAESLR | IPI00470607 | family with sequence similarity 20, member C | FAM20C |
| WEAINIFR | IPI00295461, IPI00375396 | Isoform 1 of Seprase | FAP |
| AITPPHPASQANIIFDITEGNLR | IPI00894529, IPI00413623, IPI00296537, IPI00889714, IPI00333852, IPI00893821, IPI00893588, IPI00889637, IPI00296534, IPI00889648, IPI00218803, IPI00894386, IPI00889740 | Isoform B of Fibulin-1 | FBLN1 |
| CLAFECPENYR | IPI00894529, IPI00413623, IPI00296537, IPI00889714, IPI00333852, IPI00893821, IPI00893588, IPI00889637, IPI00296534, IPI00889648, IPI00218803, IPI00894386, IPI00889740 | Isoform B of Fibulin-1 | FBLN1 |
| CVDVDECAPPAEPCGK | IPI00894529, IPI00413623, IPI00296537, IPI00889714, IPI00333852, IPI00893821, IPI00893588, IPI00889637, IPI00296534, IPI00889648, IPI00218803, IPI00894386, IPI00889740 | Isoform B of Fibulin-1 | FBLN1 |
| DCSLPYATESK | IPI00894529, IPI00413623, IPI00296537, IPI00889714, IPI00333852, IPI00893821, IPI00893588, IPI00889637, IPI00296534, IPI00889648, IPI00218803, IPI00894386, IPI00889740 | Isoform B of Fibulin-1 | FBLN1 |
| DIDECESGIHNCLPDFICQNTLGSFR | IPI00894529, IPI00413623, IPI00296537, IPI00889714, IPI00333852, IPI00893821, IPI00893588, IPI00889637, IPI00296534, IPI00889648, IPI00218803, IPI00894386, IPI00889740 | Isoform B of Fibulin-1 | FBLN1 |
| DSSCGTGYELTEDNSCK | IPI00894529, IPI00413623, IPI00296537, IPI00889714, IPI00333852, IPI00893821, IPI00893588, IPI00889637, IPI00296534, IPI00889648, IPI00218803, IPI00894386, IPI00889740 | Isoform B of Fibulin-1 | FBLN1 |
| EFTRPEEIIFLR | IPI00894529, IPI00413623, IPI00296537, IPI00889714, IPI00333852, IPI00893821, IPI00893588, IPI00889637, IPI00296534, IPI00889648, IPI00218803, IPI00894386, IPI00889740 | Isoform B of Fibulin-1 | FBLN1 |
| GYQLSDVDGVTCEDIDECALPTGGHICSYR | IPI00894529, IPI00413623, IPI00296537, IPI00889714, IPI00333852, IPI00893821, IPI00893588, IPI00889637, IPI00296534, IPI00889648, IPI00218803, IPI00894386, IPI00889740 | Isoform B of Fibulin-1 | FBLN1 |
| IIEVEEEQEDPYLNDR | IPI00894529, IPI00413623, IPI00296537, IPI00889714, IPI00333852, IPI00893821, IPI00893588, IPI00889637, IPI00296534, IPI00889648, IPI00218803, IPI00894386, IPI00889740 | Isoform B of Fibulin-1 | FBLN1 |
| ITYYHLSFPTNIQAPAVVFR | IPI00894529, IPI00413623, IPI00296537, IPI00889714, IPI00333852, IPI00893821, IPI00893588, IPI00889637, IPI00296534, IPI00889648, IPI00218803, IPI00894386, IPI00889740 | Isoform B of Fibulin-1 | FBLN1 |
| LCGHKCENTLGSYLCSCSVGFR | IPI00894529, IPI00413623, IPI00296537, IPI00889714, IPI00333852, IPI00893821, IPI00893588, IPI00889637, IPI00296534, IPI00889648, IPI00218803, IPI00894386, IPI00889740 | Isoform B of Fibulin-1 | FBLN1 |
| MCVDVNECQR | IPI00894529, IPI00413623, IPI00296537, IPI00889714, IPI00333852, IPI00893821, IPI00893588, IPI00889637, IPI00296534, IPI00889648, IPI00218803, IPI00894386, IPI00889740 | Isoform B of Fibulin-1 | FBLN1 |
| MGPSSAVPGDSMQLAITGGNEEGFFTTR | IPI00894529, IPI00413623, IPI00296537, IPI00889714, IPI00333852, IPI00893821, IPI00893588, IPI00889637, IPI00296534, IPI00889648, IPI00218803, IPI00894386, IPI00889740 | Isoform B of Fibulin-1 | FBLN1 |
| MVQEQCCHSQLEELHCATGISLANEQDR | IPI00894529, IPI00413623, IPI00296537, IPI00889714, IPI00333852, IPI00893821, IPI00893588, IPI00889637, IPI00296534, IPI00889648, IPI00218803, IPI00894386, IPI00889740 | Isoform B of Fibulin-1 | FBLN1 |
| SQETGDLDVGGLQETDK | IPI00894529, IPI00413623, IPI00296537, IPI00889714, IPI00333852, IPI00893821, IPI00893588, IPI00889637, IPI00296534, IPI00889648, IPI00218803, IPI00894386, IPI00889740 | Isoform B of Fibulin-1 | FBLN1 |
| SQETGDLDVGGLQETDKIIEVEEEQEDPYLNDR | IPI00894529, IPI00413623, IPI00296537, IPI00889714, IPI00333852, IPI00893821, IPI00893588, IPI00889637, IPI00296534, IPI00889648, IPI00218803, IPI00894386, IPI00889740 | Isoform B of Fibulin-1 | FBLN1 |
| TGYYFDGISR | IPI00894529, IPI00413623, IPI00296537, IPI00889714, IPI00333852, IPI00893821, IPI00893588, IPI00889637, IPI00296534, IPI00889648, IPI00218803, IPI00894386, IPI00889740 | Isoform B of Fibulin-1 | FBLN1 |
| APGWDPLCWDECR | IPI00936296, IPI00242956, IPI00936055, IPI00935210, IPI00940916, IPI00941166, IPI00888280 | IgGFc-binding protein | FCGBP |
| EGCVCDAGFVLSGDTCVPVGQCGCLHDDR | IPI00936296, IPI00242956, IPI00936055, IPI00935210, IPI00940916, IPI00941166, IPI00888280 | IgGFc-binding protein | FCGBP |
| NPQGPFATCQAVLSPSEYFR | IPI00936296, IPI00242956, IPI00936055, IPI00935210, IPI00940916, IPI00941166, IPI00888280 | IgGFc-binding protein | FCGBP |
| TVLSPVEPSCEGMQCAAGQR | IPI00936296, IPI00242956, IPI00936055, IPI00935210, IPI00940916, IPI00941166, IPI00888280 | IgGFc-binding protein | FCGBP |
| VAYDLVYYVR | IPI00936296, IPI00242956, IPI00936055, IPI00935210, IPI00940916, IPI00941166, IPI00888280 | IgGFc-binding protein | FCGBP |
| LEPPWINVLQEDSVTLTCQGAR | IPI00023505, IPI00943196, IPI00646445 | Isoform 1 of Low affinity immunoglobulin gamma Fc region receptor II-a | FCGR2A |
| AVVFLEPQWYR | IPI00640044, IPI00895917, IPI00795501, IPI00853533, IPI00937967, IPI00218834, IPI00023858 | Fc-gamma receptor IIIb | FCGR3B |
| AVVFLEPQWYSVLEK | IPI00640044, IPI00895917, IPI00795501, IPI00853533, IPI00937967, IPI00218834, IPI00023858 | Fc-gamma receptor IIIb | FCGR3B |
| WVFKEEDPIHLR | IPI00640044, IPI00895917, IPI00795501, IPI00853533, IPI00937967, IPI00218834, IPI00023858 | Fc-gamma receptor IIIb | FCGR3B |
| QGTWGGDWPEALAISQR | IPI00026646, IPI00943111 | IgG receptor FcRn large subunit p51 (Fragment) | FCGRT |
| ALPVFCDMDTEGGGWLVFQR | IPI00293925, IPI00419744 | Isoform 1 of Ficolin-3 precursor | FCN3 |
| LLGEVDHYQLALGK | IPI00293925, IPI00419744 | Isoform 1 of Ficolin-3 precursor | FCN3 |
| YGIDWASGR | IPI00293925, IPI00419744 | Isoform 1 of Ficolin-3 precursor | FCN3 |
| AQAVVGDLLELHCEAPR | IPI00168766, IPI00647675, IPI00921452, IPI00290135, IPI00641062 | Isoform 1 of Fc receptor-like protein 5 | FCRL5 |
| AIFYMNNPSR | IPI00553061, IPI00925428, IPI00552199, IPI00924616, IPI00743766, IPI00792024 | GUGU beta form | FETUB |
| ASSQWVVGPSYFVEYLIK | IPI00553061, IPI00925428, IPI00552199, IPI00924616, IPI00743766, IPI00792024 | GUGU beta form | FETUB |
| GGLGSLFYLTLDVLETDCHVLR | IPI00553061, IPI00925428, IPI00552199, IPI00924616, IPI00743766, IPI00792024 | GUGU beta form | FETUB |
| GSVQYLPDLDDK | IPI00553061, IPI00925428, IPI00552199, IPI00924616, IPI00743766, IPI00792024 | GUGU beta form | FETUB |
| GSVQYLPDLDDKNSQEK | IPI00553061, IPI00925428, IPI00552199, IPI00924616, IPI00743766, IPI00792024 | GUGU beta form | FETUB |
| LVVLPFPK | IPI00553061, IPI00925428, IPI00552199, IPI00924616, IPI00743766, IPI00792024 | GUGU beta form | FETUB |
| SQASSCSLQSSDSVPVGLCK | IPI00553061, IPI00925428, IPI00552199, IPI00924616, IPI00743766, IPI00792024 | GUGU beta form | FETUB |
| VNDAQEYR | IPI00553061, IPI00925428, IPI00552199, IPI00924616, IPI00743766, IPI00792024 | GUGU beta form | FETUB |
| IFFESVYGQCK | IPI00743766 | Uncharacterized protein FETUB | FETUB |
| ALTDMPQMR | IPI00029717, IPI00871469, IPI00021885, IPI00902755 | Isoform 1 of Fibrinogen alpha chain | FGA |
| DSHSLTTNIMEILR | IPI00029717, IPI00871469, IPI00021885, IPI00902755 | Isoform 1 of Fibrinogen alpha chain | FGA |
| EVDLKDYEDQQK | IPI00029717, IPI00871469, IPI00021885, IPI00902755 | Isoform 1 of Fibrinogen alpha chain | FGA |
| EVVTSEDGSDCPEAMDLGTLSGIGTLDGFR | IPI00029717, IPI00871469, IPI00021885, IPI00902755 | Isoform 1 of Fibrinogen alpha chain | FGA |
| GDSTFESK | IPI00029717, IPI00871469, IPI00021885, IPI00902755 | Isoform 1 of Fibrinogen alpha chain | FGA |
| GGSTSYGTGSETESPR | IPI00029717, IPI00871469, IPI00021885, IPI00902755 | Isoform 1 of Fibrinogen alpha chain | FGA |
| GLIDEVNQDFTNR | IPI00029717, IPI00871469, IPI00021885, IPI00902755 | Isoform 1 of Fibrinogen alpha chain | FGA |
| GSESGIFTNTK | IPI00029717, IPI00871469, IPI00021885, IPI00902755 | Isoform 1 of Fibrinogen alpha chain | FGA |
| HPDEAAFFDTASTGK | IPI00029717, IPI00871469, IPI00021885, IPI00902755 | Isoform 1 of Fibrinogen alpha chain | FGA |
| MADEAGSEADHEGTHSTK | IPI00029717, IPI00871469, IPI00021885, IPI00902755 | Isoform 1 of Fibrinogen alpha chain | FGA |
| MELERPGGNEITR | IPI00029717, IPI00871469, IPI00021885, IPI00902755 | Isoform 1 of Fibrinogen alpha chain | FGA |
| MKPVPDLVPGNFK | IPI00029717, IPI00871469, IPI00021885, IPI00902755 | Isoform 1 of Fibrinogen alpha chain | FGA |
| NNKDSHSLTTNIMEILR | IPI00029717, IPI00871469, IPI00021885, IPI00902755 | Isoform 1 of Fibrinogen alpha chain | FGA |
| NPSSAGSWNSGSSGPGSTGNR | IPI00029717, IPI00871469, IPI00021885, IPI00902755 | Isoform 1 of Fibrinogen alpha chain | FGA |
| NSLFEYQK | IPI00029717, IPI00871469, IPI00021885, IPI00902755 | Isoform 1 of Fibrinogen alpha chain | FGA |
| RLEVDIDIK | IPI00029717, IPI00871469, IPI00021885, IPI00902755 | Isoform 1 of Fibrinogen alpha chain | FGA |
| TFPGFFSPMLGEFVSETESR | IPI00029717, IPI00871469, IPI00021885, IPI00902755 | Isoform 1 of Fibrinogen alpha chain | FGA |
| TVIGPDGHK | IPI00029717, IPI00871469, IPI00021885, IPI00902755 | Isoform 1 of Fibrinogen alpha chain | FGA |
| TVIGPDGHKEVTK | IPI00029717, IPI00871469, IPI00021885, IPI00902755 | Isoform 1 of Fibrinogen alpha chain | FGA |
| VQHIQLLQK | IPI00029717, IPI00871469, IPI00021885, IPI00902755 | Isoform 1 of Fibrinogen alpha chain | FGA |
| GFGSLNDEGEGEFWLGNDYLHLLTQR | IPI00021885 | Isoform 1 of Fibrinogen alpha chain precursor | FGA |
| AHYGGFTVQNEANK | IPI00298497 | Fibrinogen beta chain precursor | FGB |
| DNENVVNEYSSELEK | IPI00298497 | Fibrinogen beta chain precursor | FGB |
| ECEEIIR | IPI00298497 | Fibrinogen beta chain precursor | FGB |
| EDGGGWWYNR | IPI00298497 | Fibrinogen beta chain precursor | FGB |
| EEAPSLRPAPPPISGGGYR | IPI00298497, IPI00816687 | Fibrinogen beta chain precursor | FGB |
| GGETSEMYLIQPDSSVKPYR | IPI00298497 | Fibrinogen beta chain precursor | FGB |
| GSWYSMR | IPI00298497 | Fibrinogen beta chain precursor | FGB |
| HGTDDGVVWMNWK | IPI00298497 | Fibrinogen beta chain precursor | FGB |
| HQLYIDETVNSNIPTNLR | IPI00298497 | Fibrinogen beta chain precursor | FGB |
| IRPFFPQQ | IPI00298497 | Fibrinogen beta chain precursor | FGB |
| KGGETSEMYLIQPDSSVKPYR | IPI00298497 | Fibrinogen beta chain precursor | FGB |
| LESDVSAQMEYCR | IPI00298497 | Fibrinogen beta chain precursor | FGB |
| MGPTELLIEMEDWK | IPI00298497 | Fibrinogen beta chain precursor | FGB |
| NSVDELNNNVEAVSQTSSSSFQYMYLLK | IPI00298497, IPI00816687 | Fibrinogen beta chain precursor | FGB |
| NYCGLPGEYWLGNDK | IPI00298497 | Fibrinogen beta chain precursor | FGB |
| QGFGNVATNTDGK | IPI00298497 | Fibrinogen beta chain precursor | FGB |
| REEAPSLRPAPPPISGGGYR | IPI00298497, IPI00816687 | Fibrinogen beta chain precursor | FGB |
| SILENLR | IPI00298497 | Fibrinogen beta chain precursor | FGB |
| TMTIHNGMFFSTYDR | IPI00298497 | Fibrinogen beta chain precursor | FGB |
| TMTIHNGMFFSTYDRDNDGWLTSDPR | IPI00298497 | Fibrinogen beta chain precursor | FGB |
| TPCTVSCNIPVVSGK | IPI00298497 | Fibrinogen beta chain precursor | FGB |
| TPCTVSCNIPVVSGKECEEIIR | IPI00298497 | Fibrinogen beta chain precursor | FGB |
| VYCDMNTENGGWTVIQNR | IPI00298497 | Fibrinogen beta chain precursor | FGB |
| YYWGGQYTWDMAK | IPI00298497 | Fibrinogen beta chain precursor | FGB |
| AIQLTYNPDESSKPNMIDAATLK | IPI00219713, IPI00411626, IPI00877625, IPI00021891, IPI00877703, IPI00877792 | Isoform Gamma-B of Fibrinogen gamma chain | FGG |
| ANQQFLVYCEIDGSGNGWTVFQK | IPI00219713, IPI00411626, IPI00877625, IPI00021891, IPI00877703, IPI00877792 | Isoform Gamma-B of Fibrinogen gamma chain | FGG |
| ASTPNGYDNGIIWATWK | IPI00219713, IPI00411626, IPI00877625, IPI00021891, IPI00877703, IPI00877792 | Isoform Gamma-B of Fibrinogen gamma chain | FGG |
| DCQDIANK | IPI00219713, IPI00411626, IPI00877625, IPI00021891, IPI00877703, IPI00877792 | Isoform Gamma-B of Fibrinogen gamma chain | FGG |
| DNCCILDER | IPI00219713, IPI00411626, IPI00877625, IPI00021891, IPI00877703, IPI00877792 | Isoform Gamma-B of Fibrinogen gamma chain | FGG |
| FGSYCPTTCGIADFLSTYQTK | IPI00219713, IPI00411626, IPI00877625, IPI00021891, IPI00877703, IPI00877792 | Isoform Gamma-B of Fibrinogen gamma chain | FGG |
| IHLISTQSAIPYALR | IPI00219713, IPI00411626, IPI00877625, IPI00021891, IPI00877703, IPI00877792 | Isoform Gamma-B of Fibrinogen gamma chain | FGG |
| LDGSVDFK | IPI00219713, IPI00411626, IPI00877625, IPI00021891, IPI00877703, IPI00877792 | Isoform Gamma-B of Fibrinogen gamma chain | FGG |
| LTIGEGQQHHLGGAK | IPI00219713, IPI00411626, IPI00877625, IPI00021891, IPI00877703, IPI00877792 | Isoform Gamma-B of Fibrinogen gamma chain | FGG |
| LTYAYFAGGDAGDAFDGFDFGDDPSDK | IPI00219713, IPI00411626, IPI00877625, IPI00021891, IPI00877703, IPI00877792 | Isoform Gamma-B of Fibrinogen gamma chain | FGG |
| TSTADYAMFK | IPI00219713, IPI00411626, IPI00877625, IPI00021891, IPI00877703, IPI00877792 | Isoform Gamma-B of Fibrinogen gamma chain | FGG |
| VAQLEAQCQEPCKDTVQIHDITGK | IPI00219713, IPI00411626, IPI00877625, IPI00021891, IPI00877703, IPI00877792 | Isoform Gamma-B of Fibrinogen gamma chain | FGG |
| VELEDWNGR | IPI00219713, IPI00411626, IPI00877625, IPI00021891, IPI00877703, IPI00877792 | Isoform Gamma-B of Fibrinogen gamma chain | FGG |
| VGPEADKYR | IPI00219713, IPI00411626, IPI00877625, IPI00021891, IPI00877703, IPI00877792 | Isoform Gamma-B of Fibrinogen gamma chain | FGG |
| YLQEIYNSNNQK | IPI00219713, IPI00411626, IPI00877625, IPI00021891, IPI00877703, IPI00877792 | Isoform Gamma-B of Fibrinogen gamma chain | FGG |
| LAELFER | IPI00396025 | hypothetical protein LOC254122 | FLJ30934 |
| FNEEHIPDSPFVVPVASPSGDAR | IPI00644576, IPI00657767, IPI00909642, IPI00552858, IPI00333541, IPI00302592, IPI00910863 | Filamin A, alpha | FLNA |
| IAGPGLGSGVR | IPI00382697, IPI00940093, IPI00382698, IPI00289334, IPI00900293, IPI00382699, IPI00382700, IPI00382696, IPI00477536 | Filamin B | FLNB |
| DDKESVPISDTIIPAVPPPTDLR | IPI00339228, IPI00022418, IPI00556632, IPI00873210, IPI00867588, IPI00855777, IPI00922213, IPI00479723, IPI00414283, IPI00339223, IPI00339226, IPI00411462, IPI00339319, IPI00845263, IPI00339227, IPI00339225, IPI00856050, IPI00339224, IPI00855785 | Isoform 1 of Fibronectin | FN1 |
| DSMIWDCTCIGAGR | IPI00339228, IPI00022418, IPI00556632, IPI00873210, IPI00867588, IPI00855777, IPI00922213, IPI00479723, IPI00414283, IPI00339223, IPI00339226, IPI00411462, IPI00339319, IPI00845263, IPI00339227, IPI00339225, IPI00856050, IPI00339224, IPI00855785 | Isoform 1 of Fibronectin | FN1 |
| EESPLLIGQQSTVSDVPR | IPI00339228, IPI00022418, IPI00556632, IPI00873210, IPI00867588, IPI00855777, IPI00922213, IPI00479723, IPI00414283, IPI00339223, IPI00339226, IPI00411462, IPI00339319, IPI00845263, IPI00339227, IPI00339225, IPI00856050, IPI00339224, IPI00855785 | Isoform 1 of Fibronectin | FN1 |
| EYLGAICSCTCFGGQR | IPI00339228, IPI00022418, IPI00556632, IPI00873210, IPI00867588, IPI00855777, IPI00922213, IPI00479723, IPI00414283, IPI00339223, IPI00339226, IPI00411462, IPI00339319, IPI00845263, IPI00339227, IPI00339225, IPI00856050, IPI00339224, IPI00855785 | Isoform 1 of Fibronectin | FN1 |
| FGFCPMAAHEEICTTNEGVMYR | IPI00339228, IPI00022418, IPI00556632, IPI00873210, IPI00867588, IPI00855777, IPI00922213, IPI00479723, IPI00414283, IPI00339223, IPI00339226, IPI00411462, IPI00339319, IPI00845263, IPI00339227, IPI00339225, IPI00856050, IPI00339224, IPI00855785 | Isoform 1 of Fibronectin | FN1 |
| FLATTPNSLLVSWQPPR | IPI00339228, IPI00022418, IPI00556632, IPI00873210, IPI00867588, IPI00855777, IPI00922213, IPI00479723, IPI00414283, IPI00339223, IPI00339226, IPI00411462, IPI00339319, IPI00845263, IPI00339227, IPI00339225, IPI00856050, IPI00339224, IPI00855785 | Isoform 1 of Fibronectin | FN1 |
| FTQVTPTSLSAQWTPPNVQLTGYR | IPI00339228, IPI00022418, IPI00556632, IPI00873210, IPI00867588, IPI00855777, IPI00922213, IPI00479723, IPI00414283, IPI00339223, IPI00339226, IPI00411462, IPI00339319, IPI00845263, IPI00339227, IPI00339225, IPI00856050, IPI00339224, IPI00855785 | Isoform 1 of Fibronectin | FN1 |
| GATYNIIVEALKDQQR | IPI00339228, IPI00022418, IPI00556632, IPI00873210, IPI00867588, IPI00855777, IPI00922213, IPI00479723, IPI00414283, IPI00339223, IPI00339226, IPI00411462, IPI00339319, IPI00845263, IPI00339227, IPI00339225, IPI00856050, IPI00339224, IPI00855785 | Isoform 1 of Fibronectin | FN1 |
| GDSPASSKPISINYR | IPI00339228, IPI00022418, IPI00556632, IPI00873210, IPI00867588, IPI00855777, IPI00922213, IPI00479723, IPI00414283, IPI00339223, IPI00339226, IPI00411462, IPI00339319, IPI00845263, IPI00339227, IPI00339225, IPI00856050, IPI00339224, IPI00855785 | Isoform 1 of Fibronectin | FN1 |
| GEWTCIAYSQLR | IPI00339228, IPI00022418, IPI00556632, IPI00873210, IPI00867588, IPI00855777, IPI00922213, IPI00479723, IPI00414283, IPI00339223, IPI00339226, IPI00411462, IPI00339319, IPI00845263, IPI00339227, IPI00339225, IPI00856050, IPI00339224, IPI00855785 | Isoform 1 of Fibronectin | FN1 |
| GFNCESKPEAEETCFDK | IPI00339228, IPI00022418, IPI00556632, IPI00873210, IPI00867588, IPI00855777, IPI00922213, IPI00479723, IPI00414283, IPI00339223, IPI00339226, IPI00411462, IPI00339319, IPI00845263, IPI00339227, IPI00339225, IPI00856050, IPI00339224, IPI00855785 | Isoform 1 of Fibronectin | FN1 |
| GNLLQCICTGNGR | IPI00339228, IPI00022418, IPI00556632, IPI00873210, IPI00867588, IPI00855777, IPI00922213, IPI00479723, IPI00414283, IPI00339223, IPI00339226, IPI00411462, IPI00339319, IPI00845263, IPI00339227, IPI00339225, IPI00856050, IPI00339224, IPI00855785 | Isoform 1 of Fibronectin | FN1 |
| HYQINQQWER | IPI00339228, IPI00022418, IPI00556632, IPI00873210, IPI00867588, IPI00855777, IPI00922213, IPI00479723, IPI00414283, IPI00339223, IPI00339226, IPI00411462, IPI00339319, IPI00845263, IPI00339227, IPI00339225, IPI00856050, IPI00339224, IPI00855785 | Isoform 1 of Fibronectin | FN1 |
| ITYGETGGNSPVQEFTVPGSK | IPI00339228, IPI00022418, IPI00556632, IPI00873210, IPI00867588, IPI00855777, IPI00922213, IPI00479723, IPI00414283, IPI00339223, IPI00339226, IPI00411462, IPI00339319, IPI00845263, IPI00339227, IPI00339225, IPI00856050, IPI00339224, IPI00855785 | Isoform 1 of Fibronectin | FN1 |
| IYLYTLNDNAR | IPI00339228, IPI00022418, IPI00556632, IPI00873210, IPI00867588, IPI00855777, IPI00922213, IPI00479723, IPI00414283, IPI00339223, IPI00339226, IPI00411462, IPI00339319, IPI00845263, IPI00339227, IPI00339225, IPI00856050, IPI00339224, IPI00855785 | Isoform 1 of Fibronectin | FN1 |
| NSITLTNLTPGTEYVVSIVALNGR | IPI00339228, IPI00022418, IPI00556632, IPI00873210, IPI00867588, IPI00855777, IPI00922213, IPI00479723, IPI00414283, IPI00339223, IPI00339226, IPI00411462, IPI00339319, IPI00845263, IPI00339227, IPI00339225, IPI00856050, IPI00339224, IPI00855785 | Isoform 1 of Fibronectin | FN1 |
| NTFAEVTGLSPGVTYYFK | IPI00339228, IPI00022418, IPI00556632, IPI00873210, IPI00867588, IPI00855777, IPI00922213, IPI00479723, IPI00414283, IPI00339223, IPI00339226, IPI00411462, IPI00339319, IPI00845263, IPI00339227, IPI00339225, IPI00856050, IPI00339224, IPI00855785 | Isoform 1 of Fibronectin | FN1 |
| QAQQMVQPQSPVAVSQSKPGCYDNGK | IPI00339228, IPI00022418, IPI00556632, IPI00873210, IPI00867588, IPI00855777, IPI00922213, IPI00479723, IPI00414283, IPI00339223, IPI00339226, IPI00411462, IPI00339319, IPI00845263, IPI00339227, IPI00339225, IPI00856050, IPI00339224, IPI00855785 | Isoform 1 of Fibronectin | FN1 |
| QGENGQMMSCTCLGNGK | IPI00339228, IPI00022418, IPI00556632, IPI00873210, IPI00867588, IPI00855777, IPI00922213, IPI00479723, IPI00414283, IPI00339223, IPI00339226, IPI00411462, IPI00339319, IPI00845263, IPI00339227, IPI00339225, IPI00856050, IPI00339224, IPI00855785 | Isoform 1 of Fibronectin | FN1 |
| QYNVGPSVSK | IPI00339228, IPI00022418, IPI00556632, IPI00873210, IPI00867588, IPI00855777, IPI00922213, IPI00479723, IPI00414283, IPI00339223, IPI00339226, IPI00411462, IPI00339319, IPI00845263, IPI00339227, IPI00339225, IPI00856050, IPI00339224, IPI00855785 | Isoform 1 of Fibronectin | FN1 |
| RPGGEPSPEGTTGQSYNQYSQR | IPI00339228, IPI00022418, IPI00556632, IPI00873210, IPI00867588, IPI00855777, IPI00922213, IPI00479723, IPI00414283, IPI00339223, IPI00339226, IPI00411462, IPI00339319, IPI00845263, IPI00339227, IPI00339225, IPI00856050, IPI00339224, IPI00855785 | Isoform 1 of Fibronectin | FN1 |
| STATISGLKPGVDYTITVYAVTGR | IPI00339228, IPI00022418, IPI00556632, IPI00873210, IPI00867588, IPI00855777, IPI00922213, IPI00479723, IPI00414283, IPI00339223, IPI00339226, IPI00411462, IPI00339319, IPI00845263, IPI00339227, IPI00339225, IPI00856050, IPI00339224, IPI00855785 | Isoform 1 of Fibronectin | FN1 |
| TETITGFQVDAVPANGQTPIQR | IPI00339228, IPI00022418, IPI00556632, IPI00873210, IPI00867588, IPI00855777, IPI00922213, IPI00479723, IPI00414283, IPI00339223, IPI00339226, IPI00411462, IPI00339319, IPI00845263, IPI00339227, IPI00339225, IPI00856050, IPI00339224, IPI00855785 | Isoform 1 of Fibronectin | FN1 |
| TKTETITGFQVDAVPANGQTPIQR | IPI00339228, IPI00022418, IPI00556632, IPI00873210, IPI00867588, IPI00855777, IPI00922213, IPI00479723, IPI00414283, IPI00339223, IPI00339226, IPI00411462, IPI00339319, IPI00845263, IPI00339227, IPI00339225, IPI00856050, IPI00339224, IPI00855785 | Isoform 1 of Fibronectin | FN1 |
| TYLGNALVCTCYGGSR | IPI00339228, IPI00022418, IPI00556632, IPI00873210, IPI00867588, IPI00855777, IPI00922213, IPI00479723, IPI00414283, IPI00339223, IPI00339226, IPI00411462, IPI00339319, IPI00845263, IPI00339227, IPI00339225, IPI00856050, IPI00339224, IPI00855785 | Isoform 1 of Fibronectin | FN1 |
| VDVIPVNLPGEHGQR | IPI00339228, IPI00022418, IPI00556632, IPI00873210, IPI00867588, IPI00855777, IPI00922213, IPI00479723, IPI00414283, IPI00339223, IPI00339226, IPI00411462, IPI00339319, IPI00845263, IPI00339227, IPI00339225, IPI00856050, IPI00339224, IPI00855785 | Isoform 1 of Fibronectin | FN1 |
| VPGTSTSATLTGLTR | IPI00339228, IPI00022418, IPI00556632, IPI00873210, IPI00867588, IPI00855777, IPI00922213, IPI00479723, IPI00414283, IPI00339223, IPI00339226, IPI00411462, IPI00339319, IPI00845263, IPI00339227, IPI00339225, IPI00856050, IPI00339224, IPI00855785 | Isoform 1 of Fibronectin | FN1 |
| VTDATETTITISWR | IPI00339228, IPI00022418, IPI00556632, IPI00873210, IPI00867588, IPI00855777, IPI00922213, IPI00479723, IPI00414283, IPI00339223, IPI00339226, IPI00411462, IPI00339319, IPI00845263, IPI00339227, IPI00339225, IPI00856050, IPI00339224, IPI00855785 | Isoform 1 of Fibronectin | FN1 |
| VTWAPPPSIDLTNFLVR | IPI00339228, IPI00022418, IPI00556632, IPI00873210, IPI00867588, IPI00855777, IPI00922213, IPI00479723, IPI00414283, IPI00339223, IPI00339226, IPI00411462, IPI00339319, IPI00845263, IPI00339227, IPI00339225, IPI00856050, IPI00339224, IPI00855785 | Isoform 1 of Fibronectin | FN1 |
| VVTPLSPPTNLHLEANPDTGVLTVSWER | IPI00339228, IPI00022418, IPI00556632, IPI00873210, IPI00867588, IPI00855777, IPI00922213, IPI00479723, IPI00414283, IPI00339223, IPI00339226, IPI00411462, IPI00339319, IPI00845263, IPI00339227, IPI00339225, IPI00856050, IPI00339224, IPI00855785 | Isoform 1 of Fibronectin | FN1 |
| WCGTTQNYDADQK | IPI00339228, IPI00022418, IPI00556632, IPI00873210, IPI00867588, IPI00855777, IPI00922213, IPI00479723, IPI00414283, IPI00339223, IPI00339226, IPI00411462, IPI00339319, IPI00845263, IPI00339227, IPI00339225, IPI00856050, IPI00339224, IPI00855785 | Isoform 1 of Fibronectin | FN1 |
| WLPSSSPVTGYR | IPI00339228, IPI00022418, IPI00556632, IPI00873210, IPI00867588, IPI00855777, IPI00922213, IPI00479723, IPI00414283, IPI00339223, IPI00339226, IPI00411462, IPI00339319, IPI00845263, IPI00339227, IPI00339225, IPI00856050, IPI00339224, IPI00855785 | Isoform 1 of Fibronectin | FN1 |
| YSFCTDHTVLVQTR | IPI00339228, IPI00022418, IPI00556632, IPI00873210, IPI00867588, IPI00855777, IPI00922213, IPI00479723, IPI00414283, IPI00339223, IPI00339226, IPI00411462, IPI00339319, IPI00845263, IPI00339227, IPI00339225, IPI00856050, IPI00339224, IPI00855785 | Isoform 1 of Fibronectin | FN1 |
| VTMASAFR | IPI00513845, IPI00921807 | fibronectin type III and SPRY domain containing 1-like isoform 1 | FSD1NL |
| CALEDETYADGAETEVDCNR | IPI00029723, IPI00908368 | Follistatin-related protein 1 | FSTL1 |
| LSFQEFLK | IPI00029723, IPI00908368 | Follistatin-related protein 1 | FSTL1 |
| TDPHLCDFLETHFLDEEVK | IPI00893729, IPI00375676 | Ferritin light chain | FTL |
| ALFQDIK | IPI00375676 | Ferritin light polypeptide variant | FTL |
| LGGPEAGLGEYLFER | IPI00375676 | Ferritin light polypeptide variant | FTL |
| FFHPEEWADLFQAAGAK | IPI00385751, IPI00843910 | Tissue alpha-L-fucosidase | FUCA1 |
| DNYPPGFSYADFGPQFTAR | IPI00843910 | Tissue alpha-L-fucosidase precursor | FUCA1 |
| FFNANQWADIFQASGAK | IPI00012440, IPI00385460 | Plasma alpha-L-fucosidase | FUCA2 |
| LVINGNPITIFQER | IPI00794605, IPI00788737, IPI00789134, IPI00793922, IPI00797221, IPI00219018, IPI00795257, IPI00795622 | Glyceraldehyde-3-phosphate dehydrogenase | GAPDH |
| VIHDNFGIVEGLMTTVHAITATQK | IPI00794605, IPI00788737, IPI00789134, IPI00793922, IPI00797221, IPI00219018, IPI00795257, IPI00795622 | Glyceraldehyde-3-phosphate dehydrogenase | GAPDH |
| VPTANVSVVDLTCR | IPI00794605, IPI00788737, IPI00789134, IPI00793922, IPI00797221, IPI00219018, IPI00795257, IPI00795622 | Glyceraldehyde-3-phosphate dehydrogenase | GAPDH |
| TCQDIDECADSEACGEAR | IPI00032532, IPI00412412, IPI00412410 | Isoform 2 of Growth arrest-specific protein 6 | GAS6 |
| EDFTSLSLVLYSR | IPI00555812, IPI00742696 | Vitamin D-binding protein precursor | GC |
| EFSHLGKEDFTSLSLVLYSR | IPI00555812, IPI00742696 | Vitamin D-binding protein precursor | GC |
| EVVSLTEACCAEGADPDCYDTR | IPI00555812, IPI00742696 | Vitamin D-binding protein precursor | GC |
| GQELCADYSENTFTEYK | IPI00555812, IPI00742696 | Vitamin D-binding protein precursor | GC |
| HQPQEFPTYVEPTNDEICEAFR | IPI00555812, IPI00742696 | Vitamin D-binding protein precursor | GC |
| KFPSGTFEQVSQLVK | IPI00555812, IPI00742696 | Vitamin D-binding protein precursor | GC |
| RTHLPEVFLSK | IPI00555812, IPI00742696 | Vitamin D-binding protein precursor | GC |
| SCESNSPFPVHPGTAECCTK | IPI00555812, IPI00742696 | Vitamin D-binding protein precursor | GC |
| SDFASNCCSINSPPLYCDSEIDAELK | IPI00555812, IPI00742696 | Vitamin D-binding protein precursor | GC |
| SLGECCDVEDSTTCFNAK | IPI00555812, IPI00742696 | Vitamin D-binding protein precursor | GC |
| SYLSMVGSCCTSASPTVCFLK | IPI00555812, IPI00742696 | Vitamin D-binding protein precursor | GC |
| THLPEVFLSK | IPI00555812, IPI00742696 | Vitamin D-binding protein precursor | GC |
| VCSQYAAYGEK | IPI00555812, IPI00742696 | Vitamin D-binding protein precursor | GC |
| VMDKYTFELSR | IPI00555812, IPI00742696 | Vitamin D-binding protein precursor | GC |
| VPTADLEDVLPLAEDITNILSK | IPI00555812, IPI00742696 | Vitamin D-binding protein precursor | GC |
| FFNVLTTNTDGK | IPI00023728 | Gamma-glutamyl hydrolase precursor | GGH |
| IEFISTMEGYK | IPI00023728 | Gamma-glutamyl hydrolase precursor | GGH |
| LDLTEKDYEILFK | IPI00023728 | Gamma-glutamyl hydrolase precursor | GGH |
| NLDGISHAPNAVK | IPI00023728 | Gamma-glutamyl hydrolase precursor | GGH |
| SINGILFPGGSVDLR | IPI00023728 | Gamma-glutamyl hydrolase precursor | GGH |
| TAFYLAEFFVNEAR | IPI00023728 | Gamma-glutamyl hydrolase precursor | GGH |
| YYIAASYVK | IPI00023728 | Gamma-glutamyl hydrolase precursor | GGH |
| DLLGETLAQLIR | IPI00339373, IPI00002243, IPI00877887, IPI00855794 | Isoform 1 of Gamma-glutamyltransferase 5 | GGTLA1 |
| NLGPIQLFYTR | IPI00410327, IPI00410328, IPI00410329, IPI00023599 | Isoform 1 of Growth hormone receptor precursor | GHR |
| DCIGGCSDLVSLQQSGELLTR | IPI00219025 | Glutaredoxin-1 | GLRX |
| SEFVVPDLELPSWLTTGNYR | IPI00018236 | Ganglioside GM2 activator precursor | GM2A |
| QWDQVEQDLADELITPQGHEK | IPI00000137, IPI00382750 | N-acetylglucosamine-1-phosphotransferase subunit gamma precursor | GNPTG |
| TLFEDAGYLK | IPI00000137, IPI00382750 | N-acetylglucosamine-1-phosphotransferase subunit gamma precursor | GNPTG |
| TPEENEPTQLEGGPDSLGFETLENCR | IPI00000137, IPI00382750 | N-acetylglucosamine-1-phosphotransferase subunit gamma precursor | GNPTG |
| FLFPFFDSAYQGFASGNLER | IPI00922421, IPI00219029 | Aspartate aminotransferase, cytoplasmic | GOT1 |
| GLGELQELYLK | IPI00748955, IPI00943099 | Platelet glycoprotein Ib alpha chain | GP1BA |
| LTSLPLGALR | IPI00748955, IPI00943099 | Platelet glycoprotein Ib alpha chain | GP1BA |
| LVSLDSGLLNSLGALTELQFHR | IPI00027410 | Platelet glycoprotein V precursor | GP5 |
| MVLLEQLFLDHNALR | IPI00027410 | Platelet glycoprotein V precursor | GP5 |
| ALEFLQLHNGR | IPI00376358, IPI00299503 | Isoform 1 of Phosphatidylinositol-glycan-specific phospholipase D precursor | GPLD1 |
| AQYVLISPEASSR | IPI00299503 | Isoform 1 of Phosphatidylinositol-glycan-specific phospholipase D precursor | GPLD1 |
| ELLLEHQDAYQAGIVFPDCFYPSICK | IPI00376358, IPI00299503 | Isoform 1 of Phosphatidylinositol-glycan-specific phospholipase D precursor | GPLD1 |
| FGSSLITVR | IPI00299503 | Isoform 1 of Phosphatidylinositol-glycan-specific phospholipase D precursor | GPLD1 |
| IADVTSGLIGGEDGR | IPI00299503 | Isoform 1 of Phosphatidylinositol-glycan-specific phospholipase D precursor | GPLD1 |
| ILEGFQPSGR | IPI00299503 | Isoform 1 of Phosphatidylinositol-glycan-specific phospholipase D precursor | GPLD1 |
| SWITPCPEEK | IPI00299503 | Isoform 1 of Phosphatidylinositol-glycan-specific phospholipase D precursor | GPLD1 |
| TLLLVGSPTWK | IPI00299503 | Isoform 1 of Phosphatidylinositol-glycan-specific phospholipase D precursor | GPLD1 |
| TMFIGGSQLSQK | IPI00299503 | Isoform 1 of Phosphatidylinositol-glycan-specific phospholipase D precursor | GPLD1 |
| VAFLTVTLHQGGATR | IPI00299503 | Isoform 1 of Phosphatidylinositol-glycan-specific phospholipase D precursor | GPLD1 |
| VYLIYGNDLGLPPVDLDLDKEAHR | IPI00299503 | Isoform 1 of Phosphatidylinositol-glycan-specific phospholipase D precursor | GPLD1 |
| ISVVIQNILR | IPI00651770, IPI00423340, IPI00423342, IPI00217481 | G protein-coupled receptor 126 beta 2 precursor | GPR126 |
| FLVGPDGIPIMR | IPI00026199 | Glutathione peroxidase 3 precursor | GPX3 |
| FYTFLK | IPI00026199 | Glutathione peroxidase 3 precursor | GPX3 |
| LFWEPMK | IPI00026199 | Glutathione peroxidase 3 precursor | GPX3 |
| MDILSYMR | IPI00026199 | Glutathione peroxidase 3 precursor | GPX3 |
| NSCPPTSELLGTSDR | IPI00026199 | Glutathione peroxidase 3 precursor | GPX3 |
| QEPGENSEILPTLK | IPI00026199 | Glutathione peroxidase 3 precursor | GPX3 |
| TTVSNVK | IPI00026199 | Glutathione peroxidase 3 precursor | GPX3 |
| WNFEK | IPI00026199 | Glutathione peroxidase 3 precursor | GPX3 |
| YVRPGGGFVPNFQLFEK | IPI00026199 | Glutathione peroxidase 3 precursor | GPX3 |
| AGALNSNDAFVLK | IPI00796316, IPI00026314, IPI00647556, IPI00513782, IPI00646773, IPI00641047, IPI00377087 | Isoform 1 of Gelsolin precursor | GSN |
| AGKEPGLQIWR | IPI00796316, IPI00026314, IPI00647556, IPI00513782, IPI00646773, IPI00641047, IPI00377087 | Isoform 1 of Gelsolin precursor | GSN |
| AQPVQVAEGSEPDGFWEALGGK | IPI00796316, IPI00026314, IPI00647556, IPI00513782, IPI00646773, IPI00641047, IPI00377087 | Isoform 1 of Gelsolin precursor | GSN |
| AQPVQVAEGSEPDGFWEALGGKAAYR | IPI00796316, IPI00026314, IPI00647556, IPI00513782, IPI00646773, IPI00641047, IPI00377087 | Isoform 1 of Gelsolin precursor | GSN |
| DSQEEEKTEALTSAK | IPI00796316, IPI00026314, IPI00647556, IPI00513782, IPI00646773, IPI00641047, IPI00377087 | Isoform 1 of Gelsolin precursor | GSN |
| EPAHLMSLFGGKPMIIYK | IPI00796316, IPI00026314, IPI00647556, IPI00513782, IPI00646773, IPI00641047, IPI00377087 | Isoform 1 of Gelsolin precursor | GSN |
| EPGLQIWR | IPI00796316, IPI00026314, IPI00647556, IPI00513782, IPI00646773, IPI00641047, IPI00377087 | Isoform 1 of Gelsolin precursor | GSN |
| EVQGFESATFLGYFK | IPI00796316, IPI00026314, IPI00647556, IPI00513782, IPI00646773, IPI00641047, IPI00377087 | Isoform 1 of Gelsolin precursor | GSN |
| FDLVPVPTNLYGDFFTGDAYVILK | IPI00796316, IPI00026314, IPI00647556, IPI00513782, IPI00646773, IPI00641047, IPI00377087 | Isoform 1 of Gelsolin precursor | GSN |
| GGVASGFK | IPI00796316, IPI00026314, IPI00647556, IPI00513782, IPI00646773, IPI00641047, IPI00377087 | Isoform 1 of Gelsolin precursor | GSN |
| HVVPNEVVVQR | IPI00796316, IPI00026314, IPI00647556, IPI00513782, IPI00646773, IPI00641047, IPI00377087 | Isoform 1 of Gelsolin precursor | GSN |
| IEGSNKVPVDPATYGQFYGGDSYIILYNYR | IPI00796316, IPI00026314, IPI00647556, IPI00513782, IPI00646773, IPI00641047, IPI00377087 | Isoform 1 of Gelsolin precursor | GSN |
| NWRDPDQTDGLGLSYLSSHIANVER | IPI00796316, IPI00026314, IPI00647556, IPI00513782, IPI00646773, IPI00641047, IPI00377087 | Isoform 1 of Gelsolin precursor | GSN |
| QTQVSVLPEGGETPLFK | IPI00796316, IPI00026314, IPI00647556, IPI00513782, IPI00646773, IPI00641047, IPI00377087 | Isoform 1 of Gelsolin precursor | GSN |
| RTPITVVK | IPI00796316, IPI00026314, IPI00647556, IPI00513782, IPI00646773, IPI00641047, IPI00377087 | Isoform 1 of Gelsolin precursor | GSN |
| SEDCFILDHGK | IPI00796316, IPI00026314, IPI00647556, IPI00513782, IPI00646773, IPI00641047, IPI00377087 | Isoform 1 of Gelsolin precursor | GSN |
| SEDCFILDHGKDGK | IPI00796316, IPI00026314, IPI00647556, IPI00513782, IPI00646773, IPI00641047, IPI00377087 | Isoform 1 of Gelsolin precursor | GSN |
| TASDFITK | IPI00796316, IPI00026314, IPI00647556, IPI00513782, IPI00646773, IPI00641047, IPI00377087 | Isoform 1 of Gelsolin precursor | GSN |
| TEALTSAK | IPI00796316, IPI00026314, IPI00647556, IPI00513782, IPI00646773, IPI00641047, IPI00377087 | Isoform 1 of Gelsolin precursor | GSN |
| TGAQELLR | IPI00796316, IPI00026314, IPI00647556, IPI00513782, IPI00646773, IPI00641047, IPI00377087 | Isoform 1 of Gelsolin precursor | GSN |
| TPSAAYLWVGTGASEAEK | IPI00796316, IPI00026314, IPI00647556, IPI00513782, IPI00646773, IPI00641047, IPI00377087 | Isoform 1 of Gelsolin precursor | GSN |
| VPEARPNSMVVEHPEFLK | IPI00026314 | Isoform 1 of Gelsolin precursor | GSN |
| VPFDAATLHTSTAMAAQHGMDDDGTGQK | IPI00796316, IPI00026314, IPI00647556, IPI00513782, IPI00646773, IPI00641047, IPI00377087 | Isoform 1 of Gelsolin precursor | GSN |
| VPVDPATYGQFYGGDSYIILYNYR | IPI00796316, IPI00026314, IPI00647556, IPI00513782, IPI00646773, IPI00641047, IPI00377087 | Isoform 1 of Gelsolin precursor | GSN |
| VSNGAGTMSVSLVADENPFAQGALK | IPI00796316, IPI00026314, IPI00647556, IPI00513782, IPI00646773, IPI00641047, IPI00377087 | Isoform 1 of Gelsolin precursor | GSN |
| YIETDPANR | IPI00796316, IPI00026314, IPI00647556, IPI00513782, IPI00646773, IPI00641047, IPI00377087 | Isoform 1 of Gelsolin precursor | GSN |
| ALSAGAGPSWR | IPI00016862, IPI00794573 | Isoform Mitochondrial of Glutathione reductase, mitochondrial | GSR |
| ALAEGVLLR | IPI00010706, IPI00915302 | Glutathione synthetase | GSS |
| GSAPPGPVPEGSIR | IPI00019755 | Glutathione transferase omega-1 | GSTO1 |
| MILELFSK | IPI00019755, IPI00513927, IPI00642936 | Glutathione transferase omega-1 | GSTO1 |
| VPSLVGSFIR | IPI00019755, IPI00513927, IPI00642936 | Glutathione transferase omega-1 | GSTO1 |
| GSAVVGQYQSYSEQVR | IPI00607861 | GDH/6PGL endoplasmic bifunctional protein precursor | H6PD |
| LCAEEDQGAQIYAR | IPI00607861 | GDH/6PGL endoplasmic bifunctional protein precursor | H6PD |
| LLDFEFSSGR | IPI00607861 | GDH/6PGL endoplasmic bifunctional protein precursor | H6PD |
| LQVFQALR | IPI00607861 | GDH/6PGL endoplasmic bifunctional protein precursor | H6PD |
| QLVFHIGHGDLGSPAVLVSR | IPI00607861 | GDH/6PGL endoplasmic bifunctional protein precursor | H6PD |
| DEIPHNDIALLK | IPI00746623 | Uncharacterized protein HABP2 | HABP2 |
| FTCACPDQFK | IPI00746623 | Uncharacterized protein HABP2 | HABP2 |
| GQCLITQSPPYYR | IPI00746623 | Uncharacterized protein HABP2 | HABP2 |
| IYGGFK | IPI00746623 | Uncharacterized protein HABP2 | HABP2 |
| LIANTLCNSR | IPI00746623 | Uncharacterized protein HABP2 | HABP2 |
| MFLSFPTTK | IPI00410714, IPI00853068 | Hemoglobin subunit alpha | HBA |
| VGAHAGEYGAEALER | IPI00410714 | Hemoglobin subunit alpha | HBA |
| EFTPPVQAAYQK | IPI00654755 | Hemoglobin subunit beta | HBB |
| FFESFGDLSTPDAVMGNPK | IPI00220706, IPI00796636, IPI00791558, IPI00749035, IPI00658153, IPI00939160, IPI00383795, IPI00940370, IPI00654755, IPI00939544, IPI00554676, IPI00657660, IPI00816644, IPI00473011, IPI00815947, IPI00884107, IPI00744503, IPI00853641, IPI00217471, IPI00829896, IPI00930351, IPI00657911, IPI00657703 | Hemoglobin subunit epsilon | HBD |
| GTFATLSELHCDK | IPI00220706, IPI00796636, IPI00791558, IPI00749035, IPI00658153, IPI00939160, IPI00383795, IPI00940370, IPI00654755, IPI00939544, IPI00554676, IPI00657660, IPI00816644, IPI00473011, IPI00815947, IPI00884107, IPI00744503, IPI00853641, IPI00217471, IPI00829896, IPI00930351, IPI00657911, IPI00657703 | Hemoglobin subunit epsilon | HBD |
| LHVDPENFR | IPI00220706, IPI00796636, IPI00791558, IPI00749035, IPI00658153, IPI00939160, IPI00383795, IPI00940370, IPI00654755, IPI00939544, IPI00554676, IPI00657660, IPI00816644, IPI00473011, IPI00815947, IPI00884107, IPI00744503, IPI00853641, IPI00217471, IPI00829896, IPI00930351, IPI00657911, IPI00657703 | Hemoglobin subunit epsilon | HBD |
| LLGNVLVCVLAHHFGK | IPI00220706, IPI00796636, IPI00791558, IPI00749035, IPI00658153, IPI00939160, IPI00383795, IPI00940370, IPI00654755, IPI00939544, IPI00554676, IPI00657660, IPI00816644, IPI00473011, IPI00815947, IPI00884107, IPI00744503, IPI00853641, IPI00217471, IPI00829896, IPI00930351, IPI00657911, IPI00657703 | Hemoglobin subunit epsilon | HBD |
| LLGNVLVCVLAR | IPI00220706, IPI00796636, IPI00791558, IPI00749035, IPI00658153, IPI00939160, IPI00383795, IPI00940370, IPI00654755, IPI00939544, IPI00554676, IPI00657660, IPI00816644, IPI00473011, IPI00815947, IPI00884107, IPI00744503, IPI00853641, IPI00217471, IPI00829896, IPI00930351, IPI00657911, IPI00657703 | Hemoglobin subunit epsilon | HBD |
| LLVVYPWTQR | IPI00220706, IPI00796636, IPI00791558, IPI00749035, IPI00658153, IPI00939160, IPI00383795, IPI00940370, IPI00654755, IPI00939544, IPI00554676, IPI00657660, IPI00816644, IPI00473011, IPI00815947, IPI00884107, IPI00744503, IPI00853641, IPI00217471, IPI00829896, IPI00930351, IPI00657911, IPI00657703 | Hemoglobin subunit epsilon | HBD |
| VHLTPEEK | IPI00220706, IPI00796636, IPI00791558, IPI00749035, IPI00658153, IPI00939160, IPI00383795, IPI00940370, IPI00654755, IPI00939544, IPI00554676, IPI00657660, IPI00816644, IPI00473011, IPI00815947, IPI00884107, IPI00744503, IPI00853641, IPI00217471, IPI00829896, IPI00930351, IPI00657911, IPI00657703 | Hemoglobin subunit epsilon | HBD |
| VLGAFSDGLAHLDNLK | IPI00220706, IPI00796636, IPI00791558, IPI00749035, IPI00658153, IPI00939160, IPI00383795, IPI00940370, IPI00654755, IPI00939544, IPI00554676, IPI00657660, IPI00816644, IPI00473011, IPI00815947, IPI00884107, IPI00744503, IPI00853641, IPI00217471, IPI00829896, IPI00930351, IPI00657911, IPI00657703 | Hemoglobin subunit epsilon | HBD |
| INESLQELR | IPI00452728, IPI00452727, IPI00915924, IPI00916699 | Isoform 1 of Transcription cofactor HES-6 | HES6 |
| IQPDTIIQVWR | IPI00941167, IPI00027851, IPI00909914 | Beta-hexosaminidase subunit alpha | HEXA |
| EISEVFPDQFIHLGGDEVEFK | IPI00012585 | Beta-hexosaminidase beta chain precursor | HEXB |
| CQIAGWGHLDENVSGYSSSLR | IPI00029193 | Hepatocyte growth factor activator precursor | HGFAC |
| CSSPEVYGADISPNMLCAGYFDCK | IPI00029193 | Hepatocyte growth factor activator precursor | HGFAC |
| DSALSWEYCR | IPI00029193 | Hepatocyte growth factor activator precursor | HGFAC |
| EALVPLVADHK | IPI00029193 | Hepatocyte growth factor activator precursor | HGFAC |
| LCNIEPDER | IPI00029193 | Hepatocyte growth factor activator precursor | HGFAC |
| LEACESLTR | IPI00029193 | Hepatocyte growth factor activator precursor | HGFAC |
| NGVAYLYGIISWGDGCGR | IPI00029193 | Hepatocyte growth factor activator precursor | HGFAC |
| NPDNDERPWCYVVK | IPI00029193 | Hepatocyte growth factor activator precursor | HGFAC |
| QGHVEQCECFGGR | IPI00029193 | Hepatocyte growth factor activator precursor | HGFAC |
| SDACQGDSGGPLACEK | IPI00029193 | Hepatocyte growth factor activator precursor | HGFAC |
| SQFVQPICLPEPGSTFPAGHK | IPI00029193 | Hepatocyte growth factor activator precursor | HGFAC |
| TTDVTQTFGIEK | IPI00029193 | Hepatocyte growth factor activator precursor | HGFAC |
| VANYVDWINDR | IPI00029193 | Hepatocyte growth factor activator precursor | HGFAC |
| VQLSPDLLATLPEPASPGR | IPI00029193 | Hepatocyte growth factor activator precursor | HGFAC |
| YEYLEGGDR | IPI00029193 | Hepatocyte growth factor activator precursor | HGFAC |
| YIPYTLYSVFNPSDHDLVLIR | IPI00029193 | Hepatocyte growth factor activator precursor | HGFAC |
| DGEDQTQDTELVETRPAGDGTFQK | IPI00026650, IPI00647634, IPI00760554, IPI00940270, IPI00939416, IPI00472222, IPI00936159, IPI00742968, IPI00893894, IPI00893004, IPI00937046, IPI00941803, IPI00930360, IPI00795906, IPI00004672, IPI00893694, IPI00894106, IPI00940896, IPI00914606, IPI00893131, IPI00914567, IPI00893023, IPI00939503, IPI00788914, IPI00472882, IPI00941924, IPI00816779, IPI00647457, IPI00472013, IPI00930530, IPI00876963, IPI00745699, IPI00472825, IPI00472162, IPI00892868, IPI00651697, IPI00644748, IPI00939166, IPI00935944, IPI00642409, IPI00936164, IPI00472112, IPI00939770, IPI00939887, IPI00941854, IPI00940006, IPI00789567, IPI00940303, IPI00790450, IPI00472736, IPI00940059, IPI00940116, IPI00418883, IPI00939717, IPI00797121, IPI00743716, IPI00472855, IPI00657976, IPI00472605, IPI00472903, IPI00893133, IPI00796489, IPI00745649, IPI00894325, IPI00936210, IPI00941126, IPI00471951, IPI00790240, IPI00654689, IPI00940735, IPI00472943, IPI00942730, IPI00829792, IPI00892737, IPI00744964, IPI00892834, IPI00743359, IPI00941415, IPI00939629, IPI00789627, IPI00892768, IPI00794678, IPI00942604, IPI00646225, IPI00936093, IPI00942762, IPI00743503, IPI00935329, IPI00026569, IPI00472448, IPI00892776, IPI00885217, IPI00472035, IPI00930330, IPI00893949, IPI00797359, IPI00472921, IPI00914583, IPI00942958, IPI00785070, IPI00889629, IPI00914650, IPI00749025, IPI00473006, IPI00472151 | Putative HLA class I histocompatibility antigen, alpha chain H | HLA1 |
| AVGDKLPECEAVCGKPK | IPI00478493, IPI00431645, IPI00641737, IPI00607707, IPI00941812, IPI00942787, IPI00477597 | Haptoglobin | HP |
| DIAPTLTLYVGK | IPI00478493, IPI00431645, IPI00641737, IPI00607707, IPI00941812, IPI00942787, IPI00477597 | Haptoglobin | HP |
| GSFPWQAK | IPI00478493, IPI00431645, IPI00641737, IPI00607707, IPI00941812, IPI00942787, IPI00477597 | Haptoglobin | HP |
| HYEGSTVPEK | IPI00478493, IPI00431645, IPI00641737, IPI00607707, IPI00941812, IPI00942787, IPI00477597 | Haptoglobin | HP |
| HYEGSTVPEKK | IPI00478493, IPI00431645, IPI00641737, IPI00607707, IPI00941812, IPI00942787, IPI00477597 | Haptoglobin | HP |
| ILGGHLDAK | IPI00478493, IPI00431645, IPI00641737, IPI00607707, IPI00941812, IPI00942787, IPI00477597 | Haptoglobin | HP |
| LPECEAVCGKPK | IPI00478493, IPI00431645, IPI00641737, IPI00607707, IPI00941812, IPI00942787, IPI00477597 | Haptoglobin | HP |
| SCAVAEYGVYVK | IPI00478493, IPI00431645, IPI00641737, IPI00607707, IPI00941812, IPI00942787, IPI00477597 | Haptoglobin | HP |
| SPVGVQPILNEHTFCAGMSK | IPI00478493, IPI00431645, IPI00641737, IPI00607707, IPI00941812, IPI00942787, IPI00477597 | Haptoglobin | HP |
| TEGDGVYTLNDK | IPI00478493, IPI00431645, IPI00641737, IPI00607707, IPI00941812, IPI00942787, IPI00477597 | Haptoglobin | HP |
| TEGDGVYTLNDKK | IPI00478493, IPI00431645, IPI00641737, IPI00607707, IPI00941812, IPI00942787, IPI00477597 | Haptoglobin | HP |
| TEGDGVYTLNNEK | IPI00478493, IPI00431645, IPI00641737, IPI00607707, IPI00941812, IPI00942787, IPI00477597 | Haptoglobin | HP |
| VGYVSGWGQSDNFK | IPI00478493, IPI00431645, IPI00641737, IPI00607707, IPI00941812, IPI00942787, IPI00477597 | Haptoglobin | HP |
| VMPICLPSK | IPI00478493, IPI00431645, IPI00641737, IPI00607707, IPI00941812, IPI00942787, IPI00477597 | Haptoglobin | HP |
| VMPICLPSKDYAEVGR | IPI00478493, IPI00431645, IPI00641737, IPI00607707, IPI00941812, IPI00942787, IPI00477597 | Haptoglobin | HP |
| VTSIQDWVQK | IPI00478493, IPI00431645, IPI00641737, IPI00607707, IPI00941812, IPI00942787, IPI00477597 | Haptoglobin | HP |
| YVMLPVADQDQCIR | IPI00478493, IPI00431645, IPI00641737, IPI00607707, IPI00941812, IPI00942787, IPI00477597 | Haptoglobin | HP |
| YVMLPVADQYDCITHYEGSTCPK | IPI00478493, IPI00431645, IPI00641737, IPI00607707, IPI00941812, IPI00942787, IPI00477597 | Haptoglobin | HP |
| NFPSPVDAAFR | IPI00022488, IPI00922564 | Hemopexin | HPX |
| DYFMPCPGR | IPI00022488 | Hemopexin precursor | HPX |
| EVGTPHGIILDSVDAAFICPGSSR | IPI00022488 | Hemopexin precursor | HPX |
| EWFWDLATGTMK | IPI00022488 | Hemopexin precursor | HPX |
| GECQAEGVLFFQGDR | IPI00022488 | Hemopexin precursor | HPX |
| GECQAEGVLFFQGDREWFWDLATGTMK | IPI00022488 | Hemopexin precursor | HPX |
| LHIMAGR | IPI00022488 | Hemopexin precursor | HPX |
| LLQDEFPGIPSPLDAAVECHR | IPI00022488 | Hemopexin precursor | HPX |
| LWWLDLK | IPI00022488 | Hemopexin precursor | HPX |
| RLWWLDLK | IPI00022488 | Hemopexin precursor | HPX |
| SGAQATWTELPWPHEK | IPI00022488 | Hemopexin precursor | HPX |
| SLGPNSCSANGPGLYLIHGPNLYCYSDVEK | IPI00022488 | Hemopexin precursor | HPX |
| VWVYPPEK | IPI00022488 | Hemopexin precursor | HPX |
| WKNFPSPVDAAFR | IPI00022488 | Hemopexin precursor | HPX |
| YYCFQGNQFLR | IPI00022488 | Hemopexin precursor | HPX |
| DGYLFQLLR | IPI00022371 | Histidine-rich glycoprotein precursor | HRG |
| DSPVLIDFFEDTER | IPI00022371 | Histidine-rich glycoprotein precursor | HRG |
| GGEGTGYFVDFSVR | IPI00022371 | Histidine-rich glycoprotein precursor | HRG |
| HPNVFGFCR | IPI00022371 | Histidine-rich glycoprotein precursor | HRG |
| YKEENDDFASFR | IPI00022371 | Histidine-rich glycoprotein precursor | HRG |
| AYALAFAER | IPI00019912 | Peroxisomal multifunctional enzyme type 2 | HSD17B4 |
| DNHLLGTFDLTGIPPAPR | IPI00003362 | HSPA5 protein | HSPA5 |
| FEELNADLFR | IPI00939442, IPI00902596, IPI00910870, IPI00893099, IPI00940553, IPI00003362, IPI00939595, IPI00940023, IPI00942547, IPI00939753, IPI00942823, IPI00037070, IPI00877807, IPI00830052, IPI00643152, IPI00003865, IPI00007702 | HSPA5 protein | HSPA5 |
| FEELNMDLFR | IPI00003362 | HSPA5 protein | HSPA5 |
| IEWLESHQDADIEDFK | IPI00003362 | HSPA5 protein | HSPA5 |
| IINEPTAAAIAYGLDK | IPI00939442, IPI00902596, IPI00910870, IPI00893099, IPI00940553, IPI00003362, IPI00939595, IPI00940023, IPI00942547, IPI00939753, IPI00942823, IPI00037070, IPI00877807, IPI00830052, IPI00643152, IPI00003865, IPI00007702 | HSPA5 protein | HSPA5 |
| ITPSYVAFTPEGER | IPI00003362 | HSPA5 protein | HSPA5 |
| NELESYAYSLK | IPI00003362 | HSPA5 protein | HSPA5 |
| NQLTSNPENTVFDAK | IPI00003362 | HSPA5 protein | HSPA5 |
| QTQTFTTYSDNQPGVLIQVYEGER | IPI00939442, IPI00902596, IPI00910870, IPI00893099, IPI00940553, IPI00003362, IPI00939595, IPI00940023, IPI00942547, IPI00939753, IPI00942823, IPI00037070, IPI00877807, IPI00830052, IPI00643152, IPI00003865, IPI00007702 | HSPA5 protein | HSPA5 |
| SDIDEIVLVGGSTR | IPI00003362 | HSPA5 protein | HSPA5 |
| SQIFSTASDNQPTVTIK | IPI00003362 | HSPA5 protein | HSPA5 |
| TWNDPSVQQDIK | IPI00003362 | HSPA5 protein | HSPA5 |
| VTHAVVTVPAYFNDAQR | IPI00003362 | HSPA5 protein | HSPA5 |
| AASGPGPEQEASFTVTVPPSEGSSYR | IPI00939313, IPI00747758, IPI00943326, IPI00024284, IPI00514485 | Basement membrane-specific heparan sulfate proteoglycan core protein | HSPG2 |
| AELLVTEAPSKPITVTVEEQR | IPI00939313, IPI00747758, IPI00943326, IPI00024284, IPI00514485 | Basement membrane-specific heparan sulfate proteoglycan core protein | HSPG2 |
| AMDFNGILTIR | IPI00939313, IPI00747758, IPI00943326, IPI00024284, IPI00514485 | Basement membrane-specific heparan sulfate proteoglycan core protein | HSPG2 |
| CAPGYYGNPSQGQPCQR | IPI00939313, IPI00747758, IPI00943326, IPI00024284, IPI00514485 | Basement membrane-specific heparan sulfate proteoglycan core protein | HSPG2 |
| CPPGYIGLSCQDCAPGYTR | IPI00939313, IPI00747758, IPI00943326, IPI00024284, IPI00514485 | Basement membrane-specific heparan sulfate proteoglycan core protein | HSPG2 |
| DFISLGLQDGHLVFR | IPI00939313, IPI00747758, IPI00943326, IPI00024284, IPI00514485 | Basement membrane-specific heparan sulfate proteoglycan core protein | HSPG2 |
| DSQVPGPIGCNCDPQGSVSSQCDAAGQCQCK | IPI00939313, IPI00747758, IPI00943326, IPI00024284, IPI00514485 | Basement membrane-specific heparan sulfate proteoglycan core protein | HSPG2 |
| EHLLMALADLDELLIR | IPI00939313, IPI00747758, IPI00943326, IPI00024284, IPI00514485 | Basement membrane-specific heparan sulfate proteoglycan core protein | HSPG2 |
| EHLLMALAGIDTLLIR | IPI00939313, IPI00747758, IPI00943326, IPI00024284, IPI00514485 | Basement membrane-specific heparan sulfate proteoglycan core protein | HSPG2 |
| LLSGPYFWSLPSR | IPI00939313, IPI00747758, IPI00943326, IPI00024284, IPI00514485 | Basement membrane-specific heparan sulfate proteoglycan core protein | HSPG2 |
| LPAVEPTDQAQYLCR | IPI00939313, IPI00747758, IPI00943326, IPI00024284, IPI00514485 | Basement membrane-specific heparan sulfate proteoglycan core protein | HSPG2 |
| LPQVSPADSGEYVCR | IPI00939313, IPI00747758, IPI00943326, IPI00024284, IPI00514485 | Basement membrane-specific heparan sulfate proteoglycan core protein | HSPG2 |
| SLPEVPETIELEVR | IPI00939313, IPI00747758, IPI00943326, IPI00024284, IPI00514485 | Basement membrane-specific heparan sulfate proteoglycan core protein | HSPG2 |
| SPGPNVAVNAK | IPI00939313, IPI00747758, IPI00943326, IPI00024284, IPI00514485 | Basement membrane-specific heparan sulfate proteoglycan core protein | HSPG2 |
| SQSVRPGADVTFICTAK | IPI00939313, IPI00747758, IPI00943326, IPI00024284, IPI00514485 | Basement membrane-specific heparan sulfate proteoglycan core protein | HSPG2 |
| AANSLEAFIFETQDK | IPI00000877, IPI00922838 | Hypoxia up-regulated protein 1 | HYOU1 |
| EVQYLLNK | IPI00000877, IPI00922838 | Hypoxia up-regulated protein 1 | HYOU1 |
| KLCQGLFFR | IPI00000877, IPI00922838 | Hypoxia up-regulated protein 1 | HYOU1 |
| LCQGLFFR | IPI00000877, IPI00922838 | Hypoxia up-regulated protein 1 | HYOU1 |
| LIPEMDQIFTEVEMTTLEK | IPI00000877, IPI00922838 | Hypoxia up-regulated protein 1 | HYOU1 |
| LSAASTWLEDEGVGATTVMLK | IPI00000877, IPI00922838 | Hypoxia up-regulated protein 1 | HYOU1 |
| LYQPEYQEVSTEEQREEISGK | IPI00000877, IPI00922838 | Hypoxia up-regulated protein 1 | HYOU1 |
| ASVSVTAEDEGTQR | IPI00008494, IPI00909039, IPI00910734, IPI00385789, IPI00642425 | Intercellular adhesion molecule 1 | ICAM1 |
| DGTFPLPIGESVTVTR | IPI00008494, IPI00909039, IPI00910734, IPI00385789, IPI00642425 | Intercellular adhesion molecule 1 | ICAM1 |
| LLGIETPLPK | IPI00008494, IPI00909039, IPI00910734, IPI00385789, IPI00642425 | Intercellular adhesion molecule 1 | ICAM1 |
| REPAVGEPAEVTTTVLVR | IPI00008494, IPI00909039, IPI00910734, IPI00385789, IPI00642425 | Intercellular adhesion molecule 1 | ICAM1 |
| TFLTVYWTPER | IPI00008494, IPI00909039, IPI00910734, IPI00385789, IPI00642425 | Intercellular adhesion molecule 1 | ICAM1 |
| VELAPLPSWQPVGK | IPI00008494, IPI00909039, IPI00910734, IPI00385789, IPI00642425 | Intercellular adhesion molecule 1 | ICAM1 |
| VTLNGVPAQPLGPR | IPI00008494, IPI00909039, IPI00910734, IPI00385789, IPI00642425 | Intercellular adhesion molecule 1 | ICAM1 |
| ILLDEQAQWK | IPI00790846, IPI00009477 | Intercellular adhesion molecule 2 | ICAM2 |
| VPTVEPLDSLTLFLFR | IPI00009477 | Intercellular adhesion molecule 2 precursor | ICAM2 |
| AMVGSDVELSCACPEGSR | IPI00219131, IPI00790218, IPI00414888, IPI00796189 | Isoform 1 of ICOS ligand precursor | ICOSLG |
| GLYDVVSVLR | IPI00219131, IPI00790218, IPI00414888, IPI00796189 | Isoform 1 of ICOS ligand precursor | ICOSLG |
| IAEFAFEYAR | IPI00792971, IPI00921820, IPI00030702, IPI00909577 | Isoform 1 of Isocitrate dehydrogenase [NAD] subunit alpha, mitochondrial | IDH3A |
| APQTGIVDECCFR | IPI00433029, IPI00852675, IPI00793994, IPI00853336, IPI00941100, IPI00001610, IPI00797681 | Insulin-like growth factor IA | IGF1 |
| GFYFNKPTGYGSSSR | IPI00433029, IPI00852675, IPI00793994, IPI00853336, IPI00941100, IPI00001610, IPI00797681 | Insulin-like growth factor IA | IGF1 |
| RAPQTGIVDECCFR | IPI00433029, IPI00852675, IPI00793994, IPI00853336, IPI00941100, IPI00001610, IPI00797681 | Insulin-like growth factor IA | IGF1 |
| RLEMYCAPLKPAK | IPI00433029, IPI00852675, IPI00793994, IPI00853336, IPI00941100, IPI00001610, IPI00797681 | Insulin-like growth factor IA | IGF1 |
| FFQYDTWK | IPI00215977, IPI00940065, IPI00001611 | Isoform 1 of Insulin-like growth factor II | IGF2 |
| GIVEECCFR | IPI00215977, IPI00940065, IPI00001611 | Isoform 1 of Insulin-like growth factor II | IGF2 |
| SCDLALLETYCATPAK | IPI00215977, IPI00940065, IPI00001611 | Isoform 1 of Insulin-like growth factor II | IGF2 |
| ATLITFLCDR | IPI00289819, IPI00903213 | Cation-independent mannose-6-phosphate receptor | IGF2R |
| ETSDCSYLFEWR | IPI00289819, IPI00903213 | Cation-independent mannose-6-phosphate receptor | IGF2R |
| LDFCDGHSPAVTITFVCPSER | IPI00289819, IPI00903213 | Cation-independent mannose-6-phosphate receptor | IGF2R |
| AFWLDVSHNR | IPI00925635, IPI00020996 | Insulin-like growth factor-binding protein complex acid labile chain | IGFALS |
| AGAFLGLTNVAVMNLSGNCLR | IPI00925635, IPI00020996 | Insulin-like growth factor-binding protein complex acid labile chain | IGFALS |
| ANVFVQLPR | IPI00925635, IPI00020996 | Insulin-like growth factor-binding protein complex acid labile chain | IGFALS |
| DFALQNPSAVPR | IPI00925635, IPI00020996 | Insulin-like growth factor-binding protein complex acid labile chain | IGFALS |
| DLHFLEELQLGHNR | IPI00925635, IPI00020996 | Insulin-like growth factor-binding protein complex acid labile chain | IGFALS |
| DLSEAHFAPC | IPI00925635, IPI00020996 | Insulin-like growth factor-binding protein complex acid labile chain | IGFALS |
| ELVLAGNR | IPI00925635, IPI00020996 | Insulin-like growth factor-binding protein complex acid labile chain | IGFALS |
| LAELPADALGPLQR | IPI00925635, IPI00020996 | Insulin-like growth factor-binding protein complex acid labile chain | IGFALS |
| LAYLQPALFSGLAELR | IPI00925635, IPI00020996 | Insulin-like growth factor-binding protein complex acid labile chain | IGFALS |
| LEALPNSLLAPLGR | IPI00925635, IPI00020996 | Insulin-like growth factor-binding protein complex acid labile chain | IGFALS |
| LEYLLLSR | IPI00925635, IPI00020996 | Insulin-like growth factor-binding protein complex acid labile chain | IGFALS |
| LFQGLGKLEYLLLSR | IPI00925635, IPI00020996 | Insulin-like growth factor-binding protein complex acid labile chain | IGFALS |
| LWLEGNPWDCGCPLK | IPI00925635, IPI00020996 | Insulin-like growth factor-binding protein complex acid labile chain | IGFALS |
| NLIAAVAPGAFLGLK | IPI00925635, IPI00020996 | Insulin-like growth factor-binding protein complex acid labile chain | IGFALS |
| NLPEQVFR | IPI00925635, IPI00020996 | Insulin-like growth factor-binding protein complex acid labile chain | IGFALS |
| SFEGLGQLEVLTLDHNQLQEVK | IPI00925635, IPI00020996 | Insulin-like growth factor-binding protein complex acid labile chain | IGFALS |
| SLALGTFAHTPALASLGLSNNR | IPI00925635, IPI00020996 | Insulin-like growth factor-binding protein complex acid labile chain | IGFALS |
| TFTPQPPGLER | IPI00925635, IPI00020996 | Insulin-like growth factor-binding protein complex acid labile chain | IGFALS |
| VAGLLEDTFPGLLGLR | IPI00925635, IPI00020996 | Insulin-like growth factor-binding protein complex acid labile chain | IGFALS |
| WLDLSHNR | IPI00925635, IPI00020996 | Insulin-like growth factor-binding protein complex acid labile chain | IGFALS |
| ALPGEQQPLHALTR | IPI00926019, IPI00031086 | Insulin-like growth factor-binding protein 1 | IGFBP1 |
| GDPECHLFYNEQQEAR | IPI00927165, IPI00926948, IPI00556442, IPI00297284 | Insulin-like growth factor-binding protein 2 | IGFBP2 |
| GECWCVNPNTGK | IPI00927165, IPI00926948, IPI00556442, IPI00297284 | Insulin-like growth factor-binding protein 2 | IGFBP2 |
| LAACGPPPVAPPAAVAAVAGGAR | IPI00927165, IPI00926948, IPI00556442, IPI00297284 | Insulin-like growth factor-binding protein 2 | IGFBP2 |
| LEGEACGVYTPR | IPI00927165, IPI00926948, IPI00556442, IPI00297284 | Insulin-like growth factor-binding protein 2 | IGFBP2 |
| LIQGAPTIR | IPI00927165, IPI00926948, IPI00556442, IPI00297284 | Insulin-like growth factor-binding protein 2 | IGFBP2 |
| TPCQQELDQVLER | IPI00927165, IPI00926948, IPI00556442, IPI00297284 | Insulin-like growth factor-binding protein 2 | IGFBP2 |
| ALAQCAPPPAVCAELVR | IPI00924814, IPI00903293, IPI00925358, IPI00556155, IPI00926261, IPI00855835, IPI00902471, IPI00444386, IPI00018305 | Insulin-like growth factor-binding protein 3 | IGFBP3 |
| CQPSPDEARPLQALLDGR | IPI00924814, IPI00903293, IPI00925358, IPI00556155, IPI00926261, IPI00855835, IPI00902471, IPI00444386, IPI00018305 | Insulin-like growth factor-binding protein 3 | IGFBP3 |
| EMEDTLNHLK | IPI00924814, IPI00903293, IPI00925358, IPI00556155, IPI00926261, IPI00855835, IPI00902471, IPI00444386, IPI00018305 | Insulin-like growth factor-binding protein 3 | IGFBP3 |
| EPGCGCCLTCALSEGQPCGIYTER | IPI00924814, IPI00903293, IPI00925358, IPI00556155, IPI00926261, IPI00855835, IPI00902471, IPI00444386, IPI00018305 | Insulin-like growth factor-binding protein 3 | IGFBP3 |
| FLNVLSPR | IPI00924814, IPI00903293, IPI00925358, IPI00556155, IPI00926261, IPI00855835, IPI00902471, IPI00444386, IPI00018305 | Insulin-like growth factor-binding protein 3 | IGFBP3 |
| GFCWCVDK | IPI00924814, IPI00903293, IPI00925358, IPI00556155, IPI00926261, IPI00855835, IPI00902471, IPI00444386, IPI00018305 | Insulin-like growth factor-binding protein 3 | IGFBP3 |
| GVHIPNCDK | IPI00924814, IPI00903293, IPI00925358, IPI00556155, IPI00926261, IPI00855835, IPI00902471, IPI00444386, IPI00018305 | Insulin-like growth factor-binding protein 3 | IGFBP3 |
| GVHIPNCDKK | IPI00924814, IPI00903293, IPI00925358, IPI00556155, IPI00926261, IPI00855835, IPI00902471, IPI00444386, IPI00018305 | Insulin-like growth factor-binding protein 3 | IGFBP3 |
| VDYESQSTDTQNFSSESK | IPI00924814, IPI00903293, IPI00925358, IPI00556155, IPI00926261, IPI00855835, IPI00902471, IPI00444386, IPI00018305 | Insulin-like growth factor-binding protein 3 | IGFBP3 |
| YGQPLPGYTTK | IPI00924814, IPI00903293, IPI00925358, IPI00556155, IPI00926261, IPI00855835, IPI00902471, IPI00444386, IPI00018305 | Insulin-like growth factor-binding protein 3 | IGFBP3 |
| YKVDYESQSTDTQNFSSESK | IPI00924814, IPI00903293, IPI00925358, IPI00556155, IPI00926261, IPI00855835, IPI00902471, IPI00444386, IPI00018305 | Insulin-like growth factor-binding protein 3 | IGFBP3 |
| CRPPVGCEELVR | IPI00305380 | Insulin-like growth factor-binding protein 4 precursor | IGFBP4 |
| EPGCGCCATCALGLGMPCGVYTPR | IPI00305380 | Insulin-like growth factor-binding protein 4 precursor | IGFBP4 |
| GELDCHQLADSFRE | IPI00305380 | Insulin-like growth factor-binding protein 4 precursor | IGFBP4 |
| ALSMCPPSPLGCELVK | IPI00924537, IPI00029236 | Insulin-like growth factor-binding protein 5 | IGFBP5 |
| EPGCGCCMTCALAEGQSCGVYTER | IPI00924537, IPI00029236 | Insulin-like growth factor-binding protein 5 | IGFBP5 |
| IISAPEMR | IPI00924537, IPI00029236 | Insulin-like growth factor-binding protein 5 | IGFBP5 |
| GICWCVDK | IPI00029236 | Insulin-like growth factor-binding protein 5 precursor | IGFBP5 |
| HMEASLQELK | IPI00029236 | Insulin-like growth factor-binding protein 5 precursor | IGFBP5 |
| APAVAEENPK | IPI00029235 | Insulin-like growth factor-binding protein 6 precursor | IGFBP6 |
| CPGCGQGVQAGCPGGCVEEEDGGSPAEGCAEAEGCLR | IPI00029235 | Insulin-like growth factor-binding protein 6 precursor | IGFBP6 |
| EGQECGVYTPNCAPGLQCHPPKDDEAPLR | IPI00029235 | Insulin-like growth factor-binding protein 6 precursor | IGFBP6 |
| GAQTLYVPNCDHR | IPI00029235 | Insulin-like growth factor-binding protein 6 precursor | IGFBP6 |
| HLDSVLQQLQTEVYR | IPI00029235 | Insulin-like growth factor-binding protein 6 precursor | IGFBP6 |
| NPGTSTTPSQPNSAGVQDTEMGPCR | IPI00029235 | Insulin-like growth factor-binding protein 6 precursor | IGFBP6 |
| AGAAAGGPGVSGVCVCK | IPI00016915 | Insulin-like growth factor-binding protein 7 precursor | IGFBP7 |
| ITVVDALHEIPVK | IPI00016915 | Insulin-like growth factor-binding protein 7 precursor | IGFBP7 |
| YPVCGSDGTTYPSGCQLR | IPI00016915 | Insulin-like growth factor-binding protein 7 precursor | IGFBP7 |
| VDDTAVYYCAR | IPI00386879 | CDNA FLJ14473 fis, clone MAMMA1001080, highly similar to Homo sapiens SNC73 protein (SNC73) mRNA | IGHA1 |
| APDVFPIISGCR | IPI00829636, IPI00163446, IPI00893578 | Isoform 2 of Ig delta chain C region | IGHD |
| ATFTCFVVGSDLK | IPI00829636, IPI00163446, IPI00893578 | Isoform 2 of Ig delta chain C region | IGHD |
| DSYYMTSSQLSTPLQQWR | IPI00829636, IPI00163446, IPI00893578 | Isoform 2 of Ig delta chain C region | IGHD |
| EPAAQAPVK | IPI00829636, IPI00163446, IPI00893578 | Isoform 2 of Ig delta chain C region | IGHD |
| EVNTSGFAPARPPPQPGSTTFWAWSVLR | IPI00829636, IPI00163446, IPI00893578 | Isoform 2 of Ig delta chain C region | IGHD |
| TPECPSHTQPLGVYLLTPAVQDLWLR | IPI00829636, IPI00163446, IPI00893578 | Isoform 2 of Ig delta chain C region | IGHD |
| VPAPPSPQPATYTCVVSHEDSR | IPI00829636, IPI00163446, IPI00893578 | Isoform 2 of Ig delta chain C region | IGHD |
| VPTGGVEEGLLER | IPI00829636, IPI00163446, IPI00893578 | Isoform 2 of Ig delta chain C region | IGHD |
| DGFFGNPR | IPI00941961, IPI00479708, IPI00477090, IPI00385264, IPI00940918, IPI00892870, IPI00941355, IPI00939620, IPI00940716, IPI00896380, IPI00940750 | Ig mu heavy chain disease protein | IGHM |
| DVMQGTDEHVVCK | IPI00941961, IPI00479708, IPI00477090, IPI00385264, IPI00940918, IPI00892870, IPI00941355, IPI00939620, IPI00940716, IPI00896380, IPI00940750 | Ig mu heavy chain disease protein | IGHM |
| ESATITCLVTGFSPADVFVQWMQR | IPI00941961, IPI00479708, IPI00477090, IPI00385264, IPI00940918, IPI00892870, IPI00941355, IPI00939620, IPI00940716, IPI00896380, IPI00940750 | Ig mu heavy chain disease protein | IGHM |
| ESDWLGQSMFTCR | IPI00941961, IPI00479708, IPI00477090, IPI00385264, IPI00940918, IPI00892870, IPI00941355, IPI00939620, IPI00940716, IPI00896380, IPI00940750 | Ig mu heavy chain disease protein | IGHM |
| ESDWLSQSMFTCR | IPI00941961, IPI00479708, IPI00477090, IPI00385264, IPI00940918, IPI00892870, IPI00941355, IPI00939620, IPI00940716, IPI00896380, IPI00940750 | Ig mu heavy chain disease protein | IGHM |
| ESGPTTYK | IPI00941961, IPI00479708, IPI00477090, IPI00385264, IPI00940918, IPI00892870, IPI00941355, IPI00939620, IPI00940716, IPI00896380, IPI00940750 | Ig mu heavy chain disease protein | IGHM |
| FTCTVTHTDLPSPLK | IPI00941961, IPI00479708, IPI00477090, IPI00385264, IPI00940918, IPI00892870, IPI00941355, IPI00939620, IPI00940716, IPI00896380, IPI00940750 | Ig mu heavy chain disease protein | IGHM |
| GQPLSPEK | IPI00941961, IPI00479708, IPI00477090, IPI00385264, IPI00940918, IPI00892870, IPI00941355, IPI00939620, IPI00940716, IPI00896380, IPI00940750 | Ig mu heavy chain disease protein | IGHM |
| GQPLSPEKYVTSAPMPEPQAPGR | IPI00941961, IPI00479708, IPI00477090, IPI00385264, IPI00940918, IPI00892870, IPI00941355, IPI00939620, IPI00940716, IPI00896380, IPI00940750 | Ig mu heavy chain disease protein | IGHM |
| GVALHRPDVYLLPPAR | IPI00941961, IPI00479708, IPI00477090, IPI00385264, IPI00940918, IPI00892870, IPI00941355, IPI00939620, IPI00940716, IPI00896380, IPI00940750 | Ig mu heavy chain disease protein | IGHM |
| LICQATGFSPR | IPI00941961, IPI00479708, IPI00477090, IPI00385264, IPI00940918, IPI00892870, IPI00941355, IPI00939620, IPI00940716, IPI00896380, IPI00940750 | Ig mu heavy chain disease protein | IGHM |
| LTCLVTDLTTYDSVTISWTR | IPI00941961, IPI00479708, IPI00477090, IPI00385264, IPI00940918, IPI00892870, IPI00941355, IPI00939620, IPI00940716, IPI00896380, IPI00940750 | Ig mu heavy chain disease protein | IGHM |
| NVPLPVIAELPPK | IPI00941961, IPI00479708, IPI00477090, IPI00385264, IPI00940918, IPI00892870, IPI00941355, IPI00939620, IPI00940716, IPI00896380, IPI00940750 | Ig mu heavy chain disease protein | IGHM |
| NVPLPVIAELPPKVSVFVPPR | IPI00941961, IPI00479708, IPI00477090, IPI00385264, IPI00940918, IPI00892870, IPI00941355, IPI00939620, IPI00940716, IPI00896380, IPI00940750 | Ig mu heavy chain disease protein | IGHM |
| QIQVSWLR | IPI00941961, IPI00479708, IPI00477090, IPI00385264, IPI00940918, IPI00892870, IPI00941355, IPI00939620, IPI00940716, IPI00896380, IPI00940750 | Ig mu heavy chain disease protein | IGHM |
| QVGSGVTTDQVQAEAK | IPI00941961, IPI00479708, IPI00477090, IPI00385264, IPI00940918, IPI00892870, IPI00941355, IPI00939620, IPI00940716, IPI00896380, IPI00940750 | Ig mu heavy chain disease protein | IGHM |
| STGKPTLYNVSLVMSDTAGTCY | IPI00941961, IPI00479708, IPI00477090, IPI00385264, IPI00940918, IPI00892870, IPI00941355, IPI00939620, IPI00940716, IPI00896380, IPI00940750 | Ig mu heavy chain disease protein | IGHM |
| VFAIPPSFASIFLTK | IPI00941961, IPI00479708, IPI00477090, IPI00385264, IPI00940918, IPI00892870, IPI00941355, IPI00939620, IPI00940716, IPI00896380, IPI00940750 | Ig mu heavy chain disease protein | IGHM |
| VSVFVPPR | IPI00941961, IPI00479708, IPI00477090, IPI00385264, IPI00940918, IPI00892870, IPI00941355, IPI00939620, IPI00940716, IPI00896380, IPI00940750 | Ig mu heavy chain disease protein | IGHM |
| VTSTLTIK | IPI00941961, IPI00479708, IPI00477090, IPI00385264, IPI00940918, IPI00892870, IPI00941355, IPI00939620, IPI00940716, IPI00896380, IPI00940750 | Ig mu heavy chain disease protein | IGHM |
| YAATSQVLLPSK | IPI00941961, IPI00479708, IPI00477090, IPI00385264, IPI00940918, IPI00892870, IPI00941355, IPI00939620, IPI00940716, IPI00896380, IPI00940750 | Ig mu heavy chain disease protein | IGHM |
| YAATSQVLLPSKDVMQGTDEHVVCK | IPI00941961, IPI00479708, IPI00477090, IPI00385264, IPI00940918, IPI00892870, IPI00941355, IPI00939620, IPI00940716, IPI00896380, IPI00940750 | Ig mu heavy chain disease protein | IGHM |
| YFAHSILTVSEEEWNTGETYTCVVAHEALPNR | IPI00941961, IPI00479708, IPI00477090, IPI00385264, IPI00940918, IPI00892870, IPI00941355, IPI00939620, IPI00940716, IPI00896380, IPI00940750 | Ig mu heavy chain disease protein | IGHM |
| YVTSAPMPEPQAPGR | IPI00941961, IPI00479708, IPI00477090, IPI00385264, IPI00940918, IPI00892870, IPI00941355, IPI00939620, IPI00940716, IPI00896380, IPI00940750 | Ig mu heavy chain disease protein | IGHM |
| CYTAVVPLVYGGETK | IPI00178926 | immunoglobulin J chain | IGJ |
| IIVPLNNR | IPI00178926 | immunoglobulin J chain | IGJ |
| SSEDPNEDIVER | IPI00178926 | immunoglobulin J chain | IGJ |
| DIQMTQSPSTLSASVGDR | IPI00829956, IPI00387024, IPI00829912, IPI00387026 | Ig kappa chain V-I region CAR | IGKV1-5 |
| ASQSVSSYLAWYQQKPGQAPR | IPI00784430 | Similar to Ig kappa chain V-III region VG precursor | IGKV3D-11 |
| LLIYDASNR | IPI00829653, IPI00784430, IPI00816799 | Similar to Ig kappa chain V-III region VG precursor | IGKV3D-11 |
| DIVMTQSPDSLAVSLGER | IPI00942259, IPI00385143, IPI00026197, IPI00387120, IPI00386132, IPI00943265, IPI00386133 | Ig kappa chain V-IV region | IGKV4-1 |
| LLIYWASTR | IPI00942259, IPI00385143, IPI00026197, IPI00387120, IPI00386132, IPI00943265, IPI00386133 | Ig kappa chain V-IV region | IGKV4-1 |
| CPQVPYWLWASVSPR | IPI00021382, IPI00924851, IPI00924548 | Interleukin-1 receptor type II | IL1R2 |
| LEGEPVALR | IPI00021382, IPI00924851, IPI00924548 | Interleukin-1 receptor type II | IL1R2 |
| CPLFEHFLK | IPI00107619, IPI00925621, IPI00337687, IPI00792619, IPI00927047, IPI00031789, IPI00926779, IPI00926554, IPI00925851 | Isoform 3 of Interleukin-1 receptor accessory protein | IL1RAP |
| DLEEPINFR | IPI00107619, IPI00925621, IPI00337687, IPI00792619, IPI00927047, IPI00031789, IPI00926779, IPI00926554, IPI00925851 | Isoform 3 of Interleukin-1 receptor accessory protein | IL1RAP |
| EPGEELLIPCTVYFSFLMDSR | IPI00107619, IPI00925621, IPI00337687, IPI00792619, IPI00927047, IPI00031789, IPI00926779, IPI00926554, IPI00925851 | Isoform 3 of Interleukin-1 receptor accessory protein | IL1RAP |
| NEVWWTIDGK | IPI00107619, IPI00925621, IPI00337687, IPI00792619, IPI00927047, IPI00031789, IPI00926779, IPI00926554, IPI00925851 | Isoform 3 of Interleukin-1 receptor accessory protein | IL1RAP |
| QDRDLEEPINFR | IPI00107619, IPI00925621, IPI00337687, IPI00792619, IPI00927047, IPI00031789, IPI00926779, IPI00926554, IPI00925851 | Isoform 3 of Interleukin-1 receptor accessory protein | IL1RAP |
| QIQVFEDEPAR | IPI00107619, IPI00925621, IPI00337687, IPI00792619, IPI00927047, IPI00031789, IPI00926779, IPI00926554, IPI00925851 | Isoform 3 of Interleukin-1 receptor accessory protein | IL1RAP |
| VAFPLEVVQK | IPI00107619, IPI00925621, IPI00337687, IPI00792619, IPI00927047, IPI00031789, IPI00926779, IPI00926554, IPI00925851 | Isoform 3 of Interleukin-1 receptor accessory protein | IL1RAP |
| ILDYEVTLTR | IPI00939624, IPI00554522, IPI00297124, IPI00554518 | Isoform 1 of Interleukin-6 receptor subunit beta | IL6ST |
| SSFTVQDLKPFTEYVFR | IPI00939624, IPI00554522, IPI00297124, IPI00554518 | Isoform 1 of Interleukin-6 receptor subunit beta | IL6ST |
| SDAAVLTIPACDFQATHPVMDLK | IPI00297124 | Isoform 1 of Interleukin-6 receptor subunit beta precursor | IL6ST |
| AGGQCPACGGPTLELESQR | IPI00023314, IPI00795132 | Inhibin beta C chain precursor | INHBC |
| QEFFVDFR | IPI00023314 | Inhibin beta C chain precursor | INHBC |
| ALPGTPVASSQPR | IPI00941086, IPI00023648 | Immunoglobulin superfamily containing leucine-rich repeat protein | ISLR |
| EVPLLQSLWLAHNEIR | IPI00023648 | Immunoglobulin superfamily containing leucine-rich repeat protein precursor | ISLR |
| LPGLPEGAFR | IPI00023648 | Immunoglobulin superfamily containing leucine-rich repeat protein precursor | ISLR |
| YGFQIADCAYR | IPI00023648 | Immunoglobulin superfamily containing leucine-rich repeat protein precursor | ISLR |
| WQTGTNPLYR | IPI00926088, IPI00000151, IPI00926712, IPI00917837 | Integrin beta-6 | ITGB6 |
| QNTGMWESNANVK | IPI00217987, IPI00645887 | Integrin alpha-M precursor | ITGM |
| ADVQAHGEGQEFSITCLVDEEEMK | IPI00292530, IPI00383338, IPI00877852 | Inter-alpha-trypsin inhibitor heavy chain H1 | ITIH1 |
| GMADQDGLKPTIDKPSEDSPPLEMLGPR | IPI00292530, IPI00383338, IPI00877852 | Inter-alpha-trypsin inhibitor heavy chain H1 | ITIH1 |
| LWAYLTIQELLAK | IPI00292530, IPI00383338, IPI00877852 | Inter-alpha-trypsin inhibitor heavy chain H1 | ITIH1 |
| QYYEGSEIVVAGR | IPI00292530, IPI00383338, IPI00877852 | Inter-alpha-trypsin inhibitor heavy chain H1 | ITIH1 |
| AAISGENAGLVR | IPI00292530 | Inter-alpha-trypsin inhibitor heavy chain H1 precursor | ITIH1 |
| ELAAQTIK | IPI00292530 | Inter-alpha-trypsin inhibitor heavy chain H1 precursor | ITIH1 |
| EVAFDLEIPK | IPI00292530 | Inter-alpha-trypsin inhibitor heavy chain H1 precursor | ITIH1 |
| FAHYVVTSQVVNTANEAR | IPI00292530 | Inter-alpha-trypsin inhibitor heavy chain H1 precursor | ITIH1 |
| GFSLDEATNLNGGLLR | IPI00292530 | Inter-alpha-trypsin inhibitor heavy chain H1 precursor | ITIH1 |
| GSLVQASEANLQAAQDFVR | IPI00292530 | Inter-alpha-trypsin inhibitor heavy chain H1 precursor | ITIH1 |
| ILGDMQPGDYFDLVLFGTR | IPI00292530 | Inter-alpha-trypsin inhibitor heavy chain H1 precursor | ITIH1 |
| NVVFVIDISGSMR | IPI00292530 | Inter-alpha-trypsin inhibitor heavy chain H1 precursor | ITIH1 |
| QAVDTAVDGVFIR | IPI00292530 | Inter-alpha-trypsin inhibitor heavy chain H1 precursor | ITIH1 |
| QLVHHFEIDVDIFEPQGISK | IPI00292530 | Inter-alpha-trypsin inhibitor heavy chain H1 precursor | ITIH1 |
| AEDHFSVIDFNQNIR | IPI00305461, IPI00910636, IPI00645038, IPI00514159 | Inter-alpha-trypsin inhibitor heavy chain H2 | ITIH2 |
| AGELEVFNGYFVHFFAPDNLDPIPK | IPI00305461, IPI00910636, IPI00645038, IPI00514159 | Inter-alpha-trypsin inhibitor heavy chain H2 | ITIH2 |
| ETAVDGELVVLYDVK | IPI00305461, IPI00910636, IPI00645038, IPI00514159 | Inter-alpha-trypsin inhibitor heavy chain H2 | ITIH2 |
| FLHVPDTFEGHFDGVPVISK | IPI00305461, IPI00910636, IPI00645038, IPI00514159 | Inter-alpha-trypsin inhibitor heavy chain H2 | ITIH2 |
| FYNQVSTPLLR | IPI00305461, IPI00910636, IPI00645038, IPI00514159 | Inter-alpha-trypsin inhibitor heavy chain H2 | ITIH2 |
| HLEVDVWVIEPQGLR | IPI00305461, IPI00910636, IPI00645038, IPI00514159 | Inter-alpha-trypsin inhibitor heavy chain H2 | ITIH2 |
| IQPSGGTNINEALLR | IPI00305461, IPI00910636, IPI00645038, IPI00514159 | Inter-alpha-trypsin inhibitor heavy chain H2 | ITIH2 |
| KLWAYLTINQLLAER | IPI00305461, IPI00910636, IPI00645038, IPI00514159 | Inter-alpha-trypsin inhibitor heavy chain H2 | ITIH2 |
| LWAYLTINQLLAER | IPI00305461, IPI00910636, IPI00645038, IPI00514159 | Inter-alpha-trypsin inhibitor heavy chain H2 | ITIH2 |
| MLADAPPQDPSCCSGALYYGSK | IPI00305461, IPI00910636, IPI00645038, IPI00514159 | Inter-alpha-trypsin inhibitor heavy chain H2 | ITIH2 |
| SILQMSLDHHIVTPLTSLVIENEAGDER | IPI00305461, IPI00910636, IPI00645038, IPI00514159 | Inter-alpha-trypsin inhibitor heavy chain H2 | ITIH2 |
| SLAPTAAAK | IPI00305461, IPI00910636, IPI00645038, IPI00514159 | Inter-alpha-trypsin inhibitor heavy chain H2 | ITIH2 |
| SSALDMENFR | IPI00305461, IPI00910636, IPI00645038, IPI00514159 | Inter-alpha-trypsin inhibitor heavy chain H2 | ITIH2 |
| VVNNSPQPQNVVFDVQIPK | IPI00305461, IPI00910636, IPI00645038, IPI00514159 | Inter-alpha-trypsin inhibitor heavy chain H2 | ITIH2 |
| DFLGFYVVDSHR | IPI00028413, IPI00876950, IPI00909807, IPI00873416 | Isoform 1 of Inter-alpha-trypsin inhibitor heavy chain H3 | ITIH3 |
| DYIFGNYIER | IPI00028413, IPI00876950, IPI00909807, IPI00873416 | Isoform 1 of Inter-alpha-trypsin inhibitor heavy chain H3 | ITIH3 |
| EHLVQATPENLQEAR | IPI00028413, IPI00876950, IPI00909807, IPI00873416 | Isoform 1 of Inter-alpha-trypsin inhibitor heavy chain H3 | ITIH3 |
| ESPGNVQIVNGYFVHFFAPQGLPVVPK | IPI00028413, IPI00876950, IPI00909807, IPI00873416 | Isoform 1 of Inter-alpha-trypsin inhibitor heavy chain H3 | ITIH3 |
| FAHNVVTMR | IPI00028413, IPI00876950, IPI00909807, IPI00873416 | Isoform 1 of Inter-alpha-trypsin inhibitor heavy chain H3 | ITIH3 |
| FPLYNLGFGNNLNYNFLENMALENHGFAR | IPI00028413, IPI00876950, IPI00909807, IPI00873416 | Isoform 1 of Inter-alpha-trypsin inhibitor heavy chain H3 | ITIH3 |
| GHGATNDLTFTEEVDMK | IPI00028413, IPI00876950, IPI00909807, IPI00873416 | Isoform 1 of Inter-alpha-trypsin inhibitor heavy chain H3 | ITIH3 |
| GMTNINDGLLR | IPI00028413, IPI00876950, IPI00909807, IPI00873416 | Isoform 1 of Inter-alpha-trypsin inhibitor heavy chain H3 | ITIH3 |
| LIQDAVTGLTVNGQITGDKR | IPI00028413, IPI00876950, IPI00909807, IPI00873416 | Isoform 1 of Inter-alpha-trypsin inhibitor heavy chain H3 | ITIH3 |
| LVDEDMNSFK | IPI00028413, IPI00876950, IPI00909807, IPI00873416 | Isoform 1 of Inter-alpha-trypsin inhibitor heavy chain H3 | ITIH3 |
| LWAYLTIEQLLEK | IPI00028413, IPI00876950, IPI00909807, IPI00873416 | Isoform 1 of Inter-alpha-trypsin inhibitor heavy chain H3 | ITIH3 |
| NVAFVIDISGSMAGR | IPI00028413, IPI00876950, IPI00909807, IPI00873416 | Isoform 1 of Inter-alpha-trypsin inhibitor heavy chain H3 | ITIH3 |
| SCPTCTDSLLNGDFTITYDVNR | IPI00028413, IPI00876950, IPI00909807, IPI00873416 | Isoform 1 of Inter-alpha-trypsin inhibitor heavy chain H3 | ITIH3 |
| SLPEGVANGIEVYSTK | IPI00028413, IPI00876950, IPI00909807, IPI00873416 | Isoform 1 of Inter-alpha-trypsin inhibitor heavy chain H3 | ITIH3 |
| STSIVIMLTDGDANVGESRPEK | IPI00028413, IPI00876950, IPI00909807, IPI00873416 | Isoform 1 of Inter-alpha-trypsin inhibitor heavy chain H3 | ITIH3 |
| VSDIRPGSDPTKPDATLVVK | IPI00028413, IPI00876950, IPI00909807, IPI00873416 | Isoform 1 of Inter-alpha-trypsin inhibitor heavy chain H3 | ITIH3 |
| YHFVTPLTSMVVTKPEDNEDER | IPI00028413, IPI00876950, IPI00909807, IPI00873416 | Isoform 1 of Inter-alpha-trypsin inhibitor heavy chain H3 | ITIH3 |
| AEAQAQYSAAVAK | IPI00896413, IPI00922043, IPI00896419, IPI00556036, IPI00791097, IPI00798006, IPI00218192 | Isoform 2 of Inter-alpha-trypsin inhibitor heavy chain H4 | ITIH4 |
| AGFSWIEVTFK | IPI00896413, IPI00922043, IPI00896419, IPI00556036, IPI00791097, IPI00798006, IPI00218192 | Isoform 2 of Inter-alpha-trypsin inhibitor heavy chain H4 | ITIH4 |
| AISGGSIQIENGYFVHYFAPEGLTTMPK | IPI00896413, IPI00922043, IPI00896419, IPI00556036, IPI00791097, IPI00798006, IPI00218192 | Isoform 2 of Inter-alpha-trypsin inhibitor heavy chain H4 | ITIH4 |
| ANTVQEATFQMELPK | IPI00896413, IPI00922043, IPI00896419, IPI00556036, IPI00791097, IPI00798006, IPI00218192 | Isoform 2 of Inter-alpha-trypsin inhibitor heavy chain H4 | ITIH4 |
| DQFNLIVFSTEATQWRPSLVPASAENVNK | IPI00896413, IPI00922043, IPI00896419, IPI00556036, IPI00791097, IPI00798006, IPI00218192 | Isoform 2 of Inter-alpha-trypsin inhibitor heavy chain H4 | ITIH4 |
| EALIKILDDLSPR | IPI00896413, IPI00922043, IPI00896419, IPI00556036, IPI00791097, IPI00798006, IPI00218192 | Isoform 2 of Inter-alpha-trypsin inhibitor heavy chain H4 | ITIH4 |
| EKAEAQAQYSAAVAK | IPI00896413, IPI00922043, IPI00896419, IPI00556036, IPI00791097, IPI00798006, IPI00218192 | Isoform 2 of Inter-alpha-trypsin inhibitor heavy chain H4 | ITIH4 |
| ETLFSVMPGLK | IPI00896413, IPI00922043, IPI00896419, IPI00556036, IPI00791097, IPI00798006, IPI00218192 | Isoform 2 of Inter-alpha-trypsin inhibitor heavy chain H4 | ITIH4 |
| FAHTVVTSR | IPI00896413, IPI00922043, IPI00896419, IPI00556036, IPI00791097, IPI00798006, IPI00218192 | Isoform 2 of Inter-alpha-trypsin inhibitor heavy chain H4 | ITIH4 |
| FKPTLSQQQK | IPI00896413, IPI00922043, IPI00896419, IPI00556036, IPI00791097, IPI00798006, IPI00218192 | Isoform 2 of Inter-alpha-trypsin inhibitor heavy chain H4 | ITIH4 |
| FSSHVGGTLGQFYQEVLWGSPAASDDGR | IPI00896413, IPI00922043, IPI00896419, IPI00556036, IPI00791097, IPI00798006, IPI00218192 | Isoform 2 of Inter-alpha-trypsin inhibitor heavy chain H4 | ITIH4 |
| GPDVLTATVSGK | IPI00896413, IPI00922043, IPI00896419, IPI00556036, IPI00791097, IPI00798006, IPI00218192 | Isoform 2 of Inter-alpha-trypsin inhibitor heavy chain H4 | ITIH4 |
| GSEMVVAGK | IPI00896413, IPI00922043, IPI00896419, IPI00556036, IPI00791097, IPI00798006, IPI00218192 | Isoform 2 of Inter-alpha-trypsin inhibitor heavy chain H4 | ITIH4 |
| ILDDLSPR | IPI00896413, IPI00922043, IPI00896419, IPI00556036, IPI00791097, IPI00798006, IPI00218192 | Isoform 2 of Inter-alpha-trypsin inhibitor heavy chain H4 | ITIH4 |
| ITFELVYEELLK | IPI00896413, IPI00922043, IPI00896419, IPI00556036, IPI00791097, IPI00798006, IPI00218192 | Isoform 2 of Inter-alpha-trypsin inhibitor heavy chain H4 | ITIH4 |
| ITFELVYEELLKR | IPI00896413, IPI00922043, IPI00896419, IPI00556036, IPI00791097, IPI00798006, IPI00218192 | Isoform 2 of Inter-alpha-trypsin inhibitor heavy chain H4 | ITIH4 |
| LALDNGGLAR | IPI00896413, IPI00922043, IPI00896419, IPI00556036, IPI00791097, IPI00798006, IPI00218192 | Isoform 2 of Inter-alpha-trypsin inhibitor heavy chain H4 | ITIH4 |
| LGVYELLLK | IPI00896413, IPI00922043, IPI00896419, IPI00556036, IPI00791097, IPI00798006, IPI00218192 | Isoform 2 of Inter-alpha-trypsin inhibitor heavy chain H4 | ITIH4 |
| LPEGSVSLIILLTDGDPTVGETNPR | IPI00896413, IPI00922043, IPI00896419, IPI00556036, IPI00791097, IPI00798006, IPI00218192 | Isoform 2 of Inter-alpha-trypsin inhibitor heavy chain H4 | ITIH4 |
| LQDRGPDVLTATVSGK | IPI00896413, IPI00922043, IPI00896419, IPI00556036, IPI00791097, IPI00798006, IPI00218192 | Isoform 2 of Inter-alpha-trypsin inhibitor heavy chain H4 | ITIH4 |
| LWAYLTIQQLLEQTVSASDADQQALR | IPI00896413, IPI00922043, IPI00896419, IPI00556036, IPI00791097, IPI00798006, IPI00218192 | Isoform 2 of Inter-alpha-trypsin inhibitor heavy chain H4 | ITIH4 |
| MNFRPGVLSSR | IPI00896413, IPI00922043, IPI00896419, IPI00556036, IPI00791097, IPI00798006, IPI00218192 | Isoform 2 of Inter-alpha-trypsin inhibitor heavy chain H4 | ITIH4 |
| NGIDIYSLTVDSR | IPI00896413, IPI00922043, IPI00896419, IPI00556036, IPI00791097, IPI00798006, IPI00218192 | Isoform 2 of Inter-alpha-trypsin inhibitor heavy chain H4 | ITIH4 |
| NMEQFQVSVSVAPNAK | IPI00896413, IPI00922043, IPI00896419, IPI00556036, IPI00791097, IPI00798006, IPI00218192 | Isoform 2 of Inter-alpha-trypsin inhibitor heavy chain H4 | ITIH4 |
| NPLVWVHASPEHVVVTR | IPI00896413, IPI00922043, IPI00896419, IPI00556036, IPI00791097, IPI00798006, IPI00218192 | Isoform 2 of Inter-alpha-trypsin inhibitor heavy chain H4 | ITIH4 |
| NVHSGSTFFK | IPI00896413, IPI00922043, IPI00896419, IPI00556036, IPI00791097, IPI00798006, IPI00218192 | Isoform 2 of Inter-alpha-trypsin inhibitor heavy chain H4 | ITIH4 |
| NVVFVIDK | IPI00896413, IPI00922043, IPI00896419, IPI00556036, IPI00791097, IPI00798006, IPI00218192 | Isoform 2 of Inter-alpha-trypsin inhibitor heavy chain H4 | ITIH4 |
| NVVFVIDKSGSMSGR | IPI00896413, IPI00922043, IPI00896419, IPI00556036, IPI00791097, IPI00798006, IPI00218192 | Isoform 2 of Inter-alpha-trypsin inhibitor heavy chain H4 | ITIH4 |
| QGPVNLLSDPEQGVEVTGQYER | IPI00896413, IPI00922043, IPI00896419, IPI00556036, IPI00791097, IPI00798006, IPI00218192 | Isoform 2 of Inter-alpha-trypsin inhibitor heavy chain H4 | ITIH4 |
| QLGLPGPPDVPDHAAYHPFR | IPI00896413, IPI00922043, IPI00896419, IPI00556036, IPI00791097, IPI00798006, IPI00218192 | Isoform 2 of Inter-alpha-trypsin inhibitor heavy chain H4 | ITIH4 |
| RLDYQEGPPGVEISCWSVEL | IPI00896413, IPI00922043, IPI00896419, IPI00556036, IPI00791097, IPI00798006, IPI00218192 | Isoform 2 of Inter-alpha-trypsin inhibitor heavy chain H4 | ITIH4 |
| RLGVYELLLK | IPI00896413, IPI00922043, IPI00896419, IPI00556036, IPI00791097, IPI00798006, IPI00218192 | Isoform 2 of Inter-alpha-trypsin inhibitor heavy chain H4 | ITIH4 |
| SFAAGIQALGGTNINDAMLMAVQLLDSSNQEER | IPI00896413, IPI00922043, IPI00896419, IPI00556036, IPI00791097, IPI00798006, IPI00218192 | Isoform 2 of Inter-alpha-trypsin inhibitor heavy chain H4 | ITIH4 |
| SPEQQETVLDGNLIIR | IPI00896413, IPI00922043, IPI00896419, IPI00556036, IPI00791097, IPI00798006, IPI00218192 | Isoform 2 of Inter-alpha-trypsin inhibitor heavy chain H4 | ITIH4 |
| TGLLLLSDPDK | IPI00896413, IPI00922043, IPI00896419, IPI00556036, IPI00791097, IPI00798006, IPI00218192 | Isoform 2 of Inter-alpha-trypsin inhibitor heavy chain H4 | ITIH4 |
| TGLLLLSDPDKVTIGLLFWDGR | IPI00896413, IPI00922043, IPI00896419, IPI00556036, IPI00791097, IPI00798006, IPI00218192 | Isoform 2 of Inter-alpha-trypsin inhibitor heavy chain H4 | ITIH4 |
| VRPQQLVK | IPI00896413, IPI00922043, IPI00896419, IPI00556036, IPI00791097, IPI00798006, IPI00218192 | Isoform 2 of Inter-alpha-trypsin inhibitor heavy chain H4 | ITIH4 |
| VTIGLLFWDGR | IPI00896413, IPI00922043, IPI00896419, IPI00556036, IPI00791097, IPI00798006, IPI00218192 | Isoform 2 of Inter-alpha-trypsin inhibitor heavy chain H4 | ITIH4 |
| WKETLFSVMPGLK | IPI00896413, IPI00922043, IPI00896419, IPI00556036, IPI00791097, IPI00798006, IPI00218192 | Isoform 2 of Inter-alpha-trypsin inhibitor heavy chain H4 | ITIH4 |
| YIFHNFMER | IPI00896413, IPI00922043, IPI00896419, IPI00556036, IPI00791097, IPI00798006, IPI00218192 | Isoform 2 of Inter-alpha-trypsin inhibitor heavy chain H4 | ITIH4 |
| APAVPAPIQAPSAILPLPGQSVER | IPI00218192 | Isoform 2 of Inter-alpha-trypsin inhibitor heavy chain H4 precursor | ITIH4 |
| VLVQGARSEER | IPI00645189, IPI00021449 | Isoform 1 of Inositol-trisphosphate 3-kinase B | ITPKB |
| LQLHLGEIR | IPI00289837 | Coiled-coil domain-containing protein 85A | KIAA1912 |
| NVCLLK | IPI00879984 | cDNA FLJ51250, highly similar to Plasma kallikrein | KLKB1 |
| EKGEIQNILQK | IPI00654888 | Plasma kallikrein | KLKB1 |
| EQPGVYTK | IPI00654888 | Plasma kallikrein | KLKB1 |
| GEIQNILQK | IPI00654888 | Plasma kallikrein | KLKB1 |
| LVGITSWGEGCAR | IPI00654888 | Plasma kallikrein | KLKB1 |
| MVCAGYK | IPI00654888 | Plasma kallikrein | KLKB1 |
| NNCLLK | IPI00654888 | Plasma kallikrein | KLKB1 |
| REQPGVYTK | IPI00654888 | Plasma kallikrein | KLKB1 |
| TSESGTPSSSTPQENTISGYSLLTCK | IPI00654888 | Plasma kallikrein | KLKB1 |
| VAEYMDWILEK | IPI00654888 | Plasma kallikrein | KLKB1 |
| VNIPLVTNEECQK | IPI00654888 | Plasma kallikrein | KLKB1 |
| AATGECTATVGK | IPI00797097, IPI00215894, IPI00797175, IPI00797833, IPI00924859, IPI00789376, IPI00032328 | Isoform HMW of Kininogen-1 | KNG1 |
| IASFSQNCDIYPGK | IPI00797097, IPI00215894, IPI00797175, IPI00797833, IPI00924859, IPI00789376, IPI00032328 | Isoform HMW of Kininogen-1 | KNG1 |
| IGEIKEETTSHLR | IPI00797097, IPI00215894, IPI00797175, IPI00797833, IPI00924859, IPI00789376, IPI00032328 | Isoform HMW of Kininogen-1 | KNG1 |
| KYFIDFVAR | IPI00797097, IPI00215894, IPI00797175, IPI00797833, IPI00924859, IPI00789376, IPI00032328 | Isoform HMW of Kininogen-1 | KNG1 |
| LGQSLDCNAEVYVVPWEK | IPI00797097, IPI00215894, IPI00797175, IPI00797833, IPI00924859, IPI00789376, IPI00032328 | Isoform HMW of Kininogen-1 | KNG1 |
| QVVAGLNFR | IPI00797097, IPI00215894, IPI00797175, IPI00797833, IPI00924859, IPI00789376, IPI00032328 | Isoform HMW of Kininogen-1 | KNG1 |
| RPPGFSPFR | IPI00797097, IPI00215894, IPI00797175, IPI00797833, IPI00924859, IPI00789376, IPI00032328 | Isoform HMW of Kininogen-1 | KNG1 |
| SLWNGDTGECTDNAYIDIQLR | IPI00797097, IPI00215894, IPI00797175, IPI00797833, IPI00924859, IPI00789376, IPI00032328 | Isoform HMW of Kininogen-1 | KNG1 |
| TVGSDTFYSFK | IPI00797097, IPI00215894, IPI00797175, IPI00797833, IPI00924859, IPI00789376, IPI00032328 | Isoform HMW of Kininogen-1 | KNG1 |
| TWQDCEYK | IPI00797097, IPI00215894, IPI00797175, IPI00797833, IPI00924859, IPI00789376, IPI00032328 | Isoform HMW of Kininogen-1 | KNG1 |
| YFIDFVAR | IPI00797097, IPI00215894, IPI00797175, IPI00797833, IPI00924859, IPI00789376, IPI00032328 | Isoform HMW of Kininogen-1 | KNG1 |
| YNSQNQSNNQFVLYR | IPI00797097, IPI00215894, IPI00797175, IPI00797833, IPI00924859, IPI00789376, IPI00032328 | Isoform HMW of Kininogen-1 | KNG1 |
| AEAESLYQSK | IPI00220327 | Keratin, type II cytoskeletal 1 | KRT1 |
| FSSCGGGGGSFGAGGGFGSR | IPI00220327 | Keratin, type II cytoskeletal 1 | KRT1 |
| GGSGGGGGGSSGGR | IPI00220327 | Keratin, type II cytoskeletal 1 | KRT1 |
| SLDLDSIIAEVK | IPI00220327 | Keratin, type II cytoskeletal 1 | KRT1 |
| SLNNQFASFIDK | IPI00220327 | Keratin, type II cytoskeletal 1 | KRT1 |
| THNLEPYFESFINNLR | IPI00220327 | Keratin, type II cytoskeletal 1 | KRT1 |
| WELLQQVDTSTR | IPI00220327 | Keratin, type II cytoskeletal 1 | KRT1 |
| NVSTGDVNVEMNAAPGVDLTQLLNNMR | IPI00009865 | Keratin, type I cytoskeletal 10 | KRT10 |
| SQYEQLAEQNR | IPI00009865 | Keratin, type I cytoskeletal 10 | KRT10 |
| VLDELTLTK | IPI00009865 | Keratin, type I cytoskeletal 10 | KRT10 |
| YCVQLSQIQAQISALEEQLQQIR | IPI00009865 | Keratin, type I cytoskeletal 10 | KRT10 |
| IIAATIENAQPILQIDNAR | IPI00795719, IPI00789536, IPI00929669, IPI00217963 | Keratin, type I cytoskeletal 16 | Krt16 |
| QSVEADINGLR | IPI00004550, IPI00328103, IPI00009866, IPI00171196, IPI00009865, IPI00550661 | Keratin, type I cytoskeletal 24 | KRT24 |
| FLEQQNQVLQTK | IPI00021304, IPI00376379, IPI00220327, IPI00293665 | Keratin, type II cytoskeletal 2 epidermal | KRT2A |
| YEELQITAGR | IPI00021304, IPI00376379, IPI00220327, IPI00293665 | Keratin, type II cytoskeletal 2 epidermal | KRT2A |
| YEELQVTVGR | IPI00021304 | Keratin, type II cytoskeletal 2 epidermal | KRT2A |
| VQALEEANNDLENK | IPI00019359 | Keratin, type I cytoskeletal 9 | KRT9 |
| VAPQQDDLDSPQQISISNAEAR | IPI00218725, IPI00942809, IPI00479834, IPI00940615, IPI00844276, IPI00873889 | Laminin subunit alpha-2 | LAMA2 |
| CGNTIPDDDNQVVSLSPGSR | IPI00013976, IPI00852733, IPI00852737, IPI00853454 | Laminin subunit beta-1 precursor | LAMB1 |
| GIETPQCDQSTGQCVCVEGVEGPR | IPI00013976, IPI00852733, IPI00852737, IPI00853454 | Laminin subunit beta-1 precursor | LAMB1 |
| FFLQGIQLNTILPDAR | IPI00884105, IPI00908532 | Lysosome-associated membrane glycoprotein 1 | LAMP1 |
| GILTVDELLAIR | IPI00922445, IPI00739827, IPI00216172, IPI00009030 | Isoform LAMP-2A of Lysosome-associated membrane glycoprotein 2 | LAMP2 |
| IPLNDLFR | IPI00922445, IPI00739827, IPI00216172, IPI00009030 | Isoform LAMP-2A of Lysosome-associated membrane glycoprotein 2 | LAMP2 |
| GFSVVADTPELQR | IPI00883946, IPI00909481, IPI00909678, IPI00000861, IPI00909939, IPI00386803 | Isoform 1 of LIM and SH3 domain protein 1 | LASP1 |
| ATAQMLEVMFK | IPI00032311 | Lipopolysaccharide-binding protein precursor | LBP |
| GLQYAAQEGLLALQSELLR | IPI00032311 | Lipopolysaccharide-binding protein precursor | LBP |
| SFRPFVPR | IPI00032311 | Lipopolysaccharide-binding protein precursor | LBP |
| DLLAGLPAPGVEVYCLYGVGLPTPR | IPI00022331 | Phosphatidylcholine-sterol acyltransferase precursor | LCAT |
| ITTTSPWMFPSR | IPI00022331 | Phosphatidylcholine-sterol acyltransferase precursor | LCAT |
| LAGYLHTLVQNLVNNGYVR | IPI00022331 | Phosphatidylcholine-sterol acyltransferase precursor | LCAT |
| LEPGQQEEYYR | IPI00022331 | Phosphatidylcholine-sterol acyltransferase precursor | LCAT |
| SSGLVSNAPGVQIR | IPI00022331 | Phosphatidylcholine-sterol acyltransferase precursor | LCAT |
| STELCGLWQGR | IPI00022331 | Phosphatidylcholine-sterol acyltransferase precursor | LCAT |
| TYIYDHGFPYTDPVGVLYEDGDDTVATR | IPI00022331 | Phosphatidylcholine-sterol acyltransferase precursor | LCAT |
| VPLQQNFQDNQFQGK | IPI00643623, IPI00299547 | Neutrophil gelatinase-associated lipocalin | LCN2 |
| WYVVGLAGNAILR | IPI00643623, IPI00299547 | Neutrophil gelatinase-associated lipocalin | LCN2 |
| AACLPLPGYR | IPI00646259, IPI00643270, IPI00908503, IPI00216694, IPI00010471, IPI00032304, IPI00909658 | Plastin-2 | LCP1 |
| AECMLQQAER | IPI00010471 | Plastin-2 | LCP1 |
| EGICAIGGTSEQSSVGTQHSYSEEEK | IPI00646259, IPI00643270, IPI00908503, IPI00216694, IPI00010471, IPI00032304, IPI00909658 | Plastin-2 | LCP1 |
| EITENLMATGDLDQDGR | IPI00646259, IPI00643270, IPI00908503, IPI00216694, IPI00010471, IPI00032304, IPI00909658 | Plastin-2 | LCP1 |
| FSLVGIGGQDLNEGNR | IPI00646259, IPI00643270, IPI00908503, IPI00216694, IPI00010471, IPI00032304, IPI00909658 | Plastin-2 | LCP1 |
| GDEEGVPAVVIDMSGLR | IPI00010471 | Plastin-2 | LCP1 |
| GSVSDEEMMELR | IPI00646259, IPI00643270, IPI00908503, IPI00216694, IPI00010471, IPI00032304, IPI00909658 | Plastin-2 | LCP1 |
| HVIPMNPNTNDLFNAVGDGIVLCK | IPI00646259, IPI00643270, IPI00908503, IPI00216694, IPI00010471, IPI00032304, IPI00909658 | Plastin-2 | LCP1 |
| IGLFADIELSR | IPI00646259, IPI00643270, IPI00908503, IPI00216694, IPI00010471, IPI00032304, IPI00909658 | Plastin-2 | LCP1 |
| ISFDEFIK | IPI00646259, IPI00643270, IPI00908503, IPI00216694, IPI00010471, IPI00032304, IPI00909658 | Plastin-2 | LCP1 |
| ISTSLPVLDLIDAIQPGSINYDLLK | IPI00646259, IPI00643270, IPI00908503, IPI00216694, IPI00010471, IPI00032304, IPI00909658 | Plastin-2 | LCP1 |
| LNLAFIANLFNR | IPI00010471 | Plastin-2 | LCP1 |
| LSPEELLLR | IPI00646259, IPI00643270, IPI00908503, IPI00216694, IPI00010471, IPI00032304, IPI00909658 | Plastin-2 | LCP1 |
| MINLSVPDTIDER | IPI00646259, IPI00643270, IPI00908503, IPI00216694, IPI00010471, IPI00032304, IPI00909658 | Plastin-2 | LCP1 |
| MVMTVFACLMGK | IPI00646259, IPI00643270, IPI00908503, IPI00216694, IPI00010471, IPI00032304, IPI00909658 | Plastin-2 | LCP1 |
| NEALIALLR | IPI00010471 | Plastin-2 | LCP1 |
| QFVTATDVVR | IPI00010471 | Plastin-2 | LCP1 |
| TLTLALIWQLMR | IPI00646259, IPI00643270, IPI00908503, IPI00216694, IPI00010471, IPI00032304, IPI00909658 | Plastin-2 | LCP1 |
| VDTDGNGYISFNELNDLFK | IPI00646259, IPI00643270, IPI00908503, IPI00216694, IPI00010471, IPI00032304, IPI00909658 | Plastin-2 | LCP1 |
| VNDDIIVNWVNETLR | IPI00646259, IPI00643270, IPI00908503, IPI00216694, IPI00010471, IPI00032304, IPI00909658 | Plastin-2 | LCP1 |
| VNHLYSDLSDALVIFQLYEK | IPI00010471 | Plastin-2 | LCP1 |
| VNKPPYPK | IPI00646259, IPI00643270, IPI00908503, IPI00216694, IPI00010471, IPI00032304, IPI00909658 | Plastin-2 | LCP1 |
| VYALPEDLVEVNPK | IPI00646259, IPI00643270, IPI00908503, IPI00216694, IPI00010471, IPI00032304, IPI00909658 | Plastin-2 | LCP1 |
| YAFVNWINK | IPI00646259, IPI00643270, IPI00908503, IPI00216694, IPI00010471, IPI00032304, IPI00909658 | Plastin-2 | LCP1 |
| YPALHKPENQDIDWGALEGETR | IPI00010471 | Plastin-2 | LCP1 |
| LVIITAGAR | IPI00607708, IPI00148061, IPI00554498, IPI00748298, IPI00910754, IPI00795075, IPI00939286, IPI00788938, IPI00908791, IPI00789173, IPI00219217, IPI00016768, IPI00217966 | L-lactate dehydrogenase A-like 6B | LDHB |
| SLADELALVDVLEDK | IPI00607708, IPI00148061, IPI00554498, IPI00748298, IPI00910754, IPI00795075, IPI00939286, IPI00788938, IPI00908791, IPI00789173, IPI00219217, IPI00016768, IPI00217966 | L-lactate dehydrogenase A-like 6B | LDHB |
| VIGSGCNLDSAR | IPI00607708, IPI00148061, IPI00554498, IPI00748298, IPI00910754, IPI00795075, IPI00939286, IPI00788938, IPI00908791, IPI00789173, IPI00219217, IPI00016768, IPI00217966 | L-lactate dehydrogenase A-like 6B | LDHB |
| GMYGIENEVFLSLPCILNAR | IPI00219217 | L-lactate dehydrogenase B chain | LDHB |
| AVGSIAYLFFTNR | IPI00000070, IPI00908663 | Low-density lipoprotein receptor | LDLR |
| NVIQISNDLENLR | IPI00012760 | Leptin precursor | LEP |
| VTGLDFIPGLHPILTLSK | IPI00012760 | Leptin precursor | LEP |
| AAFGQGSGPIMLDEVQCTGTEASLADCK | IPI00023673, IPI00794873, IPI00908762, IPI00902654, IPI00796246 | Galectin-3-binding protein | LGALS3BP |
| AVDTWSWGER | IPI00023673, IPI00794873, IPI00908762, IPI00902654, IPI00796246 | Galectin-3-binding protein | LGALS3BP |
| GQWGTVCDNLWDLTDASVVCR | IPI00023673, IPI00794873, IPI00908762, IPI00902654, IPI00796246 | Galectin-3-binding protein | LGALS3BP |
| SDLAVPSELALLK | IPI00023673, IPI00794873, IPI00908762, IPI00902654, IPI00796246 | Galectin-3-binding protein | LGALS3BP |
| TLQALEFHTVPFQLLAR | IPI00023673, IPI00794873, IPI00908762, IPI00902654, IPI00796246 | Galectin-3-binding protein | LGALS3BP |
| YSSDYFQAPSDYR | IPI00023673, IPI00794873, IPI00908762, IPI00902654, IPI00796246 | Galectin-3-binding protein | LGALS3BP |
| ELSEALGQIFDSQR | IPI00023673 | Galectin-3-binding protein precursor | LGALS3BP |
| AIFSVGPVSPSR | IPI00792726, IPI00915286, IPI00641337, IPI00893611, IPI00917084, IPI00789466, IPI00916786, IPI00853070, IPI00646137, IPI00916871, IPI00020967, IPI00329104, IPI00893824, IPI00385703, IPI00852687, IPI00894149, IPI00657948, IPI00853520, IPI00157163, IPI00643313, IPI00385705, IPI00009984, IPI00657726, IPI00935629, IPI00658078, IPI00027972, IPI00375194 | Isoform 1 of Leukocyte immunoglobulin-like receptor subfamily A member 1 | LILRA1 |
| GWSWAIFSVGPVSPSR | IPI00792726, IPI00915286, IPI00641337, IPI00893611, IPI00917084, IPI00789466, IPI00916786, IPI00853070, IPI00646137, IPI00916871, IPI00020967, IPI00329104, IPI00893824, IPI00385703, IPI00852687, IPI00894149, IPI00657948, IPI00853520, IPI00157163, IPI00643313, IPI00385705, IPI00009984, IPI00657726, IPI00935629, IPI00658078, IPI00027972, IPI00375194 | Isoform 1 of Leukocyte immunoglobulin-like receptor subfamily A member 1 | LILRA1 |
| LTFQCGSDAGYDR | IPI00792726, IPI00915286, IPI00641337, IPI00893611, IPI00917084, IPI00789466, IPI00916786, IPI00853070, IPI00646137, IPI00916871, IPI00020967, IPI00329104, IPI00893824, IPI00385703, IPI00852687, IPI00894149, IPI00657948, IPI00853520, IPI00157163, IPI00643313, IPI00385705, IPI00009984, IPI00657726, IPI00935629, IPI00658078, IPI00027972, IPI00375194 | Isoform 1 of Leukocyte immunoglobulin-like receptor subfamily A member 1 | LILRA1 |
| GPSQALFPVGPVTPSCR | IPI00829955, IPI00893901, IPI00009961 | Isoform 1 of Leukocyte immunoglobulin-like receptor subfamily B member 5 | LILRB5 |
| DNFHGLAIFLDTYPNDETTER | IPI00009950 | Vesicular integral-membrane protein VIP36 precursor | LMAN2 |
| TIAVLLDDILQR | IPI00658073, IPI00061448, IPI00917602 | Coiled-coil domain-containing protein 126 | LOC90693 |
| IECLNDIK | IPI00021956 | Lipin-2 | LPIN2 |
| AAELFSQWMESSGK | IPI00465261, IPI00747807 | Isoform 1 of Endoplasmic reticulum aminopeptidase 2 | LRAP |
| TQNLAALLHAIAR | IPI00465261, IPI00747807 | Isoform 1 of Endoplasmic reticulum aminopeptidase 2 | LRAP |
| VGLIHDVFQLVGAGR | IPI00465261, IPI00747807 | Isoform 1 of Endoplasmic reticulum aminopeptidase 2 | LRAP |
| ALGHLDLSGNR | IPI00022417 | Leucine-rich alpha-2-glycoprotein precursor | LRG1 |
| CAGPEAVK | IPI00022417 | Leucine-rich alpha-2-glycoprotein precursor | LRG1 |
| DCQVFR | IPI00022417 | Leucine-rich alpha-2-glycoprotein precursor | LRG1 |
| DGFDISGNPWICDQNLSDLYR | IPI00022417 | Leucine-rich alpha-2-glycoprotein precursor | LRG1 |
| DLLLPQPDLR | IPI00022417 | Leucine-rich alpha-2-glycoprotein precursor | LRG1 |
| ENQLEVLEVSWLHGLK | IPI00022417 | Leucine-rich alpha-2-glycoprotein precursor | LRG1 |
| GPLQLER | IPI00022417 | Leucine-rich alpha-2-glycoprotein precursor | LRG1 |
| GQTLLAVAK | IPI00022417 | Leucine-rich alpha-2-glycoprotein precursor | LRG1 |
| LHLEGNK | IPI00022417 | Leucine-rich alpha-2-glycoprotein precursor | LRG1 |
| LQELHLSSNGLESLSPEFLRPVPQLR | IPI00022417 | Leucine-rich alpha-2-glycoprotein precursor | LRG1 |
| LQVLGK | IPI00022417 | Leucine-rich alpha-2-glycoprotein precursor | LRG1 |
| LQVLGKDLLLPQPDLR | IPI00022417 | Leucine-rich alpha-2-glycoprotein precursor | LRG1 |
| NALTGLPPGLFQASATLDTLVLK | IPI00022417 | Leucine-rich alpha-2-glycoprotein precursor | LRG1 |
| QLDMLDLSNNSLASVPEGLWASLGQPNWDMR | IPI00022417 | Leucine-rich alpha-2-glycoprotein precursor | LRG1 |
| TLDLGENQLETLPPDLLR | IPI00022417 | Leucine-rich alpha-2-glycoprotein precursor | LRG1 |
| VAAGAFQGLR | IPI00022417 | Leucine-rich alpha-2-glycoprotein precursor | LRG1 |
| WLQAQK | IPI00022417 | Leucine-rich alpha-2-glycoprotein precursor | LRG1 |
| YLFLNGNK | IPI00022417 | Leucine-rich alpha-2-glycoprotein precursor | LRG1 |
| IVFPHGITLDLVSR | IPI00020557, IPI00448904 | Prolow-density lipoprotein receptor-related protein 1 | LRP1 |
| ILWIDAR | IPI00020557 | Prolow-density lipoprotein receptor-related protein 1 precursor | LRP1 |
| LDGLCIPLR | IPI00020557 | Prolow-density lipoprotein receptor-related protein 1 precursor | LRP1 |
| LILTPFER | IPI00300094, IPI00926855 | Large subunit GTPase 1 homolog | LSG1 |
| ELVALMSAIR | IPI00793812, IPI00514090, IPI00219077, IPI00790872, IPI00794758 | Leukotriene A4 hydrolase variant | LTA4H |
| EQDAPVAGLQPVER | IPI00292150 | Latent-transforming growth factor beta-binding protein 2 precursor | LTBP2 |
| MACVDINECDEAEAASPLCVNAR | IPI00873371, IPI00783492, IPI00395783, IPI00792792 | Isoform 4 of Latent-transforming growth factor beta-binding protein 4 | LTBP4 |
| EDAIWNLLR | IPI00298860, IPI00925547, IPI00789477, IPI00903112 | Lactoferrin | LTF |
| ESTVFEDLSDEAERDEYELLCPDNTR | IPI00298860, IPI00925547, IPI00789477, IPI00903112 | Lactoferrin | LTF |
| SQQSSDPDPNCVDRPVEGYLAVAVVR | IPI00298860, IPI00925547, IPI00789477, IPI00903112 | Lactoferrin | LTF |
| FNALQYLR | IPI00794403, IPI00020986, IPI00796888 | Lumican precursor | LUM |
| ILGPLSYSK | IPI00020986 | Lumican precursor | LUM |
| ISETSLPPDMYECLR | IPI00020986 | Lumican precursor | LUM |
| ISNIPDEYFK | IPI00794403, IPI00020986, IPI00796888 | Lumican precursor | LUM |
| LKEDAVSAAFK | IPI00794403, IPI00020986, IPI00796888 | Lumican precursor | LUM |
| LKEDAVSAAFKGLK | IPI00794403, IPI00020986, IPI00796888 | Lumican precursor | LUM |
| LPSGLPVSLLTLYLDNNK | IPI00794403, IPI00020986, IPI00796888 | Lumican precursor | LUM |
| NIPTVNENLENYYLEVNQLEK | IPI00020986 | Lumican precursor | LUM |
| NNQIDHIDEK | IPI00794403, IPI00020986, IPI00796888 | Lumican precursor | LUM |
| RFNALQYLR | IPI00794403, IPI00020986, IPI00796888 | Lumican precursor | LUM |
| SLEDLQLTHNK | IPI00794403, IPI00020986, IPI00796888 | Lumican precursor | LUM |
| SLEYLDLSFNQIAR | IPI00794403, IPI00020986, IPI00796888 | Lumican precursor | LUM |
| SVPMVPPGIK | IPI00794403, IPI00020986, IPI00796888 | Lumican precursor | LUM |
| GISLANWMCLAK | IPI00019038 | Lysozyme C precursor | LYZ |
| STDYGIFQINSR | IPI00019038 | Lysozyme C precursor | LYZ |
| TPGAVNACHLSCSALLQDNIADAVACAK | IPI00019038 | Lysozyme C precursor | LYZ |
| WESGYNTR | IPI00019038 | Lysozyme C precursor | LYZ |
| YWCNDGK | IPI00019038 | Lysozyme C precursor | LYZ |
| FVGGLLSAYYLSGEEIFR | IPI00844511, IPI00439446 | MAN1A1 protein | MAN1A1 |
| GLPPVDFVPPIGVESR | IPI00844511, IPI00439446 | MAN1A1 protein | MAN1A1 |
| FDGGVEAIATR | IPI00844511 | Mannosyl-oligosaccharide 1,2-alpha-mannosidase IA | MAN1A1 |
| KMYFDAVQAIETHLIR | IPI00844511 | Mannosyl-oligosaccharide 1,2-alpha-mannosidase IA | MAN1A1 |
| MYFDAVQAIETHLIR | IPI00844511 | Mannosyl-oligosaccharide 1,2-alpha-mannosidase IA | MAN1A1 |
| AAEILYYFALR | IPI00003802 | Alpha-mannosidase 2 | MAN2A1 |
| VLLAPLGDDFR | IPI00003802 | Alpha-mannosidase 2 | MAN2A1 |
| DACAGDSGGPMVTLNR | IPI00299307 | Complement-activating component of Ra-reactive factor precursor | MASP1 |
| DSDLLSPSDFK | IPI00299307 | Complement-activating component of Ra-reactive factor precursor | MASP1 |
| EGGKDACAGDSGGPMVTLNR | IPI00299307 | Complement-activating component of Ra-reactive factor precursor | MASP1 |
| FPETLMEIEIPIVDHSTCQK | IPI00299307 | Complement-activating component of Ra-reactive factor precursor | MASP1 |
| GQWYLVGTVSWGDDCGK | IPI00299307 | Complement-activating component of Ra-reactive factor precursor | MASP1 |
| SLPTCLPVCGLPK | IPI00299307 | Complement-activating component of Ra-reactive factor precursor | MASP1 |
| AAGNECPELQPPVHGK | IPI00924973, IPI00924706, IPI00216882, IPI00925177, IPI00299307, IPI00290283 | Isoform 3 of Mannan-binding lectin serine protease 1 | MASP1 |
| APEPISTQSHSVLILFHSDNSGENR | IPI00924973, IPI00924706, IPI00216882, IPI00925177, IPI00299307, IPI00290283 | Isoform 3 of Mannan-binding lectin serine protease 1 | MASP1 |
| APGELEHGLITFSTR | IPI00924973, IPI00924706, IPI00216882, IPI00925177, IPI00299307, IPI00290283 | Isoform 3 of Mannan-binding lectin serine protease 1 | MASP1 |
| DNVEMDTFQIECLK | IPI00924973, IPI00924706, IPI00216882, IPI00925177, IPI00299307, IPI00290283 | Isoform 3 of Mannan-binding lectin serine protease 1 | MASP1 |
| DQVLVSCDTGYK | IPI00924973, IPI00924706, IPI00216882, IPI00925177, IPI00299307, IPI00290283 | Isoform 3 of Mannan-binding lectin serine protease 1 | MASP1 |
| DTCLGDSGGAFVIFDDLSQR | IPI00924973, IPI00924706, IPI00216882, IPI00925177, IPI00299307, IPI00290283 | Isoform 3 of Mannan-binding lectin serine protease 1 | MASP1 |
| ETTDTEQTPGQEVVLSPGSFMSITFR | IPI00924973, IPI00924706, IPI00216882, IPI00925177, IPI00299307, IPI00290283 | Isoform 3 of Mannan-binding lectin serine protease 1 | MASP1 |
| FTGFDAHYMAVDVDECK | IPI00924973, IPI00924706, IPI00216882, IPI00925177, IPI00299307, IPI00290283 | Isoform 3 of Mannan-binding lectin serine protease 1 | MASP1 |
| LPVVPHAECK | IPI00924973, IPI00924706, IPI00216882, IPI00925177, IPI00299307, IPI00290283 | Isoform 3 of Mannan-binding lectin serine protease 1 | MASP1 |
| NAEPGLFPWQALIVVEDTSR | IPI00924973, IPI00924706, IPI00216882, IPI00925177, IPI00299307, IPI00290283 | Isoform 3 of Mannan-binding lectin serine protease 1 | MASP1 |
| SLPTCLPECGQPSR | IPI00924973, IPI00924706, IPI00216882, IPI00925177, IPI00299307, IPI00290283 | Isoform 3 of Mannan-binding lectin serine protease 1 | MASP1 |
| TGVITSPDFPNPYPK | IPI00924973, IPI00924706, IPI00216882, IPI00925177, IPI00299307, IPI00290283 | Isoform 3 of Mannan-binding lectin serine protease 1 | MASP1 |
| TLSDVLQYVK | IPI00924973, IPI00924706, IPI00216882, IPI00925177, IPI00299307, IPI00290283 | Isoform 3 of Mannan-binding lectin serine protease 1 | MASP1 |
| VECSDNLFTQR | IPI00924973, IPI00924706, IPI00216882, IPI00925177, IPI00299307, IPI00290283 | Isoform 3 of Mannan-binding lectin serine protease 1 | MASP1 |
| VETEDQVLATFCGR | IPI00924973, IPI00924706, IPI00216882, IPI00925177, IPI00299307, IPI00290283 | Isoform 3 of Mannan-binding lectin serine protease 1 | MASP1 |
| VLGPFCGEK | IPI00924973, IPI00924706, IPI00216882, IPI00925177, IPI00299307, IPI00290283 | Isoform 3 of Mannan-binding lectin serine protease 1 | MASP1 |
| VLKDNVEMDTFQIECLK | IPI00924973, IPI00924706, IPI00216882, IPI00925177, IPI00299307, IPI00290283 | Isoform 3 of Mannan-binding lectin serine protease 1 | MASP1 |
| VSNYVDWVWEQMGLPQSVVEPQVER | IPI00924973, IPI00924706, IPI00216882, IPI00925177, IPI00299307, IPI00290283 | Isoform 3 of Mannan-binding lectin serine protease 1 | MASP1 |
| WVVQGLVSWGGPEECGSK | IPI00924973, IPI00924706, IPI00216882, IPI00925177, IPI00299307, IPI00290283 | Isoform 3 of Mannan-binding lectin serine protease 1 | MASP1 |
| YSCQEPYYK | IPI00924973, IPI00924706, IPI00216882, IPI00925177, IPI00299307, IPI00290283 | Isoform 3 of Mannan-binding lectin serine protease 1 | MASP1 |
| APGKDTFYSLGSSLDITFR | IPI00871597, IPI00306378, IPI00294713 | Isoform 1 of Mannan-binding lectin serine protease 2 precursor | MASP2 |
| LASPGFPGEYANDQER | IPI00871597, IPI00306378, IPI00294713 | Isoform 1 of Mannan-binding lectin serine protease 2 precursor | MASP2 |
| LYFTHFDLELSHLCEYDFVK | IPI00871597, IPI00306378, IPI00294713 | Isoform 1 of Mannan-binding lectin serine protease 2 precursor | MASP2 |
| SLPVCEPVCGLSAR | IPI00294713 | Isoform 1 of Mannan-binding lectin serine protease 2 precursor | MASP2 |
| VLATLCGQESTDTER | IPI00871597, IPI00306378, IPI00294713 | Isoform 1 of Mannan-binding lectin serine protease 2 precursor | MASP2 |
| WPEPVFGR | IPI00871597, IPI00306378, IPI00294713 | Isoform 1 of Mannan-binding lectin serine protease 2 precursor | MASP2 |
| WTLTAPPGYR | IPI00871597, IPI00306378, IPI00294713 | Isoform 1 of Mannan-binding lectin serine protease 2 precursor | MASP2 |
| HGATVLTALGGILK | IPI00879081, IPI00878918, IPI00879413, IPI00878623, IPI00217493, IPI00879863 | Myoglobin | MB |
| VEADIPGHGQEVLIR | IPI00879081, IPI00878918, IPI00879413, IPI00878623, IPI00217493, IPI00879863 | Myoglobin | MB |
| FFLTNGEIMTFEK | IPI00004373 | Mannose-binding protein C precursor | MBL2 |
| LTYTNWNEGEPNNAGSDEDCVLLLK | IPI00004373 | Mannose-binding protein C precursor | MBL2 |
| TEGQFVDLTGNR | IPI00004373 | Mannose-binding protein C precursor | MBL2 |
| WLTFSLGK | IPI00004373 | Mannose-binding protein C precursor | MBL2 |
| APEEPNIQVNPLGIPVNSKEPEEVATCVGR | IPI00016334 | Isoform 1 of Cell surface glycoprotein MUC18 precursor | MCAM |
| EAEEETTNDNGVLVLEPAR | IPI00016334, IPI00445227 | Isoform 1 of Cell surface glycoprotein MUC18 precursor | MCAM |
| EPEEVATCVGR | IPI00016334 | Isoform 1 of Cell surface glycoprotein MUC18 precursor | MCAM |
| GATLALTQVTPQDER | IPI00016334 | Isoform 1 of Cell surface glycoprotein MUC18 precursor | MCAM |
| GPVLQLHDLK | IPI00016334, IPI00445227 | Isoform 1 of Cell surface glycoprotein MUC18 precursor | MCAM |
| FVEGLPINDFSR | IPI00291005, IPI00915869, IPI00916111, IPI00916861 | Malate dehydrogenase, cytoplasmic | MDH1 |
| LTLGGPDPR | IPI00384225 | Meteorin precursor | METRN |
| QVEVHQEPGAAVPR | IPI00443799, IPI00884960 | Isoform 1 of Putative sodium-coupled neutral amino acid transporter 10 | MGC15523 |
| ATIADLILSALER | IPI00166766, IPI00432405, IPI00886722 | hypothetical protein LOC146556 isoform 2 | MGC45438 |
| NANTFISPQQR | IPI00028714 | Matrix Gla protein precursor | MGP |
| DILQSCQTSEECELAR | IPI00293748 | Isoform 1 of Multiple inositol polyphosphate phosphatase 1 precursor | MINPP1 |
| SPWCDVFDIDDAK | IPI00293748 | Isoform 1 of Multiple inositol polyphosphate phosphatase 1 precursor | MINPP1 |
| SSCTLFQDIFQHLDK | IPI00293748 | Isoform 1 of Multiple inositol polyphosphate phosphatase 1 precursor | MINPP1 |
| VLEYLNDLK | IPI00293748 | Isoform 1 of Multiple inositol polyphosphate phosphatase 1 precursor | MINPP1 |
| DLGAALADWPLWYADWMDGQLVEK | IPI00293748, IPI00607763, IPI00028553 | Isoform 2 of Multiple inositol polyphosphate phosphatase 1 | MINPP1 |
| DPVASSLSPYFGTK | IPI00293748, IPI00607763, IPI00028553 | Isoform 2 of Multiple inositol polyphosphate phosphatase 1 | MINPP1 |
| LASLFPALFSR | IPI00293748, IPI00607763, IPI00028553 | Isoform 2 of Multiple inositol polyphosphate phosphatase 1 | MINPP1 |
| QLHGLLQAR | IPI00293748, IPI00607763, IPI00028553 | Isoform 2 of Multiple inositol polyphosphate phosphatase 1 | MINPP1 |
| EDSTADDSK | IPI00004233 | Isoform Long of Antigen KI-67 | MKI67 |
| LWDFIENR | IPI00927618, IPI00396406, IPI00927374 | Makorin-2 | MKRN2 |
| AFQVWSDVTPLR | IPI00895858, IPI00027780 | 72 kDa type IV collagenase | MMP2 |
| CGNPDVANYNFFPR | IPI00895858, IPI00027780 | 72 kDa type IV collagenase | MMP2 |
| FFGLPQTGDLDQNTIETMR | IPI00895858, IPI00027780 | 72 kDa type IV collagenase | MMP2 |
| QDIVFDGIAQIR | IPI00895858, IPI00027780 | 72 kDa type IV collagenase | MMP2 |
| VDAAFNWSK | IPI00895858, IPI00027780 | 72 kDa type IV collagenase | MMP2 |
| KSNEQATSLNTVGGTGGIGGVGGTGGVGNR | IPI00012269, IPI00745154 | Multimerin-1 precursor | MMRN1 |
| EAEPLVDIR | IPI00908953, IPI00015525 | Multimerin-2 | MMRN2 |
| IGLDLPALNMQR | IPI00877025, IPI00236556, IPI00007244, IPI00236554 | Isoform H17 of Myeloperoxidase precursor | MPO |
| VGPLLACIIGTQFR | IPI00877025, IPI00236556, IPI00007244, IPI00236554 | Isoform H17 of Myeloperoxidase precursor | MPO |
| IFGFMEEER | IPI00027848, IPI00473066 | Macrophage mannose receptor 1 precursor | MRC1L1 |
| TNFWIGLFR | IPI00027848, IPI00473066 | Macrophage mannose receptor 1 precursor | MRC1L1 |
| TPLWIGLAGEEGSR | IPI00005707 | Macrophage mannose receptor 2 precursor | MRC2 |
| DNAMLEYLK | IPI00872814, IPI00903145, IPI00843975, IPI00017367, IPI00384282, IPI00872684, IPI00219365 | Radixin, isoform CRA_a | MSN |
| IGFPWSEIR | IPI00872814, IPI00903145, IPI00843975, IPI00017367, IPI00384282, IPI00872684, IPI00219365 | Radixin, isoform CRA_a | MSN |
| KTQEQLALEMAELTAR | IPI00872814, IPI00903145, IPI00843975, IPI00017367, IPI00384282, IPI00872684, IPI00219365 | Radixin, isoform CRA_a | MSN |
| AAFCYQIR | IPI00922041, IPI00847702, IPI00873854, IPI00925679, IPI00922577, IPI00292218, IPI00384647, IPI00925822, IPI00925898, IPI00718805, IPI00884434, IPI00925540, IPI00908726, IPI00902670 | Hepatocyte growth factor-like protein | MST1 |
| CADDQPPSILDPPDQVQFEK | IPI00922041, IPI00847702, IPI00873854, IPI00925679, IPI00922577, IPI00292218, IPI00384647, IPI00925822, IPI00925898, IPI00718805, IPI00884434, IPI00925540, IPI00908726, IPI00902670 | Hepatocyte growth factor-like protein | MST1 |
| CEIAGWGETK | IPI00922041, IPI00847702, IPI00873854, IPI00925679, IPI00922577, IPI00292218, IPI00384647, IPI00925822, IPI00925898, IPI00718805, IPI00884434, IPI00925540, IPI00908726, IPI00902670 | Hepatocyte growth factor-like protein | MST1 |
| EAACVWCNGEEYR | IPI00922041, IPI00847702, IPI00873854, IPI00925679, IPI00922577, IPI00292218, IPI00384647, IPI00925822, IPI00925898, IPI00718805, IPI00884434, IPI00925540, IPI00908726, IPI00902670 | Hepatocyte growth factor-like protein | MST1 |
| EQWILTAR | IPI00922041, IPI00847702, IPI00873854, IPI00925679, IPI00922577, IPI00292218, IPI00384647, IPI00925822, IPI00925898, IPI00718805, IPI00884434, IPI00925540, IPI00908726, IPI00902670 | Hepatocyte growth factor-like protein | MST1 |
| FLDQGLDDNYCR | IPI00922041, IPI00847702, IPI00873854, IPI00925679, IPI00922577, IPI00292218, IPI00384647, IPI00925822, IPI00925898, IPI00718805, IPI00884434, IPI00925540, IPI00908726, IPI00902670 | Hepatocyte growth factor-like protein | MST1 |
| GTELQHLLHAVVPGPWQEDVADAEECAGR | IPI00922041, IPI00847702, IPI00873854, IPI00925679, IPI00922577, IPI00292218, IPI00384647, IPI00925822, IPI00925898, IPI00718805, IPI00884434, IPI00925540, IPI00908726, IPI00902670 | Hepatocyte growth factor-like protein | MST1 |
| GTMATTVGGLPCQAWSHK | IPI00922041, IPI00847702, IPI00873854, IPI00925679, IPI00922577, IPI00292218, IPI00384647, IPI00925822, IPI00925898, IPI00718805, IPI00884434, IPI00925540, IPI00908726, IPI00902670 | Hepatocyte growth factor-like protein | MST1 |
| MVCGPSGSQLVLLK | IPI00922041, IPI00847702, IPI00873854, IPI00925679, IPI00922577, IPI00292218, IPI00384647, IPI00925822, IPI00925898, IPI00718805, IPI00884434, IPI00925540, IPI00908726, IPI00902670 | Hepatocyte growth factor-like protein | MST1 |
| NGLEENFCR | IPI00922041, IPI00847702, IPI00873854, IPI00925679, IPI00922577, IPI00292218, IPI00384647, IPI00925822, IPI00925898, IPI00718805, IPI00884434, IPI00925540, IPI00908726, IPI00902670 | Hepatocyte growth factor-like protein | MST1 |
| NPDGDPGGPWCYTTDPAVR | IPI00922041, IPI00847702, IPI00873854, IPI00925679, IPI00922577, IPI00292218, IPI00384647, IPI00925822, IPI00925898, IPI00718805, IPI00884434, IPI00925540, IPI00908726, IPI00902670 | Hepatocyte growth factor-like protein | MST1 |
| NPDGDSHGPWCYTMDPR | IPI00922041, IPI00847702, IPI00873854, IPI00925679, IPI00922577, IPI00292218, IPI00384647, IPI00925822, IPI00925898, IPI00718805, IPI00884434, IPI00925540, IPI00908726, IPI00902670 | Hepatocyte growth factor-like protein | MST1 |
| NPDGSEAPWCFTLRPGMR | IPI00922041, IPI00847702, IPI00873854, IPI00925679, IPI00922577, IPI00292218, IPI00384647, IPI00925822, IPI00925898, IPI00718805, IPI00884434, IPI00925540, IPI00908726, IPI00902670 | Hepatocyte growth factor-like protein | MST1 |
| NPDGSERPWCYTTDPQIER | IPI00922041, IPI00847702, IPI00873854, IPI00925679, IPI00922577, IPI00292218, IPI00384647, IPI00925822, IPI00925898, IPI00718805, IPI00884434, IPI00925540, IPI00908726, IPI00902670 | Hepatocyte growth factor-like protein | MST1 |
| QEATTVSCFR | IPI00922041, IPI00847702, IPI00873854, IPI00925679, IPI00922577, IPI00292218, IPI00384647, IPI00925822, IPI00925898, IPI00718805, IPI00884434, IPI00925540, IPI00908726, IPI00902670 | Hepatocyte growth factor-like protein | MST1 |
| QGQHFCGGSLVK | IPI00922041, IPI00847702, IPI00873854, IPI00925679, IPI00922577, IPI00292218, IPI00384647, IPI00925822, IPI00925898, IPI00718805, IPI00884434, IPI00925540, IPI00908726, IPI00902670 | Hepatocyte growth factor-like protein | MST1 |
| SPLNDFQVLR | IPI00922041, IPI00847702, IPI00873854, IPI00925679, IPI00922577, IPI00292218, IPI00384647, IPI00925822, IPI00925898, IPI00718805, IPI00884434, IPI00925540, IPI00908726, IPI00902670 | Hepatocyte growth factor-like protein | MST1 |
| TCIMNNGVGYR | IPI00922041, IPI00847702, IPI00873854, IPI00925679, IPI00922577, IPI00292218, IPI00384647, IPI00925822, IPI00925898, IPI00718805, IPI00884434, IPI00925540, IPI00908726, IPI00902670 | Hepatocyte growth factor-like protein | MST1 |
| TPFDYCALR | IPI00922041, IPI00847702, IPI00873854, IPI00925679, IPI00922577, IPI00292218, IPI00384647, IPI00925822, IPI00925898, IPI00718805, IPI00884434, IPI00925540, IPI00908726, IPI00902670 | Hepatocyte growth factor-like protein | MST1 |
| VALICLPPEWYVVPPGTK | IPI00922041, IPI00847702, IPI00873854, IPI00925679, IPI00922577, IPI00292218, IPI00384647, IPI00925822, IPI00925898, IPI00718805, IPI00884434, IPI00925540, IPI00908726, IPI00902670 | Hepatocyte growth factor-like protein | MST1 |
| VSVFVDWIHK | IPI00922041, IPI00847702, IPI00873854, IPI00925679, IPI00922577, IPI00292218, IPI00384647, IPI00925822, IPI00925898, IPI00718805, IPI00884434, IPI00925540, IPI00908726, IPI00902670 | Hepatocyte growth factor-like protein | MST1 |
| VVGGHPGNSPWTVSLR | IPI00922041, IPI00847702, IPI00873854, IPI00925679, IPI00922577, IPI00292218, IPI00384647, IPI00925822, IPI00925898, IPI00718805, IPI00884434, IPI00925540, IPI00908726, IPI00902670 | Hepatocyte growth factor-like protein | MST1 |
| WSAETPHKPQFTFTSEPHAQLEENFCR | IPI00922041, IPI00847702, IPI00873854, IPI00925679, IPI00922577, IPI00292218, IPI00384647, IPI00925822, IPI00925898, IPI00718805, IPI00884434, IPI00925540, IPI00908726, IPI00902670 | Hepatocyte growth factor-like protein | MST1 |
| AAPAPAPPPEPERPK | IPI00926541, IPI00243742 | Myosin light chain 3 | MYL3 |
| QVFEYDLISQFMQGYPSK | IPI00844385, IPI00019190 | Myocilin precursor | MYOC |
| AAAVSEAEADFYEQNSR | IPI00746033, IPI00555656, IPI00008787 | Alpha-N-acetylglucosaminidase precursor | NAGLU |
| AGGVLAYELLPALDEVLASDSR | IPI00746033, IPI00555656, IPI00008787 | Alpha-N-acetylglucosaminidase precursor | NAGLU |
| LLGPGPAADFSVSVER | IPI00008787 | Alpha-N-acetylglucosaminidase precursor | NAGLU |
| LLVLDLFAESQPVYTR | IPI00746033, IPI00555656, IPI00008787 | Alpha-N-acetylglucosaminidase precursor | NAGLU |
| SFGMTPVLPAFAGHVPEAVTR | IPI00008787 | Alpha-N-acetylglucosaminidase precursor | NAGLU |
| CVVTGEDGSESEATVNVK | IPI00913951, IPI00902968, IPI00900302, IPI00795918, IPI00435020, IPI00385035, IPI00900340, IPI00908926, IPI00220737, IPI00411478 | Isoform 3 of Neural cell adhesion molecule 1 | NCAM1 |
| DIQVIVNVPPTIQAR | IPI00913951, IPI00902968, IPI00900302, IPI00795918, IPI00435020, IPI00385035, IPI00900340, IPI00908926, IPI00220737, IPI00411478 | Isoform 3 of Neural cell adhesion molecule 1 | NCAM1 |
| EASMEGIVTIVGLKPETTYAVR | IPI00913951, IPI00902968, IPI00900302, IPI00795918, IPI00435020, IPI00385035, IPI00900340, IPI00908926, IPI00220737, IPI00411478 | Isoform 3 of Neural cell adhesion molecule 1 | NCAM1 |
| GLGEISAASEFK | IPI00913951, IPI00902968, IPI00900302, IPI00795918, IPI00435020, IPI00385035, IPI00900340, IPI00908926, IPI00220737, IPI00411478 | Isoform 3 of Neural cell adhesion molecule 1 | NCAM1 |
| TQPVQGEPSAPK | IPI00913951, IPI00902968, IPI00900302, IPI00795918, IPI00435020, IPI00385035, IPI00900340, IPI00908926, IPI00220737, IPI00411478 | Isoform 3 of Neural cell adhesion molecule 1 | NCAM1 |
| DQAGEYECSAENDVSFPDVR | IPI00176221, IPI00645710 | Neuronal growth regulator 1 precursor | NEGR1 |
| LIVAGLPR | IPI00556554, IPI00472011, IPI00217291, IPI00023814 | Isoform 1 of Neogenin precursor | NEO1 |
| TFTPFYFLVEPVDTLSVR | IPI00556554, IPI00472011, IPI00217291, IPI00023814 | Isoform 1 of Neogenin precursor | NEO1 |
| GNLYWTDWNR | IPI00384542, IPI00028908, IPI00026944, IPI00293033 | Isoform 1 of Nidogen-1 precursor | NID1 |
| VLFETDLVNPR | IPI00384542, IPI00028908, IPI00026944, IPI00293033 | Isoform 1 of Nidogen-1 precursor | NID1 |
| CESAPGQLPVCSCPQGWQGPR | IPI00029819 | Neurogenic locus notch homolog protein 3 precursor | NOTCH3 |
| GESCSDLEPCDESSGLYCDR | IPI00011140 | Protein NOV homolog precursor | NOV |
| AVVHGILMGVPVPFPIPEPDGCK | IPI00940960, IPI00301579 | Epididymal secretory protein E1 | NPC2 |
| EVNVSPCPTQPCQLSK | IPI00940960, IPI00301579 | Epididymal secretory protein E1 | NPC2 |
| SGINCPIQK | IPI00940960, IPI00301579 | Epididymal secretory protein E1 | NPC2 |
| SLTEILLER | IPI00014594 | Isoform 1 of Podocin | NPHS2 |
| CEWLIQAPDPYQR | IPI00299594, IPI00939515, IPI00607733, IPI00646134, IPI00165438, IPI00643127, IPI00749187, IPI00639917 | Muscle type neuropilin 1 | NRP1 |
| EGNKPVLFQGNTNPTDVVVAVFPKPLITR | IPI00299594, IPI00939515, IPI00607733, IPI00646134, IPI00165438, IPI00643127, IPI00749187, IPI00639917 | Muscle type neuropilin 1 | NRP1 |
| EWIQVDLGLLR | IPI00299594, IPI00939515, IPI00607733, IPI00646134, IPI00165438, IPI00643127, IPI00749187, IPI00639917 | Muscle type neuropilin 1 | NRP1 |
| FVSDYETHGAGFSIR | IPI00299594, IPI00939515, IPI00607733, IPI00646134, IPI00165438, IPI00643127, IPI00749187, IPI00639917 | Muscle type neuropilin 1 | NRP1 |
| IDVSSNGEDWITIK | IPI00299594, IPI00939515, IPI00607733, IPI00646134, IPI00165438, IPI00643127, IPI00749187, IPI00639917 | Muscle type neuropilin 1 | NRP1 |
| IMINFNPHFDLEDR | IPI00299594, IPI00939515, IPI00607733, IPI00646134, IPI00165438, IPI00643127, IPI00749187, IPI00639917 | Muscle type neuropilin 1 | NRP1 |
| IMINFNPHFDLEDRDCK | IPI00299594, IPI00939515, IPI00607733, IPI00646134, IPI00165438, IPI00643127, IPI00749187, IPI00639917 | Muscle type neuropilin 1 | NRP1 |
| LEIWDGFPDVGPHIGR | IPI00299594, IPI00939515, IPI00607733, IPI00646134, IPI00165438, IPI00643127, IPI00749187, IPI00639917 | Muscle type neuropilin 1 | NRP1 |
| LNYPENGWTPGEDSYR | IPI00299594, IPI00939515, IPI00607733, IPI00646134, IPI00165438, IPI00643127, IPI00749187, IPI00639917 | Muscle type neuropilin 1 | NRP1 |
| MSEIILEFESFDLEPDSNPPGGMFCR | IPI00299594, IPI00939515, IPI00607733, IPI00646134, IPI00165438, IPI00643127, IPI00749187, IPI00639917 | Muscle type neuropilin 1 | NRP1 |
| NWMPENIR | IPI00299594, IPI00939515, IPI00607733, IPI00646134, IPI00165438, IPI00643127, IPI00749187, IPI00639917 | Muscle type neuropilin 1 | NRP1 |
| SFEGNNNYDTPELR | IPI00299594, IPI00939515, IPI00607733, IPI00646134, IPI00165438, IPI00643127, IPI00749187, IPI00639917 | Muscle type neuropilin 1 | NRP1 |
| VDIPEIHER | IPI00029693, IPI00218781 | Isoform A22 of Neuropilin-2 | NRP2 |
| DLELLIQTATR | IPI00893190, IPI00892657, IPI00892782, IPI00892540, IPI00295542 | Nucleobindin-1 | NUCB1 |
| LVTLEEFLASTQR | IPI00893190, IPI00892657, IPI00892782, IPI00892540, IPI00295542 | Nucleobindin-1 | NUCB1 |
| ITTLAFQVHDGLGR | IPI00374294, IPI00924444 | Isoform 1 of Lysophospholipid acyltransferase 1 | OACT1 |
| LPDGQVTEESLQADSDADSISLELR | IPI00328703 | Out at first protein homolog precursor | OAF |
| LSLLEELSLAENQLLK | IPI00025465, IPI00515092 | Osteoglycin | OGN |
| LLIPVTCEFPR | IPI00328215 | Isoform 1 of Oncoprotein-induced transcript 3 protein precursor | OIT3 |
| LYTISEGYVPNLR | IPI00328215 | Isoform 1 of Oncoprotein-induced transcript 3 protein precursor | OIT3 |
| CICTVVAPQQTMCSR | IPI00017841, IPI00419820, IPI00472517, IPI00419822, IPI00550145 | Isoform 1 of Noelin | OLFM1 |
| FGSWMTDPLAPEGDNR | IPI00017841, IPI00419820, IPI00472517, IPI00419822, IPI00550145 | Isoform 1 of Noelin | OLFM1 |
| LTGISDPVTVK | IPI00017841, IPI00419820, IPI00472517, IPI00419822, IPI00550145 | Isoform 1 of Noelin | OLFM1 |
| MDELRPLIPVLEEYK | IPI00017841, IPI00419820, IPI00472517, IPI00419822, IPI00550145 | Isoform 1 of Noelin | OLFM1 |
| VWYMDGYHNNR | IPI00017841, IPI00419820, IPI00472517, IPI00419822, IPI00550145 | Isoform 1 of Noelin | OLFM1 |
| DKCEPLEK | IPI00022429, IPI00884926, IPI00020091 | Alpha-1-acid glycoprotein 2 | ORM2 |
| EQLGEFYEALDCLR | IPI00022429, IPI00884926, IPI00020091 | Alpha-1-acid glycoprotein 2 | ORM2 |
| EQLGEFYEALDCLRIPK | IPI00022429, IPI00884926, IPI00020091 | Alpha-1-acid glycoprotein 2 | ORM2 |
| KDKCEPLEK | IPI00022429, IPI00884926, IPI00020091 | Alpha-1-acid glycoprotein 2 | ORM2 |
| NWGLSVYADKPETTK | IPI00022429, IPI00884926, IPI00020091 | Alpha-1-acid glycoprotein 2 | ORM2 |
| NWGLSVYADKPETTKEQLGEFYEALDCLR | IPI00022429, IPI00884926, IPI00020091 | Alpha-1-acid glycoprotein 2 | ORM2 |
| SDVVYTDWK | IPI00022429, IPI00884926, IPI00020091 | Alpha-1-acid glycoprotein 2 | ORM2 |
| TEDTIFLR | IPI00022429, IPI00884926, IPI00020091 | Alpha-1-acid glycoprotein 2 | ORM2 |
| TYMLAFDVNDEK | IPI00022429, IPI00884926, IPI00020091 | Alpha-1-acid glycoprotein 2 | ORM2 |
| WFYIASAFR | IPI00022429, IPI00884926, IPI00020091 | Alpha-1-acid glycoprotein 2 | ORM2 |
| YVGGQEHFAHLLILR | IPI00022429, IPI00884926, IPI00020091 | Alpha-1-acid glycoprotein 2 | ORM2 |
| EHVAHLLFLR | IPI00020091 | Alpha-1-acid glycoprotein 2 precursor | ORM2 |
| EQLGEFYEALDCLCIPR | IPI00020091 | Alpha-1-acid glycoprotein 2 precursor | ORM2 |
| NWGLSFYADKPETTK | IPI00020091 | Alpha-1-acid glycoprotein 2 precursor | ORM2 |
| SDVMYTDWK | IPI00020091 | Alpha-1-acid glycoprotein 2 precursor | ORM2 |
| TLMFGSYLDDEK | IPI00020091 | Alpha-1-acid glycoprotein 2 precursor | ORM2 |
| TLMFGSYLDDEKNWGLSFYADKPETTK | IPI00020091 | Alpha-1-acid glycoprotein 2 precursor | ORM2 |
| FGLFKPGEIAPLLFR | IPI00107731, IPI00178869, IPI00292153, IPI00409588, IPI00795786, IPI00872241, IPI00647357, IPI00657951, IPI00921018, IPI00795142 | Isoform 6 of Osteoclast-associated immunoglobulin-like receptor | OSCAR |
| HSAQPWADFTLLGAR | IPI00107731, IPI00178869, IPI00292153, IPI00409588, IPI00795786, IPI00872241, IPI00647357, IPI00657951, IPI00921018, IPI00795142 | Isoform 6 of Osteoclast-associated immunoglobulin-like receptor | OSCAR |
| ILEFFGLK | IPI00879437, IPI00878551, IPI00010796 | Protein disulfide-isomerase | P4HB |
| LITLEEEMTK | IPI00879437, IPI00878551, IPI00010796 | Protein disulfide-isomerase | P4HB |
| NFEDVAFDEK | IPI00879437, IPI00878551, IPI00010796 | Protein disulfide-isomerase | P4HB |
| IPVDEEAFVIDFKPR | IPI00940221, IPI00219042, IPI00177543, IPI00219043, IPI00878690 | peptidylglycine alpha-amidating monooxygenase isoform a preproprotein | PAM |
| QSPQLPQAFYPVGHPVDVSFGDLLAAR | IPI00940221, IPI00219042, IPI00177543, IPI00219043, IPI00878690 | peptidylglycine alpha-amidating monooxygenase isoform a preproprotein | PAM |
| SLIPIEISESAAVGTR | IPI00914534, IPI00030618 | Isoform 1 of Protocadherin-18 | PCDH18 |
| ECIWTITVPEGQTVSLSFR | IPI00908730, IPI00299738 | Procollagen C-endopeptidase enhancer 1 | PCOLCE |
| FCGTFRPAPLVAPGNQVTLR | IPI00908730, IPI00299738 | Procollagen C-endopeptidase enhancer 1 | PCOLCE |
| GESGYVASEGFPNLYPPNK | IPI00908730, IPI00299738 | Procollagen C-endopeptidase enhancer 1 | PCOLCE |
| GFLLWYSGR | IPI00908730, IPI00299738 | Procollagen C-endopeptidase enhancer 1 | PCOLCE |
| VFDLELHPACR | IPI00908730, IPI00299738 | Procollagen C-endopeptidase enhancer 1 | PCOLCE |
| YDALEVFAGSGTSGQR | IPI00908730, IPI00299738 | Procollagen C-endopeptidase enhancer 1 | PCOLCE |
| EPGEGLAVTVSLIGAYK | IPI00299738 | Procollagen C-endopeptidase enhancer 1 precursor | PCOLCE |
| FDLEPDTYCR | IPI00299738 | Procollagen C-endopeptidase enhancer 1 precursor | PCOLCE |
| GPVLPPESFVVLHRPNQDQILTNLSK | IPI00299738 | Procollagen C-endopeptidase enhancer 1 precursor | PCOLCE |
| GVSYLLMGQVEENR | IPI00299738 | Procollagen C-endopeptidase enhancer 1 precursor | PCOLCE |
| SQPPEKTEESPSAPDAPTCPK | IPI00299738 | Procollagen C-endopeptidase enhancer 1 precursor | PCOLCE |
| TEESPSAPDAPTCPK | IPI00299738 | Procollagen C-endopeptidase enhancer 1 precursor | PCOLCE |
| YDSVSVFNGAVSDDSR | IPI00299738 | Procollagen C-endopeptidase enhancer 1 precursor | PCOLCE |
| CAPDEELLSCSSFSR | IPI00387168 | Isoform 1 of Proprotein convertase subtilisin/kexin type 9 precursor | PCSK9 |
| DVINEAWFPEDQR | IPI00387168 | Isoform 1 of Proprotein convertase subtilisin/kexin type 9 precursor | PCSK9 |
| AGVVLVTAAGNFR | IPI00387168, IPI00909849, IPI00909087, IPI00387169 | Proprotein convertase subtilisin/kexin type 9 | PCSK9 |
| ILHVFHGLLPGFLVK | IPI00387168, IPI00909849, IPI00909087, IPI00387169 | Proprotein convertase subtilisin/kexin type 9 | PCSK9 |
| KSQLVQPVGPLVVLLPLAGGYSR | IPI00387168, IPI00909849, IPI00909087, IPI00387169 | Proprotein convertase subtilisin/kexin type 9 | PCSK9 |
| MSGDLLELALK | IPI00387168, IPI00909849, IPI00909087, IPI00387169 | Proprotein convertase subtilisin/kexin type 9 | PCSK9 |
| VMVTDFENVPEEDGTR | IPI00387168, IPI00909849, IPI00909087, IPI00387169 | Proprotein convertase subtilisin/kexin type 9 | PCSK9 |
| ELSDFISYLQR | IPI00847663, IPI00025252, IPI00791418 | Protein disulfide-isomerase A3 | PDIA3 |
| FLQDYFDGNLK | IPI00847663, IPI00025252, IPI00791418 | Protein disulfide-isomerase A3 | PDIA3 |
| TFSHELSDFGLESTAGEIPVVAIR | IPI00025252 | Protein disulfide-isomerase A3 precursor | PDIA3 |
| FHLGEPEASTQFMTQNYQDSPTLQAPR | IPI00163563 | PEBP family protein precursor | PEBP4 |
| ITSWMEPIVK | IPI00163563 | PEBP family protein precursor | PEBP4 |
| YQFFVYLQEGK | IPI00163563 | PEBP family protein precursor | PEBP4 |
| KNPAVQAGSIVVLQGGEETQR | IPI00257882, IPI00909399, IPI00910514 | Xaa-Pro dipeptidase | PEPD |
| TVEEIEACMAGCDK | IPI00257882, IPI00909399, IPI00910514 | Xaa-Pro dipeptidase | PEPD |
| VPLALFALNR | IPI00257882, IPI00909399, IPI00910514 | Xaa-Pro dipeptidase | PEPD |
| CEELQGQK | IPI00021364 | Properdin precursor | PFC |
| CSAPEPSQKPPGKPCPGLAYEQR | IPI00021364 | Properdin precursor | PFC |
| CVGWNGQCSGK | IPI00021364 | Properdin precursor | PFC |
| GLLGGGVSVEDCCLNTAFAYQK | IPI00021364 | Properdin precursor | PFC |
| RPCLHVPACKDPEEEEL | IPI00021364 | Properdin precursor | PFC |
| SISCQEIPGQQSR | IPI00021364 | Properdin precursor | PFC |
| TCNHPVPQHGGPFCAGDATR | IPI00021364 | Properdin precursor | PFC |
| DSLLQDGEFSMDLR | IPI00216691 | Profilin-1 | PFN1 |
| SSFYVNGLTLGGQK | IPI00216691 | Profilin-1 | PFN1 |
| TLVLLMGK | IPI00216691 | Profilin-1 | PFN1 |
| LALLVDTVGPR | IPI00005794, IPI00796411, IPI00007664 | Plasma glutamate carboxypeptidase | PGCP |
| VGALASLIR | IPI00005794, IPI00796411, IPI00007664 | Plasma glutamate carboxypeptidase | PGCP |
| AGLLRPDYALLGHR | IPI00394992, IPI00163207 | Isoform 1 of N-acetylmuramoyl-L-alanine amidase precursor | PGLYRP2 |
| ASLLTMAFLNGALDGVILGDYLSR | IPI00394992, IPI00163207 | Isoform 1 of N-acetylmuramoyl-L-alanine amidase precursor | PGLYRP2 |
| DGSPDVTTADIGANTPDATK | IPI00394992, IPI00163207 | Isoform 1 of N-acetylmuramoyl-L-alanine amidase precursor | PGLYRP2 |
| DTLPSCAVR | IPI00394992, IPI00163207 | Isoform 1 of N-acetylmuramoyl-L-alanine amidase precursor | PGLYRP2 |
| EFTEAFLGCPAIHPR | IPI00394992, IPI00163207 | Isoform 1 of N-acetylmuramoyl-L-alanine amidase precursor | PGLYRP2 |
| EGKEYGVVLAPDGSTVAVEPLLAGLEAGLQGR | IPI00394992, IPI00163207 | Isoform 1 of N-acetylmuramoyl-L-alanine amidase precursor | PGLYRP2 |
| EYGVVLAPDGSTVAVEPLLAGLEAGLQGR | IPI00394992, IPI00163207 | Isoform 1 of N-acetylmuramoyl-L-alanine amidase precursor | PGLYRP2 |
| GCPDVQASLPDAK | IPI00394992, IPI00163207 | Isoform 1 of N-acetylmuramoyl-L-alanine amidase precursor | PGLYRP2 |
| GSQTQSHPDLGTEGCWDQLSAPR | IPI00394992, IPI00163207 | Isoform 1 of N-acetylmuramoyl-L-alanine amidase precursor | PGLYRP2 |
| HTASAWLMSAPNSGPHNR | IPI00394992, IPI00163207 | Isoform 1 of N-acetylmuramoyl-L-alanine amidase precursor | PGLYRP2 |
| LLQLPLGFLYVHHTYVPAPPCTDFTR | IPI00394992, IPI00163207 | Isoform 1 of N-acetylmuramoyl-L-alanine amidase precursor | PGLYRP2 |
| RVINLPLDSMAAPWETGDTFPDVVAIAPDVR | IPI00394992, IPI00163207 | Isoform 1 of N-acetylmuramoyl-L-alanine amidase precursor | PGLYRP2 |
| TDCPGDALFDLLR | IPI00394992, IPI00163207 | Isoform 1 of N-acetylmuramoyl-L-alanine amidase precursor | PGLYRP2 |
| TFTLLDPK | IPI00394992, IPI00163207 | Isoform 1 of N-acetylmuramoyl-L-alanine amidase precursor | PGLYRP2 |
| YHQDTQGWGDIGYSFVVGSDGYVYEGR | IPI00394992, IPI00163207 | Isoform 1 of N-acetylmuramoyl-L-alanine amidase precursor | PGLYRP2 |
| GENLFAITDEGMDVPLAMEEWHHER | IPI00301143, IPI00845506 | Isoform 1 of Peptidase inhibitor 16 precursor | PI16 |
| LMVELHNLYR | IPI00301143, IPI00845506 | Isoform 1 of Peptidase inhibitor 16 precursor | PI16 |
| WDEELAAFAK | IPI00301143, IPI00845506 | Isoform 1 of Peptidase inhibitor 16 precursor | PI16 |
| EAEAAIYHLQLFEELR | IPI00847989, IPI00910979, IPI00479186 | Pyruvate kinase | PKM2 |
| YWPVLDNALR | IPI00060310 | Phospholipase D4 | PLD4 |
| EDPAYLHYYDPAGAEDPLGAIHLR | IPI00306311 | Pleckstrin | PLEK |
| QQDHFFQAAFLEER | IPI00306311, IPI00742980 | Pleckstrin | PLEK |
| FVTWIEGVMR | IPI00019580, IPI00937938 | Plasminogen | PLG |
| ATTVTGTPCQDWAAQEPHR | IPI00019580 | Plasminogen precursor | PLG |
| LFLEPTR | IPI00019580 | Plasminogen precursor | PLG |
| NPDGDVGGPWCYTTNPR | IPI00019580 | Plasminogen precursor | PLG |
| TPENYPNAGLTMNYCR | IPI00019580 | Plasminogen precursor | PLG |
| VILGAHQEVNLEPHVQEIEVSR | IPI00019580 | Plasminogen precursor | PLG |
| VIPACLPSPNYVVADR | IPI00019580 | Plasminogen precursor | PLG |
| WELCDIPR | IPI00019580 | Plasminogen precursor | PLG |
| WEYCNLK | IPI00019580 | Plasminogen precursor | PLG |
| FLEQELETITIPDLR | IPI00217778, IPI00909702, IPI00643034, IPI00022733 | 45 kDa protein | PLTP |
| GAFFPLTER | IPI00217778, IPI00909702, IPI00643034, IPI00022733 | 45 kDa protein | PLTP |
| TMLQIGVMPMLNER | IPI00217778, IPI00909702, IPI00643034, IPI00022733 | 45 kDa protein | PLTP |
| VPHDLDMLLR | IPI00217778, IPI00909702, IPI00643034, IPI00022733 | 45 kDa protein | PLTP |
| VYDFLSTFITSGMR | IPI00217778, IPI00909702, IPI00643034, IPI00022733 | 45 kDa protein | PLTP |
| QDWVDSGCPEESK | IPI00073777, IPI00640599, IPI00044369 | Isoform 1 of Plexin domain-containing protein 2 precursor | PLXDC2 |
| VGLSDAFVVVHR | IPI00073777, IPI00640599, IPI00044369 | Isoform 1 of Plexin domain-containing protein 2 precursor | PLXDC2 |
| SFVASNDEGVATVGLVSSTGPGGDR | IPI00853369, IPI00816626 | PLXNB2 protein | PLXNB2 |
| EVQPVELPNCNLVK | IPI00916980, IPI00218732 | Serum paraoxonase/arylesterase 1 | PON1 |
| FDVSSFNPHGISTFTDEDNAMYLLVVNHPDAK | IPI00218732 | Serum paraoxonase/arylesterase 1 | PON1 |
| IFFYDSENPPASEVLR | IPI00218732 | Serum paraoxonase/arylesterase 1 | PON1 |
| IHVYEK | IPI00218732 | Serum paraoxonase/arylesterase 1 | PON1 |
| ILLMDLNEEDPTVLELGITGSK | IPI00218732 | Serum paraoxonase/arylesterase 1 | PON1 |
| IQNILTEEPK | IPI00218732 | Serum paraoxonase/arylesterase 1 | PON1 |
| LLIGTVFHK | IPI00218732 | Serum paraoxonase/arylesterase 1 | PON1 |
| SFNPNSPGK | IPI00218732 | Serum paraoxonase/arylesterase 1 | PON1 |
| SLDFNTLVDNISVDPETGDLWVGCHPNGMK | IPI00218732 | Serum paraoxonase/arylesterase 1 | PON1 |
| STVELFK | IPI00218732 | Serum paraoxonase/arylesterase 1 | PON1 |
| VVAEGFDFANGINISPDGK | IPI00218732 | Serum paraoxonase/arylesterase 1 | PON1 |
| YVYIAELLAHK | IPI00218732 | Serum paraoxonase/arylesterase 1 | PON1 |
| AAAITSDILEALGR | IPI00007960, IPI00641231, IPI00910262, IPI00410241, IPI00218585 | Isoform 1 of Periostin | POSTN |
| GCPAVLPIDHVYGTLGIVGATTTQR | IPI00007960, IPI00641231, IPI00910262, IPI00410241, IPI00218585 | Isoform 1 of Periostin | POSTN |
| EESLDSDLYAELR | IPI00022445 | Platelet basic protein precursor | PPBP |
| GKEESLDSDLYAELR | IPI00022445 | Platelet basic protein precursor | PPBP |
| GTHCNQVEVIATLK | IPI00022445 | Platelet basic protein precursor | PPBP |
| ICLDPDAPR | IPI00022445 | Platelet basic protein precursor | PPBP |
| KICLDPDAPR | IPI00022445 | Platelet basic protein precursor | PPBP |
| NIQSLEVIGK | IPI00022445 | Platelet basic protein precursor | PPBP |
| IIPGFMCQGGDFTR | IPI00741973, IPI00419585, IPI00030144, IPI00455871, IPI00886744, IPI00743690, IPI00935462, IPI00888100, IPI00376170, IPI00925411, IPI00925747, IPI00910407, IPI00787316 | Peptidylprolyl cis-trans isomerase A-like 4B | PPIA |
| VSFELFADK | IPI00741973, IPI00419585, IPI00030144, IPI00455871, IPI00886744, IPI00743690, IPI00935462, IPI00888100, IPI00376170, IPI00925411, IPI00925747, IPI00910407, IPI00787316 | Peptidylprolyl cis-trans isomerase A-like 4B | PPIA |
| VYFDLR | IPI00646304 | peptidylprolyl isomerase B precursor | PPIB |
| LVALLNTLDR | IPI00910771, IPI00853207, IPI00893197, IPI00935879, IPI00941236, IPI00640101, IPI00293102, IPI00853351, IPI00880122, IPI00217296 | Protein phosphatase 2A activator, regulatory subunit 4 | PPP2R4 |
| LWVMPNHQVLLGPEEDQDHIYHPQ | IPI00939511, IPI00855875, IPI00465255, IPI00855900, IPI00922335 | Isoform 1 of Proline-rich acidic protein 1 | PRAP1 |
| SQGPPPPGKPQGPPPQGGSK | IPI00552432 | Basic salivary proline-rich protein 2 | PRB2 |
| AMLVFAEHR | IPI00001593, IPI00399307 | Lysosomal Pro-X carboxypeptidase precursor | PRCP |
| DITDTLVAVTISEGAHHLDLR | IPI00001593, IPI00399307 | Lysosomal Pro-X carboxypeptidase precursor | PRCP |
| HLNFLTSEQALADFAELIK | IPI00001593, IPI00399307 | Lysosomal Pro-X carboxypeptidase precursor | PRCP |
| NALDPMSVLLAR | IPI00001593, IPI00399307 | Lysosomal Pro-X carboxypeptidase precursor | PRCP |
| EGGLGPLNIPLLADVTR | IPI00909207, IPI00000874, IPI00794777, IPI00027350, IPI00641244, IPI00640741 | Peroxiredoxin-1 | PRDX1 |
| GLFIIDGK | IPI00909207, IPI00000874, IPI00794777, IPI00027350, IPI00641244, IPI00640741 | Peroxiredoxin-1 | PRDX1 |
| KEGGLGPLNIPLLADVTR | IPI00909207, IPI00000874, IPI00794777, IPI00027350, IPI00641244, IPI00640741 | Peroxiredoxin-1 | PRDX1 |
| QITVNDLPVGR | IPI00909207, IPI00000874, IPI00794777, IPI00027350, IPI00641244, IPI00640741 | Peroxiredoxin-1 | PRDX1 |
| LPFPIIDDR | IPI00220301 | Peroxiredoxin-6 | PRDX6 |
| SLQTFSQAWFTCR | IPI00847535, IPI00010341 | Bone marrow proteoglycan precursor | PRG2 |
| TLPEDEETPEQEMEETPCR | IPI00847535, IPI00010341 | Bone marrow proteoglycan precursor | PRG2 |
| DATCNCDYNCQHYMECCPDFK | IPI00655927, IPI00656111, IPI00655676, IPI00024825, IPI00655976, IPI00656092 | Isoform A of Proteoglycan-4 precursor | PRG4 |
| GLPNVVTSAISLPNIR | IPI00655927, IPI00656111, IPI00655676, IPI00024825, IPI00655976, IPI00656092 | Isoform A of Proteoglycan-4 precursor | PRG4 |
| ITEVWGIPSPIDTVFTR | IPI00655927, IPI00656111, IPI00655676, IPI00024825, IPI00655976, IPI00656092 | Isoform A of Proteoglycan-4 precursor | PRG4 |
| ANSFLEELR | IPI00917472, IPI00902485, IPI00877104, IPI00916520, IPI00916591, IPI00911093, IPI00916433, IPI00940493, IPI00816176, IPI00021817, IPI00908685 | Vitamin K-dependent protein C | PROC |
| CSCAPGYK | IPI00917472, IPI00902485, IPI00877104, IPI00916520, IPI00916591, IPI00911093, IPI00916433, IPI00940493, IPI00816176, IPI00021817, IPI00908685 | Vitamin K-dependent protein C | PROC |
| FPCGRPWK | IPI00917472, IPI00902485, IPI00877104, IPI00916520, IPI00916591, IPI00911093, IPI00916433, IPI00940493, IPI00816176, IPI00021817, IPI00908685 | Vitamin K-dependent protein C | PROC |
| IPVVPHNECSEVMSNMVSENMLCAGILGDR | IPI00917472, IPI00902485, IPI00877104, IPI00916520, IPI00916591, IPI00911093, IPI00916433, IPI00940493, IPI00816176, IPI00021817, IPI00908685 | Vitamin K-dependent protein C | PROC |
| LGDDLLQCHPAVK | IPI00917472, IPI00902485, IPI00877104, IPI00916520, IPI00916591, IPI00911093, IPI00916433, IPI00940493, IPI00816176, IPI00021817, IPI00908685 | Vitamin K-dependent protein C | PROC |
| RGDSPWQVVLLDSK | IPI00917472, IPI00902485, IPI00877104, IPI00916520, IPI00916591, IPI00911093, IPI00916433, IPI00940493, IPI00816176, IPI00021817, IPI00908685 | Vitamin K-dependent protein C | PROC |
| STTDNDIALLHLAQPATLSQTIVPICLPDSGLAER | IPI00917472, IPI00902485, IPI00877104, IPI00916520, IPI00916591, IPI00911093, IPI00916433, IPI00940493, IPI00816176, IPI00021817, IPI00908685 | Vitamin K-dependent protein C | PROC |
| TFVLNFIK | IPI00917472, IPI00902485, IPI00877104, IPI00916520, IPI00916591, IPI00911093, IPI00916433, IPI00940493, IPI00816176, IPI00021817, IPI00908685 | Vitamin K-dependent protein C | PROC |
| WELDLDIK | IPI00917472, IPI00902485, IPI00877104, IPI00916520, IPI00916591, IPI00911093, IPI00916433, IPI00940493, IPI00816176, IPI00021817, IPI00908685 | Vitamin K-dependent protein C | PROC |
| CFLGCELPPEGSR | IPI00009276 | Endothelial protein C receptor precursor | PROCR |
| EFLEDTCVQYVQK | IPI00009276 | Endothelial protein C receptor precursor | PROCR |
| LHMLQISYFR | IPI00009276 | Endothelial protein C receptor precursor | PROCR |
| TLAFPLTIR | IPI00009276 | Endothelial protein C receptor precursor | PROCR |
| TQSGLQSYLLQFHGLVR | IPI00009276 | Endothelial protein C receptor precursor | PROCR |
| CEFDINECK | IPI00879470, IPI00795619, IPI00930047, IPI00879815, IPI00294004, IPI00794924, IPI00878131 | Vitamin K-dependent protein S | PROS1 |
| DVDECSLKPSICGTAVCK | IPI00879470, IPI00795619, IPI00930047, IPI00879815, IPI00294004, IPI00794924, IPI00878131 | Vitamin K-dependent protein S | PROS1 |
| EAVMDINKPGPLFKPENGLLETK | IPI00879470, IPI00795619, IPI00930047, IPI00879815, IPI00294004, IPI00794924, IPI00878131 | Vitamin K-dependent protein S | PROS1 |
| FSAEFDFR | IPI00879470, IPI00795619, IPI00930047, IPI00879815, IPI00294004, IPI00794924, IPI00878131 | Vitamin K-dependent protein S | PROS1 |
| KVESELIKPINPR | IPI00879470, IPI00795619, IPI00930047, IPI00879815, IPI00294004, IPI00794924, IPI00878131 | Vitamin K-dependent protein S | PROS1 |
| NGFVMLSNK | IPI00879470, IPI00795619, IPI00930047, IPI00879815, IPI00294004, IPI00794924, IPI00878131 | Vitamin K-dependent protein S | PROS1 |
| NIPGDFECECPEGYR | IPI00879470, IPI00795619, IPI00930047, IPI00879815, IPI00294004, IPI00794924, IPI00878131 | Vitamin K-dependent protein S | PROS1 |
| NNLELSTPLKIETISHEDLQR | IPI00879470, IPI00795619, IPI00930047, IPI00879815, IPI00294004, IPI00794924, IPI00878131 | Vitamin K-dependent protein S | PROS1 |
| QSTNAYPDLR | IPI00879470, IPI00795619, IPI00930047, IPI00879815, IPI00294004, IPI00794924, IPI00878131 | Vitamin K-dependent protein S | PROS1 |
| SCVNAIPDQCSPLPCNEDGYMSCK | IPI00879470, IPI00795619, IPI00930047, IPI00879815, IPI00294004, IPI00794924, IPI00878131 | Vitamin K-dependent protein S | PROS1 |
| SFQTGLFTAAR | IPI00879470, IPI00795619, IPI00930047, IPI00879815, IPI00294004, IPI00794924, IPI00878131 | Vitamin K-dependent protein S | PROS1 |
| SQDILLSVENTVIYR | IPI00879470, IPI00795619, IPI00930047, IPI00879815, IPI00294004, IPI00794924, IPI00878131 | Vitamin K-dependent protein S | PROS1 |
| VYFAGFPR | IPI00879470, IPI00795619, IPI00930047, IPI00879815, IPI00294004, IPI00794924, IPI00878131 | Vitamin K-dependent protein S | PROS1 |
| APDLQDLPWQVK | IPI00027843, IPI00216065 | Isoform 1 of Vitamin K-dependent protein Z precursor | PROZ |
| DFAEHLLIPR | IPI00027843, IPI00216065 | Isoform 1 of Vitamin K-dependent protein Z precursor | PROZ |
| DFCGGVIIR | IPI00027843, IPI00216065 | Isoform 1 of Vitamin K-dependent protein Z precursor | PROZ |
| GLLSGWAR | IPI00027843, IPI00216065 | Isoform 1 of Vitamin K-dependent protein Z precursor | PROZ |
| GSWFLTGVLGSQPVGGQAHMVLVTK | IPI00027843, IPI00216065 | Isoform 1 of Vitamin K-dependent protein Z precursor | PROZ |
| QCVPHDQCACGVLTSEK | IPI00027843, IPI00216065 | Isoform 1 of Vitamin K-dependent protein Z precursor | PROZ |
| TDGCQHFCLPGQESYTCSCAQGYR | IPI00027843, IPI00216065 | Isoform 1 of Vitamin K-dependent protein Z precursor | PROZ |
| ILTEAEIDAHLVALAERD | IPI00029623 | Proteasome subunit alpha type-6 | PSMA6 |
| FILNLPTFSVR | IPI00028006 | Proteasome subunit beta type-2 | PSMB2 |
| AQGFTEDTIVFLPQTDK | IPI00514285, IPI00013179, IPI00513767 | Prostaglandin-H2 D-isomerase | PTGDS |
| AAGTEGPFQEVDGVATTR | IPI00465186, IPI00641061, IPI00655814, IPI00480183, IPI00853550, IPI00642154, IPI00107831, IPI00384626 | Isoform 1 of Receptor-type tyrosine-protein phosphatase F | PTPRF |
| AHTDVGPGPESSPVLVR | IPI00465186, IPI00641061, IPI00655814, IPI00480183, IPI00853550, IPI00642154, IPI00107831, IPI00384626 | Isoform 1 of Receptor-type tyrosine-protein phosphatase F | PTPRF |
| DFLPVDPATSNGR | IPI00465186, IPI00641061, IPI00655814, IPI00480183, IPI00853550, IPI00642154, IPI00107831, IPI00384626 | Isoform 1 of Receptor-type tyrosine-protein phosphatase F | PTPRF |
| ELPGELLGYR | IPI00465186, IPI00641061, IPI00655814, IPI00480183, IPI00853550, IPI00642154, IPI00107831, IPI00384626 | Isoform 1 of Receptor-type tyrosine-protein phosphatase F | PTPRF |
| SDMGVGVFTPTIEAR | IPI00465186, IPI00641061, IPI00655814, IPI00480183, IPI00853550, IPI00642154, IPI00107831, IPI00384626 | Isoform 1 of Receptor-type tyrosine-protein phosphatase F | PTPRF |
| VPEDQTGLSGGVASFVCQATGEPKPR | IPI00465186, IPI00641061, IPI00655814, IPI00480183, IPI00853550, IPI00642154, IPI00107831, IPI00384626 | Isoform 1 of Receptor-type tyrosine-protein phosphatase F | PTPRF |
| VTFDPTSSYTLEDLKPDTLYR | IPI00465186, IPI00641061, IPI00655814, IPI00480183, IPI00853550, IPI00642154, IPI00107831, IPI00384626 | Isoform 1 of Receptor-type tyrosine-protein phosphatase F | PTPRF |
| DLLPASLGSYYR | IPI00873341, IPI00011651, IPI00796281 | Isoform 1 of Receptor-type tyrosine-protein phosphatase gamma precursor | PTPRG |
| ETFLDPFVLR | IPI00873341, IPI00011651, IPI00796281 | Isoform 1 of Receptor-type tyrosine-protein phosphatase gamma precursor | PTPRG |
| IIGAMAIFFQVSPR | IPI00873341, IPI00011651, IPI00796281 | Isoform 1 of Receptor-type tyrosine-protein phosphatase gamma precursor | PTPRG |
| DTEVLLVGLEPGTR | IPI00847732, IPI00290328, IPI00943343 | Receptor-type tyrosine-protein phosphatase eta precursor | PTPRJ |
| VITEPIPVSDLR | IPI00847732, IPI00290328, IPI00943343 | Receptor-type tyrosine-protein phosphatase eta precursor | PTPRJ |
| DFLPVDPSASNGR | IPI00299590, IPI00332272, IPI00289831, IPI00743517, IPI00293275, IPI00332271, IPI00332273 | Isoform PTPS of Receptor-type tyrosine-protein phosphatase S | PTPRS |
| HNVDDSLLTTVGSLLEDETYTVR | IPI00299590, IPI00332272, IPI00289831, IPI00743517, IPI00293275, IPI00332271, IPI00332273 | Isoform PTPS of Receptor-type tyrosine-protein phosphatase S | PTPRS |
| TDEDVPSAPPR | IPI00299590, IPI00332272, IPI00289831, IPI00743517, IPI00293275, IPI00332271, IPI00332273 | Isoform PTPS of Receptor-type tyrosine-protein phosphatase S | PTPRS |
| TFDPTTSYVVEDLKPNTEYAFR | IPI00299590, IPI00332272, IPI00289831, IPI00743517, IPI00293275, IPI00332271, IPI00332273 | Isoform PTPS of Receptor-type tyrosine-protein phosphatase S | PTPRS |
| WMQGAEDLTPEDDMPVGR | IPI00299590, IPI00332272, IPI00289831, IPI00743517, IPI00293275, IPI00332271, IPI00332273 | Isoform PTPS of Receptor-type tyrosine-protein phosphatase S | PTPRS |
| WQAESGDGR | IPI00023197, IPI00743691, IPI00107472, IPI00843858, IPI00943208, IPI00384563 | Isoform 2 of Receptor-type tyrosine-protein phosphatase U | PTPRU |
| VLAKPQNTAEVQK | IPI00219425, IPI00219427, IPI00219426, IPI00299158 | Isoform Beta of Poliovirus receptor precursor | PVR |
| VQLTGEPVPMAR | IPI00219425, IPI00219427, IPI00219426, IPI00299158 | Isoform Beta of Poliovirus receptor precursor | PVR |
| AFQPFFVELTMPYSVIR | IPI00878729, IPI00789547, IPI00025426, IPI00748437, IPI00478003, IPI00922117, IPI00796830, IPI00884981 | Isoform 1 of Pregnancy zone protein | PZP |
| AGAFCLSEDAGLGISSTASLR | IPI00878729, IPI00789547, IPI00025426, IPI00748437, IPI00478003, IPI00922117, IPI00796830, IPI00884981 | Isoform 1 of Pregnancy zone protein | PZP |
| ALLAYAFSLLGK | IPI00878729, IPI00789547, IPI00025426, IPI00748437, IPI00478003, IPI00922117, IPI00796830, IPI00884981 | Isoform 1 of Pregnancy zone protein | PZP |
| ATVLNYLPK | IPI00878729, IPI00789547, IPI00025426, IPI00748437, IPI00478003, IPI00922117, IPI00796830, IPI00884981 | Isoform 1 of Pregnancy zone protein | PZP |
| AVGYLITGYQR | IPI00878729, IPI00789547, IPI00025426, IPI00748437, IPI00478003, IPI00922117, IPI00796830, IPI00884981 | Isoform 1 of Pregnancy zone protein | PZP |
| DLKPAIVK | IPI00878729, IPI00789547, IPI00025426, IPI00748437, IPI00478003, IPI00922117, IPI00796830, IPI00884981 | Isoform 1 of Pregnancy zone protein | PZP |
| DMYSFLEDMGLK | IPI00878729, IPI00789547, IPI00025426, IPI00748437, IPI00478003, IPI00922117, IPI00796830, IPI00884981 | Isoform 1 of Pregnancy zone protein | PZP |
| FEIENCLANK | IPI00878729, IPI00789547, IPI00025426, IPI00748437, IPI00478003, IPI00922117, IPI00796830, IPI00884981 | Isoform 1 of Pregnancy zone protein | PZP |
| GEESYCICGSER | IPI00878729, IPI00789547, IPI00025426, IPI00748437, IPI00478003, IPI00922117, IPI00796830, IPI00884981 | Isoform 1 of Pregnancy zone protein | PZP |
| GSFALSFPVESDVAPIAR | IPI00878729, IPI00789547, IPI00025426, IPI00748437, IPI00478003, IPI00922117, IPI00796830, IPI00884981 | Isoform 1 of Pregnancy zone protein | PZP |
| HNVYINGITYTPVSSTNEK | IPI00878729, IPI00789547, IPI00025426, IPI00748437, IPI00478003, IPI00922117, IPI00796830, IPI00884981 | Isoform 1 of Pregnancy zone protein | PZP |
| HNVYINGITYTPVSSTNEKDMYSFLEDMGLK | IPI00878729, IPI00789547, IPI00025426, IPI00748437, IPI00478003, IPI00922117, IPI00796830, IPI00884981 | Isoform 1 of Pregnancy zone protein | PZP |
| IISIMDEK | IPI00878729, IPI00789547, IPI00025426, IPI00748437, IPI00478003, IPI00922117, IPI00796830, IPI00884981 | Isoform 1 of Pregnancy zone protein | PZP |
| IQHPFTVEEFVLPK | IPI00878729, IPI00789547, IPI00025426, IPI00748437, IPI00478003, IPI00922117, IPI00796830, IPI00884981 | Isoform 1 of Pregnancy zone protein | PZP |
| KLSFYYLIMAK | IPI00878729, IPI00789547, IPI00025426, IPI00748437, IPI00478003, IPI00922117, IPI00796830, IPI00884981 | Isoform 1 of Pregnancy zone protein | PZP |
| LEAGINQLSFPLSSEPIQGSYR | IPI00878729, IPI00789547, IPI00025426, IPI00748437, IPI00478003, IPI00922117, IPI00796830, IPI00884981 | Isoform 1 of Pregnancy zone protein | PZP |
| LLIYAVLPTGDVIGDSAK | IPI00878729, IPI00789547, IPI00025426, IPI00748437, IPI00478003, IPI00922117, IPI00796830, IPI00884981 | Isoform 1 of Pregnancy zone protein | PZP |
| LSFYYLIMAK | IPI00878729, IPI00789547, IPI00025426, IPI00748437, IPI00478003, IPI00922117, IPI00796830, IPI00884981 | Isoform 1 of Pregnancy zone protein | PZP |
| MCPQLQQYEMHGPEGLR | IPI00878729, IPI00789547, IPI00025426, IPI00748437, IPI00478003, IPI00922117, IPI00796830, IPI00884981 | Isoform 1 of Pregnancy zone protein | PZP |
| MFIFAILPDGEVVGDSEK | IPI00878729, IPI00789547, IPI00025426, IPI00748437, IPI00478003, IPI00922117, IPI00796830, IPI00884981 | Isoform 1 of Pregnancy zone protein | PZP |
| MVSGFIPLKPTVK | IPI00878729, IPI00789547, IPI00025426, IPI00748437, IPI00478003, IPI00922117, IPI00796830, IPI00884981 | Isoform 1 of Pregnancy zone protein | PZP |
| NEDSLVFVQTDK | IPI00878729, IPI00789547, IPI00025426, IPI00748437, IPI00478003, IPI00922117, IPI00796830, IPI00884981 | Isoform 1 of Pregnancy zone protein | PZP |
| NELIPLIYLENPR | IPI00878729, IPI00789547, IPI00025426, IPI00748437, IPI00478003, IPI00922117, IPI00796830, IPI00884981 | Isoform 1 of Pregnancy zone protein | PZP |
| NQGNTWLTAFVLK | IPI00878729, IPI00789547, IPI00025426, IPI00748437, IPI00478003, IPI00922117, IPI00796830, IPI00884981 | Isoform 1 of Pregnancy zone protein | PZP |
| NQGNTWLTAFVLKTFAQAR | IPI00878729, IPI00789547, IPI00025426, IPI00748437, IPI00478003, IPI00922117, IPI00796830, IPI00884981 | Isoform 1 of Pregnancy zone protein | PZP |
| QGIPFFAQVLLVDGK | IPI00878729, IPI00789547, IPI00025426, IPI00748437, IPI00478003, IPI00922117, IPI00796830, IPI00884981 | Isoform 1 of Pregnancy zone protein | PZP |
| SDIAPVAR | IPI00878729, IPI00789547, IPI00025426, IPI00748437, IPI00478003, IPI00922117, IPI00796830, IPI00884981 | Isoform 1 of Pregnancy zone protein | PZP |
| SIYKPGQTVK | IPI00878729, IPI00789547, IPI00025426, IPI00748437, IPI00478003, IPI00922117, IPI00796830, IPI00884981 | Isoform 1 of Pregnancy zone protein | PZP |
| SLFTDLEAENDVLHCVAFAVPK | IPI00878729, IPI00789547, IPI00025426, IPI00748437, IPI00478003, IPI00922117, IPI00796830, IPI00884981 | Isoform 1 of Pregnancy zone protein | PZP |
| SSGSLLNNAIK | IPI00878729, IPI00789547, IPI00025426, IPI00748437, IPI00478003, IPI00922117, IPI00796830, IPI00884981 | Isoform 1 of Pregnancy zone protein | PZP |
| SSSNEEVMFLTVQVK | IPI00878729, IPI00789547, IPI00025426, IPI00748437, IPI00478003, IPI00922117, IPI00796830, IPI00884981 | Isoform 1 of Pregnancy zone protein | PZP |
| TGTHGLLVK | IPI00878729, IPI00789547, IPI00025426, IPI00748437, IPI00478003, IPI00922117, IPI00796830, IPI00884981 | Isoform 1 of Pregnancy zone protein | PZP |
| TLLVEAEGIEQEK | IPI00878729, IPI00789547, IPI00025426, IPI00748437, IPI00478003, IPI00922117, IPI00796830, IPI00884981 | Isoform 1 of Pregnancy zone protein | PZP |
| VDLSFSPSQSLPASHAHLR | IPI00878729, IPI00789547, IPI00025426, IPI00748437, IPI00478003, IPI00922117, IPI00796830, IPI00884981 | Isoform 1 of Pregnancy zone protein | PZP |
| VQTVPQTCDGHK | IPI00878729, IPI00789547, IPI00025426, IPI00748437, IPI00478003, IPI00922117, IPI00796830, IPI00884981 | Isoform 1 of Pregnancy zone protein | PZP |
| VTAAPQSVCALR | IPI00878729, IPI00789547, IPI00025426, IPI00748437, IPI00478003, IPI00922117, IPI00796830, IPI00884981 | Isoform 1 of Pregnancy zone protein | PZP |
| VVSVDENFRPR | IPI00878729, IPI00789547, IPI00025426, IPI00748437, IPI00478003, IPI00922117, IPI00796830, IPI00884981 | Isoform 1 of Pregnancy zone protein | PZP |
| YDVENCLANK | IPI00878729, IPI00789547, IPI00025426, IPI00748437, IPI00478003, IPI00922117, IPI00796830, IPI00884981 | Isoform 1 of Pregnancy zone protein | PZP |
| YGAATFTR | IPI00878729, IPI00789547, IPI00025426, IPI00748437, IPI00478003, IPI00922117, IPI00796830, IPI00884981 | Isoform 1 of Pregnancy zone protein | PZP |
| DLFHCVSFTLPR | IPI00025426 | Pregnancy zone protein precursor | PZP |
| SLFTDLVAEK | IPI00025426 | Pregnancy zone protein precursor | PZP |
| AAPGQEPPEHMAELQR | IPI00003590 | Isoform 1 of Sulfhydryl oxidase 1 precursor | QSCN6 |
| AHFSPSNIILDFPAAGSAAR | IPI00465016, IPI00015916, IPI00872013, IPI00645742, IPI00003590 | Isoform 1 of Sulfhydryl oxidase 1 precursor | QSCN6 |
| AWRPALYLAALDCAEETNSAVCR | IPI00465016, IPI00015916, IPI00872013, IPI00645742, IPI00003590 | Isoform 1 of Sulfhydryl oxidase 1 precursor | QSCN6 |
| DCASHFEQMAAASMHR | IPI00465016, IPI00015916, IPI00872013, IPI00645742, IPI00003590 | Isoform 1 of Sulfhydryl oxidase 1 precursor | QSCN6 |
| DFNIPGFPTVR | IPI00465016, IPI00015916, IPI00872013, IPI00645742, IPI00003590 | Isoform 1 of Sulfhydryl oxidase 1 precursor | QSCN6 |
| DVQNVAAAPELAMGALELESR | IPI00465016, IPI00015916, IPI00872013, IPI00645742, IPI00003590 | Isoform 1 of Sulfhydryl oxidase 1 precursor | QSCN6 |
| FGVTDFPSCYLLFR | IPI00465016, IPI00015916, IPI00872013, IPI00645742, IPI00003590 | Isoform 1 of Sulfhydryl oxidase 1 precursor | QSCN6 |
| FPVLEGQR | IPI00465016, IPI00015916, IPI00872013, IPI00645742, IPI00003590 | Isoform 1 of Sulfhydryl oxidase 1 precursor | QSCN6 |
| GYVHYFFGCR | IPI00465016, IPI00015916, IPI00872013, IPI00645742, IPI00003590 | Isoform 1 of Sulfhydryl oxidase 1 precursor | QSCN6 |
| IEVGRFPVLEGQR | IPI00465016, IPI00015916, IPI00872013, IPI00645742, IPI00003590 | Isoform 1 of Sulfhydryl oxidase 1 precursor | QSCN6 |
| IYMADLESALHYILR | IPI00465016, IPI00015916, IPI00872013, IPI00645742, IPI00003590 | Isoform 1 of Sulfhydryl oxidase 1 precursor | QSCN6 |
| KFGVTDFPSCYLLFR | IPI00465016, IPI00015916, IPI00872013, IPI00645742, IPI00003590 | Isoform 1 of Sulfhydryl oxidase 1 precursor | QSCN6 |
| KVNWIGCQGSEPHFR | IPI00465016, IPI00015916, IPI00872013, IPI00645742, IPI00003590 | Isoform 1 of Sulfhydryl oxidase 1 precursor | QSCN6 |
| LAGAPSEDPQFPK | IPI00465016, IPI00015916, IPI00872013, IPI00645742, IPI00003590 | Isoform 1 of Sulfhydryl oxidase 1 precursor | QSCN6 |
| LDVPVWDVEATLNFLK | IPI00465016, IPI00015916, IPI00872013, IPI00645742, IPI00003590 | Isoform 1 of Sulfhydryl oxidase 1 precursor | QSCN6 |
| LEEIDGFFAR | IPI00465016, IPI00015916, IPI00872013, IPI00645742, IPI00003590 | Isoform 1 of Sulfhydryl oxidase 1 precursor | QSCN6 |
| LWGPLEVR | IPI00465016, IPI00015916, IPI00872013, IPI00645742, IPI00003590 | Isoform 1 of Sulfhydryl oxidase 1 precursor | QSCN6 |
| NGSGAVFPVAGADVQTLR | IPI00465016, IPI00015916, IPI00872013, IPI00645742, IPI00003590 | Isoform 1 of Sulfhydryl oxidase 1 precursor | QSCN6 |
| NKIPYSFFK | IPI00465016, IPI00015916, IPI00872013, IPI00645742, IPI00003590 | Isoform 1 of Sulfhydryl oxidase 1 precursor | QSCN6 |
| NNEEYLALIFEK | IPI00465016, IPI00015916, IPI00872013, IPI00645742, IPI00003590 | Isoform 1 of Sulfhydryl oxidase 1 precursor | QSCN6 |
| RDVQNVAAAPELAMGALELESR | IPI00465016, IPI00015916, IPI00872013, IPI00645742, IPI00003590 | Isoform 1 of Sulfhydryl oxidase 1 precursor | QSCN6 |
| VGSPNAAVLWLWSSHNR | IPI00465016, IPI00015916, IPI00872013, IPI00645742, IPI00003590 | Isoform 1 of Sulfhydryl oxidase 1 precursor | QSCN6 |
| VLNTEANVVR | IPI00465016, IPI00015916, IPI00872013, IPI00645742, IPI00003590 | Isoform 1 of Sulfhydryl oxidase 1 precursor | QSCN6 |
| VPVLMESR | IPI00465016, IPI00015916, IPI00872013, IPI00645742, IPI00003590 | Isoform 1 of Sulfhydryl oxidase 1 precursor | QSCN6 |
| GLQVALEEFHK | IPI00019176 | Retinoic acid receptor responder protein 2 precursor | RARRES2 |
| HPPVQWAFQETSVESAVDTPFPAGIFVR | IPI00019176 | Retinoic acid receptor responder protein 2 precursor | RARRES2 |
| LVHCPIETQVLR | IPI00019176 | Retinoic acid receptor responder protein 2 precursor | RARRES2 |
| FSGTWYAMAK | IPI00844536, IPI00480192, IPI00022420 | Plasma retinol-binding protein precursor | RBP4 |
| LIVHNGYCDGR | IPI00844536, IPI00480192, IPI00022420 | Plasma retinol-binding protein precursor | RBP4 |
| LLNLDGTCADSYSFVFSR | IPI00844536, IPI00480192, IPI00022420 | Plasma retinol-binding protein precursor | RBP4 |
| QEELCLAR | IPI00844536, IPI00480192, IPI00022420 | Plasma retinol-binding protein precursor | RBP4 |
| YWGVASFLQK | IPI00844536, IPI00480192, IPI00022420 | Plasma retinol-binding protein precursor | RBP4 |
| ESGTDDFNVWIGLHDPK | IPI00009027 | Lithostathine 1 alpha precursor | REG1A |
| AGGEFLLR | IPI00796170, IPI00937516, IPI00646161, IPI00943101, IPI00879688 | renin binding protein | RENBP |
| CKPVNTFVHEPLVDVQNVCFQEK | IPI00014048 | Ribonuclease pancreatic precursor | RNASE1 |
| HIIVACEGSPYVPVHFDASVEDST | IPI00014048 | Ribonuclease pancreatic precursor | RNASE1 |
| YAQTPANMFYIVACDNR | IPI00289860, IPI00019449 | Non-secretory ribonuclease precursor | RNASE2 |
| FNTFIHEDIWNIR | IPI00029699 | Ribonuclease 4 precursor | RNASE4 |
| RVVIACEGNPQVPVHFDG | IPI00029699 | Ribonuclease 4 precursor | RNASE4 |
| VVIACEGNPQVPVHFDG | IPI00029699 | Ribonuclease 4 precursor | RNASE4 |
| YCNLMMQR | IPI00029699 | Ribonuclease 4 precursor | RNASE4 |
| MLLPSGSLFFLR | IPI00420043, IPI00936239, IPI00219798, IPI00939730, IPI00941618, IPI00740934, IPI00385980, IPI00896492, IPI00418122, IPI00900311, IPI00829739, IPI00418121 | Isoform 1 of Roundabout homolog 1 | ROBO1 |
| EDFQIQPR | IPI00418145, IPI00103871, IPI00179778, IPI00418144 | Isoform 1 of Roundabout homolog 4 precursor | ROBO4 |
| VSGPAAPAQSYTALFR | IPI00418145, IPI00103871, IPI00179778, IPI00418144 | Isoform 1 of Roundabout homolog 4 precursor | ROBO4 |
| VSIQEPQDYTEPVELLAVR | IPI00418145, IPI00103871, IPI00179778, IPI00418144 | Isoform 1 of Roundabout homolog 4 precursor | ROBO4 |
| LVEGEVYAVDER | IPI00900332 | AIG2-like domain-containing protein 1 | RP11-151A6.2 |
| IQDKEGIPPDQQR | IPI00654754, IPI00798127, IPI00789107, IPI00719280, IPI00796007, IPI00793810, IPI00794205, IPI00784990, IPI00936175, IPI00790633, IPI00797400, IPI00792712, IPI00418813, IPI00794211, IPI00793729, IPI00792139, IPI00796600, IPI00795527, IPI00456429, IPI00879555, IPI00798155, IPI00793330, IPI00794925, IPI00179330, IPI00937730, IPI00789823 | ubiquitin and ribosomal protein S27a precursor | RPS27A |
| GPLPAAPPVAPER | IPI00021766, IPI00478442, IPI00298289 | Isoform 1 of Reticulon-4 | RTN4 |
| HLQALEELDLGDNR | IPI00328746, IPI00787628 | Reticulon-4 receptor-like 2 precursor | RTN4RL2 |
| LFLQNNLIR | IPI00328746, IPI00787628 | Reticulon-4 receptor-like 2 precursor | RTN4RL2 |
| LLTEHVFR | IPI00328746 | Reticulon-4 receptor-like 2 precursor | RTN4RL2 |
| SLEPDTFQGLER | IPI00328746, IPI00787628 | Reticulon-4 receptor-like 2 precursor | RTN4RL2 |
| VSSSDVTCATPPER | IPI00328746 | Reticulon-4 receptor-like 2 precursor | RTN4RL2 |
| LQDAEIAR | IPI00027463 | Protein S100-A6 | S100A6 |
| GNFHAVYR | IPI00007047 | Protein S100-A8 | S100A8 |
| LLETECPQYIR | IPI00007047 | Protein S100-A8 | S100A8 |
| MLTELEK | IPI00007047 | Protein S100-A8 | S100A8 |
| KDLQNFLK | IPI00939362, IPI00027462 | Protein S100-A9 | S100A9 |
| LGHPDTLNQGEFK | IPI00939362, IPI00027462 | Protein S100-A9 | S100A9 |
| NIETIINTFHQYSVK | IPI00939362, IPI00027462 | Protein S100-A9 | S100A9 |
| QLSFEEFIMLMAR | IPI00027462 | Protein S100-A9 | S100A9 |
| SFFSFLGEAFDGAR | IPI00895943, IPI00552578, IPI00006146 | serum amyloid A2 isoform a | SAA2 |
| FRPDGLPK | IPI00019399 | Serum amyloid A-4 protein precursor | SAA4 |
| LVDTLPQKPR | IPI00006705 | Uteroglobin precursor | SCGB1A1 |
| VTLEQDSR | IPI00023442 | Isoform 1 of Protein transport protein Sec31B | SEC31L2 |
| EEIVYLPCIYR | IPI00943000, IPI00852953, IPI00852838, IPI00030385, IPI00892906, IPI00942368, IPI00853528, IPI00852768, IPI00012303 | Selenium binding protein 1, isoform CRA_b | SELENBP1 |
| LVLPSLISSR | IPI00943000, IPI00852953, IPI00852838, IPI00030385, IPI00892906, IPI00942368, IPI00853528, IPI00852768, IPI00012303 | Selenium binding protein 1, isoform CRA_b | SELENBP1 |
| SLTEEAENWGDGEPNNK | IPI00218795 | L-selectin precursor | SELL |
| SLTEEAENWGDGEPNNKK | IPI00218795 | L-selectin precursor | SELL |
| SYYWIGIR | IPI00643835, IPI00646956, IPI00514728, IPI00640405, IPI00514521, IPI00645768, IPI00514796, IPI00514233, IPI00644144, IPI00218795, IPI00296542, IPI00645234, IPI00295339, IPI00514342 | L-selectin precursor | SELL |
| TICESSGIWSNPSPICQK | IPI00218795 | L-selectin precursor | SELL |
| LFLGGLDALYSLR | IPI00792373, IPI00024570 | Semaphorin-3G precursor | SEMA3G |
| MLLLQPQAR | IPI00790523, IPI00513964, IPI00419724 | semaphorin 4B precursor | SEMA4B |
| CINQLLCK | IPI00847381, IPI00029061 | Selenoprotein P | SEPP1 |
| DDFLIYDR | IPI00847381, IPI00029061 | Selenoprotein P | SEPP1 |
| DMPASEDLQDLQK | IPI00847381, IPI00029061 | Selenoprotein P | SEPP1 |
| LPTDSELAPR | IPI00847381, IPI00029061 | Selenoprotein P | SEPP1 |
| LVYHLGLPFSFLTFPYVEEAIK | IPI00847381, IPI00029061 | Selenoprotein P | SEPP1 |
| QPPAWSIR | IPI00847381, IPI00029061 | Selenoprotein P | SEPP1 |
| HLIFEK | IPI00847381 | selenoprotein P isoform 2 | SEPP1 |
| AVLTIDEK | IPI00790784, IPI00305457, IPI00736763, IPI00553177, IPI00869004 | Alpha-antitrypsin | SERPINA1 |
| DTEEEDFHVDQVTTVK | IPI00790784, IPI00305457, IPI00736763, IPI00553177, IPI00869004 | Alpha-antitrypsin | SERPINA1 |
| ELDRDTVFALVNYIFFK | IPI00790784, IPI00305457, IPI00736763, IPI00553177, IPI00869004 | Alpha-antitrypsin | SERPINA1 |
| FLENEDRR | IPI00790784, IPI00305457, IPI00736763, IPI00553177, IPI00869004 | Alpha-antitrypsin | SERPINA1 |
| FNKPFVFLMIEQNTK | IPI00790784, IPI00305457, IPI00736763, IPI00553177, IPI00869004 | Alpha-antitrypsin | SERPINA1 |
| GKWERPFEVK | IPI00790784, IPI00305457, IPI00736763, IPI00553177, IPI00869004 | Alpha-antitrypsin | SERPINA1 |
| GTEAAGAMFLEAIPMSIPPEVK | IPI00790784, IPI00305457, IPI00736763, IPI00553177, IPI00869004 | Alpha-antitrypsin | SERPINA1 |
| ITPNLAEFAFSLYR | IPI00790784, IPI00305457, IPI00736763, IPI00553177, IPI00869004 | Alpha-antitrypsin | SERPINA1 |
| KLSSWVLLMK | IPI00790784, IPI00305457, IPI00736763, IPI00553177, IPI00869004 | Alpha-antitrypsin | SERPINA1 |
| KLYHSEAFTVNFGDTEEAK | IPI00790784, IPI00305457, IPI00736763, IPI00553177, IPI00869004 | Alpha-antitrypsin | SERPINA1 |
| KQINDYVEK | IPI00790784, IPI00305457, IPI00736763, IPI00553177, IPI00869004 | Alpha-antitrypsin | SERPINA1 |
| LGMFNIQHCK | IPI00790784, IPI00305457, IPI00736763, IPI00553177, IPI00869004 | Alpha-antitrypsin | SERPINA1 |
| LQHLENELTHDIITK | IPI00790784, IPI00305457, IPI00736763, IPI00553177, IPI00869004 | Alpha-antitrypsin | SERPINA1 |
| LSITGTYDLK | IPI00790784, IPI00305457, IPI00736763, IPI00553177, IPI00869004 | Alpha-antitrypsin | SERPINA1 |
| LSSWVLLMK | IPI00790784, IPI00305457, IPI00736763, IPI00553177, IPI00869004 | Alpha-antitrypsin | SERPINA1 |
| LVDKFLEDVK | IPI00790784, IPI00305457, IPI00736763, IPI00553177, IPI00869004 | Alpha-antitrypsin | SERPINA1 |
| LVDKFLEDVKK | IPI00790784, IPI00305457, IPI00736763, IPI00553177, IPI00869004 | Alpha-antitrypsin | SERPINA1 |
| LYHSEAFTVNFGDTEEAK | IPI00790784, IPI00305457, IPI00736763, IPI00553177, IPI00869004 | Alpha-antitrypsin | SERPINA1 |
| QINDYVEK | IPI00790784, IPI00305457, IPI00736763, IPI00553177, IPI00869004 | Alpha-antitrypsin | SERPINA1 |
| SVLGQLGITK | IPI00790784, IPI00305457, IPI00736763, IPI00553177, IPI00869004 | Alpha-antitrypsin | SERPINA1 |
| TLNQPDSQLQLTTGNGLFLSEGLK | IPI00790784, IPI00305457, IPI00736763, IPI00553177, IPI00869004 | Alpha-antitrypsin | SERPINA1 |
| VFSNGADLSGVTEEAPLK | IPI00790784, IPI00305457, IPI00736763, IPI00553177, IPI00869004 | Alpha-antitrypsin | SERPINA1 |
| WERPFEVK | IPI00790784, IPI00305457, IPI00736763, IPI00553177, IPI00869004 | Alpha-antitrypsin | SERPINA1 |
| AWLMASR | IPI00007199 | Protein Z-dependent protease inhibitor precursor | SERPINA10 |
| ETSNFGFSLLR | IPI00007199 | Protein Z-dependent protease inhibitor precursor | SERPINA10 |
| FASTFDK | IPI00007199 | Protein Z-dependent protease inhibitor precursor | SERPINA10 |
| GLHLQALKPTKPGLLPSLFK | IPI00007199 | Protein Z-dependent protease inhibitor precursor | SERPINA10 |
| HDGNMVFSPFGMSLAMTGLMLGATGPTETQIK | IPI00007199 | Protein Z-dependent protease inhibitor precursor | SERPINA10 |
| IFSPFADLSELSATGR | IPI00007199 | Protein Z-dependent protease inhibitor precursor | SERPINA10 |
| LILVDYILFK | IPI00007199 | Protein Z-dependent protease inhibitor precursor | SERPINA10 |
| MGDHLALEDYLTTDLVETWLR | IPI00007199 | Protein Z-dependent protease inhibitor precursor | SERPINA10 |
| NLELGLTQGSFAFIHK | IPI00007199 | Protein Z-dependent protease inhibitor precursor | SERPINA10 |
| NMEVFFPK | IPI00007199 | Protein Z-dependent protease inhibitor precursor | SERPINA10 |
| WLTPFDPVFTEVDTFHLDK | IPI00007199 | Protein Z-dependent protease inhibitor precursor | SERPINA10 |
| WLTPFDPVFTEVDTFHLDKYK | IPI00007199 | Protein Z-dependent protease inhibitor precursor | SERPINA10 |
| YEMHELLR | IPI00007199 | Protein Z-dependent protease inhibitor precursor | SERPINA10 |
| YFDTECVPMNFR | IPI00007199 | Protein Z-dependent protease inhibitor precursor | SERPINA10 |
| ITPTITNFALR | IPI00333828 | Serpin A11 precursor | SERPINA11 |
| QVEAALQPQTLR | IPI00333828 | Serpin A11 precursor | SERPINA11 |
| WGQLLLPSLLDLHLPR | IPI00333828 | Serpin A11 precursor | SERPINA11 |
| ALWEKPFISSR | IPI00328609 | Kallistatin precursor | SERPINA4 |
| ATLDVDEAGTEAAAATSFAIK | IPI00328609 | Kallistatin precursor | SERPINA4 |
| DFYVDENTTVR | IPI00328609 | Kallistatin precursor | SERPINA4 |
| DVLMVLVNYIYFK | IPI00328609 | Kallistatin precursor | SERPINA4 |
| EIEEVLTPEMLMR | IPI00328609 | Kallistatin precursor | SERPINA4 |
| FFSAQTNR | IPI00328609 | Kallistatin precursor | SERPINA4 |
| FSISGSYVLDQILPR | IPI00328609 | Kallistatin precursor | SERPINA4 |
| FYYLIASETPGK | IPI00328609 | Kallistatin precursor | SERPINA4 |
| IAPANADFAFR | IPI00328609 | Kallistatin precursor | SERPINA4 |
| IVDLVSELK | IPI00328609 | Kallistatin precursor | SERPINA4 |
| IVDLVSELKK | IPI00328609 | Kallistatin precursor | SERPINA4 |
| KDVLMVLVNYIYFK | IPI00328609 | Kallistatin precursor | SERPINA4 |
| KLELHLPK | IPI00328609 | Kallistatin precursor | SERPINA4 |
| LELHLPK | IPI00328609 | Kallistatin precursor | SERPINA4 |
| LFHTNFYDTVGTIQLINDHVK | IPI00328609 | Kallistatin precursor | SERPINA4 |
| LGFTDLFSK | IPI00328609 | Kallistatin precursor | SERPINA4 |
| MDYKGDATVFFILPNQGK | IPI00328609 | Kallistatin precursor | SERPINA4 |
| NIFFSPLSISAAYAMLSLGACSHSR | IPI00328609 | Kallistatin precursor | SERPINA4 |
| VGSALFLSHNLK | IPI00328609 | Kallistatin precursor | SERPINA4 |
| WADLSGITK | IPI00328609 | Kallistatin precursor | SERPINA4 |
| WNNLLR | IPI00328609 | Kallistatin precursor | SERPINA4 |
| AAAATGTIFTFR | IPI00917825, IPI00007221 | Plasma serine protease inhibitor | SERPINA5 |
| AVVEVDESGTR | IPI00917825, IPI00007221 | Plasma serine protease inhibitor | SERPINA5 |
| FSIEGSYQLEK | IPI00917825, IPI00007221 | Plasma serine protease inhibitor | SERPINA5 |
| MQQVENGLSEK | IPI00917825, IPI00007221 | Plasma serine protease inhibitor | SERPINA5 |
| QINDYVAK | IPI00917825, IPI00007221 | Plasma serine protease inhibitor | SERPINA5 |
| QLELYLPK | IPI00917825, IPI00007221 | Plasma serine protease inhibitor | SERPINA5 |
| TLYLADTFPTNFR | IPI00917825, IPI00007221 | Plasma serine protease inhibitor | SERPINA5 |
| DFTFDLYR | IPI00007221 | Plasma serine protease inhibitor precursor | SERPINA5 |
| GFQQLLQELNQPR | IPI00007221 | Plasma serine protease inhibitor precursor | SERPINA5 |
| MQILEGLGLNLQK | IPI00007221 | Plasma serine protease inhibitor precursor | SERPINA5 |
| RDFTFDLYR | IPI00007221 | Plasma serine protease inhibitor precursor | SERPINA5 |
| AVLQLNEEGVDTAGSTGVTLNLTSKPIILR | IPI00027482 | Corticosteroid-binding globulin precursor | SERPINA6 |
| EENFYVDETTVVK | IPI00027482 | Corticosteroid-binding globulin precursor | SERPINA6 |
| GLASANVDFAFSLYK | IPI00550991, IPI00847635, IPI00607870, IPI00027482 | Corticosteroid-binding globulin precursor | SERPINA6 |
| GTHVDLGLASANVDFAFSLYK | IPI00550991, IPI00847635, IPI00607870, IPI00027482 | Corticosteroid-binding globulin precursor | SERPINA6 |
| GTWTQPFDLASTR | IPI00027482 | Corticosteroid-binding globulin precursor | SERPINA6 |
| HLVALSPK | IPI00027482 | Corticosteroid-binding globulin precursor | SERPINA6 |
| HYYESEVLAMNFQDWATASR | IPI00027482 | Corticosteroid-binding globulin precursor | SERPINA6 |
| ITQDAQLK | IPI00027482 | Corticosteroid-binding globulin precursor | SERPINA6 |
| KNIFISPVSISMALAMLSLGTCGHTR | IPI00027482 | Corticosteroid-binding globulin precursor | SERPINA6 |
| MNTVIAALSR | IPI00027482 | Corticosteroid-binding globulin precursor | SERPINA6 |
| NIFISPVSISMALAMLSLGTCGHTR | IPI00027482 | Corticosteroid-binding globulin precursor | SERPINA6 |
| QINSYVK | IPI00027482 | Corticosteroid-binding globulin precursor | SERPINA6 |
| WSAGLTSSQVDLYIPK | IPI00027482 | Corticosteroid-binding globulin precursor | SERPINA6 |
| AQWANPFDPSK | IPI00292946 | Thyroxine-binding globulin precursor | SERPINA7 |
| AQWANPFDPSKTEDSSSFLIDK | IPI00292946 | Thyroxine-binding globulin precursor | SERPINA7 |
| AVLHIGEK | IPI00292946 | Thyroxine-binding globulin precursor | SERPINA7 |
| EGQMESVEAAMSSK | IPI00292946 | Thyroxine-binding globulin precursor | SERPINA7 |
| FAFNLYR | IPI00292946 | Thyroxine-binding globulin precursor | SERPINA7 |
| FLNDVK | IPI00292946 | Thyroxine-binding globulin precursor | SERPINA7 |
| FSISATYDLGATLLK | IPI00292946 | Thyroxine-binding globulin precursor | SERPINA7 |
| GTEAAAVPEVELSDQPENTFLHPIIQIDR | IPI00292946 | Thyroxine-binding globulin precursor | SERPINA7 |
| GWVDLFVPK | IPI00292946 | Thyroxine-binding globulin precursor | SERPINA7 |
| KELELQIGNALFIGK | IPI00292946 | Thyroxine-binding globulin precursor | SERPINA7 |
| LSNAAHK | IPI00292946 | Thyroxine-binding globulin precursor | SERPINA7 |
| MGIQHAYSENADFSGLTEDNGLK | IPI00292946 | Thyroxine-binding globulin precursor | SERPINA7 |
| MSSINADFAFNLYR | IPI00292946 | Thyroxine-binding globulin precursor | SERPINA7 |
| NALALFVLPK | IPI00292946 | Thyroxine-binding globulin precursor | SERPINA7 |
| QEINSHVEMQTK | IPI00292946 | Thyroxine-binding globulin precursor | SERPINA7 |
| SFMLLILER | IPI00292946 | Thyroxine-binding globulin precursor | SERPINA7 |
| SILFLGK | IPI00292946 | Thyroxine-binding globulin precursor | SERPINA7 |
| STRSILFLGKVVNPTEA | IPI00292946 | Thyroxine-binding globulin precursor | SERPINA7 |
| TEDSSSFLIDK | IPI00292946 | Thyroxine-binding globulin precursor | SERPINA7 |
| TLYETEVFSTDFSNISAAK | IPI00292946 | Thyroxine-binding globulin precursor | SERPINA7 |
| VVGLIQDLKPNTIMVLVNYIHFK | IPI00292946 | Thyroxine-binding globulin precursor | SERPINA7 |
| ADGESCSASMMYQEGK | IPI00032179 | Antithrombin III variant | SERPINC1 |
| AFLEVNEEGSEAAASTAVVIAGR | IPI00032179, IPI00844156 | Antithrombin III variant | SERPINC1 |
| ANRPFLVFIR | IPI00032179, IPI00844156 | Antithrombin III variant | SERPINC1 |
| ATEDEGSEQKIPEATNR | IPI00032179, IPI00844156 | Antithrombin III variant | SERPINC1 |
| EQLQDMGLVDLFSPEK | IPI00032179 | Antithrombin III variant | SERPINC1 |
| EVPLNTIIFMGR | IPI00032179, IPI00844156 | Antithrombin III variant | SERPINC1 |
| LQPLDFKENAEQSR | IPI00032179 | Antithrombin III variant | SERPINC1 |
| NDNDNIFLSPLSISTAFAMTK | IPI00032179, IPI00844156 | Antithrombin III variant | SERPINC1 |
| DALENIDPATQMMILNCIYFK | IPI00879573, IPI00292950 | Serpin peptidase inhibitor, clade D (Heparin cofactor), member 1 | SERPIND1 |
| EYYFAEAQIADFSDPAFISK | IPI00879573, IPI00292950 | Serpin peptidase inhibitor, clade D (Heparin cofactor), member 1 | SERPIND1 |
| FTVDRPFLFLIYEHR | IPI00879573, IPI00292950 | Serpin peptidase inhibitor, clade D (Heparin cofactor), member 1 | SERPIND1 |
| GGETAQSADPQWEQLNNK | IPI00879573, IPI00292950 | Serpin peptidase inhibitor, clade D (Heparin cofactor), member 1 | SERPIND1 |
| GPLDQLEK | IPI00879573, IPI00292950 | Serpin peptidase inhibitor, clade D (Heparin cofactor), member 1 | SERPIND1 |
| HQGTITVNEEGTQATTVTTVGFMPLSTQVR | IPI00879573, IPI00292950 | Serpin peptidase inhibitor, clade D (Heparin cofactor), member 1 | SERPIND1 |
| MLFDKNGNMAGISDQR | IPI00879573, IPI00292950 | Serpin peptidase inhibitor, clade D (Heparin cofactor), member 1 | SERPIND1 |
| NYNLVESLK | IPI00879573, IPI00292950 | Serpin peptidase inhibitor, clade D (Heparin cofactor), member 1 | SERPIND1 |
| QFPILLDFK | IPI00879573, IPI00292950 | Serpin peptidase inhibitor, clade D (Heparin cofactor), member 1 | SERPIND1 |
| RNFGYTLR | IPI00879573, IPI00292950 | Serpin peptidase inhibitor, clade D (Heparin cofactor), member 1 | SERPIND1 |
| SVNDLYIQK | IPI00879573, IPI00292950 | Serpin peptidase inhibitor, clade D (Heparin cofactor), member 1 | SERPIND1 |
| TSCLLFMGR | IPI00879573, IPI00292950 | Serpin peptidase inhibitor, clade D (Heparin cofactor), member 1 | SERPIND1 |
| YEITTIHNLFR | IPI00879573, IPI00292950 | Serpin peptidase inhibitor, clade D (Heparin cofactor), member 1 | SERPIND1 |
| LVQGFMPHFFR | IPI00926949, IPI00007118 | Plasminogen activator inhibitor 1 | SERPINE1 |
| ALYYDLISSPDIHGTYK | IPI00006114 | Pigment epithelium-derived factor precursor | SERPINF1 |
| DTDTGALLFIGK | IPI00796279, IPI00006114, IPI00790473 | Pigment epithelium-derived factor precursor | SERPINF1 |
| EIPDEISILLLGVAHFK | IPI00006114 | Pigment epithelium-derived factor precursor | SERPINF1 |
| ELLDTVTAPQK | IPI00006114 | Pigment epithelium-derived factor precursor | SERPINF1 |
| GQWVTK | IPI00006114 | Pigment epithelium-derived factor precursor | SERPINF1 |
| IAQLPLTGSMSIIFFLPLK | IPI00796279, IPI00006114, IPI00790473 | Pigment epithelium-derived factor precursor | SERPINF1 |
| KTSLEDFYLDEER | IPI00796279, IPI00006114, IPI00790473 | Pigment epithelium-derived factor precursor | SERPINF1 |
| LAAAVSNFGYDLYR | IPI00796279, IPI00006114, IPI00790473 | Pigment epithelium-derived factor precursor | SERPINF1 |
| LDLQEINNWVQAQMK | IPI00006114 | Pigment epithelium-derived factor precursor | SERPINF1 |
| LQSLFDSPDFSK | IPI00796279, IPI00006114, IPI00790473 | Pigment epithelium-derived factor precursor | SERPINF1 |
| LSYEGEVTK | IPI00796279, IPI00006114, IPI00790473 | Pigment epithelium-derived factor precursor | SERPINF1 |
| SSFVAPLEK | IPI00006114 | Pigment epithelium-derived factor precursor | SERPINF1 |
| SSMSPTTNVLLSPLSVATALSALSLGAEQR | IPI00006114 | Pigment epithelium-derived factor precursor | SERPINF1 |
| TSLEDFYLDEER | IPI00796279, IPI00006114, IPI00790473 | Pigment epithelium-derived factor precursor | SERPINF1 |
| TVQAVLTVPK | IPI00796279, IPI00006114, IPI00790473 | Pigment epithelium-derived factor precursor | SERPINF1 |
| VPMMSDPK | IPI00796279, IPI00006114, IPI00790473 | Pigment epithelium-derived factor precursor | SERPINF1 |
| VRSSMSPTTNVLLSPLSVATALSALSLGAEQR | IPI00006114 | Pigment epithelium-derived factor precursor | SERPINF1 |
| YGLDSDLSCK | IPI00796279, IPI00006114, IPI00790473 | Pigment epithelium-derived factor precursor | SERPINF1 |
| DSFHLDEQFTVPVEMMQAR | IPI00879231, IPI00879608, IPI00029863, IPI00877925, IPI00879937 | Alpha-2 antiplasmin | SERPINF2 |
| FDPSLTQR | IPI00879231, IPI00879608, IPI00029863, IPI00877925, IPI00879937 | Alpha-2 antiplasmin | SERPINF2 |
| GFPIKEDFLEQSEQLFGAKPVSLTGK | IPI00879231, IPI00879608, IPI00029863, IPI00877925, IPI00879937 | Alpha-2 antiplasmin | SERPINF2 |
| HQMDLVATLSQLGLQELFQAPDLR | IPI00879231, IPI00879608, IPI00029863, IPI00877925, IPI00879937 | Alpha-2 antiplasmin | SERPINF2 |
| LCQDLGPGAFR | IPI00879231, IPI00879608, IPI00029863, IPI00877925, IPI00879937 | Alpha-2 antiplasmin | SERPINF2 |
| LGNQEPGGQTALK | IPI00879231, IPI00879608, IPI00029863, IPI00877925, IPI00879937 | Alpha-2 antiplasmin | SERPINF2 |
| NKFDPSLTQR | IPI00879231, IPI00879608, IPI00029863, IPI00877925, IPI00879937 | Alpha-2 antiplasmin | SERPINF2 |
| QEDDLANINQWVK | IPI00879231, IPI00879608, IPI00029863, IPI00877925, IPI00879937 | Alpha-2 antiplasmin | SERPINF2 |
| QLTSGPNQEQVSPLTLLK | IPI00879231, IPI00879608, IPI00029863, IPI00877925, IPI00879937 | Alpha-2 antiplasmin | SERPINF2 |
| WFLLEQPEIQVAHFPFK | IPI00879231, IPI00879608, IPI00029863, IPI00877925, IPI00879937 | Alpha-2 antiplasmin | SERPINF2 |
| DFTCVHQALK | IPI00556459, IPI00877698, IPI00479178, IPI00877850, IPI00879931, IPI00291866, IPI00879796 | Plasma protease C1 inhibitor | SERPING1 |
| FQPTLLTLPR | IPI00556459, IPI00877698, IPI00479178, IPI00877850, IPI00879931, IPI00291866, IPI00879796 | Plasma protease C1 inhibitor | SERPING1 |
| GVTSVSQIFHSPDLAIR | IPI00556459, IPI00877698, IPI00479178, IPI00877850, IPI00879931, IPI00291866, IPI00879796 | Plasma protease C1 inhibitor | SERPING1 |
| KYPVAHFIDQTLK | IPI00556459, IPI00877698, IPI00479178, IPI00877850, IPI00879931, IPI00291866, IPI00879796 | Plasma protease C1 inhibitor | SERPING1 |
| LEDMEQALSPSVFK | IPI00556459, IPI00877698, IPI00479178, IPI00877850, IPI00879931, IPI00291866, IPI00879796 | Plasma protease C1 inhibitor | SERPING1 |
| LLDSLPSDTR | IPI00556459, IPI00877698, IPI00479178, IPI00877850, IPI00879931, IPI00291866, IPI00879796 | Plasma protease C1 inhibitor | SERPING1 |
| LVLLNAIYLSAK | IPI00556459, IPI00877698, IPI00479178, IPI00877850, IPI00879931, IPI00291866, IPI00879796 | Plasma protease C1 inhibitor | SERPING1 |
| LYHAFSAMK | IPI00556459, IPI00877698, IPI00479178, IPI00877850, IPI00879931, IPI00291866, IPI00879796 | Plasma protease C1 inhibitor | SERPING1 |
| TLLVFEVQQPFLFVLWDQQHK | IPI00556459, IPI00877698, IPI00479178, IPI00877850, IPI00879931, IPI00291866, IPI00879796 | Plasma protease C1 inhibitor | SERPING1 |
| TNLESILSYPK | IPI00556459, IPI00877698, IPI00479178, IPI00877850, IPI00879931, IPI00291866, IPI00879796 | Plasma protease C1 inhibitor | SERPING1 |
| VATTVISK | IPI00556459, IPI00877698, IPI00479178, IPI00877850, IPI00879931, IPI00291866, IPI00879796 | Plasma protease C1 inhibitor | SERPING1 |
| VTTSQDMLSIMEK | IPI00556459, IPI00877698, IPI00479178, IPI00877850, IPI00879931, IPI00291866, IPI00879796 | Plasma protease C1 inhibitor | SERPING1 |
| EAIFQDTMR | IPI00915806, IPI00296083 | Pulmonary surfactant-associated protein B precursor | SFTPB |
| ALALPPLGLAPLLNLWAKPQGR | IPI00219583, IPI00884958, IPI00940099, IPI00929685, IPI00885070, IPI00884913, IPI00885112, IPI00023019 | Isoform 1 of Sex hormone-binding globulin | SHBG |
| DDWFMLGLR | IPI00219583, IPI00884958, IPI00940099, IPI00929685, IPI00885070, IPI00884913, IPI00885112, IPI00023019 | Isoform 1 of Sex hormone-binding globulin | SHBG |
| DIPQPHAEPWAFSLDLGLK | IPI00219583, IPI00884958, IPI00940099, IPI00929685, IPI00885070, IPI00884913, IPI00885112, IPI00023019 | Isoform 1 of Sex hormone-binding globulin | SHBG |
| IALGGLLFPASNLR | IPI00219583, IPI00884958, IPI00940099, IPI00929685, IPI00885070, IPI00884913, IPI00885112, IPI00023019 | Isoform 1 of Sex hormone-binding globulin | SHBG |
| LDVDQALNR | IPI00219583, IPI00884958, IPI00940099, IPI00929685, IPI00885070, IPI00884913, IPI00885112, IPI00023019 | Isoform 1 of Sex hormone-binding globulin | SHBG |
| LFLGALPGEDSSTSFCLNGLWAQGQR | IPI00219583, IPI00884958, IPI00940099, IPI00929685, IPI00885070, IPI00884913, IPI00885112, IPI00023019 | Isoform 1 of Sex hormone-binding globulin | SHBG |
| LPLVPALDGCLR | IPI00219583, IPI00884958, IPI00940099, IPI00929685, IPI00885070, IPI00884913, IPI00885112, IPI00023019 | Isoform 1 of Sex hormone-binding globulin | SHBG |
| MEGDSVLLEVDGEEVLR | IPI00219583, IPI00884958, IPI00940099, IPI00929685, IPI00885070, IPI00884913, IPI00885112, IPI00023019 | Isoform 1 of Sex hormone-binding globulin | SHBG |
[truncated: 380,240 more chars]
